# NCI 7977: A Phase I Dose-Escalation Study of Intermittent Oral ABT-888 (Veliparib) plus Intravenous Irinotecan Administered in Patients with Advanced Solid Tumors

**DOI:** 10.1158/2767-9764.CRC-22-0485

**Published:** 2023-06-26

**Authors:** Michael Cecchini, Zenta Walther, Wei Wei, Navid Hafez, Mary Jo Pilat, Scott A. Boerner, Diane E. Durecki, Joseph P. Eder, Kurt A. Schalper, Alice P. Chen, Patricia LoRusso

**Affiliations:** 1 Department of Internal Medicine (Medical Oncology), Yale University School of Medicine, New Haven, Connecticut.; 2 Department of Pathology, Yale University School of Medicine, New Haven, Connecticut.; 3 Department of Biostatistics, Yale School of Public Health, New Haven, Connecticut.; 4 Barbara Ann Karmanos Cancer Institute, Wayne State University, Detroit, Michigan.; 5 Division of Cancer Treatment and Diagnosis, NCI, Bethesda, Maryland.

## Abstract

**Purpose:**

Veliparib is a PARP inhibitor (PARPi) with activity in *BRCA* 1/2/*PALB2*-deficient tumors. Preclinical observations reveal topoisomerase inhibitors like irinotecan are synergistic with PARPi irrespective of homologous recombination deficiency (HRD), potentially expanding the role for PARPi.

**Experimental Design:**

NCI 7977 was a multicohort phase I clinical trial evaluating the safety and efficacy of multiple dose schedules of veliparib with irinotecan for solid tumors. In the intermittent veliparib cohort, escalating doses of veliparib were given twice daily at dose level (DL) 1 (50 mg) and DL 2 (100 mg) days 1–4 and 8–11 with irinotecan 100 mg/m^2^ days 3 and 10 in 21-day cycles.

**Results:**

Fifteen patients enrolled, 8 of 15 (53%) received ≥4 prior systemic treatments. At DL1, 1 of 6 patients experienced a dose-limiting toxicity (DLT) of diarrhea. At DL2, 9 patients were treated, with 3 unevaluable for DLT, and 2 of 6 evaluable patients experienced a DLT of grade 3 neutropenia. Irinotecan 100 mg/m^2^ and veliparib 50 mg twice daily was the MTD. No objective responses were observed, although 4 patients had progression-free survival >6 months.

**Conclusions:**

The MTD of intermittent veliparib is 50 mg twice daily days 1–4 and 8–11 with weekly irinotecan 100 mg/m^2^ days 3 and 10 every 21 days. Multiple patients experienced prolonged stable disease irrespective of HRD and prior irinotecan. However, due to the toxicities with higher dose intermittent veliparib and irinotecan, this schedule was determined too toxic for further development and the arm was closed prematurely.

**Significance:**

The combination of intermittent veliparib with weekly irinotecan was deemed too toxic for further development. Future PARPi combinations should focus on agents with nonoverlapping toxicities to improve tolerability. The treatment combination showed limited efficacy with prolonged stable disease observed in multiple heavily pretreated patients, but no objective responses were seen.

## Introduction

Multiagent DNA-damaging chemotherapy has been a critical therapeutic tool to treat a wide variety of cancers for decades. With the development of novel targeted therapies, there have been attempts to evaluate rational combinations of targeted agents with chemotherapy. The goal of combination strategies is to enhance efficacy, without increasing the toxicity of the multiagent chemotherapies. Inhibitors of PARP are targeted agents that result in impairment of the base excision repair pathway and have monotherapy activity in tumors with homologous recombination deficiency (HRD) such as breast, ovarian, and pancreatic cancer (1–4). Because of their mechanism, efficacy, and overall tolerability, there are numerous ongoing studies investigating PARP inhibitors (PARPi) in combination with other anticancer agents.

We and others have previously outlined the preclinical data, which demonstrates synergy between PARPis and topoisomerase I inhibitors, both *in vivo* and *in vitro* (5–12). Irinotecan is a camptothecin and inhibitor of topoisomerase I that is used broadly in oncology practice, particularly in cancers of the gastrointestinal tract. While monotherapy PARPis are effective for HRD tumors, novel combinations such as irinotecan with a PARPi present an opportunity to expand the efficacy of PARPis beyond HRD tumors. On the basis of this preliminary data, we designed the NCI 7977 trial to evaluate the combination of veliparib, a PARPi, and irinotecan, an inhibitor of topoisomerase I.

The NCI 7977 trial was a multicohort phase I study evaluating different dosing schedules of veliparib in combination with irinotecan. We previously published the continuous veliparib dosing cohort which revealed a MTD of veliparib 40 mg twice daily administered on days -1 to 14 of a 21-day cycle with irinotecan 100 mg/m^2^ days 3 and 10 (11). A separate cohort of intermittent dosing veliparib (days 1–4 and 8–11 every 21 days) in combination with irinotecan was pursued in an attempt to increase the PARPi dose by using an intermittent dosing schedule for the PARPi and potentially enhance efficacy of the combination without additional toxicity. Here we present the safety and efficacy data for the NCI 7977 intermittent veliparib dosing cohort in combination with weekly irinotecan.

## Materials and Methods

### Study Design and Participants

NCI 7977 was a multicohort phase I study evaluating the safety and efficacy of veliparib and irinotecan. This manuscript describes the intermittent veliparib dosing cohort in combination with irinotecan. There was no biomarker preselection, and eligible patients had a history of metastatic or unresectable solid tumors of any histology for which standard curative or palliative measures did not exist or were no longer effective and for who irinotecan would be a viable therapy regimen. Eligible patients were required to have measurable disease according to the RECIST (version 1.1), an Eastern Cooperative Oncology Group (ECOG) performance score of 0–1, and adequate organ function as assessed by renal, hepatic, hematologic, and coagulation parameters (13). There were no restrictions on receipt of prior therapies. Patient demographics including age, sex, height, and weight were collected. The Cancer Therapy Evaluation Program at the NCI was the study sponsor, and the study was funded through the UM1 grant (1UM1CA186689). The Yale University Institutional Review Board approved the study. All enrolled patients provided written and informed consent. This cohort of NCI 7977 was only opened at the Yale Cancer Center and the study protocol is available in Supplementary Materials and Methods S1. Trial Registration: ClinicalTrials.gov identifier: (NCT0057664). The study was conducted in accordance with the Declaration of Helsinki and followed the Consolidated Standards of Reporting Trials.

### Procedures

Enrolled patients received escalating doses of oral veliparib starting at a dose of 50 mg twice daily approximately 12 hours apart on days 1–4 and 8–11 of each 21-day cycle. The veliparib dose was to be evaluated in up to six different dose levels increasing at 50 mg twice daily per dose level, starting with dose level 1 of 50 mg twice daily veliparib and dose level 6 of 300 mg twice daily veliparib (Supplementary Materials and Methods S1). The veliparib was intended to be given at higher doses with an intermittent schedule than was feasible in prolonged or continuous dosing. Irinotecan at a fixed dose of 100 mg/m^2^ was administered intravenously over 90 minutes on days 3 and 10 of each cycle. There was a lead-in period of veliparib dosing on days −14 to −11. The weekly irinotecan dose regimen was selected to increase the dose intensity of therapy over the cycle (14). Tumor biopsies were performed on days −11, 1, and 11 within 4 hours of veliparib dosing.

Molecular analysis was performed by the Oncomine Comprehensive Assay (OCA, Version 1, 2 or 3; Thermo Fisher Scientific), which interrogates a panel of cancer-related genes (143, 144, and 161 genes in OCA V1, V2, and V3, respectively) with next-generation sequencing. When available, a patient-matched germline DNA sample was sequenced in parallel with tumor DNA. One patient had Foundation One done by commercially, while all other molecular studies were performed in the Yale New Haven Hospital Tumor Profiling Laboratory.

### Outcomes

The primary endpoint was to determine the recommended phase II dose (RP2D) for irinotecan in combination with veliparib. Secondary endpoints were to determine the pharmacokinetic profile of veliparib and irinotecan and to determine the overall response rate by RECIST v1.1.

### Statistical Analysis

The study design was a standard “3+3” for dose escalation where patients were evaluated for dose-limiting toxicity (DLT) for 21 days after the initiation of cycle 1 (15). If one DLT was observed in the first 3 evaluable patients of a dose level, the cohort would be expanded to 6 evaluable patients. If ≥2 evaluable patients experience a DLT, a dose level would be determined to be the MTD and the RP2D. The DLT criteria and escalation scheme are available in the Supplementary Materials and Methods S1. The overall survival (OS) and progression-free survival (PFS) curves were generated using the Kaplan–Meier method. The median OS and PFS were reported along with their 95% confidence intervals (CI). Pending the results of dose escalation, a dose-expansion cohort was to be considered with 20 total patients with triple-negative breast cancer, which would include 10 *BRCA* mutation positive and 10 *BRCA* mutation negative.

### Data Availability Statement

The raw genomic data were generated at the Yale New Haven Hospital Tumor Profiling Laboratory. Derived data supporting the findings of this study are available from the corresponding author upon request.

## Results

Between August 1, 2016 and March 20, 2018, a total of 21 patients were screened, 6 of whom did not meet eligibility criteria (Supplementary Fig. S1). There were 15 eligible patients assigned to receive the study treatment, 6 at dose level 1 and 9 at dose level 2. The baseline demographic and disease characteristics are summarized in Table 1. There were 7 patients (47%) who received prior irinotecan and 1 patient (7%) who received a prior PARPi. Forty seven percent (7/15) of study participants had received ≥4 prior lines of systemic therapy. At dose level 1, there was one DLT of diarrhea among 6 evaluable patients. At dose level 2, the cohort was expanded to 9 patients due to multiple nonevaluable patients, and ultimately 2 of 6 evaluable patients experienced a DLT for grade 3 neutropenia. Thus, dose level 2 was determined too toxic and irinotecan 100 mg/m^2^ and veliparib 50 mg twice daily was determined to be the MTD.

**TABLE 1:** Baseline characteristics

Characteristic	Irinotecan + Veliparib (*N* = 15)
Age	
Median	63
Range	43–76
Sex – no. (%)	
Male	2 (13)
Female	13 (87)
Race – no. (%)	
White	14 (93)
Black	1 (7)
ECOG performance status – no. (%)	
0	5 (33)
1	10 (67)
Tumor type – no. (%)	
Pancreatic ductal adenocarcinoma	3 (20)
Ovarian carcinoma^a^	4 (20)
Breast cancer	3 (20)
Colon adenocarcinoma	2 (13)
Non–small cell lung cancer	1 (7)
Ampullary carcinoma	1 (7)
Biliary tract	1 (7)
Number of prior therapies – no. (%)	
1	2 (13)
2	1 (7)
3	5 (33)
≥4^b^	7 (47)
Prior PARP inhibitor – no. (%)	
Yes	1 (7)
No	14 (93)
Prior Irinotecan – no. (%)	
Yes	6 (40)
No	9 (60)

^a^Includes serous ovarian cancer and primary peritoneal carcinoma.

^b^Includes 4 patients with more than 7 lines of prior therapy.

At the time of the July 1, 2021 data cutoff for data collection, all 15 patients had discontinued study treatment and were deceased. There were no observed objective responses by RECIST version 1.1 in the study participants (Supplementary Fig. S2). As best response, 4 patients had stable disease, 8 patients had progressive disease, and 3 were unevaluable for response. One patient experienced a mixed response with −100% by RECIST in target lesions, but developed progression in nontarget lesions. This patient remained on study treatment for 9.4 months. The median PFS was 2.7 months (95% CI: 2–10.1 months) and 4 patients remained progression free for >6 months (Supplementary Fig. S3A). The median OS was 5.1 months (95% CI: 4.2–18.4 months; Supplementary Fig. S3B). The molecular results and outcomes for each patient are listed in Supplementary Table S1. For the 4 patients that experienced clinical benefit with PFS > 6 months, the tumor types were ampullary, colon, pancreatic, and ovarian and only 1 of 4 patients (ovarian) had a mutation identified in an HRD gene (*BRCA2*; Supplementary Table S1). Furthermore, for the patients with clinical benefit that previously received irinotecan, all had unequivocally progressed while receiving prior irinotecan treatment.

No patients discontinued study treatment due to treatment-related adverse events (TRAE). All study participants experienced a TRAE (Table 2) and 7 (47%) experienced a grade 3 TRAE. The majority of the grade 3 TRAE were hematologic with neutrophil count decreases in 5 (33%) participants and white blood cell count decreases in 3 (20%) which were attributed to both agents. Two patients (13%) had grade 3 diarrhea, which was attributed to irinotecan. A full list of all adverse events is provided in Supplementary Table S2. There were no treatment-related deaths.

**TABLE 2:** Treatment-related frequency of adverse events and laboratory abnormalities

Event^a^	Any grade	Grade ≥3
Any event – no. (%)	15 (100)	7 (47)^b^
Any treatment-related serious event – no. (%)	2 (13)	2 (13)
Most common events – no. (%)		
Diarrhea	7 (46)	2 (13)
Nausea	4 (27)	0
Fatigue	3 (20)	0
Flushing	3 (20)	0
Vomiting	2 (13)	0
Abdominal pain	2 (13)	0
Constipation	2 (13)	0
Alopecia	2 (13)	0
Laboratory abnormalities – no. (%)		
Neutrophil count decrease	9 (60)	5 (33)
White blood cell count decreased	9 (60)	3 (20)
Lymphocyte count decrease	3 (20)	0
Platelet count decreased	2 (13)	0
Alkaline phosphatase increased	2 (13)	0
Anemia	2 (13)	0
Hypokalemia	2 (13)	0
Hyperkalemia	2 (13)	0

^a^AE with >10% incidence.

^b^Seven patients experienced ≥ grade 3 treatment-related adverse events, diarrhea 2 (13%), neutrophil count decrease 5 (34%), white blood cell count decrease 3 (20%), and colitis 1 (7%).

## Discussion

This article presents the intermittent veliparib dosing of NCI 7977, a multicohort phase I clinical trial evaluating the safety and efficacy of the combination of veliparib and irinotecan. The safety findings revealed the MTD for the intermittent dosing cohort to be veliparib 50 mg twice daily on days 1–4 and 8–11 with irinotecan 100 mg/m^2^ on days 3 and 10 of each 21-day cycle. In the entire study population, 7 (47%) of patients experienced a grade 3 TRAE. While no confirmed objective responses were observed in this heavily pretreated patient population, 4 patients experienced >6-month PFS with a reduction in target lesions by RECIST v1.1. Among patients with prolonged stable disease, 3 of 4 patients had previously received irinotecan with unequivocal disease progression while receiving irinotecan treatment which makes sensitivity to single-agent irinotecan an unlikely explanation for the prolonged disease stability. Within this small and heavily pretreated patient population, the genomic data are of limited utility and any inferences made should be approached with caution.

There have been other recent studies that have combined veliparib with similar multiagent chemotherapy, which included irinotecan. A phase I study of veliparib in combination with 5-fluorouracil (5-FU), leucovorin, and irinotecan (FOLFIRI) revealed the RP2D to be veliparib 200 mg twice a day with standard dose FOLFIRI (irinotecan 180 mg/m^2^, leucovorin 400 mg/m^2^, 5-FU 2,400 mg/m^2^; ref. 16). The phase II study of veliparib and FOLFIRI revealed no unexpected safety concerns in 65 patients treated at a dose of veliparib 200 mg for 7 days for 14-day cycles with standard dose FOLFIRI every 2 weeks (17). Furthermore, a phase II study of veliparib and FOLFIRI as second line treatment in pancreatic cancer did not reveal any improvements in survival, and suggested increased toxicities (18). Thus, multiple studies describe similar safety profiles for veliparib with FOLFIRI compared with our experience with ≥35% of patients having a grade 3/4 TRAE, neutropenia and diarrhea being the most common. Our study differs both in the veliparib dose and schedule and in the irinotecan schedule, with weekly rather than biweekly chemotherapy. Increased toxicities may be observed with weekly irinotecan compared with other schedules (19). Moreover, the study population in NCI 7977 is more heavily pretreated than most published datasets. These differences may explain our increased toxicity rate, and inability to increase the veliparib dose safely beyond 50 mg twice daily with weekly irinotecan. This raises the possibility of selecting patients with fewer prior treatments in future studies that are examining weekly irinotecan in combination with DNA-damaging agents.

One of the major limitations of our study is the small sample size. Because toxicity concerns, and accounting for the balance of the adverse event profile and limited efficacy, the dose expansion for the higher dose intermittent veliparib dosing was not pursued. This prevents us from any clear efficacy assessments, although the 4 patients had at least minor radiographic improvements with prolonged stable disease, despite the heavily pretreated patient population and 40% of patients having received prior irinotecan. Another limitation is our use of veliparib as a PARPi, which is less potent for PARP trapping compared with other PARPis (20). Furthermore, veliparib's clinical development has largely been halted and two large phase III trials that did not reveal an OS benefit in breast cancer and non–small cell lung cancer when veliparib was added to chemotherapy (21, 22).

Our experience revealed enhanced toxicity with cytotoxic chemotherapy + PARPi combinations, and the lack of improved benefit compared with chemotherapy alone by other studies suggests more novel PARPi combinations may be preferred. For example, agents with nonoverlapping toxicity profiles such as the VEGF inhibitor bevacizumab and the PARPi olaparib. The combination of bevacizumab and olaparib is well tolerated and superior to bevacizumab alone in platinum-sensitive ovarian cancer, although it is unknown whether this is superior to olaparib monotherapy (23). We are investigating the activity of olaparib combined with ramucirumab, a mAb targeting the VEGF receptor, for metastatic gastric cancer (NCT03008278). Another combinatorial strategy may be combining PARPis with immune checkpoint inhibitors, such as niraparib and pembrolizumab, which revealed a favorable safety profile and encouraging response rates in non–small cell lung cancer (24). Moreover, the combination of durvalumab, olaparib, and paclitaxel revealed a favorable toxicity profile and pathologic complete response rate when used in the neoadjuvant setting for HER2-negative breast cancer (25). As novel DNA agents such as selective PARP1 inhibitors are developed, chemotherapy combinations may be better tolerated and thus more feasible (26).

In conclusion, the MTD of intermittent veliparib is 50 mg twice daily on days 1–4 and 8–11 with weekly irinotecan 100 mg/m^2^ days 3 and 10 every 14 days. The most common TRAE are diarrhea and hematologic toxicities. While no objective responses were seen, several patients without any evidence of HRD still demonstrated prolonged stable disease even those with prior irinotecan treatment. Because of the side-effect profile with higher dose veliparib on an intermittent schedule with weekly irinotecan as well as the limited efficacy, this arm of NCI7977 was closed prematurely without pursuing dose expansion. However, the other study arms that incorporated lower dose 14 days continuous veliparib with irinotecan were continued as planned. Thus, the toxicity profile and limited efficacy will likely limit further investigation of PARPis with irinotecan for this schedule.

## Supplementary Material

Supplementary Figure 1 Trial profile for enrolled patients.

Supplementary Figure 2 Waterfall plot for best response by RECIST version 1.1.

Supplementary Figure 3 Progression free survival and overall survival curves.

Supplementary Table 1 Molecular results and outcomes for each patient.

Supplementary Table 2 A full list of all adverse events.

Supplementary Methods 1 The clinical trial protocol for NCI 7977.
