# Supplementary figures and images for: NCI 7977: A Phase I Dose-Escalation Study of Intermittent Oral ABT-888 (Veliparib) plus Intravenous Irinotecan Administered in Patients with Advanced Solid Tumors
Source: Cancer Res Commun. 2023 Jun 26;3(6):1113–7. doi: 10.1158/2767-9764.CRC-22-0485 (PMC10292219; doi:10.1158/2767-9764.CRC-22-0485)

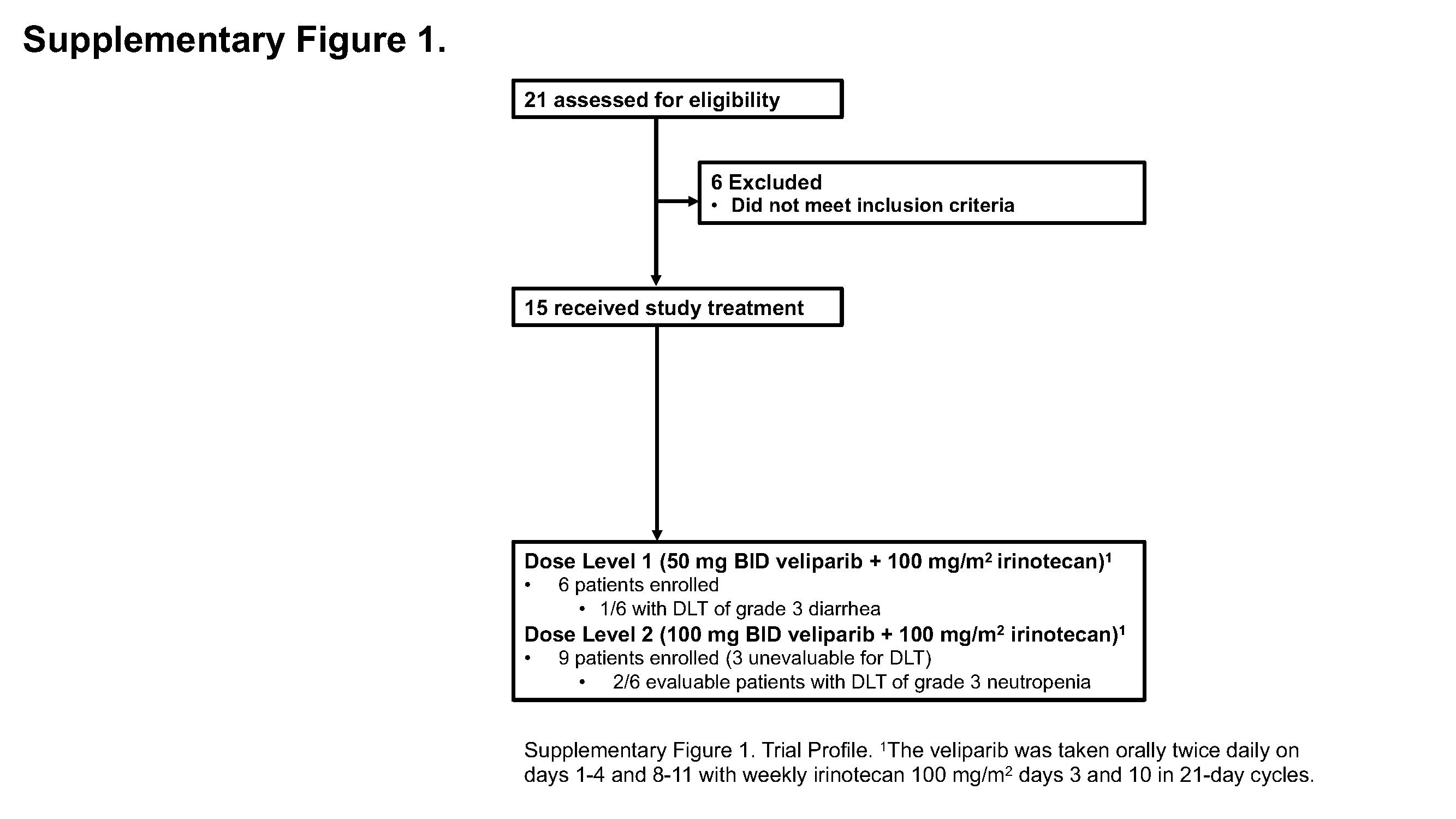

Supplement: Supplementary Figure 1 — Trial profile for enrolled patients. [file crc-22-0485-s01.png]

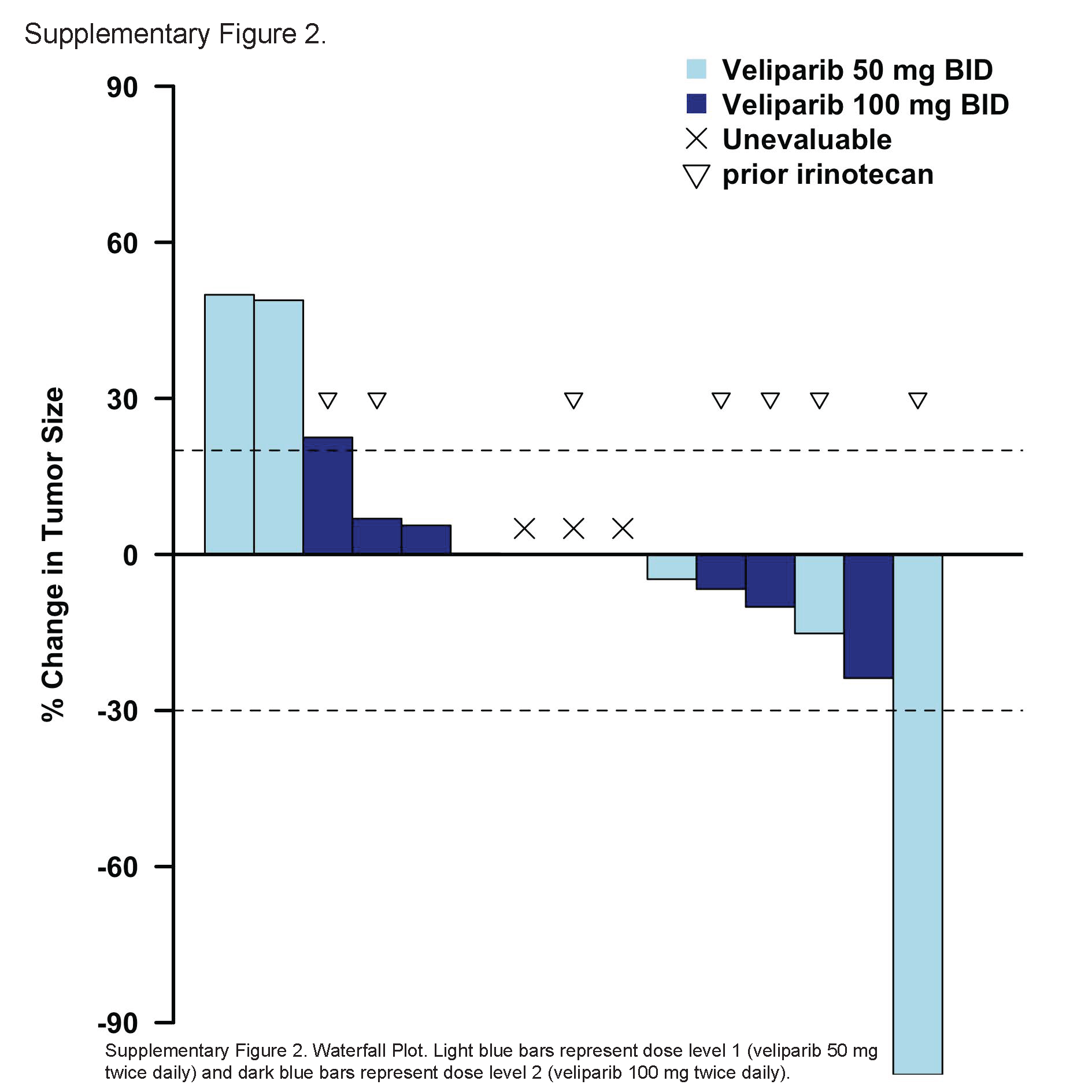

Supplement: Supplementary Figure 2 — Waterfall plot for best response by RECIST version 1.1. [file crc-22-0485-s02.png]

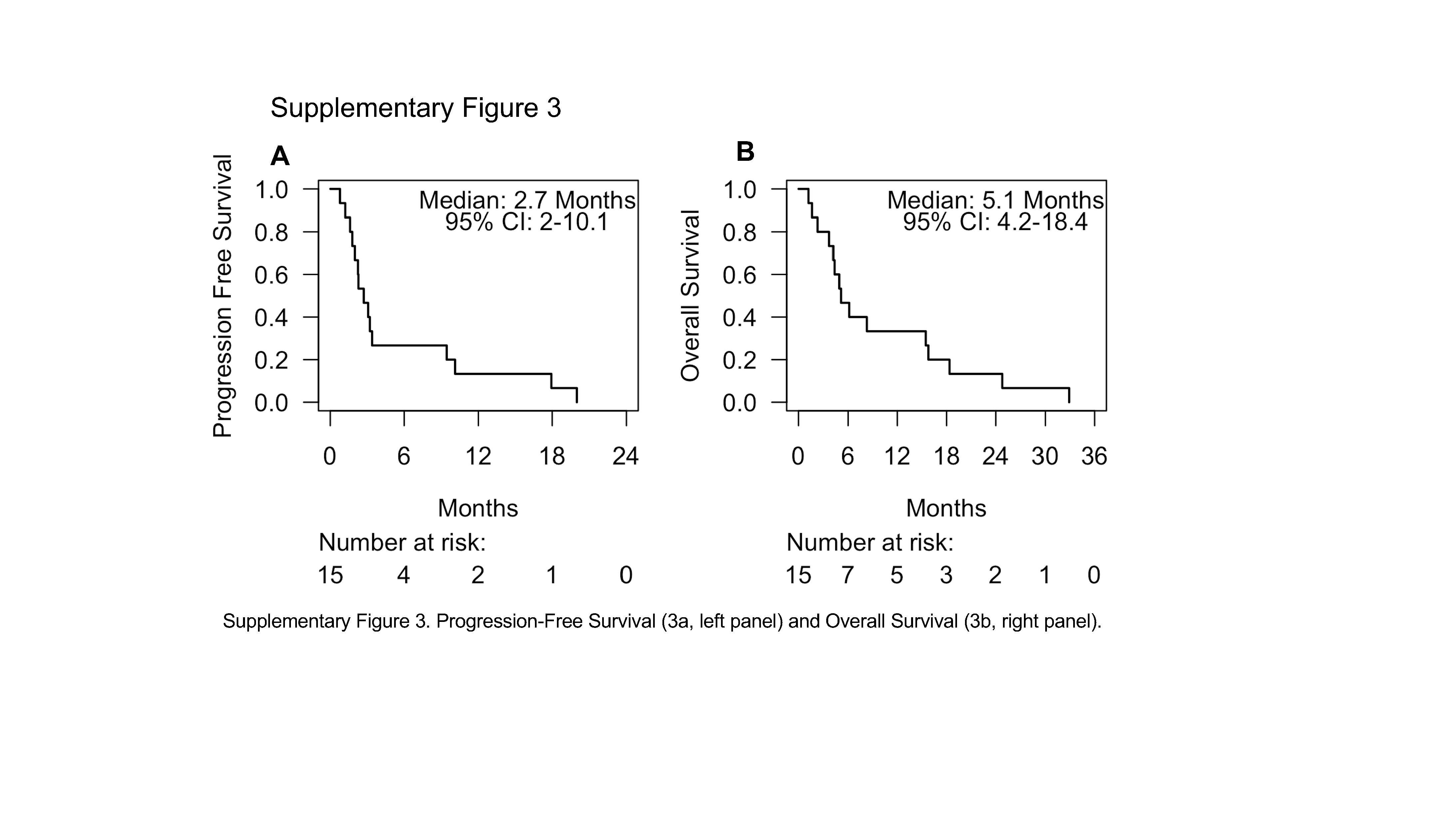

Supplement: Supplementary Figure 3 — Progression free survival and overall survival curves. [file crc-22-0485-s03.png]

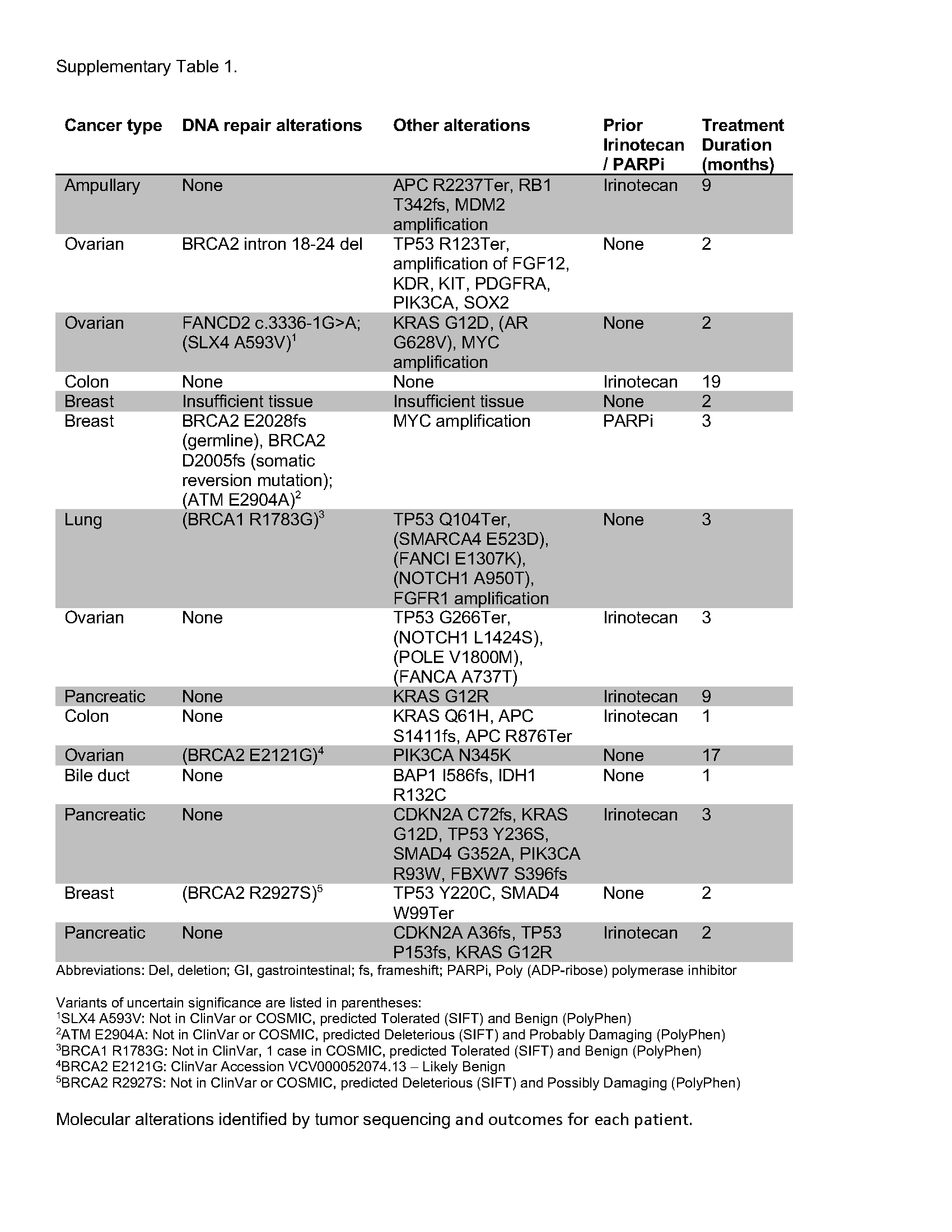

Supplement: Supplementary Table 1 — Molecular results and outcomes for each patient. [file crc-22-0485-s04.png]

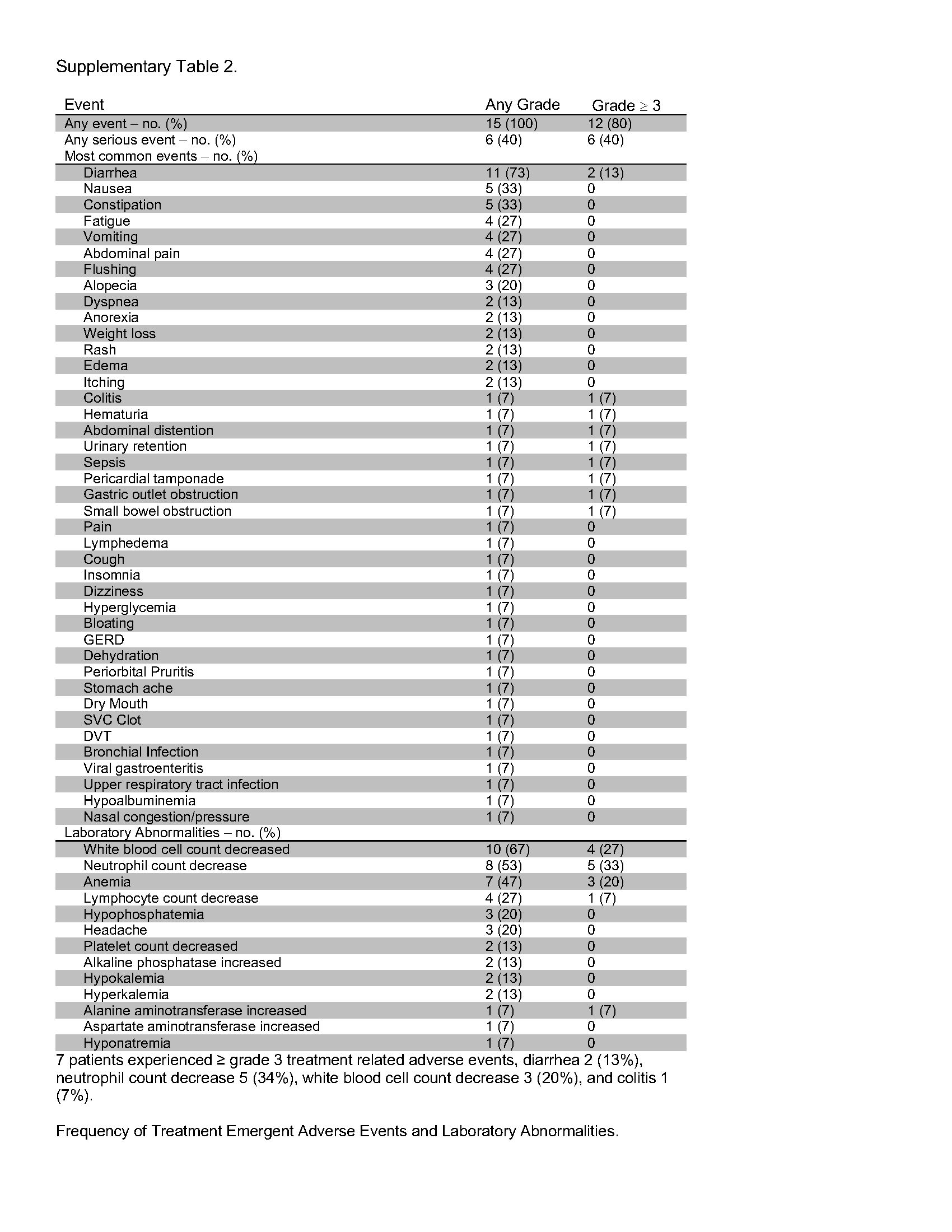

Supplement: Supplementary Table 2 — A full list of all adverse events. [file crc-22-0485-s05.png]
