# Supplementary material for: NCI 7977: A Phase I Dose-Escalation Study of Intermittent Oral ABT-888 (Veliparib) plus Intravenous Irinotecan Administered in Patients with Advanced Solid Tumors
Source: Cancer Res Commun. 2023 Jun 26;3(6):1113–7. doi: 10.1158/2767-9764.CRC-22-0485 (PMC10292219; doi:10.1158/2767-9764.CRC-22-0485)
Supplement: Supplementary Methods 1 — The clinical trial protocol for NCI 7977. [file crc-22-0485-s06.pdf]

Supplementary Methods 1

SUMMARY OF CHANGES - Protocol

For Protocol Amendment # 30 to: A Phase I Dose-Escalation Study of Oral ABT-888 (NSC #737664) Plus Intravenous Irinotecan (CPT-11, NSC#616348) Administered in Patients with Advanced Solid Tumors

NCI Protocol #: 7977
Local Protocol #: 1410014852

Protocol Date: 06/06/2018

I. CTEP Request for Rapid Amendment (RRA) dated May 24, 2018:

#	Section	Comments
1.	5.3.1.1	Removed "myelosuppression" from section
2.	5.3.1.3	Added new sub-section with RRA text about monitoring for persistent myelosuppression.
3.	5.4	As this is a Phase 1 combination study, patients will not be able to continue on irinotecan monotherapy if the events below occur. Added the following bullet points to Duration of Therapy section (criteria for removal from treatment).<br><br>• Bone marrow findings consistent with acute myeloid leukemia (AML)/MDS<br><br>• Severe persistent anemia requiring transfusion to maintain ≥10 g/dL hemoglobin (Hb)
4.	6.0	Added modified RRA ABT-888 Hematologic Toxicity Management guidelines.
5.	7.1.1	Updated ABT-888 CAEPR to Version 2.4, May 13, 2018 as per RRA instructions.

II. CTEP Review Letter Dated March 22, 2018 – Recommendations:

#	Section	Comments
1.	**4.2.3**	**Please update the CTSU Regulatory Office suite number to 3000.**<br><br>CTSU Regulatory Office<br>1818 Market Street, Suite 3000 ~~1100~~<br>Philadelphia, PA 19103<br><br>**PI Response: The suite number has been updated as recommended.**

III. Additional Changes by the Principal Investigator:

#	Section	Comments
1.	All	Updated Version Date in Footer
2.	Face Page	Updated Version Date and Amendment #
3.	6.0	Revised reference to Table 5 in bullet points 2 & 4 as new information on toxicity management inserted per RRA
4.	New Table 6<br>New Table 6.1<br>New Table 7<br>New Table 8<br>New Table 9	Renumbered Tables as new Tables inserted for hematologic toxicity management per RRA
5.	Appendix H	Updated Version Date in Patient Pill Diaries (Dose Escalation for Intermittent ABT-888 Portion)

NCI Protocol #: 7977
Local Protocol #: 1410014852
ClinicalTrials.gov Identifier: NCT00576654
Investigational New Drug (IND) #: 77840

A Phase I Dose-Escalation Study of Oral ABT-888 (NSC #737664) Plus Intravenous Irinotecan (CPT-11, NSC#616348) Administered in Patients with Advanced Solid Tumors

Corresponding Organization: **LAO-CT018** / Yale University Cancer Center LAO
Participating Institutions:

**LAO-11030** / University Health Network Princess Margaret Cancer Center LAO
**LAO-CA043** / City of Hope Comprehensive Cancer Center LAO
**LAO-CT018** / Yale University Cancer Center LAO
**LAO-MA036** / Dana-Farber - Harvard Cancer Center LAO
**LAO-MD017** / JHU Sidney Kimmel Comprehensive Cancer Center LAO
**LAO-MN026** / Mayo Clinic Cancer Center LAO
**LAO-NC010** / Duke University - Duke Cancer Institute LAO
**LAO-NJ066** / Rutgers University - Cancer Institute of New Jersey LAO
**LAO-OH007** / Ohio State University Comprehensive Cancer Center LAO
**LAO-PA015** / University of Pittsburgh Cancer Institute LAO
**LAO-TX035** / University of Texas MD Anderson Cancer Center LAO
**LAO-NCI** / National Cancer Institute LAO

Correlative Funding Source: AbbVie, Inc.

Principal Investigator: [redacted], D.O.
[redacted] Innovative Medicine
Yale Cancer Center
333 Cedar Street, WWW217
P.O. Box 208028
New Haven, CT 06520-8028
Phone: [redacted]
Fax: [redacted]
E-mail: [redacted]

Correlative Science Co-Investigators (Yale University)
NOTE: Dr. [redacted] is not participating in patient care for this study)
[redacted] Ph.D.
Departments of Therapeutic Radiology and Human Genetics
Yale University School of Medicine
Hunter Building, Room [redacted]
15 York Street
New Haven, CT 06520
Phone: [redacted]

Fax: [redacted]
E-mail: [redacted]

[redacted] M.D. Ph.D.
NOTE: Dr. [redacted] is not participating in patient care for this study)
Dept of Pathology, Rm [redacted]
Yale University School of Medicine
310 Cedar St. PO Box 208023
New Haven, CT 06520-8023
Phone: [redacted]
E-mail: [redacted]

[redacted], M.D., Ph.D.
NOTE: Dr. [redacted] is not participating in patient care for this study
310 Cedar St. BML113, Department of Pathology
Yale School of Medicine
New Haven, CT 06520-8023
Phone: [redacted]
Email: [redacted]

Biostatistician:
[redacted] Ph.D.
Karmanos Cancer Institute
87 East Canfield, Mailcode, MM03BI
Detroit, MI 48201
Phone: [redacted]
Email: [redacted]

Lead Clinical Project Manager:
[redacted] Ph. D, MS, PA-C, CCRP
[redacted]
259 Mack Avenue
Suite [redacted]
Detroit, MI 48201
Office: [redacted]
Fax: [redacted]
Email: [redacted]

Lead Site Responsible Data Manager:
[redacted]
Yale University/Yale Cancer Center
300 George St. Suite [redacted]
New Haven, CT 06511
phone: [redacted]
fax [redacted]
Email: [redacted]

NCI Supplied Agent: ABT-888 (NSC #737664)
Commercially available agent: Irinotecan (CPT-11, NSC#616348)
Version Date: 02/07/07 (Original)

04/27/07 (Resubmission to CTEP)
07/25/07 (Resubmission in response to FDA review)
10/16/07 (Resubmission in response to IRB review)
07/21/08 (Amendment 1)
03/10/09 (Amendment 2)
08/13/09 (Amendment 3)
09/30/09 (Amendment 4)
03/02/10 (Amendment 5)
06/10/10 (Amendment 6)
07/19/10 (Amendment 7)
10/01/10 (Amendment 7 revised)
01/12/11 (Amendment 8)
08/03/11 (Amendment 9)
12/02/11 (Amendment 10)
03/12/12 (Amendment 11)
03/21/12 (Amendment 12)
04/03/12 (Amendment 13)
05/25/12 (Amendment 14)
08/14/12 (Amendment 15)
10/30/12 (Amendment 16)
06/07/13 (Amendment 17)
08/07/13 (Amendment 18)
03/14/14 (Amendment 19)
08/07/14 (Amendment 20)
10/14/14 (Amendment 21)
12/08/14 (Amendment 22)
01/06/15 (Amendment 22 revised)
02/25/15 (Amendment 23)
11/13/15 (Amendment 24)
03/18/16 (Amendment 25)
06/03/16 (Amendment 26)
06/22/16 (Amendment 26 - revised)
01/13/17 (Amendment 27)
08/10/17 (Amendment 28)
01/08/18 (Amendment 28 revised – response to CIRB)
03/08/18 (Amendment 29)
06/06/18 (Amendment 30)

SCHEMA: Dose Escalation Portion

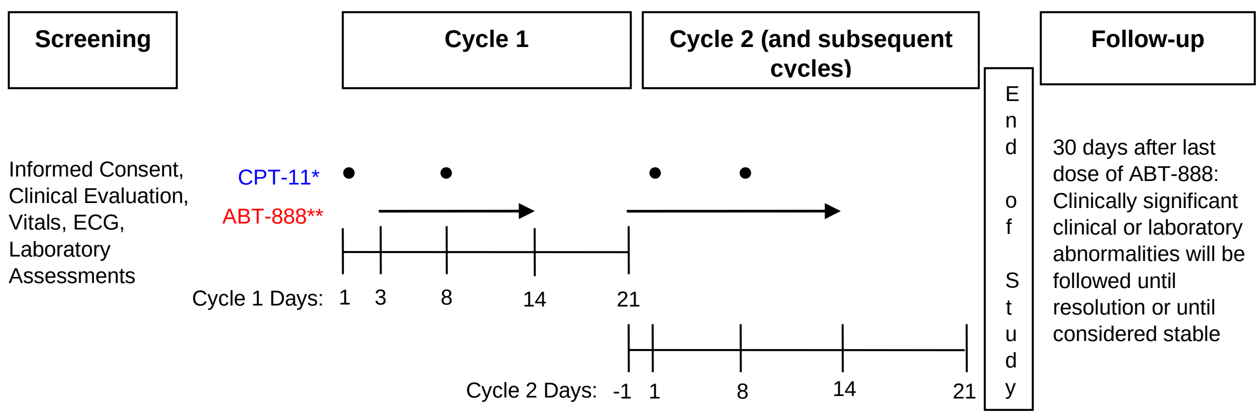

* CPT-11 administered on days 1 and 8 of each cycle
** **Cycle I:** ABT-888 administered Day 3 through Day 14
Cycle II (and subsequent cycles): ABT-888 administered from Day -1 through Day 14 (half dose on Day -1 of Cycle 2 only)

Cycle 1: Irinotecan (CPT-11) will be administered by intravenous infusion at the indicated dose over 90 minutes on both Day 1 and Day 8 of Cycle 1. Single–dose PK sampling of irinotecan will be obtained up to 48h starting on Day 1. Twice daily oral administration of ABT-888 will begin two days following infusion of irinotecan therapy (starting Day 3), and will continue twice daily for a total of 12 days (through Day 14) followed by a 6 day rest. Tumor collection for PAR level evaluation and PD assays will occur at two times: 28 hours after chemotherapy alone (Day 2) and 28 hours after the combination of ABT-888 and chemotherapy (Day 9).

Cycle 2: ABT-888 will be administered one day prior (Day -1) to irinotecan therapy (half of normal total daily dose of ABT-888 to allow for PK sampling). A single–dose PK sampling of ABT-888 will be obtained up to 28 h starting on Day -1 of cycle 2. Subsequent doses of ABT-888 will be given twice-daily and continue daily through Day 14 of Cycle 2, followed by a 6 day rest. Irinotecan will be administered by intravenous infusion at the indicated dose over 90 minutes on days 1 and 8 of Cycle 2. The first dose of ABT-888 to be administered on Day 8 of Cycle 2 will be taken at the time of the start of irinotecan infusion, so PK sampling up to 48 h can be lined up for both drugs.

Subsequent cycles: Irinotecan will be administered by intravenous infusion at the indicated dose over 90 minutes on days 1 and 8. ABT-888 will be administered orally twice daily from Day -1 through Day 14, followed by a 6 day rest.

Dose Escalation Schedule

Dose Level	Dose*	
	ABT-888 (mg po BID)	Irinotecan (CPT-11) (mg/m^2 IV)
Level 1	10	100
Level 2	20	100
Level 3	40	100
Level 4	50	100
Level 5	80	100
Level 6	120	100

SCHEMA: Expansion Portion

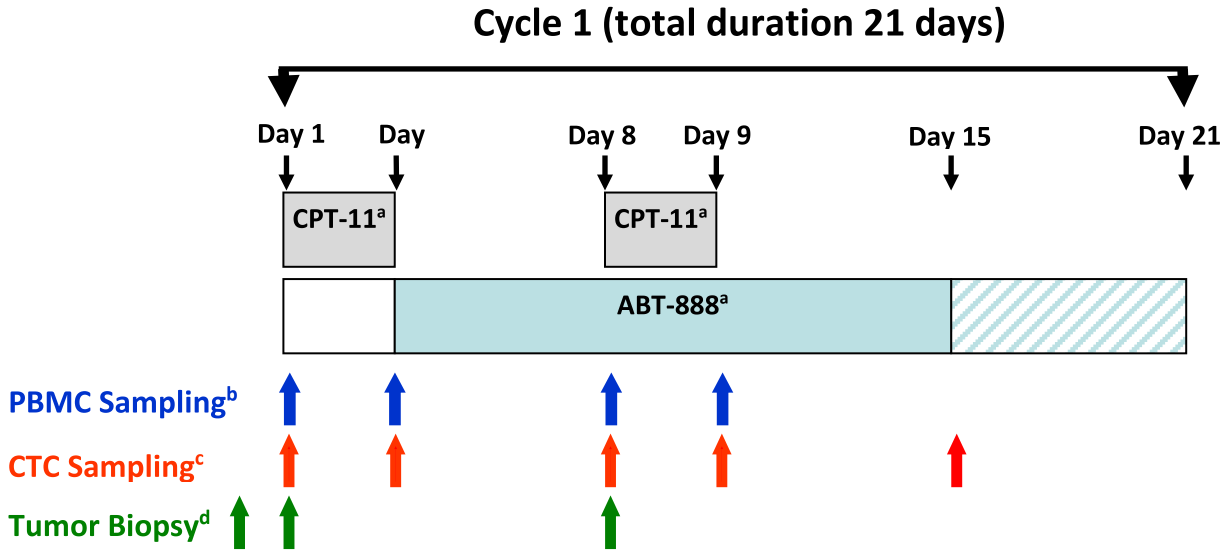

[a] CPT-11 (irinotecan) 100 mg/m^2 IV administered for 90 min on days 1 and 8 of each cycle. ABT-888 40 mg po bid on days 2-15 in cycle 1; Decision to administrate ABT-888 on a continuous schedule predicated on results from first 6 patients

[b] PBMC samples will be obtained at baseline, 4-6, 8, 22, and 24 h after CPT-11 alone on day 1 and after the combination dose on day 8 in cycle 1

[c] Circulating tumor cell (CTC) samples will be obtained at baseline, 4-6, 8, 22, and 24 h after CPT-11 alone on day 1 and after the combination dose on day 8 and once on day 15 of cycle 1

[d] Tumor biopsy will be performed at baseline, 4-6 hours after the first dose of CPT-11 (day 1) and the combination (day 8) in cycle 1.

Cycle 1: Irinotecan (CPT-11) will be administered by intravenous infusion at 100 mg/m^2 over 90 minutes on both Day 1 and Day 8 of Cycle 1. Twice daily oral administration of ABT-888 (40 mg po bid, 80 mg total daily dose) will begin 24 hours following infusion of irinotecan therapy (starting Day 2), and will continue twice daily for a total of 14 days (through Day 15) followed by a 7 day rest. Tumor collection for PD assays will occur at three times: baseline (within 2 weeks of treatment), 4-6 hours after the first dose of irinotecan (Day 1) and 4-6 hours after the combination of ABT-888 and irinotecan on Day 8.

Cycle 2 and Subsequent cycles: Irinotecan will be administered by intravenous infusion at 100 mg/m^2 over 90 minutes on days 1 and 8. ABT-888 will be administered orally twice daily (40 mg po bid, 80 mg total daily dose) from Day 1 through Day 15, followed by a 6 day rest. Decision to administrate ABT-888 on a continuous schedule will be predicated on results from first 6 patients following discussion with CTEP.

SCHEMA: Dose Escalation for Intermittent ABT-888

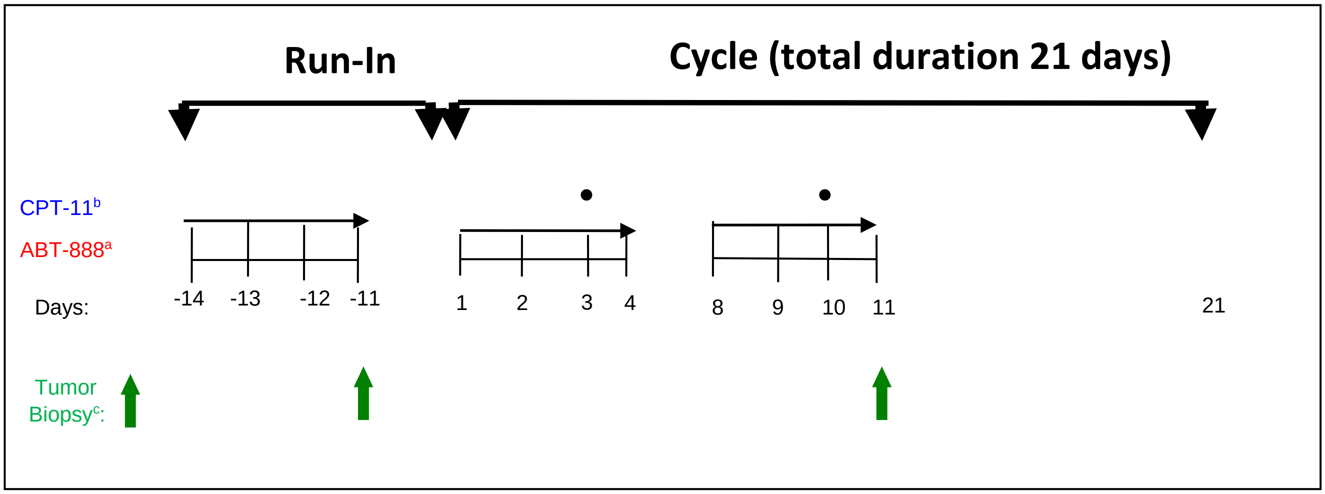

a ABT-888 po bid days -14 to -12 and once on day -11 (morning dose only) of run-in, then ABT-888 bid on days 1 to day 4 and days 8 to 11 of each 21 day cycle

b CPT-11 (irinotecan) IV administered for 90 min on days 3 and 10 of each cycle.

c Tumor biopsy will be performed at baseline, within 4 hours after morning ABT-888 dose on day -11 and within 4 hours of ABT-888 dose on cycle 1 day 11

Run-In: ABT-888 will be administered orally twice daily on days -14, -13 and -12. ABT-888 will be administered orally once on day -11. Tumor collection will occur on day -11 within 4 hours of morning dose of ABT-888.

Cycle 1: Irinotecan will be administered by intravenous infusion at 100 mg/m^2 over 90 minutes on days 3 and 10. ABT-888 will be administered orally twice daily from Day 1 through Day 4 and Days 8-11. Tumor collection will occur within 4 hours of ABT-888 dose on Cycle 1 Day 11.

Cycle 2 and Subsequent cycles: Irinotecan will be administered by intravenous infusion at 100 mg/m^2 over 90 minutes on days 3 and 10. ABT-888 will be administered orally twice daily from Day 1 through Day 4 and Days 8-11.

Intermittent ABT-888 Dose Escalation Schedule		
**Dose Level**	**Dose***	
	**<u>Run-In:</u> ABT-888 (mg po BID) Days -14, -13, -12 ABT-888 (mg po once) Day -11**	**<u>Run-In:</u> Irinotecan (CPT-11) None**
	**<u>Cycles:</u> ABT-888 (mg po BID) Days 1, 2, 3, & 4 Days 8, 9, 10 & 11**	**<u>Cycles:</u> Irinotecan (CPT-11) (mg/m^2 IV) Days 3 & 10**
Level 1	50	100

Level 2	100	100
Level 3	150	100
Level 4	200	100
Level 5	250	100
Level 6	300	100
Intermediate dosing may be permitted if needed and only after discussion with CTEP		
**Doses are stated as exact dose in units (e.g., mg/m^2) rather than as a percentage. Note that ABT-888 doses are "flat" and* ***not*** *calculated based on weight or BSA.*		

TABLE OF CONTENTS

SUMMARY OF CHANGES i
SCHEMA: Dose Escalation Portion 4
SCHEMA: Expansion Portion 5
SCHEMA: Dose Escalation for Intermittent ABT-888 6
TABLE OF CONTENTS 8
LIST OF ABBREVIATIONS 10
1. OBJECTIVES 12
1.1 Primary Objectives (ORIGINAL DOSE ESCALATION PORTION) 12
1.2 Primary Objectives (DOSE ESCALATION FOR INTERMITTENT ABT-888 PORTION) 12
1.3 Secondary Objectives 12
1.4 Secondary Objectives (DOSE ESCALATION FOR INTERMITTENT ABT-888 PORTION) 12
1.5 Exploratory Objectives (ORIGINAL DOSE ESCALATION PORTION): 12
1.6 Exploratory Objectives (EXPANSION PORTION): 13
1.7 Exploratory Objectives (DOSE ESCALATION FOR INTERMITTENT ABT-888 PORTION) 13
2. BACKGROUND 14
2.1 CTEP-Supplied Investigational Agent (ABT-888) 14
2.2 Irinotecan (CPT-11, NSC#616348) 21
2.3 Rationale 22
2.4 Correlative Studies Background 23
3 PATIENT SELECTION 31
3.1 Eligibility Criteria 31
3.2 Exclusion Criteria 32
3.3 Inclusion of Women and Minorities 34
4. REGISTRATION PROCEDURES 34
4.1 Investigator and Research Associate Registration with CTEP 34
4.2 Site Registration 35
4.3 Patient Registration 36
4.4 General Guidelines 38
5. TREATMENT PLAN 38
5.1 Agent Administration 38
5.1.1 ABT-888 42
5.1.2 Irinotecan (CPT-11) 44
5.2 Definition of Dose-Limiting Toxicity and Recommended Phase II Dose 44
5.3 General Concomitant Medication and Supportive Care Guidelines 47
5.3.1 ABT-888 47
5.3.2 Irinotecan 47
5.4 Duration of Therapy 49
5.5 Duration of Follow Up 50
5.6 Criteria for Removal from Study 50
6. DOSING DELAYS/DOSE MODIFICATIONS 50
7. ADVERSE EVENTS: LIST AND REPORTING REQUIREMENTS 54
7.1 Comprehensive Adverse Events and Potential Risks Lists (CAEPRs) 54
7.2 Adverse Event Characteristics 58
7.3 Expedited Adverse Event Reporting 58
7.4 Routine Adverse Event Reporting 60
7.5 Secondary AML/MDS 60
8. PHARMACEUTICAL INFORMATION 61
8.1 CTEP IND Agent ABT-888 (NSC # 737664) 61
8.2 Other Investigational Agent(s): N/A 63

8.3 Irinotecan hydrochloride injection (CPT-11, NSC#616348) ... 63
9. CORRELATIVE/SPECIAL STUDIES 64
9.1 Laboratory Correlative Studies (DOSE ESCALATION PORTION ONLY – THIS SECTION IS NOT USED IN THE EXPANSION PORTION. SEE SECTION 9.2 FOR LABOARATORY CORRELATES RELATED TO THE EXPANSION PORTION AND SECTION 9.3 FOR LABORATORY CORRELATES RELATED TO THE DOSE ESCALATION FOR INTERMITTENT ABT-888 PORTION) ... 64
9.2 Laboratory Correlative Studies (EXPANSION PORTION) ... 73
9.3 Laboratory Correlative Studies (DOSE ESCALATION FOR INTERMITTENT ABT-888 PORTION) ... 87
10. STUDY CALENDAR 95
10.1 Weekly Calendar (Original Dose Escalation Portion) ... 95
10.2 Daily Calendar (Cycle 1) Original Dose Escalation Portion ... 97
10.3 Daily Calendar (Subsequent Cycles) – Original Dose Escalation Portion ... 99
10.4 Weekly Calendar (Expansion Portion) ... 100
10.5 Daily Calendar (Cycle 1) Expansion Portion ... 102
10.6 Daily Calendar (Subsequent Cycles) – Expansion Portion ... 104
10.7 Weekly Calendar (Dose Escalation for Intermittent ABT-888) ... 105
10.8 Daily Calendar (Run-In and Cycle 1) Dose Escalation for Intermittent ABT-888 ... 106
10.9 Daily Calendar (Subsequent Cycles) –Dose Escalation for Intermittent ABT-888 ... 107
11 MEASUREMENT OF EFFECT 108
11.1 Antitumor Effect – Solid Tumors ... 108
12. STUDY OVERSIGHT AND DATA REPORTING / REGULATORY REQUIREMENTS 114
12.1 Study Oversight ... 115
12.2 Data Reporting ... 115
12.3 Cooperative Research and Development Agreement (CRADA)/Clinical Trials Agreement (CTA) ... 117
13. STATISTICAL CONSIDERATIONS 119
13.1 Original Dose Escalation Portion ... 119
13.2 RP2D Cohort Expansion Portion ... 120
13.3 Dose Escalation for Intermittent ABT-888 Portion ... 122
14. REFERENCES 125
APPENDIX A: Performance Status Criteria 130
APPENDIX B: CTEP MULTICENTER GUIDELINES 131
APPENDIX C: Pharmacokinetic, Pharmacodynamic, and Pharmacogenomic sample collection 132
APPENDIX D: Standard Operating Procedure (SOP) for Collection and Preparation of PBMC samples 135
APPENDIX E: Standard Operating Procedure (SOP) for Collection and Preparation of Tumor Biopsy samples for PAR Immunoassay and DNA-damage Response PD Assays 141
APPENDIX F: Processing of 2nd Biopsy (Dose Escalation Portion ONLY) 146
APPENDIX G: Pharmacokinetic Worksheet 149
APPENDIX H: Patient Pill Diaries 154
APPENDIX I: ABT-888 Administration/Dispensing Schedule 164
APPENDIX J: Standard Operating Procedure (SOP) for Collection and Preparation of Circulating Tumor Cells (CTCs) for γH2AX-Assays 166
Appendix K: Research Blood Shipping Manifest 172
Appendix L: Frozen Tissue Shipping Manifest 173
Appendix M: Formalin-Fixed, Paraffin-Embedded Tissue Shipping Manifest 174
APPENDIX N: PATIENT DRUG INFORMATION HANDOUT AND WALLET CARD 175

LIST OF ABBREVIATIONS

Abbreviation	Full text
CTEP-AERS	CTEP Adverse Event Reporting System
AE	adverse event
ALT	alanine transaminase
ANC	absolute neutrophil count
AST	aspartate aminotransferase
AUC	area under the curve
B-HCG	beta human chorionic gonadotropin
BID	twice daily
BRCA	breast cancer gene
C	Celsius
CBC	complete blood count
CFR	Code of Federal Regulations
CL	clearance
CL/F	oral clearance
C_{max}	maximum observed plasma concentration
CNS	central nervous system
CR	complete response
CT	computed tomography
CTC	Circulating tumor cell
CTCAE	Common Terminology Criteria for Adverse Events
CTEP	Cancer Therapy Evaluation Program
CTO	Clinical Trials Office
d	day
DCTD	Division of Cancer Treatment and Diagnosis
DFCI	Dana-Farber Cancer Institute
DLT	dose limiting toxicity
DMC	data monitoring committee
DNA	deoxyribonucleic acid
ECG	electrocardiogram
ECOG	Eastern Cooperative Oncology Group
EKG	electrocardiogram
F	fraction of the dose systemically available after oral administration
FDA	Food and Drug Administration
FDF	Financial Disclosure Form
Hgb	hemoglobin
HIC	Human Investigation Committee
HPLC	high performance liquid chromatography
HWCRC	Hudson-Webber Cancer Research Center
IDF	Investigator Data Form
IP	intraperitoneally
IRB	Institutional Review Board
KCI	Karmanos Cancer Institute
kg	kilogram
LHTP	Laboratory of Human Toxicology & Pharmacology
MAD	maximum administered dose
mcg	microgram

MTD	maximum tolerated dose
mg	milligram
mL	milliliter
MS	mass spectrometry
NCI	National Cancer Institute
NCTVL	National Clinical Target Validation Laboratory
NOEL	no-observed-effect level
OBD	optimal biologic dose
PADIS	Pharmacodynamic Assay Development and Implementation Section
PAR	Poly(ADP-ribose)
PARG	poly(ADP-ribose) glycohydrolase
PARP	poly(ADP-ribose) polymerase
PBMC	peripheral blood mononuclear cells
PCR	polymerase chain reaction
PD	progressive disease
PD	pharmacodynamics
PET	positron emission tomography
PI	principal investigator
PK	pharmacokinetics
PMB	Pharmaceutical Management Branch
PO	orally
PR	partial response
PRMC	Protocol Review and Monitoring Committee
PLT	platelet
PTT	partial thromboplastin time
RECIST	Response Evaluation Criteria in Solid Tumors
RNA	ribonucleic acid
RP2D	recommended phase II dose
SAE	serious adverse event
SCID	severe combined immunodeficient
SD	stable disease
SGOT	serum glutamic-oxaloacetic transaminase
SGPT	serum glutamate pyruvate transaminase
$t_{1/2}$	terminal half-life
TGen	Translational Genomics Research Institute
TRL	Translational Research Laboratory
TK	thymidine kinase
T_{max}	time of maximum observed concentration
TS	thymidylate synthase
UMGCC	University of Maryland Greenebaum Cancer Center
ULN	upper limit of normal
US	Ultrasound
V_{ss}	volume of distribution at steady-state
wk	Week
WSU	Wayne State University

1. OBJECTIVES

1.1 Primary Objectives (ORIGINAL DOSE ESCALATION PORTION)

1.1.1 To determine the optimal biologic dose (OBD) for poly(ADP-ribose) polymerase (PARP) inhibition using irinotecan (once weekly intravenously in 2 of 3 weeks), in combination with ABT-888 (twice daily orally for 2 of 3 weeks).

1.1.2 To determine the recommended phase II dose (RP2D) for irinotecan (once weekly intravenously in 2 of 3 weeks), in combination with ABT-888 (twice daily orally for 2 of 3 weeks), determined by evaluating the feasibility, safety, dose limiting toxicities and the maximally tolerated dose.

1.1.3 To determine the safety profile of irinotecan in combination with ABT-888: The incidence of adverse events (AEs) and clinically significant changes in laboratory tests, electrocardiograms (ECGs), and vital signs.

1.1.4 To determine the safety profile of irinotecan in combination with ABT-888 at the recommended phase II dose: The incidence of adverse events (AEs) and clinically significant changes in laboratory tests, ECGs, and vital signs.

1.2 Primary Objectives (DOSE ESCALATION FOR INTERMITTENT ABT-888 PORTION)

1.2.1 To determine the recommended phase II dose (RP2D) for irinotecan (once weekly intravenously in 2 of 3 weeks), in combination with ABT-888 (twice daily orally for intermittent dosing days 1 to 4 and days 8 to 11 of each cycle), determined by evaluating the feasibility, safety, dose limiting toxicities and the maximally tolerated dose (MTD).

1.2.2 To determine the safety profile of irinotecan in combination with ABT-888: The incidence of adverse events (AEs) and clinically significant changes in laboratory tests, and vital signs.

1.2.3 To determine the safety profile of irinotecan in combination with ABT-888 at the recommended phase II dose: The incidence of adverse events (AEs) and clinically significant changes in laboratory tests, and vital signs.

1.3 Secondary Objectives

1.3.1 To determine the pharmacokinetic (PK) profile of ABT-888.

1.3.2 To determine the PK profile of irinotecan (CPT-11) both as a single agent and in combination with ABT-888.

1.3.3 To determine the tumor response as assessed by the Response Evaluation Criteria in Solid Tumors (RECIST).

1.4 Secondary Objectives (DOSE ESCALATION FOR INTERMITTENT ABT-888 PORTION)

1.4.1 To determine the tumor response as assessed by the Response Evaluation Criteria in Solid Tumors (RECIST).

1.4.2 To describe response rate (RR) in patients

1.5 Exploratory Objectives (ORIGINAL DOSE ESCALATION PORTION):

1.5.1 Pharmacodynamic (PD) biomarker response: PARP inhibition in peripheral blood mononuclear cells (PBMC) by measurement of PAR levels.

1.5.2 DNA damaging effects of irinotecan and the combination of irinotecan with ABT-888: levels of γ-H2AX and Rad51 formation in tumor tissue.

1.5.3 Relevance of CYP2C9 and 2C19 polymorphisms, UGT1A1 polymorphism, and

ABCG2 polymorphism to the pharmacokinetics of irinotecan and/or ABT-888.

1.6 Exploratory Objectives (EXPANSION PORTION):

1.6.1 To explore whether a positive γ-H2AX response in tumor tissue at 4-6 hrs is reflected in CTCs between 8-24 hrs but not at 4-6 hrs, as predicted.

1.6.2 To explore whether PARP inhibition increases γH2AX response of CTCs to plasma drug by 4-6 hrs after CPT-11 administration.

1.6.3 To explore whether PARP inhibition increases γ-H2AX response of tumor cells to tissue drug level, as indicated by CTCs at 8-24 hrs after CPT-11.

1.6.4 To explore when the γ-H2AX response peak in CTCs occurs, indicating a response in tumor.

1.6.5 To explore whether there is a tumor switch between γ-H2AX and ERCC1-mediated repair in the presence of PARP inhibition, (i.e., repeat initial PBMC and tumor findings).

1.6.6 To perform analysis of CTCs at day 15 to help guide alteration in ABT-888 drug administration schedule (continuous administration).

1.6.7 To sequence the genome and transcriptome from both normal and tumor tissue from each study patient in the expansion cohort to evaluate point mutations, structural changes and copy number events.

1.6.8 To evaluate the damaging effects of irinotecan and the combination of irinotecan with ABT-888 by examining levels of Rad51 formation in tumors.

1.6.9 To evaluate the percentage of BCSC in serial breast tumor biopsies before and after irinotecan alone and after 1 cycle of treatment with the combination of irinotecan and ABT-888.

1.6.10 To perform molecular profiling of the tumor cell and BCSC populations before and after irinotecan alone and after 1 cycle of treatment with the combination of irinotecan and ABT-888.

1.6.11 To compare Rad51 foci in ALDH+ stem cell populations to the bulk tumor cells.

1.7 Exploratory Objectives (DOSE ESCALATION FOR INTERMITTENT ABT-888 PORTION)

1.7.1 To develop assays to detect trapping of PARP1 and 2 in tumor biopsy tissue in response to treatment with Irinotecan plus a PARP inhibitor, in this case ABT-888. We will work on the development of three assays and these include the subcellular fractionation assay, the FANCD2 monoubiquitination assay, and the STORM assay for PARP trapping.

1.7.2 Additional exploratory assay to be named later.

2. BACKGROUND

2.1 CTEP-Supplied Investigational Agent (ABT-888)

ABT-888 is an orally available, small molecule inhibitor of poly(ADP-ribose) polymerase (PARP). PARP is an essential nuclear enzyme that plays a role in recognition of DNA damage and facilitation of DNA repair. Therefore, inhibition of PARP is expected to enhance the effects of DNA damage. Expression of PARP is higher in tumor cells as compared to normal cells. This overexpression has been linked to drug resistance and the ability of tumor cells to withstand genotoxic stress. Hence, it is anticipated that PARP inhibitors will function as sensitizing agents for chemotherapy and radiation therapy that are designed to cause DNA damage.

Mechanism of Action

Poly (ADP-ribosyl)ation (PAR) occurs after single or double-stranded DNA damage and represents the posttranslational modification of histones and other nuclear proteins by PARP. Based on conserved genetic sequences, encoded for by 18 different genes, 18 nuclear proteins have been classified as members of the PARP superfamily. The superfamily is further subdivided into three branches, the PARP-1 group, the tankyrase group, and other PARP enyzmes. The PARP-1 group of NAD^+-dependent enzymes has been extensively studied, and its members PARP-1 and PARP-2 are generally considered as the primary enzymes involved in DNA repair (1).

PAR has been implicated in many cellular processes including replication, transcription, differentiation, gene regulation, protein degradation, and spindle maintenance. Enhanced PARP-1 expression and/or activity in tumor cells, as compared to normal cells, has been demonstrated in malignant lymphomas(2), hepatocellular carcinoma(3), cervical carcinoma (4), colorectal carcinoma (5), non-Hodgkin's lymphoma (6), leukemic lymphocytes (7), and colon adenomatous polyps (8). PARP-1 and PARP-2 are nuclear proteins and are the only members of the PARP family with zinc-finger DNA binding domains. These domains localize PARP-1 and PARP-2 to the site of DNA damage. PARP-1 is highly conserved and has three structural domains (N-terminal DNA-binding domain; automodification domain, and the NAD^+-binding domain). The catalytic domain is located at the C-terminus end of the protein. In knockout mouse models, deletion of PARP-1 is sufficient to impair DNA repair (9-11). The residual PARP-dependent repair activity (~ 10%) is due to PARP-2. This suggests that only PARP-1 and PARP-2 need to be inhibited to impair DNA repair (12-14).

The zinc finger domain of PARP binds to both single- and double-stranded DNA breaks, resulting in increased catalytic activity (12,14,15). Once activated, PARP cleaves NAD^+ and attaches multiple ADP-ribose units to the target nuclear protein. This results in a highly negative charge on the target protein and affects its function. Overactivation of PARP can be induced by DNA damage, leading to the depletion of NAD^+ and energy stores and, thus, cellular demise by necrosis. An alternate mechanism has been identified where PARP overactivation can induce cell death through apoptosis by releasing the Apoptosis Inducing Factor (AIF) from mitochondria (16). Consequently, multiple mechanisms to prevent overactivation of PARP exist. First, auto-PAR negatively regulates PARP activity (17). In addition, the cleavage of

PARP by caspases yields a peptide fragment that acts as a trans-dominant negative inhibitor for uncleaved PARP. PAR of proteins is a dynamic process with a short half-life ($t_{1/2}$) of <1 min. The enzymes responsible for degrading these polymers are poly(ADP-ribose) glycohydrolase (PARG), which cleaves ribose-ribose bonds, and ADP-ribosyl protein lyase, which removes the protein proximal to the ADP-ribose monomer.

Increased PARP activity is one of the mechanisms by which tumor cells avoid apoptosis caused by DNA damaging agents. PARP activity is essential for the repair of single-stranded DNA breaks through the base excision repair (BER) pathways (14,18). Therefore, inhibition of PARP sensitizes tumor cells to cytotoxic agents (*e.g.* alkylators [temozolomide, cyclophosphamide, BCNU] and topoisomerase I inhibitors [irinotecan, camptothecin, topotecan]) which induce DNA damage that would normally be repaired through the BER system. A significant therapeutic window appears to exist between a PARP inhibitor's ability to potentiate therapeutic benefit *versus* potentiation of undesirable side effects. As expected, PARP inhibitors do not potentiate agents that do not cause DNA damage.

Ionizing radiation induces both double- and single-stranded DNA breaks. While part of the radiosensitization caused by PARP inhibition is through the inhibition of the single-stranded break repair pathways, it appears likely that repair of double-stranded breaks, which are thought to be more cytotoxic, is also affected. Double-stranded breaks are strong activators of PARP-1, resulting in PARP-1 mediated activation of DNA-PK and Ku80, important components of the non-homologous end-joining (NHEJ) double-stranded break repair pathway (19,20). Also, small molecule inhibitors of PARP can directly inhibit the repair of double-stranded breaks (9,21). Thus, it is likely that PARP activity is important for repair of both the single- and double-stranded stranded DNA breaks caused by ionizing radiation.

Nonclinical Activity

In vitro, ABT-888 inhibited PARP-1 and PARP-2 with K_i values of 3.6 nM and 2.9 nM, respectively. These values were observed in enzyme assays measuring the incorporation of $[^3H]$-NAD^+ into histone H1, an important physiological substrate of PARP. In assays measuring inhibition of H_2O_2-induced poly(ADP-ribosyl)ation in C-41 cervical carcinoma cells, ABT-888 inhibited PARP with an EC_{50} value of 2.4 nM. The extent of DNA damage in cells was indicated by γ-H2AX levels. To determine the effect of ABT-888 in combination with cytotoxic agents on DNA damage, the cellular content of γ-H2AX in C-41 cells was assayed by flow cytometry using an anti-γ-H2AX antibody. Addition of 1 mM of temozolomide alone resulted in increased numbers of γ-H2AX foci, a result which was further potentiated by ABT-888 in a dose-dependent manner. When cell survival was measured by an AlamarBlue assay, ABT-888 potentiated cytotoxicity in the same concentration range as used in the γ-H2AX assay, demonstrating that ABT-888 potentiates cytotoxicity of temozolomide by delaying DNA repair. ABT-888 achieved a maximal potentiation of approximately 15-fold. ABT-888 also potentiates the DNA damage cause by irinotecan.

The combination of PARP inhibitors with different classes of chemotherapeutics was examined. Cisplatin-induced potentiation was observed in a long-term clonogenic

assay, but not in the short-term cytotoxicity assay. The potentiation of cisplatin by ABT-888 *in vitro* is consistent with the potent enhancement of the efficacy of platinum agents (cisplatin and carboplatin) observed *in vivo*. PARP inhibition was shown to sensitize cells that are mismatch repair (MMR)-deficient to a greater extent than cells that are MMR competent (22). Alkylating agents such as temozolomide form methyl adducts in DNA and resistance is frequently encountered in the clinic with either the overexpression of O^6-alkylguanine DNA alkyltransferase (AGT) or functional defects in the MMR system. However, when PARP was inhibited, cells were sensitized to methylpurine formation, regardless of their resistance factors (23).

There are data to suggest that PARP inhibitors have activity against some BRCA-deficient cells in the absence of any DNA damaging agent (24,25). These inhibitors did not demonstrate single agent activity in BRCA-competent cells, and restoring functional BRCA to deficient cells abrogated single agent cytotoxicity. It is possible that, in BRCA-deficient cells, PARP inhibition stops the BER pathway, and thus single-stranded breaks are carried through DNA synthesis, resulting in double-stranded breaks. The increase in double-stranded breaks cannot be repaired by homologous recombination (HR), due to the lack of BRCA1 or 2, resulting in increased cell death. However, since not all BRCA deficient cells are sensitive to the PARP inhibitors, it is unclear why single agent cytotoxicity is observed in some BRCA-deficient cells.

Consistent with PARP-1 being a radiosensitization target, PARP-1 knockout mice showed enhanced sensitivity to γ-radiation (26,27). There is evidence to suggest that PARP inhibitors sensitize cancer cells to radiation, both *in vitro* and *in vivo* (28-30). Furthermore, a PARP inhibitor in the same class as ABT-888 potentiated radiation in the HCT116 colon carcinoma model. ABT-888 was tested, in combination with cytotoxic agents, in several tumor models and demonstrated a similar profile of antitumor activity to that seen in the literature (See table 1 below). ABT-888 substantially increased the efficacy of cytotoxic therapies, when measured by either treated/control tumor volumes (%T/C) or by increased time for tumors to grow to a particular size (%ILS). Potentiation of CPT-11 (80 mg/kg/day IP) activity was observed in B16F10 mouse xenograft studies when administered in combination with oral ABT-888 (25 mg/kd/day).

Table 1: Preclinical data for ABT-888 mediated potentiation of cytotoxic agents

	**Breast carcinoma (human MX-1)**	**Glioblastom a muliforme (rat 9L)**	**B cell lymphoma (human DOHH2)**	**Melanoma (murine B16F10)**
Carboplatin	Yes			
Cisplatin	Yes		No	
Cyclophosphamide	Yes			
Irinotecan				Yes
Temozolomide		Yes		Yes

ABT-888 potentiated cytotoxic therapy when administered either parenterally or orally (PO). When administered parenterally, significant efficacy was observed at doses as low as 1 mg/kg/day, and maximal efficacy was achieved at approximately 12.5 mg/kg/day. 3.1 mg/kg/day PO (divided, twice daily) provided significant potentiation, with maximal potentiation achieved at approximately 25 mg/kg/day. No increased

toxicity was observed at any of these ABT-888 doses, either parenteral or PO. Supratherapeutic doses of ABT-888 (50 mg/kg/day), administered via osmotic minipump (OMP), resulted in skin toxicity at the pump implantation site. The observation that supratherapeutic doses of PARP inhibitors may potentiate toxicity is consistent with preclinical and clinical observations. It is also consistent with the results from a two-week ABT-888/cisplatin combination study. When administered as a continuous infusion, an ABT-888 C_{ss} (plasma concentration at steady-state) of 70 ng/mL was maximally efficacious (area under the curve [AUC]=1.7 µg•hr/mL). Comparable efficacy was seen in oral studies at a 25 mg/kg/day (divided, twice daily) dose that yielded AUCs between 1.6 and 3.0 µg•hr/mL. At this dose, the plasma concentrations were above 70 ng/mL for only 2-4 hours per dose, demonstrating that 24 hour/day coverage above 70 ng/mL was not required for efficacy.

An enzyme-linked immunosorbent assay (ELISA) that can measure PAR formation was used to demonstrate PARP inhibition in murine tumors *in vivo* and human peripheral blood mononuclear cells (PBMCs) *ex vivo* at clinically relevant doses. This ELISA was used as the primary assay for PARP biomarker analysis. The degree of PARP inhibition was assessed in B16F10 syngeneic flank tumors from mice treated *in vivo* using tumor efficacy schedules. In this study, PAR formation was measured in tumors treated with ABT-888 alone. Two hours after administration, ABT-888 inhibited PAR formation in B16F10 tumors in a dose-dependent manner. The same response was reflected in a parallel efficacy experiment, where temozolomide (50 mg/kg/day, PO, daily × 5) was administered with ABT-888. In another study, PAR formation was measured in tumors treated simultaneously with temozolomide and ABT-888. As in the ABT-888 only study, tumor PAR levels in the combination study were also inhibited. Inhibition of PARP activity was significant at 12.5, 5 and 1 mg/kg/day in both the vehicle and temozolomide treated groups. Overall, these results indicate the ability of ABT-888 to inhibit both baseline and cytotoxic-induced PARP activity in tumors treated *in vivo* and provide evidence of the ability of ABT-888 to target PARP *in vivo.*

Inhibition of PAR was similarly analyzed with *ex vivo* treatment of human PBMCs from eight healthy volunteers. The cells from one of the eight volunteers showed no detectable PARP activity, while in another patient, PARP activity was not assessable by the assay. In the remaining six individuals, not only were baseline levels of PAR detected, but more importantly, a dose-dependent inhibition of PAR was observed with *ex vivo* treatment with ABT-888. Inhibition occurred at 10 nM (2.4 ng/mL), and PAR formation was almost eliminated at 300 nM (71 ng/mL).

Nonclinical Pharmacology and Toxicology

The pharmacokinetics (PK) of ABT-888 were evaluated in CD-1 mice, Sprague-Dawley rats, beagle dogs and cynomolgus monkeys. The non-clinical PK profile of ABT-888 was characterized by high plasma clearance (CL) values, ranging from a high of 4.1 L/hr•kg in the mouse to a low of 0.57 L/hr•kg in the dog. ABT-888 exhibits moderate volumes of distribution (V_{ss}) in all species (V_{ss} > 2.0 L/kg), with terminal elimination $t_{1/2}$ in the 1.2-2.7 hr range. In rats and dogs, [^{3}H]ABT-888 was rapidly absorbed and cleared primarily in the urine as intact parent drug. A-925088 (M8), a lactam derivative and the major product of ABT-888 metabolism, was also cleared primarily in the urine. In both rats and dogs, parent drug was the major component in

systemic circulation, followed by M8. Elimination of total radioactivity was rapid, with most (>80%) of the dose recovered within 24 hours post-dose, indicating that parent drug and the major metabolites are not likely to accumulate. Bioavailability following an oral dose was high (F>50%) in all species, with values ranging from a low of 56.1% in the monkey to a high of 92.0% in the mouse, and low animal-to-animal variability across all species.

The bioavailability from a non-formulated capsule was only slightly lower than from the solution formulation with values of 59.7% and 65.5% in fasted and non-fasted dogs, respectively. This suggests that there are no major food effects. The compound has high solubility at physiological pH and high permeability. Protein binding values in plasma (assessed *in vitro* as % bound at 5 µM) for ABT-888 were moderate in all species averaging 42% in dog, 41% in monkey, 43% in mouse, 49% in rat and 51% in human. The stability of ABT-888 was evaluated in rat, dog, monkey and human plasma and the drug was found to be very stable, with minimal degradation over the 8-hour incubation interval. *In vitro* metabolism studies indicated that several CYPs (1A1, 1A2, 2C9 and 2C19) have the potential to mediate the formation of M8. However, ABT-888 is not a potent inhibitor of the major human CYPs *in vitro,* indicating a low risk for drug-drug interactions at the anticipated therapeutic concentrations. ABT-888 partitioned slowly into and out of the brain, in both mouse and rat, with high plasma to brain ratios (~3:1) during the first 3-6 hours after dosing. The plasma to brain ratios approached 1:1 in samples obtained 12 hours after dosing.

PK parameters in humans were estimated by a variety of methods. The oral clearance (CL/F) of ABT-888 was estimated as a function of the projected clearance after IV administration (CL) and the fraction of the dose systemically available after oral administration (F). Clearance predictions were based on allometric scaling. Bioavailability was estimated by simulations with sensitivity analyses using software which took into account human gastrointestinal physiology and the drug's physicochemical characteristics. V_{ss} was estimated either from an average of values observed in animal species, a method averaging the fraction unbound in animal tissues, or by allometric scaling. Terminal phase $t_{1/2}$ values were estimated either by regression relationships between animal and human $t_{1/2}$ values (31), or from the estimates of CL and V_{ss}. The human PK profile is projected to have CL=26 L/hr, with oral bioavailability of ~ 70%. The predicted human $t_{1/2}$ of ABT-888 is ~4 hrs. Simulations of 50 mg twice daily dosing in humans mimic a maximally efficacious dosing regimen in mouse (12.5 mg/kg, twice daily), with concentrations above 71 ng/mL for 8 of 24 hours and an AUC_{24} of 3 µg•hr/mL at steady state.

ABT-888 was tested in receptor-binding, CNS/neurobehavioral, cardiovascular, cardiac electrophysiological and gastrointestinal assays. In 74 receptor-binding assays at a concentration of 10 µM (2.4 µg/mL), ABT-888 displaced control-specific binding at the human H_1 (61%), the human 5-HT_{1A} (91%), and the human 5-HT_7 (84%) sites only, with IC_{50} values of 1.2-5.3 µM.

ABT-888 did not display clear adverse CNS effects in the rat and mouse between 3-30 mg/kg PO. At 100 mg/kg PO, mild sedation-like effects were observed, followed in time by mild excitation. At 300 mg/kg PO, more moderate to marked CNS effects were

observed, including abnormal gait and sedation. Further, at 100 mg/kg, PO, there was an increased incidence of death after electrically-induced tonic convulsions in mice. Death was also noted in a second convulsant model (audiogenic seizures in mice). In a repeated dosing mini-Irwin observational test, in which rats were dosed with ABT-888 at 30, 100, and 300 mg/kg intraperitoneally (IP) every day for 5 days, tonic-clonic seizures/death were observed in approximately 50% of the animals treated at the highest dose on day 1. A similar incidence of seizures was observed after dosing the remaining animals at the same dose on each of the subsequent days. In an acute follow-up study with rats dosed with ABT-888 300 mg/kg IP, protection against seizures was not provided by pretreatment with either valproic acid (300 mg/kg IP, 15 min prior to ABT-888) or diphenylhydantoin (75 mg/kg IP, 100 min prior to ABT-888). In a 2-week toxicology study, seizures were also noted in dogs treated with ABT-888 at either 60 mg/kg/day, 30 mg/kg twice daily, or 30 mg/kg every day. Plasma concentrations in dogs with seizures were in excess of 5.4 µg/mL (26-fold the predicted clinical C_{max} of 0.21 µg/mL).

In the anesthetized dog, ABT-888 produced no physiologically relevant changes in mean arterial pressure, heart rate, dP/dt_{max}, pulmonary arterial pressure, or systemic or pulmonary vascular resistance compared to vehicle controls at mean plasma concentrations as high as 4.45 ± 0.13 µg/mL (21-fold the predicted clinical C_{max} of 0.21 µg/mL). As mean plasma concentrations increased to 12.96 ± 0.92 µg/mL (62-fold), ABT-888 produced a modest reduction in mean arterial pressure (–16 ± 5% below baseline) and systemic vascular resistance (–10 ± 7% below baseline).

ABT-888 blocked hERG current with an IC_{50} value of 57.6 ± 1.7 µg/mL (236 ± 7 µM), a value 278-fold higher than the predicted clinical C_{max}. The M8 metabolite of ABT-888 (A-925088) minimally affected hERG at the highest concentration tested (81.5 µg/mL). While no effect on repolarization (*in vitro* action potential duration measures) was noted at the lowest measured concentration of ABT-888 (0.42 µg/mL, 2-fold higher than the predicted clinical C_{max}), ABT-888 prolonged the action potential duration at the intermediate and highest measured concentrations (4.8% and 18.6% prolongation at 4.22 ± 0.02 and 39.49 ± 0.70 µg/mL respectively), suggesting delayed repolarization risk between 20- and 190-times the C_{max}. There was a trend (7%) towards delayed repolarization in the anesthetized dog model (QTc intervals) at plasma concentrations 21-fold higher than the predicted clinical C_{max}; greater concentrations elicited prolongation (15 ± 3% above baseline [QTcV] at 12.96 ± 0.92 µg/mL). In humans, QTc prolongation is predicted to be less than 3 msec at the anticipated dose of 50 mg twice daily. These cardiac effects need to be monitored during clinical trials.

Gavage administration of ABT-888 up to 10 mg/kg was generally well tolerated in the ferret emesis model. No emesis was noted at this dose (resulting in mean plasma concentrations of 3.80 ± 0.11 µg/mL, a value 18-fold greater than the predicted C_{max}), with significant emesis noted in response to the 20 mg/kg dose (resulting in mean plasma concentrations of 6.61 ± 0.26 µg/mL, a value 31-fold greater than predicted C_{max}). Parenteral (subcutaneous) dosing of ABT-888 at doses and plasma concentrations similar to those used in the gavage study revealed a similar emetic dose-response relationship, suggesting a centrally-mediated emetic response. ABT-888 had no significant effect on gastrointestinal transit up to 100 mg/kg (resulting in a

mean plasma concentration of 1.63 ± 0.14 µg/mL, a value 7-fold greater than the predicted clinical C_{max}).

ABT-888 dihydrochloride was evaluated in repeated dose toxicity studies in rats and dogs. When administered as a sole agent to rats, the compound did not result in adverse effects at C_{max} values that were greater than 19-fold the estimated therapeutic peak plasma drug concentration (highest dose tested). When rats were administered ABT-888 dihydrochloride in conjunction with a cytotoxic agent (cisplatin), no clinically meaningful exacerbations of cisplatin-associated toxicity were apparent at C_{max} values that were up to 8-fold greater for ABT-888 than the estimated therapeutic value. Exacerbation of cisplatin-associated toxicity was limited to rats that received ABT-888 dihydrochloride in conjunction with cisplatin at the highest dose that yielded C_{max} values 22-fold greater than the estimated therapeutic peak plasma drug concentration. In dogs, emesis, body weight losses related to anorexia, and convulsions were observed at doses of 30 mg base/kg/day with C_{max} values 26-fold greater than the estimated therapeutic peak plasma concentration. ABT-888 induced chromosome aberrations in human lymphocytes without metabolic activation and it was clastogenic in the *in vivo* rat micronucleus assay.

The no-observed-effect level (NOEL) in the rat and dog were 25 mg/kg/day divided BID and 15/10 mg/kg/day divided BID, respectively. Emesis and QT prolongation were observed in animal models, at 31-fold and 21-fold higher concentrations than the predicted clinical C_{max} (0.21 µg/ml), respectively. Based on different sensitivities to seizures between rodents and dogs, the plasma concentration that would be associated clinically with pro-convulsant activity will be difficult to define.

Clinical Investigations

A single-dose pharmacokinetic and pharmacodynamic endpoint study in cancer patients was initiated under an exploratory IND by the National Cancer Institute as the initial study in their phase 0 program. In this study, participants had baseline assessments of PAR in peripheral blood mononuclear cells (PBMCs) and at higher dose levels, in tumor from needle biopsies, assessed by a validated immunoassay. Participants received a single dose of ABT-888 at 10, 25, or 50 mg. PBMCs were collected over a 24 hour period at all dose levels, and tumor biopsies were obtained at the 25 mg dose level, approximately 3 to 6 hours after administration of ABT-888. A total of 6 patients have been studied so far, 3 each for the 10 mg and 25 mg cohorts. No treatment related adverse events have been observed. The target plasma C_{max} of 210 nM was exceeded in 2 of 3 patients at the 10 mg dose level, and in all three patients for at least 4 hours at the 25 mg dose level. Levels of PAR were reduced 80-99% from baseline levels after administration of ABT-888 in both the PBMCs and tumor samples at the 25 mg dose level. Thus, there is reason to believe that target inhibition is seen at least at the 25 mg dose level, and may be occurring at doses lower than 25 mg.

As discussed above, the NOEL in the rat and dog were 25 mg/kg/day divided BID and 15/10 mg/kg/day divided BID, respectively. While this would support a starting dose of 24 mg/day BID in a Phase 1 clinical trial, 20 mg/day (10 mg BID) was selected in this study since ABT-888 is administered in combination with CPT-11.

Preliminary safety, PD, and PK data have been determined from the dose escalation portion of NCI#7977. 32 pts have been treated to date (2 lung, 14 breast, 4 esophageal, 7 ovarian, 4 colon, 1 anal). Median age was 53 (range 31-73). Most frequent drug-related toxicities included: diarrhea (59%), nausea (59%), leucopenia (50%), fatigue (50%), neutropenia (44%), anemia (34%), and vomiting (31%). DLTs included fatigue, diarrhea, febrile neutropenia (gr 3), leukopenia and, neutropenia (gr 4). A Clinical Benefit (CB) rate of 56% (6 PR, 1 MR, 11 SD) was observed. ABT-888 exhibited linear PK over the dose range of 10 to 50 mg BID; co-administration of irinotecan did not affect PK of ABT-888. Exploratory analysis of pre/post-ABT-888 treatment changes indicated median ERCC1 levels in tumor tissue and PBMCs differed somewhat by clinical benefit status. PAR levels in paired tumor biopsies showed > 50% reduction in 17 of 20 pts (85%) and > 90% reduction in 7 of 20 (35%) pts. The MTD and recommended Phase II dose was established as 100 mg/m^2 of irinotecan given i.v. Days 1 and 8 combined with 40 mg of ABT-888 given BID 15 days on/6 days off (21 day cycle). Initial PBMC and tumor findings will be further evaluated in the expansion portion of this study and compared to circulating tumor cells (CTCs).

2.2 Irinotecan (CPT-11, NSC#616348)

Irinotecan (CPT-11) is a derivative of camptothecin. Camptothecins interact specifically with the enzyme topoisomerase I which relieves torsional strain in DNA by inducing reversible single-strand breaks. Irinotecan is a prodrug, which is converted to the active compound SN-38 by plasma and cellular carboxylesterases, and is approved for use in the treatment of metastatic colon and rectal carcinomas (32). Irinotecan and its active metabolite SN-38 bind to the topoisomerase I-DNA complex and prevent relegation of these single-strand breaks. Current research suggests that the cytotoxicity of irinotecan is due to double-strand DNA damage produced during DNA synthesis when replication enzymes interact with the ternary complex formed by topoisomerase I, DNA, and either irinotecan or SN-38. Mammalian cells cannot efficiently repair these double-strand breaks.

Although irinotecan has proven anticancer activity, resistance to the drug is a critical clinical problem (33). It is likely that clinical resistance to irinotecan is the result of (1) inadequate accumulation of drug in the tumor, (2) resistance-conferring alterations in topoisomerase I, or (3) alterations in the cellular response to the topoisomerase I–irinotecan interaction (33).

For this study, irinotecan will be administered at the indicated dose (100 mg/m^2) by infusion over 90 minutes on Days 1 and 8 of each 21-day cycle. This regimen was selected based on toxicity data obtained using similar regimens in European phase I dose-escalating trials in patients with solid tumors (34). The dose regimen selected allows a high amount of overall dose intensity over the entire cycle with a lesser amount of toxicity compared to other regimens (e.g. a q3 weekly schedule) and is convenient for outpatient use.

Recent publications(35,36) suggest that pretreatment UGT1A1 genotyping is recommended when using high dose irinotecan regimens which is not the case in this study; therefore, UGT1A1 testing is no longer required.

2.3 Rationale

A novel molecular approach to enhance the antitumor activity of topoisomerase I inhibitors relies on the use of chemical inhibitors of PARP (30,37-40). Tentori et al. recently published a report describing the effects of the combination of irinotecan with the PARP inhibitor GPI 15427 (39). The dose-limiting toxicity of irinotecan is delayed diarrhea, which has been attributed to SN-38, generated from irinotecan by intestinal carboxylesterases or from the SN-38 glucuronide present in bile by mucosal and bacterial β-glucoronidase. When DNA damage resulting from irinotecan treatment is severe, PARP-1 overactivation causes extensive NAD^+ consumption during the synthesis of ADP-ribose polymers, which eventually leads to ATP depletion and cell death (39). Oral administration of GPI 15427 reduced damage of jejunum and severity of delayed diarrhea in animals after irinotecan treatment (39). The amelioration of intestinal damage was associated with reduced ADP-ribose polymers formation in the intestinal epithelium, suggesting a role for PARP-1 overactivation in the pathogenesis of irinotecan toxicity (39). The study demonstrated that PARP-1 overactivation is involved in intestinal toxicity induced by irinotecan and that PARP inhibition provides protection from such untoward effect (39). The ability of GPI 15427 to prevent irinotecan intestinal toxicity might be attributed to the higher concentrations of the PARP inhibitor reached in the gut by means of oral administration.

It is vitally important to continue to develop new strategies designed to enhance the efficacy and reduce toxicity of chemotherapy in advanced solid tumors. This is a Phase 1, open label study assessing the safety, tolerability, pharmacokinetic (PK) profiles, and pharmacodynamic (PD) profiles of the PARP inhibitor ABT-888 in combination with irinotecan. The optimal biologic dose (OBD) of the combination will be established, defined as the maximal decrease in PARP. The patient population to be studied will consist of subjects with advanced solid tumors, with preference to tumors of the breast, lung, pancreas, colon, or ovary.

2.3.1 Dose Escalation for Intermittent ABT-888 Cohort

We will be investigating intermittent ABT-888 dosing, as well as higher dose levels in an escalating manner as the initial dose escalation portion of the study demonstrated that the combination was tolerable.

Of interest is to determine whether patients with tumor types known to have homologous recombination repair defects, such as those arising in patients with BRCA germline mutations, are more likely to benefit from the combination. It is possible that a functioning HR pathway may repair the double-strand breaks caused by irinotecan, irrespective of the presence of a PARP inhibitor. Indeed, partial responses were seen in both BRCA carriers and BRCA WT patients. The expansion cohort for this study was conducted in which the combination of veliparib and irinotecan was administered at the MTD established in this trial in two triple-negative breast cancer populations, including those with and without BRCA germline mutations. Pre-treatment biopsies were analyzed by whole exome sequencing to assess the homologous recombination-repair proficiency of tumors. Additionally, because XPF/ERCC1 represents an alternative pathway for the removal of topoisomerase I cleavable complexes, the expression of ERCC1 at

baseline and in response to treatment was analyzed, to test the hypothesis that patients with low ERCC1 levels may be most likely to benefit. In the expansion portion, we did see a higher response rate in those patients with BRCA-mutant triple-negative breast cancer.

2.4 Correlative Studies Background

2.4.1 Immunoassay for poly-ADP-ribosylated (PAR) substrates

Because the product of the PARP enzyme is poly-ADP-ribosylated (PAR) molecules, an immunoassay to quantify the amount of cellular PAR was developed as a clinical biomarker of PARP inhibition. Abbott Laboratories and the NCI-Frederick laboratories developed and cross-validated a quantitative immunoassay for PAR (41). The validated assay is a sandwich enzyme chemiluminescence immunoassay employing commercially obtained antibodies to PAR, and pure PAR as a standard. Assay dynamic range is 31 to 2000 pg/ml PAR, with a lower limit of quantitation of approximately 15 pg/ml PAR. The standard curve is linear throughout the range (with an adjusted R2 typically better than 0.98). The assay uses high, midrange, and low controls produced from the human melanoma line Colo829. Specimen handling was optimized for both PBMCs and tumor needle biopsies (18 ga), and harmonized for use with the same standards and controls. Specimens could be subjected to at least 3 freeze-thaw cycles without a detectable loss of antigen binding. Assay precision was determined at both Abbott and the NCI-Frederick, to be better than 80% (estimated total imprecision at Abbott, 7% or less). Accuracy, as assessed by spike recovery of pure PAR into PBMC lysates, was 100% +/- 20%. Assay dilution linearity was established for the Colo829 controls and tumor lysates, although deviations from linearity are observed in some tumor homogenates, and assay conditions are controlled to compensate for that lack of linearity. The validated assay was used to measure PAR levels in PBMCs of healthy donors, in animal models after administration of a single and multiple dosing of ABT-888, and has been used successfully in real time to measure PAR in PBMCs and tumor biopsies in a phase 0 clinical trial at the NCI.

The Dose Escalation for Intermittent ABT-888 Cohort will not be assessed for PAR, PK because it was already completed in the previous schedule.

2.4.2 <u>Pharmacokinetic (PK) Evaluation</u>

The study of systemic disposition of CPT-11 and ABT-888 will be conducted in all treated patients. It will include plasma analysis of irinotecan alone, ABT-888 alone, and the combination of the two drugs. Samples will be obtained at the following times:

Sampling time:

- <u>Cycle 1, Day 1, 2, and 3 </u>: (to characterize the PK of CPT-11 alone):
  Blood samples will be collected at: pre-infusion (8ml), 30 min after the start of infusion (4ml), immediately at the end of infusion (EOI) (~89 min, 4ml),

and 2h (4ml), 3.5h (8ml), 5.5h (8ml), 8.5h (8ml) 28h (8 ml) and 48h (8ml) after the start of infusion. Blood drawn at pre-infusion, and 3.5 hours, 5.5 hours, 28 hours, and 48 hours after the start of infusion will be used for separation of plasma (for PK assay) and PBMC (for either PAR assay or PD assay, see Appendix C).

- Cycle 2, day -1 and 1: (to characterize the PK of ABT-888 alone)
  A single-dose of ABT-888 will be given on Day -1 while in the clinic, and blood samples will be obtained at: pre-dosing (8ml), 0.5h (4ml), 1h (4ml), 1.5h (4ml), 3.5h (4ml), 5.5h (8ml), 8.5h (4ml), 10h (4ml), and 28h (8ml) after the dosing. Blood drawn at pre-dosing, 5.5 hours, and 28 hours after the dosing will be used for separation of plasma (for PK assay) at KCI and PBMC (for PD assay) at KCI (see SOP for PK-PBMC collection, appendix G). ABT-888 on Cycle 2, Day 1 will be taken twice, with the first daily dose taken after the 28h blood draw.
- Cycle 2, day 8, 9, and 10: (to characterize the steady-state PK of CPT-11 and ABT-888 in the combination)
  The first and second dose of ABT-888 to be administered on day 8 will be dispensed in the clinic. The first dose of ABT-888 will be taken at the start of CPT-11 infusion, so PK sampling can be lined up for both drugs. Blood samples will be collected at: pre-infusion (8ml), 30 min (4ml) and 1 hour (4ml) after the beginning of infusion, immediately end of infusion (EOI) (~89 min, 4ml), and 2h (4ml), 3.5h (4ml), 5.5h (8ml), 8.5h (4ml), 10h (4ml, just prior to the administration of the second dose of ABT-888 on day 8), 28 h (8ml, prior to ABT-888 administration on day 9), and 48 h (4ml, prior to ABT-888 administration on day 10). Blood drawn at pre-infusion, 5.5 hours after the infusion, and 28 hours after the infusion will be used for separation of plasma (for PK assay) and PBMC (for PD assay).

2.4.3 Pharmacodynamic (PD) Evaluation

PARP-1 is activated by DNA single-strand breaks (SSB) resulting from ionizing radiation and exposure to alkylating agents (30,42). The enzyme is involved in base excision repair (BER), but not nucleotide excision repair (Calabrese et al. 2004, Fig. 1) (30). PARP-1 loosens chromatin and recruits repair proteins. If single strand breaks (SSB) are not repaired, SSBs form DNA double strand breaks (DSB) upon DNA replication. The latter are then repaired *via* homologous recombination repair (HR). Important proteins in DSB DNA damage signaling and repair are gamma-H2AX, which is phosphorylated within minutes after double strand breaks occur; the tumor suppressor proteins BRCA1 and BRCA2, together with their partner proteins Rad50 and Rad51 respectively; as well as the excision repair enzyme ERCC1 (Fig. 1) (24,30).

Figure. 1. Mechanism of PARP inhibition. If base excision repair (BER) is blocked due to PARP inhibition, DNA-double strand breaks are induced and homologous recombination repair (HR) mechanisms are activated. Key players in DSB repair are gamma-histone 2AX (H2AX), Rad51 and ERCC1.

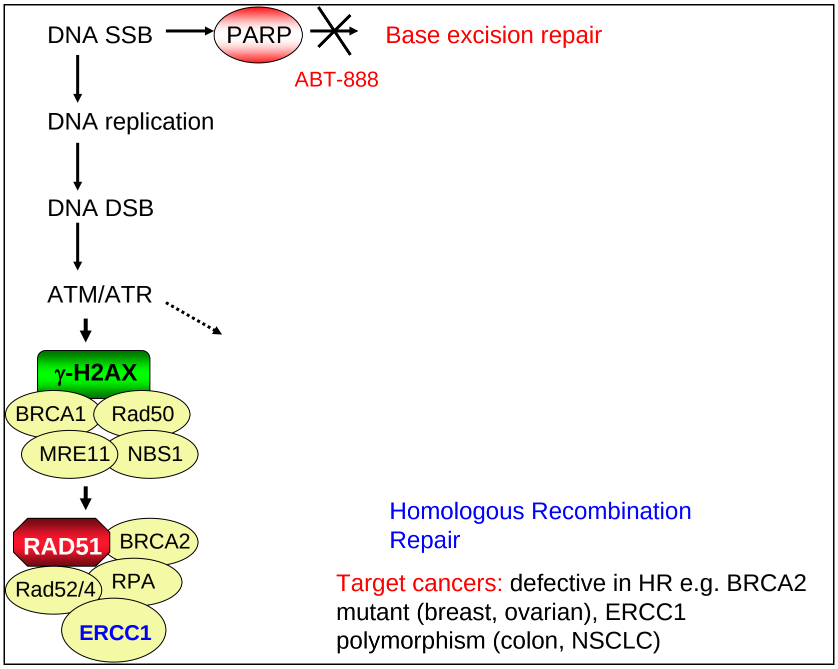

Temozolomide and topotecan have been shown to potentiate the activity of PARP inhibitors such as AG14361 (24,30,42). Temozolomide is an alkylating agent and camptothecins such as topotecan and irinotecan interact specifically with the enzyme topoisomerase I (Topo I), which relieves torsional strain in DNA by inducing reversible single-strand breaks. Reduced levels of Topo I expression; alterations in the structure of Topo I from different mutations; proteasome degradation of Topo I and/or enhanced DNA repair, have been shown to correlate with lack of response to irinotecan (43).

Optimal activity of PARP inhibition together with agents that induce SSB might be seen in tumor types that are deficient or mutant for proteins that are key players in the HR repair pathway (24). Based on DNA repair pathways described in Fig. 1, our proposal has been designed to study ABT-888 in combination with the topoisomerase I poison irinotecan. Target cancers which might respond best to PARP inhibition and thus, BER inhibition by ABT-888 might be breast or ovarian cancers with mutant BRCA2 or colon and lung cancers with polymorphism in ERCC1, thus lacking in additional HR repair (44).

Suitable PD endpoints are PAR activity inhibition in tumor cells (41,42,45), the detection of γ-H2AX foci and/or Rad51 foci in tumor biopsies (24). The determination of Topo I levels and ERCC1 status in archival paraffin blocks would also be helpful in evaluating the role of irinotecan and responsive tumors in the combination regimen (Fig. 1).

2.4.4 Pharmacogenomic Evaluation

Genetic polymorphisms in the metabolic enzymes and active transporters have been widely demonstrated to be related to the substantial inter-individual variability in drug pharmacokinetics, clinical outcomes, and toxicity profiles. For

example, the genetic variant UGT1A1*28, characterized by the presence of an additional TA repeat in the TATA sequence of the UGT1A1 promoter [$(TA)_7$TAA instead of $(TA)_6$TAA], has been repeatedly reported to be associated with reduced SN-38 glucuronidation and greater susceptibility to CPT-11 induced gastrointestinal and hematological toxicities (46-48). In addition, since CPT-11 metabolites (i.e., SN-38 and SN-38G) are good substrates for breast cancer resistance protein (ABCG2), polymorphisms of the ABCG2 gene, in particular functional ABCG2 421C>A allele, may affect the disposition and pharmacodynamic effects of CPT-11 (49).

Little is known on the pharmacokinetics and pharmacogenetics of ABT-888. *In vitro* metabolism studies indicate that CYP1A1, 1A2, 2C9, and 2C19 are important enzymes contributing to ABT-888 metabolism. Defective CYP2C9 alleles (e.g., CYP2C9*2 and *3) and CYP2C19 alleles (e.g., CYP2C19*2 and *3) may potentially affect the metabolism, and therefore, the pharmacodynamic effects of ABT-888.

Polymorphisms in UGT1A1 (e.g., UGT1A1*28 variant), CYP2C9 (e.g., CYP2C9*2 and *3 variants) and CYP2C19 (e.g., CYP2C19*2 and *3 variants), and ABCG2 (e.g., ABCG2 421C>A) will be determined using previously reported methods (50-53). Pharmacogenetic analyses will be performed in the Barbara Ann Karmanos Cancer Institute Pharmacology Core Laboratory.

2.4.5 **<u>Next Generation Molecular Profiling</u>**

TGen will sequence the genome and transcriptome from both normal and tumor tissue from each study patient in the two arms of the study using a three-pronged Next Generation Sequencing (NGS) approach. First, we will sequence exomes for both tumor and normal at roughly 100X coverage, allowing for sufficient read level power to detect point mutations (single nucleotide substitutions or small insertion/deletions) even in the face of high tissue heterogeneity (i.e. low tumor content). Second, we will sequence whole genomes of both tumor and normal using a long-insert library (>1.5 kb) at low coverage (~5X-10X). The large inserts will provide high ‘clonal’ or ‘physical coverage’ (>100X) to detect structural changes and copy number events occurring between paired or mated reads, recognizing base-level coverage will be approximately 8X outside exonic regions. Third, we will resequence RNA from the tumor and a matched normal tissue. This will allow for robust expression analysis and will provide a mechanism to interpret the consequence of a mutation. For example, our preliminary data shows three examples where a translocation, a moderate deletion, and a small deletion all impact expression of key genes (Figure 2). Additionally, this powerful and comprehensive genome and transcriptome approach delivers a comprehensive list of changes, that give way to full understanding of the pathway perturbations that occur in a tumor through multiple mechanisms further powering our abilities to detect therapeutically relevant concepts associated with drug sensitivity or resistance.

At the tumor somatic level, this strategy will impart the opportunity to detect: 1) somatic point mutations and small *indels;* 2) somatic copy number changes (amplifications and deletions); and 3) large *cis* rearrangements and translocations. Also at the tumor level, gene expression analysis by array-based technology gives way to RNA-seq with a far wider dynamic range, at the gene, exon, and in some cases allele-specific levels. At the constitutional plane, our strategy will also provide a profile for germline variants within highly penetrant cancer genes such as *BRCA1/2*, *TP53*, *CHK2*, or other genes defined by the Human Genome Mutation Database. Furthermore, we will have access to germline variants associated with steroid and drug metabolism (i.e., cytochrome P450, UDP-glucuronosyltransferase, N-acetyltransferases, GSTP1, ABCB1, XRCC1, ERCC2, and UGT1A1). Although not a direct focus of this study, we will generate data for the vast majority of germline variants for each individual within the study.

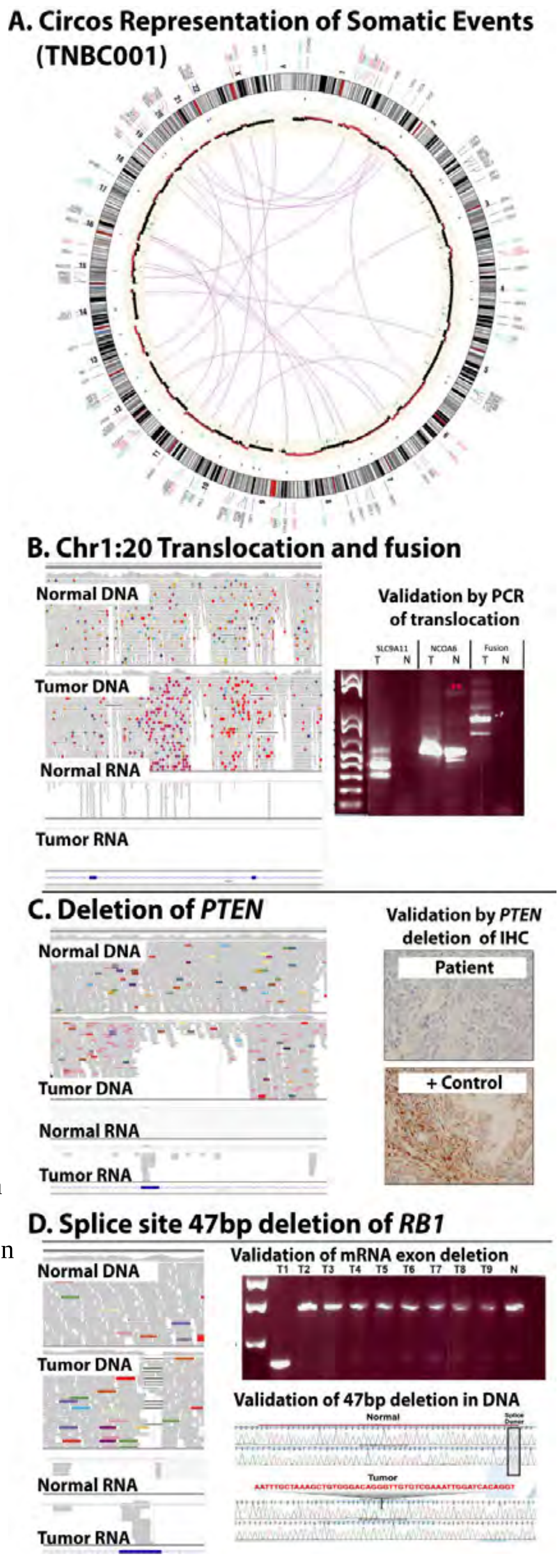

Figure 2. Integrated analysis and validation from NGS analysis for a Triple Negative Breast tumor. Within panels B-D, validation of NGS detected alterations at the DNA-level, RNA-level and protein level.

To support these activities, TGen will leverage its NGS technology platform which includes seven Life Technologies SOLiD 4 systems, four Illumina HiSeq2000 systems, two Illumina GAII systems, and one Roche 454 Jr. system. Furthermore, data processing and analysis will be supported by a

5500 parallel processor supercomputing environment and an informatics staff that are participants in the NIH 1000 Genomes initiative. This informatics team has developed custom pipelines for the discovery of somatic alterations from paired normal/tumor Exome-seq data, as well as RNA-seq data.

2.4.6 **PARP and Breast Cancer Stem Cells (BCSCs)**

To determine whether PARPi are able to specifically target breast cancer stem cells (BCSCs), we performed preliminary experiments to determine whether BCSCs in the BRCA1 deficient SUM149 cell line or BRCA1 wildtype SUM159 cell line were sensitive to PARP inhibition. As previously reported, PARPi showed a dose dependent effect with 90% reduction in tumor cell number of SUM149 after ten-day treatment (Figure 3A). However, this treatment resulted in a relative increase in the Aldefluor-positive population (Figure 3B), suggesting that the BCSCs in SUM149 cells were relatively resistant to PARP inhibition. Furthermore, we found that PARPi has no effects on both the total cell number and the Aldefluor-positive population in BRCA1 wildtype SUM159 cells (Figure 3C, 3D).

Although the molecular mechanism mediating CSC resistance to PARP inhibition in some TNBCs is not known, we have preliminary evidence that the DNA repair enzyme RAD51 may play a role. We have previously reported that CSCs in some breast cancer cell lines contain elevated levels of RAD51 (54). In our preliminary studies, utilizing western blot and RAD51 foci staining, we found that ALDH-positive SUM149 cells have higher level of total RAD51 protein and can form more active RAD51 foci after irradiation (Figure 4), but there is not much difference for RAD51 level in ALDH-positive cells and ALDH-negative cells in SUM159, in which both populations have high RAD51 level (Figure 5).

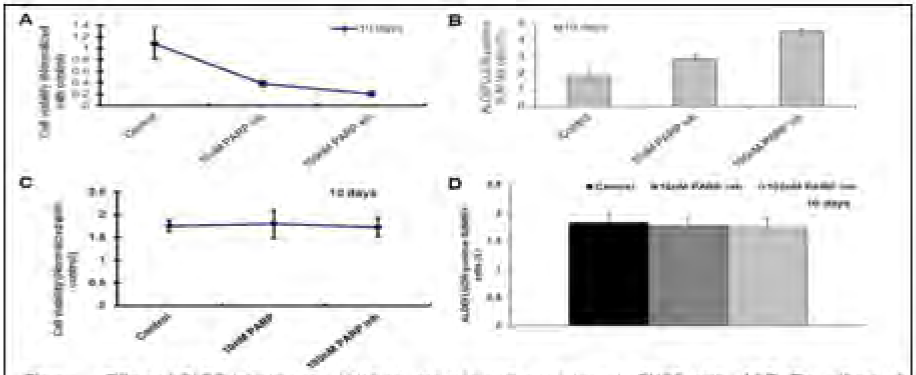

Figure 3: Effect of PARP inhibition on Aldefluor and total cell populations in TNBC cells. **A&B**. The effects of increasing concentrations of the PARPi on the Aldefluor-positive (stem cell) and total cell populations were determined in TNBC SUM149 cells which harbor a mutant BRCA1. After 10 days, the PARP inhibitor produced a dose-dependent decrease in overall cell viability (A), while increasing the relative proportion of Aldefluor-positive (stem-like) cells **(B)**. **C&D**. The effects of increasing concentrations of the PARPi on the Aldefluor-positive (stem cell) and total cell populations were determined in TNBC SUM159 cells which have wild-type BRCA1. After 10 days, the PARPi has no effects on overall cell viability (C), and no effects on the percentage of Aldefluor-positive (stem-like) cells **(D)**. These experiments demonstrate the CSCs in some TNBC lines are responding to PARPi differently.

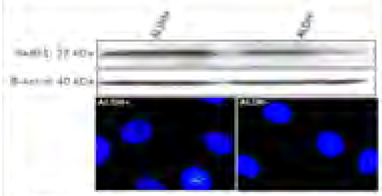

Figure 4: Rad51 protein level by immunoblot assay and immunofluorescence foci staining. ALDH-positive SUM149 cells have higher level of Rad51 protein and active Rad51 foci formation after irradiation.

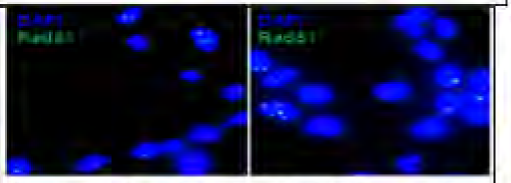

Figure 5: Rad51 protein level by immunoblot assay and immunofluorescence foci staining. ALDH-positive and ALDH-negative SUM159 cells have similar active Rad51 foci formation after irradiation.

2.4.7 The Dose Escalation for Intermittent ABT-888 Cohort

Development of a PARP 1 and 2 Trapping Assay. Recent research suggests that the ability of PARP1 and 2 to be trapped in the chromatin fraction of cells after treatment with a PARP inhibitor correlates with cytoxicity (55). The goal of our exploratory studies is to develop assays to detect trapping of PARP1 and 2 in tumor biopsy tissue in response to treatment with Irinotecan plus a PARP inhibitor, in this case ABT-888. We will work on the development of three assays and these include the subcellular fractionation assay, the FANCD2 monoubiquitination assay, and the STORM assay for PARP trapping.

The subcellular fractionation assay. This is based upon previous studies using the DU-145 prostate cancer cell line (55), in which cells were treated with various PARP inhibitors, lysed, fractionated, and western-blotted with antisera raised against PARP1 and PARP2. Our challenge is to expand this type of assay to tumor biopsy tissue. Our team has experience in lysis and cellular fractionation of tumor tissue, and also in western blotting. Our first goal is to import the subcellular fractionation assay to our laboratory. We have DU-145 cells. These cells

will be grown and treated with different concentrations of various PARP inhibitors including ABT-888 and olaparib, lysed, and subjected to cellular fractionation into nuclear and chromatin as described (55), based upon incubation with increasing salt concentrations. The fractions will be resolved by polyacrylamide gel electrophoresis (PAGE), blotted to nitrocellulose, and incubated with antisera raised against PARP1, PARP2, TOP1 (to mark the soluble nuclear fraction), histone H3 (to mark the chromatin fraction), and γ-tubulin as a loading control. Quantification will be as we describe (56), by normalization to γ-tubulin. We expect to repeat the results of the original assay (55) and demonstrate dose-dependent PARP1 and 2 trapping in the chromatin fraction.

Our second experiment will be a repeat of the above experiment but also include irinotecan, as this agent is used in the protocol. We expect to observe similar levels of PARP1 and 2 trapping as in the earlier experiment but realize that we may observe greater or lesser amounts of trapping upon addition of this drug.

Our third set of experiments will be with human triple negative breast tumor xenografts, harvested from untreated mice or mice treated with irinotecan alone or in combination with ABT-888, using doses and conditions that mimic the ones used in the clinical protocol. At least three tumors from each treatment, in addition to negative controls, will be homogenized and this lysate will be strained to separate the cells from the debris and fractionated using the Thermo Scientific Subcellular Fractionation Kit for Tissue. We have successfully fractionated cells from tissues using this kit. After fractionation, the samples will be processed as described above. We expect to observe PARP1 and 2 trapping in chromatin at levels similar to what is observed in cells treated with ABT-888. Optimization may be necessary to achieve these results and could include homogenization conditions, incubation and centrifugation times.

Once an optimized protocol is in place, we will initiate studies with core biopsy tissue. Two core biopsies will provide sufficient numbers of cells to perform up to 6 PARP trapping assays. However, we expect to require only three independent assays to reliably quantify trapping of PARP 1 and 2 in response to treatment with irinotecan and irinotecan plus ABT-888.

<u>The FANCD2 monoubiquitination assay</u>. Monoubiquitination of FANCD2 occurs in response to replication stress (57). Interestingly, cancer cells in which PARP1 is trapped in chromatin after treatment with various PARP inhibitors have been shown to have significantly increased levels of FANCD2 monoubiquitination in nuclear fractions (55). Therefore, this could serve as a biomarker for PARP trapping. In order to test this hypothesis, we will resolve aliquots of the of cytoplasmic and nuclear fractions with appropriate markers, as described above, on PAGE gels and immunoblot with antisera raised against FANC2, which can detect its monoubiquitinated form. Quantification of monoubiquitinated FANCD2 after normalization to tubulin in three replicate experiments will be performed. Statistics, using GraphPad Prism, will be performed to assess correlation between levels of PARP trapping and FANCD2 monubiquitination. A linear regression model that supports R2 for goodness of fit and a P value for the slope of the line being significantly different from zero will be used to assess a potential correlation. If a strong correlation exists it will be consistent with the idea that levels of monoubiquitinated FANCD2 correlate with levels of PARP trapping in chromatin. We will correlate with patient outcomes.

<u>The STORM assay.</u> This constitutes that most exploratory aspect of this proposal. Stochastic optical reconstruction microscopy (STORM) is a super-resolution technique for imaging of single

molecules within cells or tissues. This novel imaging technique has successfully detected clusters of RNA polymerase molecules within chromatin (58). In collaboration with Dr. David Warshaw, University of Vermont, and a world renowned expert in STORM imaging, we will develop this technique to image clusters of PARP1 molecules, using standard techniques (58). Chromatin will be visualized by immunostaining using antibodies against either H2B or linker H1 or both. PARP1 will also be visualized by immunostaining. Initially, conditions will include cells treated or not with PARP inhibitor. The distribution of chromatin width is likely to reflect PARP1 clustering such that trapping may result in either aberrantly tight or lose (more open) chromatin versus no trapping. Once these endpoints are established, we will image chromatin from tumor samples and correlate our quantitative measurements of chromatin width with PARP1 trapping levels in the fractionation assay of the same tissue. Our ultimate goal is to develop a relatively easy and efficient imaging assay to evaluate the ability of PARP inhibitors to trap PARP1 in tumor tissue.

3 PATIENT SELECTION

3.1 Eligibility Criteria

3.1.1 Patients must have histologically or cytologically confirmed diagnosis of malignancy that is metastatic or unresectable and for which standard curative or palliative measures do not exist or are no longer effective or for whom CPT-11 treatment would be a viable therapy regimen. Patients with solid hematologic malignancies (Hodgkin's and Non-Hodgkin's Lymphomas) may be included as long as a bone marrow has been performed within 6 weeks of treatment.

3.1.2 Patients enrolled on the expansion portion of the study will consist of two cohorts: those patients who are triple-negative, BRCA-mutant positive and those patients who have triple-negative, non-BRCA mutated breast cancer

3.1.3 Patients enrolled on the dose escalation for intermittent ABT-888 portion of the study must have histologically or cytologically confirmed diagnosis of malignancy that is metastatic or unresectable and for which standard curative or palliative measures do not exist or are no longer effective or for whom CPT-11 treatment would be a viable therapy regimen. Patients with solid hematologic malignancies (Hodgkin's and Non-Hodgkin's Lymphomas) may be included as long as a bone marrow has been performed within 6 weeks of treatment.

3.1.4 Patient must have measurable disease per Response Evaluation Criteria in Solid Tumors (RECIST) guidelines

3.1.5 Patients must have tumors determined to be easily accessible for biopsy (e.g. pleural-based lesions, peripheral lymph nodes, soft tissue metastases, large liver metastases, etc.)

3.1.6 Prior Therapy

3.1.6.1 Chemotherapy: Prior chemotherapy is allowed. Patients must not have received chemotherapy for 4 weeks prior to the initiation of study treatment and must have full recovery from any acute effects of any prior chemotherapy. Patients must not have had nitrosoureas or mitomycin C for 6 weeks prior to the initiation of study treatment.

3.1.6.2 Radiation: Prior radiation therapy is allowed. Patients must not have received minimal radiation therapy (≤5% of their total marrow volume) within 3 weeks prior to the initiation of study treatment. Otherwise,

patients must not have received radiation therapy (>5% of their total marrow volume) within 4 weeks prior to the initiation of study treatment. Patients who have received prior radiation to 50% or more of their total marrow volume will be excluded.

3.1.6.3 Other therapies: Prior experimental (non-FDA approved) therapies and immunotherapies are allowed. Patients must not have received these therapies for 4 weeks prior to the initiation of study treatment and must have full recovery from any acute effects of these therapies.

3.1.7 Age ≥18 years. Because no dosing or adverse event data are currently available on the use of ABT-888 in patients <18 years of age, children are excluded from this study, but may be eligible for future pediatric phase 1 combination trials.

3.1.8 ECOG performance status ≤2 (Karnofsky ≥60%, see Appendix A).

3.1.9 Life expectancy of greater than 12 weeks

3.1.10 Patients must have organ and marrow function as defined below:

3.1.10.1 Absolute neutrophil count (ANC) ≥1,500/mcL

3.1.10.2 Platelets (PLT) ≥100,000/mcL

3.1.10.3 Aspartate aminotransferase (AST) ≤ 2.5 x upper limit of normal (ULN); if liver metastases are present, ≤ 5 x ULN

3.1.10.4 Alanine aminotransferase (ALT) ≤ 2.5 x ULN; if liver metastases are present, ≤ 5 x ULN

3.1.10.5 Bilirubin ≤ 1.5 x ULN

3.1.10.6 Creatinine ≤ 1.5 x ULN

OR:

Calculated or measured creatinine clearance ≥ 60 mL/min/1.73 m^2 for patients with creatinine above institutional normal

3.1.11 The effects of ABT-888 on the developing human fetus are unknown. For this reason and because other therapeutic agents or modalities used in this trial are known to be teratogenic, women of child-bearing potential and men must agree to use adequate contraception (hormonal or barrier method of birth control; abstinence) prior to study entry, for the duration of study participation, and for three months following completion of study therapy. Should a woman become pregnant or suspect she is pregnant while participating in this study, she should inform her treating physician immediately.

3.1.12 Ability to understand and the willingness to sign a written informed consent document.

3.1.13 All patients must provide archival tissue block or paraffin sample from archival tissue block (approximately 10 sections) for use in pharmacodynamic correlative studies. (NOT required for patients enrolled on the dose escalation for intermittent ABT-888 portion of the study)

3.2 Exclusion Criteria

3.2.1 Patients who have had chemotherapy or radiotherapy within 4 weeks (6 weeks for nitrosoureas or mitomycin C) prior to entering the study or those who have not recovered from adverse events due to agents administered more than 4 weeks earlier. Patients who have been administered ABT-888 as part of a single or limited dosing study, such as a phase 0 study, should not necessarily

be excluded from participating in this study solely because of receiving prior ABT-888.

3.2.2 Patients may not have received any other investigational agents within 4 weeks of study entry.

3.2.3 History of allergic reactions attributed to the following: camptothecin derivatives (e.g. topotecan, irinotecan, exatecan), any ingredients contained within the liquid irinotecan solution (e.g. sorbitol), or any antiemetics or antidiarrheals appropriate for administration with study therapy (e.g. loperamide, dexamethasone)

3.2.4 Patients must not receive any other anti-cancer therapy (cytotoxic, biologic, radiation, or hormonal other than for replacement) while on this study except for medications that are prescribed for supportive care but may potentially have an anti-cancer effect (i.e. megestrol acetate, bisphosphonates). These medications must have been started 1 month prior to enrollment on this study. In addition, men receiving treatment for prostate cancer will be maintained at castrate levels of testosterone by continuation of luteinizing-releasing hormone agonists.

3.2.5 Patients with uncontrolled seizures

3.2.6 Patients with known active brain metastases should be excluded from this clinical trial because of their poor prognosis and because they often develop progressive neurologic dysfunction that would confound the evaluation of neurologic and other adverse events. Patients with prior treated brain metastases are allowed, providing that they were not accompanied by seizures and that a baseline brain MRI scan prior to study entry demonstrates no current evidence of brain metastases. All patients with CNS metastases must be stable for >3 months after treatment and off steroid treatment prior to study enrollment.

3.2.7 Any patient requiring chronic maintenance of white blood cell counts or granulocyte counts through the use of growth factor support (e.g. Neulasta®, Neupogen®)

3.2.8 Any patient requiring cytochrome P450 CYP3A4 isoform-inducing drugs (e.g. phenytoin, phenobarbital, carbamazepine, rifampin, rifabutin, ketoconazole, St. John's Wort) will be excluded due to the reduction of the exposure of irinotecan. CYP3A4-inducing drugs should be discontinued at least 2 weeks prior to the first cycle of irinotecan

3.2.9 Uncontrolled intercurrent illness including, but not limited to, ongoing or active infection, symptomatic congestive heart failure, unstable angina pectoris, cardiac arrhythmia, or psychiatric illness/social situations that would limit compliance with study requirements.

3.2.10 Pregnant women are excluded from this study because ABT-888 is PARP inhibitor with the potential for teratogenic or abortifacient effects. Because there is an unknown but potential risk for adverse events in nursing infants secondary to treatment of the mother with ABT-888, breastfeeding should be discontinued if the mother is treated with ABT-888. These potential risks may also apply to other agents used in this study.

3.2.11 Patients who are unable to reliably tolerate and/or receive oral medications.

3.3 Inclusion of Women and Minorities

Both men and women of all races and ethnic groups are eligible for this trial.

4. REGISTRATION PROCEDURES

4.1 Investigator and Research Associate Registration with CTEP

Food and Drug Administration (FDA) regulations and National Cancer Institute (NCI) policy require all individuals contributing to NCI-sponsored trials to register and to renew their registration annually. To register, all individuals must obtain a Cancer Therapy Evaluation Program (CTEP) Identity and Access Management (IAM) account (https://ctepcore.nci.nih.gov/iam). In addition, persons with a registration type of Investigator (IVR), Non-Physician Investigator (NPIVR), or Associate Plus (AP) (i.e., clinical site staff requiring write access to OPEN or RAVE or acting as a primary site contact) must complete their annual registration using CTEP's web-based Registration and Credential Repository (RCR) (https://ctepcore.nci.nih.gov/rcr). Documentation requirements per registration type are outlined in the table below.

Documentation Required	IVR	NPIVR	AP	A
FDA Form 1572	✔	✔		
Financial Disclosure Form	✔	✔	✔	
NCI Biosketch (education, training, employment, license, and certification)	✔	✔	✔	
HSP/GCP training	✔	✔	✔	
Agent Shipment Form (if applicable)	✔			
CV (optional)	✔	✔	✔	

An active CTEP-IAM user account and appropriate RCR registration is required to access all CTEP and CTSU (Cancer Trials Support Unit) websites and applications. In addition, IVRs and NPIVRs must list all clinical practice sites and IRBs covering their practice sites on the FDA Form 1572 in RCR to allow the following:

- Added to a site roster
- Assigned the treating, credit, consenting, or drug shipment (IVR only) tasks in OPEN
- Act as the site-protocol PI on the IRB approval.

Additional information can be found on the CTEP website at https://ctep.cancer.gov/investigatorResources/default.htm. For questions, please contact the RCR ***Help Desk*** by email at < RCRHelpDesk@nih.gov >.

4.2 Site Registration

This study is supported by the NCI Cancer Trials Support Unit (CTSU).

Each investigator or group of investigators at a clinical site must obtain IRB approval for this protocol and submit IRB approval and supporting documentation to the CTSU Regulatory Office before they can be approved to enroll patients. Assignment of site registration status in the CTSU Regulatory Support System (RSS) uses extensive data to make a determination of whether a site has fulfilled all regulatory criteria including but not limited to the following:

- An active Federal Wide Assurance (FWA) number
- An active roster affiliation with the Lead Network or a participating organization
- A valid IRB approval
- Compliance with all protocol specific requirements.

In addition, the site-protocol Principal Investigator (PI) must meet the following criteria:

- Active registration status
- The IRB number of the site IRB of record listed on their Form FDA 1572
- An active status on a participating roster at the registering site

Sites participating on the NCI CIRB initiative that are approved by the CIRB for this study are not required to submit IRB approval documentation to the CTSU Regulatory Office. For sites using the CIRB, IRB approval information is received from the CIRB and applied to the RSS in an automated process. Signatory Institutions must submit a Study Specific Worksheet for Local Context (SSW) to the CIRB via IRBManager to indicate their intent to open the study locally. The CIRB's approval of the SSW is then communicated to the CTSU Regulatory Office. In order for the SSW approval to be processed, the Signatory Institution must inform the CTSU which CIRB-approved institutions aligned with the Signatory Institution are participating in the study.

4.2.1 Downloading Regulatory Documents

Site registration forms may be downloaded from the 7977 protocol page located on the CTSU Web site. Permission to view and download this protocol is restricted and is based on person and site roster data housed in the CTSU RSS. To participate, Investigators and Associates must be associated with the Corresponding or Participating protocol organization in the RSS.

- Go to https://www.ctsu.org and log in using your CTEP-IAM username and password.
- Click on the Protocols tab in the upper left of your screen.
- Either enter the protocol # in the search field at the top of the protocol tree, or
- Click on the By Lead Organization folder to expand, then select LAO-CT018, and protocol #7977.

- Click on LPO Documents, select the Site Registration documents link, and download and complete the forms provided. (Note: For sites under the CIRB initiative, IRB data will load to RSS as described above.)

4.2.2 Requirements For 7977 Site Registration:

- IRB approval (For sites not participating via the NCI CIRB; local IRB documentation, an IRB-signed CTSU IRB Certification Form, Protocol of Human Subjects Assurance Identification/IRB Certification/Declaration of Exemption Form, or combination is accepted)

4.2.3 Submitting Regulatory Documents

Submit required forms and documents to the CTSU Regulatory Office, where they will be entered and tracked in the CTSU RSS.

Regulatory Submission Portal: www.ctsu.org (members' area) → Regulatory Tab →Regulatory Submission

When applicable, original documents should be mailed to:
CTSU Regulatory Office
1818 Market Street, Suite 3000
Philadelphia, PA 19103

Institutions with patients waiting that are unable to use the Portal should alert the CTSU Regulatory Office immediately at 1-866-651-2878 in order to receive further instruction and support.

4.2.4 Checking **Site** Registration Status

You can verify your site registration status on the members' section of the CTSU website.

- Go to https://www.ctsu.org and log in to the members' area using your CTEP-IAM username and password
- Click on the Regulatory tab at the top of your screen
- Click on the Site Registration tab
- Enter your 5-character CTEP Institution Code and click on Go

Note: The status given only reflects compliance with IRB documentation and institutional compliance with protocol-specific requirements as outlined by the Lead Network. It does not reflect compliance with protocol requirements for individuals participating on the protocol or the enrolling investigator's status with the NCI or their affiliated networks.

4.3 Patient Registration

4.3.1 OPEN / IWRS

Patient enrollment will be facilitated using the Oncology Patient Enrollment Network (OPEN). OPEN is a web-based registration system available to users on a 24/7 basis. It is integrated with the CTSU Enterprise System for regulatory and roster data interchange and with the Theradex Interactive Web Response System (IWRS) for retrieval of patient registration/randomization assignment. Patient enrollment data entered by Registrars in OPEN / IWRS will automatically transfer to the NCI's clinical data management system, Medidata Rave.

For trials with slot reservation requirements, OPEN will connect to IWRS at enrollment initiation to check slot availability. Registration staff should ensure that a slot is available and secured for the patient before completing an enrollment.

The OPEN system will provide the site with a printable confirmation of registration and treatment information. Please print this confirmation for your records.

4.3.2 OPEN/IWRS User Requirements

OPEN/IWRS users must meet the following requirements:

- Have a valid CTEP-IAM account (*i.e.*, CTEP username and password).
- To enroll patients or request slot reservations: Be on an ETCTN Corresponding or Participating Organization roster with the role of Registrar. Registrars must hold a minimum of an AP registration type.
- To approve slot reservations or access cohort management: Be identified to Theradex as the "Client Admin" for the study.
- Have regulatory approval for the conduct of the study at their site.

Prior to accessing OPEN/IWRS, site staff should verify the following:

- All eligibility criteria have been met within the protocol stated timeframes.
- If applicable, all patients have signed an appropriate consent form and HIPAA authorization form.

4.3.3 OPEN/IWRS Questions?

Further instructional information on OPEN is provided on the OPEN tab of the CTSU website at https://www.ctsu.org or at https://open.ctsu.org. For any additional questions contact the CTSU Help Desk at 1-888-823-5923 or ctsucontact@westat.com.

Theradex has developed a Slot Reservations and Cohort Management User Guide, which is available on the Theradex website: http://www.theradex.com/clinicalTechnologies/?National-Cancer-Institute-NCI-11. This link to the Theradex website is also on the CTSU website OPEN tab. For questions about the use of IWRS for slot reservations, contact the Theradex Helpdesk at 609-619-7802 or Theradex main number 609-799-7580; CTMSSupport@theradex.com.

4.4 General Guidelines

Following registration, patients should begin protocol treatment within 5 days. Issues that would cause treatment delays should be discussed with the Principal Investigator. If a patient does not receive protocol therapy following registration, the patient's registration on the study may be canceled. The Study Coordinator should be notified of cancellations as soon as possible.

5. TREATMENT PLAN

5.1 Agent Administration

This is a Phase 1, open label study assessing the safety, tolerability, pharmacokinetic (PK) profiles, and pharmacodynamic (PD) profiles of ABT-888 in combination with CPT-11 (irinotecan). The study will consist of two portions: a dose-escalation portion and an expansion portion.

In the dose escalation portion, the optimal biologic dose (OBD) of the combination will be established, defined as the maximal decrease in PAR in tumor cells. The patient population to be studied will consist of subjects with advanced solid tumors, with preference to tumors of the breast, lung, pancreas, colon, or ovary.

In the expansion portion, patients will be treated at the recommended Phase II dose and evaluated for PD biomarkers. The patient population to be studied will consist of two cohorts: subjects with triple-negative BRCA-mutant breast cancer and patients with triple-negative, non-BRCA mutated cancer.

In the Dose Escalation for Intermittent ABT-888, patients will be treated using a dose escalation schedule. The patient population to be studied will consist of subjects with advanced solid tumors, with preference to tumors of the breast, lung, pancreas, colon, or ovary.

Treatment will be administered on an outpatient basis. Please see Appendix I for details of when ABT-888 is administered in the clinic vs. dispensed to the patient. Reported adverse events and potential risks for ABT-888 and irinotecan are described in Section 7. Appropriate dose modifications for ABT-888 and irinotecan are described in Section 6. No investigational or commercial agents or therapies other than those described below may be administered with the intent to treat the patient's malignancy.

Treatment Plan (Dose Escalation Portion):

This study will consist of cohorts of n=3 to 6 patients each. Treatment cycles will consist of 21 days.

Cycle 1: Irinotecan (CPT-11) will be administered by intravenous infusion at the indicated dose over 90 minutes on both Day 1 and Day 8 of Cycle 1. Single–dose PK sampling of irinotecan will be obtained up to 48h starting on Day 1. Twice daily oral administration of ABT-888 will begin two days following infusion of irinotecan therapy (starting Day 3), and will continue twice daily for a total of 12 days (through Day 14) followed by a 6 day rest. Please see Appendix I for details of when ABT-888 is administered in the clinic vs. dispensed to the patient. Tumor collection for PAR level evaluation and PD assays will occur at two times: 28 hours after chemotherapy alone (Day 2) and 28 hours after the combination of ABT-888 and chemotherapy (Day 9).

Cycle 2: Please see Appendix I for details of when ABT-888 is administered in the clinic vs. dispensed to the patient. ABT-888 will be administered one day prior (Day -1) to irinotecan therapy (half of normal total daily dose of ABT-888 to allow for PK sampling). A single–dose PK sampling of ABT-888 will be obtained up to 28 h starting on Day -1 of cycle 2. Subsequent doses of ABT-888 will be given twice-daily and continue daily through Day 14 of Cycle 2, followed by a 6 day rest. Irinotecan will be administered by intravenous infusion at the indicated dose over 90 minutes on days 1 and 8 of Cycle 2. The first dose of ABT-888 to be administered on Day 8 of Cycle 2 will be taken at the time of the start of irinotecan infusion, so PK sampling up to 48 h can be lined up for both drugs.

Subsequent cycles: Irinotecan will be administered by intravenous infusion at the indicated dose over 90 minutes on days 1 and 8. ABT-888 will be administered orally twice daily from Day -1 through Day 14, followed by a 6 day rest.

The planned dose levels are as follows in Table 2:

Table 2: Study Dose Escalation Schedule

**Dose Level**	**Dose***	
	**ABT-888 (mg po BID)**	**Irinotecan (CPT-11) (mg/m^2 IV)**
Level 1	10	100
Level 2	20	100
Level 3	40	100
Level 4	50	100
Level 5	80	100
Level 6	120	100

Doses are stated as exact dose in units (e.g., mg/m^2) rather than as a percentage. Note that ABT-888 doses are "flat" and **not calculated based on weight or BSA.*

However, the possibility exists that the planned doses of ABT-888 will be revised depending on the outcome of safety, PK, and/or PD data obtained from the ongoing CTEP phase 0 study of the drug. In addition, predicated on PD endpoints and toxicity or lack thereof, ABT-888 dosage may be increased by additional 50 mg increments beyond 120 mg po BID or aborted before escalating to the 120 mg po BID dose.

Dose escalation to the next dose level will occur when the preceding cohort reaches the end of Cycle 1. Patients who do not complete both treatment cycles for reasons other than an ABT-888-related toxicity will be replaced. Toxicity data for each cohort will be reviewed prior to dose escalation. Intra-patient dose escalation will be allowed for patients following their completion of Cycle 2 if the following criteria are met: the patient had no > grade 2 toxicities, the subsequent dose cohort has completed Cycle 1, and evaluation of DLTs has been completed for the subsequent dose cohort and the

dose was deemed to be safe due to ≤ grade 2 toxicities. Dose escalation will occur for both ABT-888 and irinotecan according to the dose escalation schema. Patients can be dose escalated multiple times as long as they continue to meet eligibility criteria and do not exceed the final protocol-specified dose level (dose Level 7). Additional PK samples will not be drawn for intra-patient dose escalated patients during Cycle 2.

The optimal biologic dose (OBD) is defined as the dose level at which no greater inhibition of PAR levels in tumor cells is identified, relative to the next lower dose. For each dose cohort, the first tumor biopsy will occur 28 hours after the start of chemotherapy alone (Day 2, Cycle 1). The second tumor biopsy will occur 28 hours after the start of the second chemotherapy infusion which is also 4 hours after the first Day 9, Cycle 1 dose of ABT-888. For each patient, we will determine the percentage change (expected to be a decrease) in tumor PAR level relative to his/her pre-ABT-888 treatment PAR level. Among each 3-6 patient dose cohort in the study, the median of their percentage changes in PAR will be determined. We define a biologically meaningful inhibition of PAR to be a median decrease of 50% in the pre/post ABT-888 treatment PAR level. The first time that those medians from 2 successive dose level cohorts are within 15% of each other will be taken as evidence that PAR inhibition has plateaued. The *lower* of those 2 successive dose levels shall be considered the OBD. If, for a given dose cohort, the median PAR inhibition is ≥ 90%, we shall consider that dose level as the OBD. Assessment of tumor PAR levels will occur after each dose level, with specimens submitted to the NCI Contracted Laboratory. Predicated on the results of the γ-H2AX and/or Rad51 foci data, these PD endpoints may be considered at a later date in the definition of OBD after discussion with CTEP.

Treatment Plan (Expansion Portion):

Treatment cycles will consist of 21 days.

Cycle 1: Irinotecan (CPT-11) will be administered by intravenous infusion at 100 mg/m^2 over 90 minutes on both Day 1 and Day 8 of Cycle 1. Twice daily oral administration of ABT-888 (40 mg po bid, 80 mg total daily dose) will begin 24 hours following infusion of irinotecan therapy (starting Day 2), and will continue twice daily for a total of 14 days (through Day 15) followed by a 7 day rest. Tumor collection for PD assays will occur at three times: baseline (within 2 weeks of treatment), 4-6 hours after the first dose of irinotecan (Day 1) and 4-6 hours after the combination of ABT-888 and irinotecan on Day 8.

Cycle 2 and Subsequent cycles: Irinotecan will be administered by intravenous infusion at 100 mg/m^2 over 90 minutes on days 1 and 8. ABT-888 will be administered orally twice daily (40 mg po bid, 80 mg total daily dose) from Day 1 through Day 15, followed by a 6 day rest. Decision to administrate ABT-888 on a continuous schedule will be predicated on results from first 6 patients following discussion with CTEP.

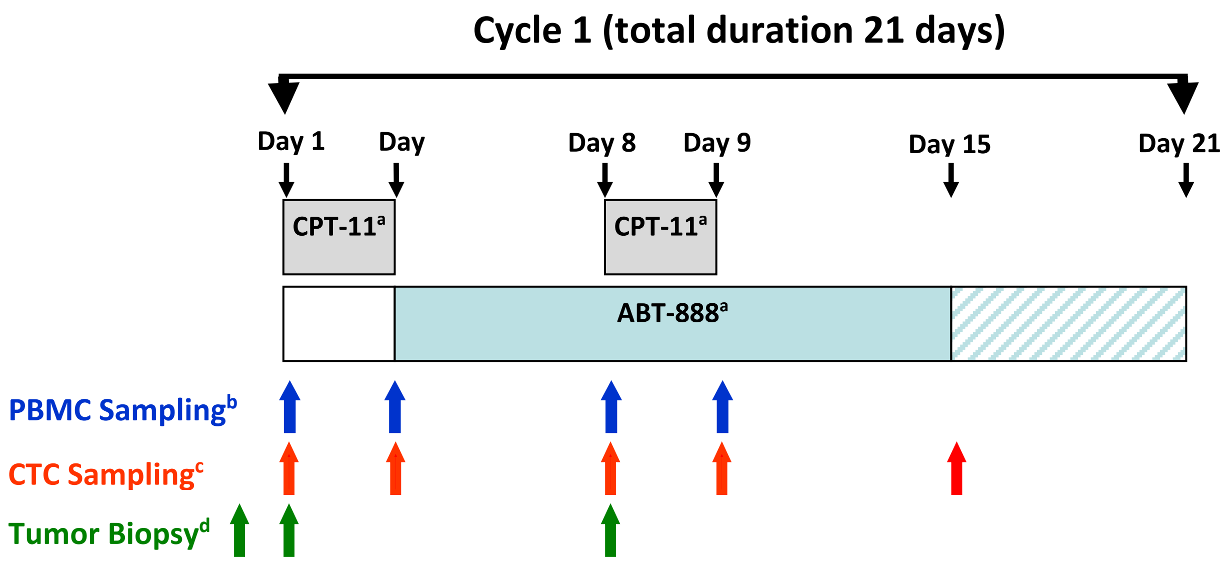

[a] CPT-11 (irinotecan) 100 mg/m^2 IV administered for 90 min on days 1 and 8 of each cycle. ABT-888 40 mg po bid on days 2-15 in cycle 1; Decision to administrate ABT-888 on a continuous schedule predicated on results from first 6 patients

[b] PBMC samples will be obtained at baseline, 4-6, 8, 22, and 24 h after CPT-11 alone on day 1 and after the combination dose on day 8 in cycle 1

[c] Circulating tumor cell (CTC) samples will be obtained at baseline, 4-6, 8, 22, and 24 h after CPT-11 alone on day 1 and after the combination dose on day 8 and once on day 15 of cycle 1

[d] Tumor biopsy will be performed at baseline, 4-6 hours after the first dose of CPT-11 (day 1) and the combination (day 8) in cycle 1.

The possibility exists that the planned schedule of administration of ABT-888 will be revised depending on the outcome of PD data obtained from the initial 6 patients placed on the expansion portion of the study. Predicated on gamma-H2AX levels evaluated in these patients, a continuous (21 day on) ABT-888 administration schedule will be considered as discussed in detail in Section 13 (statistical considerations).

Treatment Plan (Intermittent Dose Escalation Portion):
This study will consist of cohorts of n=3 to 6 patients each. Treatment cycles will consist of 21 days.

Run-In: ABT-888 will be administered orally twice daily on days -14, -13 and -12. ABT-888 will be administered orally once on day -11. Tumor collection will occur on day -11 within 4 hours of morning dose of ABT-888.

Cycle 1: Irinotecan will be administered by intravenous infusion at 100 mg/m^2 over 90 minutes on days 3 and 10. ABT-888 will be administered orally twice daily from Day 1 through Day 4 and Days 8-11. Tumor collection will occur within 4 hours of ABT-888 dose on Cycle 1 Day 11.

Cycle 2 and Subsequent cycles: Irinotecan will be administered by intravenous

infusion at 100 mg/m^2 over 90 minutes on days 3 and 10. ABT-888 will be administered orally twice daily from Day 1 through Day 4 and Days 8-11.

**Intermittent ABT-888 Dose Escalation Schedule**		
**Dose Level**	**Dose***	
	**<u>Run-In:</u>**<br>**ABT-888**<br>**(mg po BID)**<br>**Days -14, -13, -12**<br>**ABT-888**<br>**(mg po once)**<br>**Day -11**	**<u>Run-In:</u>**<br>**Irinotecan (CPT-11)**<br>**None**
	**<u>Cycles:</u> ABT-888**<br>**(mg po BID)**<br>**Days 1, 2, 3, & 4**<br>**Days 8, 9, 10 & 11**	**<u>Cycles:</u> Irinotecan**<br>**(CPT-11)**<br>**(mg/m^2 IV)**<br>**Days 3 & 10**
Level 1	50	100
Level 2	100	100
Level 3	150	100
Level 4	200	100
Level 5	250	100
Level 6	300	100
Intermediate dosing may be permitted if needed and only after discussion with CTEP		
**Doses are stated as exact dose in units (e.g., mg/m^2) rather than as a percentage. Note that ABT-888 doses are "flat" and **<u>not</u>** calculated based on weight or BSA.*		

5.1.1 ABT-888

Because there is a potential for interaction of ABT-888 with other concomitantly administered drugs, the case report form must capture the concurrent use of all other drugs, over-the-counter medications, or alternative therapies.

Potential Drug Interactions: Nonclinical studies suggest ABT-888 is a substrate of P-gp, OCT2, and MATE1/MATE2K transporters. Co-administration of ABT-888 with strong inhibitors of P-gp, OCT2, and MATE1/MATE2K drugs may result in a decrease of ABT-888 renal clearance and an increase in ABT-888 plasma concentration. Therefore, use caution when administering ABT-888 with strong inhibitors of P-gp, OCT2, and MATE1/MATE2K drugs. At high dose (e.g., 400 mg BID), ABT-888 may inhibit OCT1 in the liver and MATE1/MATE2K in the kidney.

ABT-888 is not a potent inhibitor of the major human CYPs and does not significantly induce activities of major human CYP isoforms, suggesting a

negligible potential for CYP-mediated drug-drug interactions as a perpetrator at the anticipated therapeutic concentrations.

In human, ABT-888 clears primarily in the urine as intact parent drug along with metabolites suggesting that renal function plays an important role in the drug clearance and its metabolites. Drug-associated with kidney toxicities or kidney diseases could change ABT-888 pharmacokinetics. Use cautions when concomitantly administer oxalipaltin, carboplatin, cisplatin, and topotecan in patients with pre-existing renal impairment.

Patient Care Implications: Patients may feel fatigue or tiredness. Loss of appetite and losing weight are common. Provide appropriate supportive care for diarrhea.

Original Dose Escalation Portion:

Cycle 1: ABT-888 treatment will begin on day 3 of cycle 1, and will start with twice daily dose and continue twice daily through day 14 (12 days of ABT-888 administration total).

Cycle 2: ABT-888 will be administered orally twice daily ***except*** for Day -1 of Cycle 2. A single-dose of ABT-888 will be given ***ONCE*** on day -1 of cycle 2 to get a full PK profile up to 28 h. This single dose will be half of the normal total daily dose. Subsequent doses of ABT-888 in Cycle 2 will be given twice-daily and continue daily through Day 14 of the cycle, followed by a 6 day rest (15 days of ABT-888 administration total: one day at half dose and 14 days at full twice-daily dose).

Subsequent cycles: ABT-888 will be administered orally twice daily from Day -1 of each cycle through day 14 (15 days total).

A toxicity and ABT-888 drug accountability diary (Appendix H) will be given to the participant by research staff at the beginning of each cycle in order to assess their compliance and determine the need for technique/treatment alterations.

Expansion Portion:

Cycle 1: ABT-888 treatment will begin on day 2 of cycle 1, and will start with twice daily dose and continue twice daily through day 15 (12 days of ABT-888 administration total) followed by 6 days rest.

Cycle 2 and subsequent cycles: ABT-888 will be administered orally twice daily through Day 15 of the cycle, followed by a 6 day rest (15 days of ABT-888 administration total).

A toxicity and ABT-888 drug accountability diary (Appendix H) will be given to the participant by research staff at the beginning of each cycle in order to assess their compliance and determine the need for technique/treatment alterations.

Dose Escalation for Intermittent ABT-888 Portion:

Cycles: ABT-888 will be administered orally twice daily from Day 1 through Day 4 and Days 8-11.

A toxicity and ABT-888 drug accountability diary (Appendix H) will be given to the participant by research staff at the beginning of each cycle in order to assess their compliance and determine the need for technique/treatment alterations.

Missed or vomited doses: Missed or vomited doses of ABT-888 will not be replaced.

5.1.2 Irinotecan (CPT-11)

5.1.2.1 Pre-treatment:

Adequate anti-emetic therapy and prophylactic loperamide (not to exceed 16 mg/day) must be provided. Premedication with 10mg dexamethasone in conjunction with another type of antiemetic agent (e.g. 5HT3 receptor antagonist) should be administered 30 minutes prior to irinotecan therapy.

5.1.2.2 Treatment with Irinotecan (Original Dose Escalation portion and Expansion portion):

On Days 1 and 8 of each 21-day cycle, irinotecan will be administered at the indicated dose by infusion over 90 minutes.

5.1.2.3 Treatment with Irinotecan (Dose Escalation for Intermittent ABT-888 portion)

On Days 3 and 10 of each 21-day cycle, irinotecan will be administered at the indicated dose by infusion over 90 minutes.

5.2 Definition of Dose-Limiting Toxicity and Recommended Phase II Dose

However, if DLTs occur, they will be defined based on toxicities observed following the first dose of ABT-888 until the end of Cycle 1. Toxicities will be graded using the descriptions and grading scales found in the revised NCI Common Terminology Criteria for Adverse Events (CTCAE) version 3.0 until July 31, 2010. CTCAE version 4.0 will be utilized from August 1, 2010 until March 31, 2018. CTCAE version 5.0 will be utilized for AE reporting beginning April 1, 2018. All appropriate treatment areas should have access to a copy of the CTCAE version 5.0. A copy of the CTCAE version 5.0 can be downloaded from the CTEP web site http://ctep.cancer.gov/protocolDevelopment/electronic_applications/ctc.htm .

DLTs include:

- non-hematologic toxicity Grade ≥ 3 (excluding untreated nausea or vomiting, or alopecia),
- nausea, vomiting, or diarrhea uncontrolled by aggressive treatment ≥ Grade 3
- grade 4 granulocytopenia lasting ≥ 5 days without hematopoeitic growth factor support; or febrile neutropenia of any duration
- thrombocytopenia (platelet count ≤ 25,000 cells/mm^3)

- inability to receive the subsequent planned dose of irinotecan and/or ABT-888 due to drug-related toxicity according to the above DLT criteria and after discussion with the PI and study team
- inability to begin Cycle 2 of treatment (at full dose) within 2 weeks of the scheduled dose due to unresolved toxicity
- Grade ≥ 2 non-hematological toxicity that persists beyond the first 42 days that in the judgment of the Principal Investigator is dose-limiting
- Certain Grade 2 toxicity (e.g. renal neurological, cardiovascular, gastrointestinal) which in the judgment of the Principal Investigator is dose-limiting
- If there is a ≥ grade 3 electrolyte imbalance which is secondary to another toxicity and not independent, then the toxicity leading to the electrolyte imbalance will be the toxicity used to grade for the DLT definition (e.g. in the case of hypophosphatemia or hypokalemia as a result of diarrhea , diarrhea will be graded for DLT definition).

  In addition, if electrolyte imbalance is used for a DLT definition, the electrolyte imbalance should be graded after a 24-hour replacement window (e.g. if hypokalemia is low at a grade 3 or 4, attempt replacement for 24 hours. If, after 24 hours, toxicity is not improved to ≤ grade 2, then the toxicity will be considered a DLT.)

Management and dose modifications associated with the above adverse events are outlined in Section 6. Dose escalation will proceed within each cohort according to the following scheme. Dose-limiting toxicity (DLT) is defined above.

Table 3: Dose Escalation Rules for Original Escalation Portion and Dose Escalation for Intermittent ABT-888 Portion

Number of Patients with DLT at a Given Dose Level	Escalation Decision Rule
0 out of 3	Enter 3 patients at the next dose level.
≥2	Dose escalation will be stopped. This dose level will be declared the maximally administered dose (highest dose administered). Three (3) additional patients will be entered at the next lowest dose level if only 3 patients were treated previously at that dose.
1 out of 3	Enter at least 3 more patients at this dose level.<br>• If 0 of these 3 patients experience DLT, proceed to the next dose level.<br>• If 1 or more of this group suffer DLT, then dose escalation is stopped, and this dose is declared the maximally administered dose. Three (3) additional patients will be entered at the next lowest dose level if only 3 patients were treated previously at that dose.
≤1 out of 6 at highest dose level below the maximally administered dose	This is generally the recommended phase 2 dose (RP2D). At least 6 patients must be entered at the recommended phase 2 dose.

If one patient develops a DLT, then the cohort will be expanded to include a total of 6 patients. If greater than or equal to two patients develop DLT, then that dose level will be considered the maximum administered dose (MAD) and further dose escalation will be terminated. A total of 6 patients will be treated at the next lower dose. The maximum tolerated dose (MTD) will be the dose at which no more than 1 patient develops DLT when at least 6 patients have been treated.

For the original dose escalation portion: If an effect on biological PAR levels is observed in patients treated at a specific dose, but no DLT is observed, dose escalation will continue as planned in order to demonstrate whether an increase in biological effect can be observed with increasing dosage or if such effect is maximized (ie, the OBD is attained). If the OBD has not been documented by the end of the planned dose escalation schedule, and the MTD of the combination has also not been reached, additional cohorts may be treated until maximal PAR inhibition or toxicity is documented. If the OBD has been achieved without evidence of DLTs, the OBD cohort will be expanded to 10 patients to assure safety.

Assessment of tumor response will be performed during the final week of Cycle 2. Patients with an objective response or stable disease may receive additional treatment cycles until disease progression or withdrawal criteria are met. Ongoing evaluation of response will be conducted after every other treatment cycle, and safety will be evaluated weekly.

For the original dose escalation portion: Throughout the escalation process, two biopsies will be obtained for each patient for the purpose of PAR level evaluation and PD assays: 28 hours after irinotecan chemotherapy alone (Day 2) and 28 hours after the combination of ABT-888 and irinotecan chemotherapy (Day 9). .It will be a requirement to obtain biopsies for determination of additional PD endpoints as discussed below.

Recommended Phase II Dose (RP2D) Original Dose Escalation Portion:
In the event that DLTs are observed prior to achieving the OBD, the recommended phase II dose (RP2D) will be the dose where ≤1 DLT is observed out of 6 patients treated at the highest dose level below the maximally administered dose (i.e. the MTD). However, since we do not expect ABT-888-related toxicity, the RP2D may instead be described as the OBD (dose which gives a maximal decrease in PAR levels) if DLTs are not observed prior to reaching the OBD. The RP2D is hereby defined as the dose that, based on all data compiled in this Phase 1 trial (including feasibility, safety, dose limiting toxicities, maximally tolerated dose, and levels of PARP inhibition), is determined to be taken forward into Phase II study.

Recommended Phase II Dose (RP2D) Dose Escalation for Intermittent ABT-888 Portion: The recommended phase II dose (RP2D) will be the dose where ≤1 DLT is observed out of 6 patients treated at the highest dose level below the maximally administered dose (i.e. the MTD).

5.3 General Concomitant Medication and Supportive Care Guidelines

5.3.1 ABT-888

5.3.1.1 Supportive care

In case participants develop nausea/vomiting/diarrhea, supportive medications will be prescribed as per Clinical Center and ASCO guidelines. Seizures were seen in some animal toxicology studies, although at doses much higher than those anticipated for this study. Seizures in animals were successfully treated with lorazepam. Standard-of-care measures will be used for seizures, should they be encountered.

ABT-888 is not known to be a potent inhibitor of the major human CYPs in vitro, indicating a low risk for drug-drug interactions at the proposed dosing concentrations.

5.3.1.2 QTc prolongation

In order to monitor potential cardiac effects of ABT-888, EKGs will be evaluated at baseline (prior to treatment) and as needed based on the judgment of the treating oncologist/principal investigator.

5.3.1.3 Myelosuppression

Patients should be monitored for persistent myelosuppression, including anemia, thrombocytopenia, and neutropenia that does not recover to normal or grade 1 between courses of treatment, as per Good Clinical Practice. If peripheral blood counts do not recover to normal or are persistently abnormal, the patient should be evaluated for the possible development of AML/MDS using a bone marrow aspirate with cytogenetics. ABT-888 therapy should be held and discontinued if AML/MDS is documented. A complete history of prior therapy should be documented, particularly prior platinum-based or alkylating agent therapies. Documentation of germline BRCA mutation (*gBRCA*m) status, if known, should be recorded.
See section 6.0 for management of ABT-888 hematologic toxicity.

5.3.2 Irinotecan

5.3.2.1 Supportive Care

The dose-limiting toxicities of irinotecan are diarrhea and neutropenia.

The most common and severe adverse effect of irinotecan is ***diarrhea***. Two distinct types of diarrhea associated with irinotecan have been identified – an early onset cholinergic syndrome and late-onset diarrhea. The early-onset cholinergic effects may arise up to 24 hours after treatment and includes profound warmth, diaphoresis and flushing followed by abdominal cramping and sudden diarrhea. Other symptoms may occur, including salivation, lacrimation, nasal congestion, or rhinorrhea. Acute events are managed successfully by administering IV or SC atropine 0.25 to 1.0 mg.

Because of the short half-life of atropine, using it to prevent cholinergic symptoms is controversial. Late diarrhea (occurring more than 24 hours after administration) may lead to dehydration or electrolyte imbalances, and can be life threatening. The mechanism of late onset diarrhea is not well understood, but it appears to be linked to a secretory process that may be a secondary consequence of an irinotecan cytotoxic effect on the GI mucosa. The median onset time is 11 days. It must be treated promptly with loperamide, 4 mg at the first onset of late diarrhea and then 2mg every 2 hours until the patient is diarrhea-free for at least 12 hours.

During the night the patient may take 4mg of loperamide every 4 hours. Fluid intake should be maintained to avoid dehydration. Premedication with loperamide is not recommended and laxatives should be avoided. The recommended maximum daily dose of loperamide is not to exceed 16 mg/day.

Irinotecan induced ***neutropenia*** is dose-related, generally brief, and noncumulative, with a typical onset between days 15 and 21 and recovery between days 28 and 35. The frequency of grade 3 or 4 neutropenia is higher in patients who had prior pelvic or abdominal irradiation, have elevated serum bilirubin or who receive the drug over less than 90 minutes.

Pneumonitis has been reported infrequently (predominantly in studies from Japan) following administration of irinotecan. This has been described as dyspnea, a non-productive cough, or a diffuse pulmonary infiltrate on chest x-ray. The etiology of these problems is unknown, and it is not clear whether they truly are caused by irinotecan or are actually a manifestation of the disease, primary lung cancer, or lung metastases. Japanese studies have also suggested that patients with large third space fluid collections may be at an increased risk of developing toxicity.

5.3.2.2 Potential Interactions

Because there is a potential for interaction of irinotecan with other concomitantly administered drugs through the cytochrome P450 system, the case report form must capture the concurrent use of all other drugs, over-the-counter medications, or alternative therapies. The Principal Investigator should be alerted if the patient is taking any agent known to affect or with the potential to affect selected CYP450 isoenzymes.

General guidelines for known drug-drug interactions follow:

- Anticonvulsants: Exposure to irinotecan and its active metabolite SN-38 is substantially reduced in adult and pediatric patients concomitantly receiving the CYP3A4 enzyme-inducing anticonvulsants phenytoin, phenobarbital or carbamazepine. The

appropriate starting dose for patients taking these anticonvulsants has not been formally defined. The following drugs are also CYP3A4 inducers: rifampin, rifabutin. For patients requiring anticonvulsant treatment, consideration should be given to substituting non-enzyme inducing anticonvulsants at least 2 weeks prior to initiation of irinotecan therapy. Should any participants develop seizures while on study, non-enzyme inducing anticonvulsants should be used.

- Dexamethasone does not appear to alter the pharmacokinetics of irinotecan.

- St. John's Wort: St. John's Wort is an inducer of CYP3A4 enzymes. Exposure to the active metabolite SN-38 is reduced in patients receiving concomitant St. John's Wort. St. John's Wort should be discontinued at least 2 weeks prior to the first cycle of irinotecan, and St. John's Wort is contraindicated during irinotecan therapy.

- Ketoconazole: Ketoconazole is a strong inhibitor of CYP3A4 enzymes. Patients receiving concomitant ketoconazole have increased exposure to irinotecan and its active metabolite SN-38. Patients should discontinue ketoconazole at least 1 week prior to starting irinotecan therapy and ketoconazole is contraindicated during irinotecan therapy.

- Neuromuscular blocking agents. Interaction between irinotecan and neuromuscular blocking agents cannot be ruled out. Irinotecan has anticholinesterase activity, which may prolong the neuromuscular blocking effects of suxamethonium and the neuromuscular blockade of non-depolarizing drugs may be antagonized.

- Atazanavir sulfate: Coadministration of atazanavir sulfate, a CYP3A4 and UGT1A1 inhibitor has the potential to increase systemic exposure to SN-38, the active metabolite of irinotecan. Physicians should take this into consideration when co-administering these drugs.

5.4 Duration of Therapy

In the absence of significant treatment delays due to drug-related adverse events, treatment may continue indefinitely or until one of the following criteria applies:

- Disease progression,
- Intercurrent illness that prevents further administration of treatment,
- Unacceptable adverse event(s),
- Patient decides to withdraw from the study, or
- General or specific changes in the patient's condition render the patient unacceptable for further treatment in the judgment of the investigator.

- Bone marrow findings consistent with acute myeloid leukemia (AML)/MDS
- Severe persistent anemia requiring transfusion to maintain ≥ 10 g/dL hemoglobin (Hb)

5.5 Duration of Follow Up

Patients will be followed for 30 days after removal from study or until death, whichever occurs first. Patients removed from study for unacceptable adverse events will be followed until resolution or stabilization of the adverse event.

5.6 Criteria for Removal from Study

Patients will be removed from study when any of the criteria listed in Section 5.4 applies. The reason for study removal and the date the patient was removed must be documented in the Case Report Form.

6. DOSING DELAYS/DOSE MODIFICATIONS

- The descriptions and grading scales found in the revised NCI Common Terminology Criteria for Adverse Events (CTCAE) version 3.0 will be utilized until July 31, 2010 for AE reporting. CTCAE version 4.0 will be utilized from August 1, 2010 until March 31, 2018. CTCAE version 5.0 will be utilized for AE reporting beginning April 1, 2018. All appropriate treatment areas should have access to a copy of the CTCAE version 5.0. A copy of the CTCAE version 5.0 can be downloaded from the CTEP web site http://ctep.cancer.gov/protocolDevelopment/electronic_applications/ctc.htm.
- Doses of irinotecan (CPT-11) and ABT-888 will be administered at the same dose level throughout a cycle if no DLTs or dose-reducing toxicities occur (see tables below).
- Inability to receive the subsequent planned daily dose during cycle 1 due to drug-related toxicity may be considered a DLT if agreed upon by the Principal Investigator and study team.
- If a DLT occurs, all subsequent ABT-888 and irinotecan (CPT-11) administration will be held for the remainder of the cycle until the toxicity resolves to ≤ grade 1 or baseline. Please refer to tables below for subsequent cycle treatment instructions.
- If a DLT is observed at any time during a treatment cycle, dose modification of either or both ABT-888 and irinotecan (CPT-11) may occur after discussion by the Principal Investigator and study team.
- If a toxicity is < Grade 3, the patient can continue the daily dosing of ABT-888 at the same dose level if the principal investigator deems it safe to proceed after evaluation of the toxicity.
- When DLT occurs during Cycle 1, patients may continue treatment during subsequent cycles at the next lower dose levels as specified above. In this case, pharmacokinetic sampling will be performed during the patient's first cycle at the next lowest dose level.

Dose escalation and de-escalation of ABT-888 is discussed in Section 5.2. If there is a > 2 week delay of reinstitution of ABT-888 due to ABT-888 related toxicity, then the patient will come off study. If there is a ≤ 2 week delay due to recovery of toxicity or if there is a report of grade 3 or grade 4 toxicity thought to be due to ABT-888 therapy, then the patient will be re-treated at the next lower cohort.

ABT-888 Hematologic Toxicity Management

Management of neutropenia and thrombocytopenia

Neutropenia and thrombocytopenia are recognized common adverse drug reactions reported for ABT-888. ABT-888 treatment should be managed according to Table 4:

Table 4: ABT-888 management of neutropenia and thrombocytopenia

CTCAE Grade	Definition	ABT-888 Dose
1-2	ANC >1.0 G/L or Platelet count >50 G/L	Investigator judgement to continue treatment or allow dose interruption; dose interruptions should be for a maximum of 2 weeks; appropriate supportive treatment and causality investigation.
3-4	ANC <1.0 G/L or Platelet count <50 G/L	Dose interruption until recovered to CTCAE Grade ≤1 for a maximum of 2 weeks. Upon recovery, ABT-888 dose should be reduced by one dose level.

ANC = absolute neutrophil count; CTCAE = Common Terminology Criteria for Adverse Events

Use of hematopoietic agents

Use erythropoietin-stimulating agents per standard of care National Comprehensive Cancer Network (NCCN) and/or institutional guidelines, iron supplements, and/or transfusions as clinically indicated for management of anemia. Prescribing information for the erythropoiesis stimulating agents (including Aranesp, Epogen and Procrit) highlight that there is a potential risk of shortening the time to tumor progression or disease-free survival. Primary prophylaxis with granulocyte colony-stimulating factor (G-CSF) is not recommended. Aranesp, Epogen and Procrit may not alleviate fatigue or increase energy, and should not be used in patients with uncontrolled hypertension. The package inserts for these agents should be consulted.

If a patient develops febrile neutropenia, ABT-888 should be stopped and appropriate management including G-CSF should be given according to local hospital guidelines. Please note that G-CSF should not be used within at least 24 hours of the last dose of ABT-888 unless absolutely necessary.

Platelet transfusions, if indicated, should be done according to local hospital guidelines.

Dose modifications for hematologic toxicity

Patients who have ABT-888 held for hematologic toxicities should have blood counts and differentials checked at least weekly until recovery; these data should be recorded in eCRF as extra laboratory examinations. If there is a > 2 week delay of reinstitution of ABT-888 due to ABT-888 related toxicity, then the patient will come off study. The patient must continue to be followed and if counts do not improve to CTCAE Grade 1 or better within an additional week (total of 3 weeks off of ABT-888), patients should be referred to a hematological oncologist for further assessment. A bone marrow analysis should be considered.

Management of anemia

Anemia is a common adverse drug reaction related to ABT-888. ABT-888 management of anemia is in accordance with Table 5:

Table 5: ABT-888 management of anemia

CTCAE Grade	Definition	ABT-888 Dose
2	Hb <10 but ≥8 g/dL	Give appropriate supportive treatment and investigate causality. Investigator judgement to continue ABT-888 or interrupt dose for a maximum of 2 weeks. If repeat Hb <10 but ≥8 g/dL, dose interrupt until Hb ≥10 g/dL for maximum of 2 weeks and upon recovery dose reduce by one dose level.
3	Hb <8 g/dL	Give appropriate supportive treatment and investigate causality. Interrupt ABT-888 until improved to Hb ≥10 g/dL. Upon recovery dose reduce ABT-888 by one dose level.

Hb = hemoglobin

Common treatable causes of anemia (e.g., iron, vitamin B12 or folate deficiencies and hypothyroidism) should be investigated and appropriately managed. In some cases management of anemia may require blood transfusions. Any subsequently required dose interruptions related to development of anemia, or coexistent with newly developed neutropenia, and/or thrombocytopenia, will require ABT-888 dose reduction by one dose level.

If Hb drops to <8 g/dL despite the dose reduction or more than one blood transfusion is required to recover Hb levels with no alternative explanation for the anemia, ABT-888 should be permanently discontinued.

Management of prolonged hematological toxicities while on study treatment

If a patient develops prolonged hematological toxicity such as:

- ≥ 2 week interruption/delay in ABT-888 (veliparib) due to CTCAE Grade ≥ 3 anemia (Hb <8 g/dL) and/or development of blood transfusion dependence
- ≥ 2 week interruption/delay in ABT-888 due to CTCAE Grade ≥ 3 neutropenia (ANC <1 x 10^9/L)
- ≥ 2 week interruption/delay in ABT-888 due to CTCAE Grade ≥ thrombocytopenia and/or development of platelet transfusion dependence (Platelets < 50 x 10^9/L)

If there is a > 2 week delay of reinstitution of ABT-888 due to ABT-888 related toxicity, then the patient will come off study. Check weekly differential blood counts including reticulocytes and peripheral blood smear. If any blood parameters remain clinically abnormal after 3

weeks off of ABT-888, the patient should be referred to a hematological oncologist for further investigations. Bone marrow for evaluation and cytogenetics should be considered at this stage according to standard hematological oncology practice.

Management of ABT-888 associated toxicity

Management of MDS/AML

Patients who develop MDS/AML on treatment should discontinue ABT-888 treatment and be managed appropriately.

The dose regimen of irinotecan (CPT-11) is as follows:

Table 6: irinotecan (CPT-11) dose regimen for Original Dose Escalation and Expansion Portions

**Dose Modifications of Irinotecan (CPT-11)**			
**Weekly Regimen**	100 mg/m² IV over 90 min, d 1& 8 then 1-wk rest		
(Dose Levels 1-6)	**Starting Dose & Modified Dose Levels[c] (mg/m²)**		
	Starting Dose	Dose Level -1	Dose Level -2
	100	75	50

Table 6.1 irinotecan (CPT-11) dose regimen for Dose Escalation for Intermittent ABT-888 Portion

**Dose Modifications of Irinotecan (CPT-11)**			
**Weekly Regimen**	Administered IV over 90 min, d 3 & 10 then 1-wk rest		
	**Starting Dose & Modified Dose Levels[c] (mg/m²)**		
	Starting Dose	Dose Level -1	Dose Level -2
Dose Level 1-6	100	75	50

Regimens of irinotecan (CPT-11) should be adjusted as detailed in the table below: **Recommended Dose Modifications for Irinotecan (CPT-11)**. All dose modifications should be based on the worst preceding toxicity.

A new cycle of therapy should not begin until the toxicity has recovered to grade 1 or less. Treatment may be delayed 1 to 2 weeks to allow for recovery from treatment-related toxicity. If the patient has not recovered after 2 weeks, the patient will discontinue the combination therapy and be removed from the study. Provided intolerable toxicity does not develop, treatment with additional cycles of irinotecan+ABT-888 may be continued indefinitely as long as patients continue to experience clinical benefit. Dose reductions may occur multiple times; as long as the participant continues to respond to therapy, reductions may occur down to the lowest protocol-specified dose level (dose Level 1). Dose re-escalation of either drug will not be allowed. Doses of irinotecan held due to toxicity will not be made up.

Table 7: Recommended Dose Modifications For Irinotecan (CPT-11)[a]

A new cycle of therapy should not begin until the granulocyte count has recovered to ≥1500/mm^3, and the platelet count has recovered to ≥100,000/mm^3, and treatment-related diarrhea is fully resolved. Treatment should be delayed 1 to 2 weeks to allow for recovery from treatment-related toxicities. If the patient has not recovered after a 2-week delay, consideration should be given to discontinuing irinotecan (CPT-11). If any dose of irinotecan is held during a cycle, all subsequent doses of ABT-888 may continue for the rest of that same cycle.

Worst Toxicity NCI Grade[b] (Value)	During a Cycle of Therapy	At the Start of the Next Cycles of Therapy (After Adequate Recovery), Compared with the Starting Dose in the Previous Cycle[a,e]
**No toxicity**	Maintain dose level	Maintain dose level
**Neutrophils (ANC)**		
1 (1500 to 1999/mm^3)	Maintain dose level	Maintain dose level
2 (1000 to 1499/mm^3)	↓ 25 mg/m^2	Maintain dose level
3 (500 to 999/mm^3)	Omit dose until resolved to ≤ grade 2, then ↓ 25 mg/m^2	↓ 25 mg/m^2
4 (<500/mm^3)	Omit dose until resolved to ≤ grade 2, then ↓ 50 mg/m^2	↓ 50 mg/m^2
**Febrile Neutropenia**	Omit dose until resolved, then ↓ 50 mg/m^2 when resolved	↓ 50 mg/m^2
**Other hematologic toxicities**	Dose modifications for leukocytes (total WBC), and platelets during a cycle of therapy and at the start of subsequent cycles of therapy are also based on NCI toxicity criteria and are the same as recommended for neutropenia above.	
**Diarrhea**		
1 (1-3 stools/day > pretx[c])	Maintain dose level	Maintain dose level
2 (4-6 stools/day > pretx)	↓ 25 mg/m^2	Maintain dose level
3 (≥ 7 stools/day > pretx)	Omit dose until resolved to ≤ grade 2, then ↓ 25 mg/m^2	↓ 25 mg/m^2
4 (life-threatening)	Omit dose until resolved to ≤ grade 2 then ↓ 50 mg/m^2	↓ 50 mg/m^2
**Other nonhematologic[d] toxicities**		
1	Maintain dose level	Maintain dose level
2	↓ 25 mg/m2	↓ 25 mg/m^2
3	Omit dose until resolved to ≤ grade 2, then ↓ 25 mg/m^2	↓ 25 mg/m^2
4	Omit dose until resolved to ≤ grade 2, then ↓ 50 mg/m^2	↓ 50 mg/m^2

[a] All dose modifications should be based on the worst preceding toxicity
[b] National Cancer Institute Common Terminology Criteria for Adverse Events
[c] Pretreatment
[d] Excludes alopecia, anorexia, asthenia
[e] Dose modification other than that provided here may occur following discussion with the study PI and the study team

7. ADVERSE EVENTS: LIST AND REPORTING REQUIREMENTS

Adverse event (AE) monitoring and reporting is a routine part of every clinical trial. The following list of AEs (Section 7.1) and the characteristics of an observed AE (Section 7.2) will determine whether the event requires expedited (via CTEP-AERS) **in addition** to routine reporting.

7.1 Comprehensive Adverse Events and Potential Risks Lists (CAEPRs)

7.1.1 CAEPR for ABT-888 (Veliparib, NSC 737664)

Comprehensive Adverse Events and Potential Risks list (CAEPR)

for
ABT-888 (Veliparib, NSC 737664)

The Comprehensive Adverse Events and Potential Risks list (CAEPR) provides a single list of reported and/or potential adverse events (AE) associated with an agent using a uniform presentation of events by body system. In addition to the comprehensive list, a subset, the Specific Protocol Exceptions to Expedited Reporting (SPEER), appears in a separate column and is identified with bold and italicized text. This subset of AEs (SPEER) is a list of events that are protocol specific exceptions to expedited reporting to NCI (except as noted below). Refer to the 'CTEP, NCI Guidelines: Adverse Event Reporting Requirements' http://ctep.cancer.gov/protocolDevelopment/electronic_applications/docs/aeguidelines.pdf for further clarification. *Frequency is provided based on 2310 patients.* Below is the CAEPR for ABT-888 (Veliparib).

> **NOTE**: Report AEs on the SPEER **ONLY IF** they exceed the grade noted in parentheses next to the AE in the SPEER. If this CAEPR is part of a combination protocol using multiple investigational agents and has an AE listed on different SPEERs, use the lower of the grades to determine if expedited reporting is required.

Version 2.4, May 13, 2018[1]

**Adverse Events with Possible Relationship to ABT-888 (Veliparib) (CTCAE 5.0 Term) [n= 2310]**			**Specific Protocol Exceptions to Expedited Reporting (SPEER)**
**Likely (>20%)**	**Less Likely (<=20%)**	**Rare but Serious (<3%)**	
BLOOD AND LYMPHATIC SYSTEM DISORDERS			
	Anemia		***Anemia (Gr 3)***
	Febrile neutropenia		***Febrile neutropenia (Gr 3)***
GASTROINTESTINAL DISORDERS			
	Abdominal pain		
	Constipation		***Constipation (Gr 2)***
	Diarrhea		***Diarrhea (Gr 3)***
Nausea			***Nausea (Gr 3)***
	Vomiting		***Vomiting (Gr 3)***
GENERAL DISORDERS AND ADMINISTRATION SITE CONDITIONS			
Fatigue			***Fatigue (Gr 3)***
INVESTIGATIONS			
	Lymphocyte count decreased		***Lymphocyte count decreased (Gr 4)***
	Neutrophil count decreased		***Neutrophil count decreased (Gr 4)***
Platelet count decreased			***Platelet count decreased (Gr 4)***
	Weight loss		***Weight loss (Gr 2)***
	White blood cell decreased		***White blood cell decreased (Gr 4)***
METABOLISM AND NUTRITION DISORDERS			
	Anorexia		***Anorexia (Gr 2)***
	Dehydration		***Dehydration (Gr 3)***
	Hypophosphatemia		***Hypophosphatemia (Gr 3)***
NEOPLASMS BENIGN, MALIGNANT AND UNSPECIFIED (INCL CYSTS AND POLYPS)			
		Leukemia secondary to oncology chemotherapy	
		Myelodysplastic syndrome	

Adverse Events with Possible Relationship to ABT-888 (Veliparib) (CTCAE 5.0 Term) [n= 2310]			Specific Protocol Exceptions to Expedited Reporting (SPEER)
**Likely (>20%)**	**Less Likely (<=20%)**	**Rare but Serious (<3%)**	
		Treatment related secondary malignancy	
NERVOUS SYSTEM DISORDERS			
	Dizziness		
	Dysgeusia		***Dysgeusia (Gr 2)***
	Headache		***Headache (Gr 3)***
		Seizure	
SKIN AND SUBCUTANEOUS TISSUE DISORDERS			
	Rash maculo-papular		
VASCULAR DISORDERS			
		Thromboembolic event[2]	

[1]This table will be updated as the toxicity profile of the agent is revised. Updates will be distributed to all Principal Investigators at the time of revision. The current version can be obtained by contacting PIO@CTEP.NCI.NIH.GOV. Your name, the name of the investigator, the protocol and the agent should be included in the e-mail.

[2]Thromboembolic events, including deep vein thrombosis and pulmonary embolism, have been observed at a higher frequency compared to control arm when administered in combination with temozolomide.

Adverse events reported on ABT-888 (Veliparib) trials, but for which there is insufficient evidence to suggest that there was a reasonable possibility that ABT-888 (Veliparib) caused the adverse event:

BLOOD AND LYMPHATIC SYSTEM DISORDERS - Bone marrow hypocellular; Blood and lymphatic system disorders - Other (pancytopenia)
CARDIAC DISORDERS - Cardiac disorders - Other (Takotsubo cardiomyopathy); Heart failure; Left ventricular systolic dysfunction; Palpitations; Sinus bradycardia; Sinus tachycardia
EAR AND LABYRINTH DISORDERS - Vertigo
EYE DISORDERS - Blurred vision
GASTROINTESTINAL DISORDERS - Abdominal distension; Ascites; Colitis; Colonic obstruction; Dental caries; Dry mouth; Duodenal ulcer; Dyspepsia; Dysphagia; Enterocolitis; Esophagitis; Flatulence; Gastritis; Gastroesophageal reflux disease; Lower gastrointestinal hemorrhage; Mucositis oral; Obstruction gastric; Rectal hemorrhage; Rectal pain; Small intestinal obstruction
GENERAL DISORDERS AND ADMINISTRATION SITE CONDITIONS - Chills; Edema limbs; Fever; Flu like symptoms; Malaise; Non-cardiac chest pain; Pain
HEPATOBILIARY DISORDERS - Hepatic failure; Hepatobiliary disorders - Other (cirrhosis)
INFECTIONS AND INFESTATIONS - Appendicitis; Catheter related infection; Infections and infestations - Other (peritonsillar abscess); Lung infection; Lymph gland infection; Mucosal infection; Sepsis; Shingles; Skin infection; Upper respiratory infection; Urinary tract infection
INJURY, POISONING AND PROCEDURAL COMPLICATIONS - Bruising; Dermatitis radiation; Radiation recall reaction (dermatologic)
INVESTIGATIONS - Alanine aminotransferase increased; Alkaline phosphatase increased; Aspartate aminotransferase increased; Blood bilirubin increased; Cardiac troponin I increased; Creatinine increased; Electrocardiogram QT corrected interval prolonged; Lipase increased

METABOLISM AND NUTRITION DISORDERS - Hyperglycemia; Hypernatremia; Hypoalbuminemia; Hypocalcemia; Hypokalemia; Hypomagnesemia; Hyponatremia
MUSCULOSKELETAL AND CONNECTIVE TISSUE DISORDERS - Arthralgia; Arthritis; Back pain; Bone pain; Generalized muscle weakness; Muscle cramp; Myalgia; Neck pain; Pain in extremity
NEOPLASMS BENIGN, MALIGNANT AND UNSPECIFIED (INCL CYSTS AND POLYPS) -Tumor pain
NERVOUS SYSTEM DISORDERS - Ataxia; Cognitive disturbance; Depressed level of consciousness; Dysarthria; Extrapyramidal disorder; Intracranial hemorrhage; Lethargy; Memory impairment; Movements involuntary; Paresthesia; Peripheral motor neuropathy; Peripheral sensory neuropathy; Presyncope; Reversible posterior leukoencephalopathy syndrome; Stroke; Syncope; Tremor
PSYCHIATRIC DISORDERS - Agitation; Anxiety; Confusion; Depression; Insomnia; Psychiatric disorders - Other (emotional instability); Psychosis; Restlessness
RENAL AND URINARY DISORDERS - Dysuria; Hematuria; Proteinuria
RESPIRATORY, THORACIC AND MEDIASTINAL DISORDERS - Cough; Dyspnea; Epistaxis; Hypoxia; Nasal congestion; Pharyngolaryngeal pain; Pleural effusion; Pneumonitis; Respiratory failure
SKIN AND SUBCUTANEOUS TISSUE DISORDERS - Alopecia; Dry skin; Hyperhidrosis; Nail changes; Palmar-plantar erythrodysesthesia syndrome; Pruritus; Purpura; Rash acneiform
VASCULAR DISORDERS - Flushing; Hot flashes; Hypertension; Hypotension; Vascular disorders - Other (brainstem infarction)

Note: ABT-888 (Veliparib) in combination with other agents could cause an exacerbation of any adverse event currently known to be caused by the other agent, or the combination may result in events never previously associated with either agent.

7.1.2 Adverse Event List for irinotecan

Table 8: Adverse Event List for Irinotecan

Organ Site	Side Effect	Frequency, if known
Cardiovascular	Thromboembolism	5%
	Bradycardia (during infusion)	
	Ischemia	rare
	Hypotension	6%
	Vasodilatation – flushing	11%
Dermatologic	Alopecia	61%
	Hand foot syndrome	2%
	Rashes	14%
Gastrointestinal	Diarrhea	early 51%, late 88%
	Dehydration	15%
	Nausea	86%
	Vomiting	67%
	Abdominal pain	68%
	Dyspepsia/flatulence	12%
	Constipation	32%
	Colitis and ileus	rare
	Anorexia	55%
	Stomatitis	30%
Hematologic	Neutropenia	grade 4=12%; febrile=2-6%
	Thrombocytopenia	grade 4 =2%
	Lymphopenia	

Hepatic	Increased bilirubin	84%,grade 3-4 = 7%
	Elevated liver enzymes	grade 4= 13%
Neurologic	Dizziness	21%
	Confusion	3%
	insomnia	19%
	Somnolence	9%
Other	Fatigue	69%
	Fever, chills, sweats	45%
	Hyperglycemia	
	Edema	10%
	Pain	24%
	Weight loss	30%
	Hypersensitivity	Rare
	Renal failure	Rare
	Tumour lysis	Rare
	Infection	14%
Respiratory	Dyspnea	22%
	Non-productive cough	20%
	Rhinitis	16%
	Pneumonitis	Infrequent

7.2 Adverse Event Characteristics

- **CTCAE term (AE description) and grade:** The descriptions and grading scales found in the revised NCI Common Terminology Criteria for Adverse Events (CTCAE) version 3.0 will be utilized until July 31, 2010 for AE reporting. CTCAE version 4.0 will be utilized from August 1, 2010 until March 31, 2018 for AE reporting. CTCAE version 5.0 will be utilized for AE reporting beginning April 1, 2018. All appropriate treatment areas should have access to a copy of the CTCAE version 5.0. A copy of the CTCAE version 5.0 can be downloaded from the CTEP web site http://ctep.cancer.gov/protocolDevelopment/electronic_applications/ctc.htm .

- **'Expectedness'**: AEs can be 'Unexpected' or 'Expected' (see Section 7.1 above) for expedited reporting purposes only. 'Expected' AEs (the ASAEL) are ***bold and italicized*** in the CAEPR (Section 7.1.1).

- **Attribution** of the AE:
  - Definite – The AE *is clearly related* to the study treatment.
  - Probable – The AE *is likely related* to the study treatment.
  - Possible – The AE *may be related* to the study treatment.
  - Unlikely – The AE *is doubtfully related* to the study treatment.
  - Unrelated – The AE *is clearly NOT related* to the study treatment.

7.3 Expedited Adverse Event Reporting

7.3.1 Expedited AE reporting for this study must use CTEP-AERS (CTEP Adverse Event Reporting System), accessed via the CTEP home page (http://ctep.cancer.gov). The reporting procedures to be followed are presented in the

"CTEP, NCI Guidelines: Adverse Event Reporting Requirements" which can be downloaded from the CTEP home page (http://ctep.cancer.gov). These requirements are briefly outlined in the table below (Section 7.3.3).

In the rare occurrence when Internet connectivity is lost, an AE report may be submitted using CTEP's Adverse Event Expedited Report-Single Agent or Multiple Agent paper template (available at http://ctep.cancer.gov) and faxed to 301-230-0159. A 24-hour notification is to be made to CTEP by telephone at 301-897-7497, only when Internet connectivity is disrupted. Once Internet connectivity is restored, an AE report submitted on a paper template or a 24-hour notification phoned in must be entered electronically into CTEP-AERS by the original submitter at the site.

7.3.2 CTEP-AERS is programmed for automatic electronic distribution of reports to the following individuals: Study Coordinator of the Lead Organization, Principal Investigator, and the local treating physician. CTEP-AERS provides a copy feature for other e-mail recipients.

7.3.3 **Expedited Reporting Guidelines** – CTEP-AERS Reporting Requirements for Adverse Events That Occur Within 30 Days[1] of the Last Dose of the Investigational Agent on Phase 1 Trials

Table 9: Expedited Reporting Guidelines

Phase 1 Trials								
	**Grade 1**	**Grade 2**	**Grade 2**	**Grade 3**		**Grade 3**		**Grades 4 & 5[2]**
	**Unexpected and Expected**	**Unex-pected**	**Expected**	**Unexpected**		**Expected**		**Unexpected and Expected**
				**with Hospitali-zation**	**without Hospitali-zation**	**with Hospitali-zation**	**without Hospitali-zation**	
**Unrelated Unlikely**	Not Required	Not Required	Not Required	10 Calendar Days	Not Required	10 Calendar Days	Not Required	24-Hour; 5 Calendar Days
**Possible Probable Definite**	Not Required	10 Calendar Days	Not Required	24-Hour; 5 Calendar Days	24-Hour; 5 Calendar Days	10 Calendar Days	Not Required	24-Hour; 5 Calendar Days

[1] **Adverse events with attribution of possible, probable, or definite that occur greater than 30 days after the last dose of treatment with an agent under a CTEP IND require reporting as follows:**

CTEP-AERS 24-hour notification followed by complete report within 5 calendar days for:

- Grade 3 unexpected events with hospitalization or prolongation of hospitalization
- Grade 4 unexpected events
- Grade 5 expected events and unexpected events

[2] Although an CTEP-AERS 24-hour notification is not required for death clearly related to progressive disease, a full report is required as outlined in the table.

December 15, 2004

Use the NCI protocol number and the protocol-specific patient ID assigned during trial registration on all reports.

Note: All deaths on study require both routine and expedited reporting regardless of causality. Attribution to treatment or other cause must be provided.

Death due to progressive disease should be reported as **Grade 5 "Disease progression"** in the system organ class (SOC) "General disorders and administration site conditions." Evidence that the death was a manifestation of underlying disease (e.g., radiological changes suggesting tumor growth or progression: clinical deterioration associated with a disease process) should be submitted.

- Expedited AE reporting timelines defined:

- "24 hours; 5 calendar days" – The investigator must initially report the AE via CTEP-AERS within 24 hours of learning of the event followed by a complete CTEP-AERS report within 5 calendar days of the initial 24-hour report.
- "10 calendar days" - A complete CTEP-AERS report on the AE must be submitted within 10 calendar days of the investigator learning of the event.

- Any medical event equivalent to CTCAE grade 3, 4, or 5 that precipitates hospitalization (or prolongation of existing hospitalization) must be reported regardless of attribution and designation as expected or unexpected with the exception of any events identified as protocol-specific expedited adverse event reporting exclusions.

- Any event that results in persistent or significant disabilities/incapacities, congenital anomalies, or birth defects must be reported via CTEP-AERS if the event occurs following treatment with an agent under a CTEP IND.

- Use the NCI protocol number and the protocol-specific patient ID assigned during trial registration on all reports.

7.4 Routine Adverse Event Reporting

All Adverse Events **must** be reported in routine study data submissions. **AEs reported through CTEP-AERS must also be reported in routine study data submissions.**

7.5 Secondary AML/MDS

Investigators are required to report cases of secondary AML/MDS occurring on or following treatment on NCI-sponsored chemotherapy protocols using the NCI/CTEP Secondary AML/MDS Report Form. This form can be downloaded from the CTEP web site (http://ctep.cancer.gov). Refer to the "CTEP, NCI Guidelines: Adverse Event Reporting Requirements" (available at http://ctep.cancer.gov) for additional information about secondary AML/MDS reporting.

8. PHARMACEUTICAL INFORMATION

A list of the adverse events and potential risks associated with the investigational or commercial agents administered in this study can be found in Section 7.1.

8.1 CTEP IND Agent ABT-888 (NSC # 737664)

Chemical Name: 1H-Benzimidazole-7-carboxamide, 2-[(2R)-2-methyl-2-pyrrolidinyl]-

Other Names: Veliparib, A-861695.0

Classification: Poly (ADP-ribosome) polymerase (PARP) Inhibitor

CAS Registry Number: 912444-00-9

Molecular Formula: $C_{13}H_{16}N_4O$ **M.W.**: 244.29

Approximate Solubility: ABT-888 is freely soluble at pH < 6.9, soluble at pH 6.9 to 7.1, and slightly soluble at pH > 7.1**.**

Mode of Action: ABT-888 inhibits the formation of poly (ADP-ribose) (PAR) polymers in vitro and in vivo. It inhibits the repair of DNA when the DNA is damaged by cytotoxic agents. ABT-888 increases antitumor efficacy when added to DNA-damaging therapies such as temozolomide, cisplatin, carboplatin, cyclophosphamide, irinotecan, or radiation therapy.

Description: White to light yellow solid.

How Supplied: AbbVie supplies and DCTD distributes ABT-888. ABT-888 capsules are available in 10 mg, 20 mg, 40 mg, 50 mg, and 100 mg immediate release capsules. The inactive ingredients are microcrystaline cellulose, colloidal silicon dioxide, magnesium stearate, gelatin, sodium lauryl sulfate, and titanium dioxide. It may contain FD&C blue#1, FD&C yellow #6, or FD&C yellow #5. The capsules are packaged in HDPE bottles, and each HDPE bottle contains 16 capsules or 64 capsules.

Note: ABT-888 capsules may be repackaged from the supplied HDPE bottles into amber (or other low-actinic) child resistant pharmacy dispensing bottles. Expiration will be 30 days from the repackaging date (or the original retest date, whichever is earlier) when stored at 15°C to 25°C (59°F to 77°F).

Storage: Store the original bottle at 15° to 25° C (59° to 77° F).

Stability: Shelf life stability studies for ABT-888 capsules are on-going.

Route(s) of Administration: Oral. ABT-888 capsules may be administered without regard to meals.

Availability
ABT-888 is an investigational agent supplied to investigators by the Division of Cancer Treatment and Diagnosis (DCTD), NCI.

ABT-888 is provided to the NCI under a Collaborative Agreement between Abbvie Inc. and the DCTD, NCI (see Section 12.2).

Potential Drug Interactions: Nonclinical studies suggest ABT-888 is a substrate of P-gp, OCT2, and MATE1/MATE2K transporters. Co-administration of ABT-888 with strong inhibitors of P-gp, OCT2, and MATE1/MATE2K drugs may result in a decrease of ABT-888 renal clearance and an increase in ABT-888 plasma concentration. Therefore, use caution when administering ABT-888 with strong inhibitors of P-gp, OCT2, and MATE1/MATE2K drugs. At high dose (e.g., 400 mg BID), ABT-888 may inhibit OCT1 in the liver and MATE1/MATE2K in the kidney.

ABT-888 is not a potent inhibitor of the major human CYPs and does not significantly induce activities of major human CYP isoforms, suggesting a negligible potential for CYP-mediated drug-drug interactions as a perpetrator at the anticipated therapeutic concentrations.

In human, ABT-888 clears primarily in the urine as intact parent drug along with metabolites suggesting that renal function plays an important role in the drug clearance and its metabolites. Drug-associated with kidney toxicities or kidney diseases could change ABT-888 pharmacokinetics. Use cautions when concomitantly administer oxalipaltin, carboplatin, cisplatin, and topotecan in patients with pre-existing renal impairment.

Patient Care Implications: Patients may feel fatigue or tiredness. Loss of appetite and losing weight are common. Provide appropriate supportive care for diarrhea.

8.1.1 Agent Ordering
NCI-supplied agents may be requested by the responsible investigator (or their authorized designee) at each participating institution. Pharmaceutical Management Branch (PMB) policy requires that agent be shipped directly to the institution where the patient is to be treated. PMB does not permit the transfer of agents between institutions (unless prior approval from PMB is obtained). The CTEP-assigned protocol number must be used for ordering all CTEP-supplied investigational agents. The responsible investigator at each participating institution must be registered with CTEP, DCTD through an annual submission of FDA Form 1572 (Statement of Investigator), Biosketch, Agent Shipment Form, and Financial Disclosure Form (FDF). If there are several participating investigators at one institution, CTEP-supplied investigational agents for the study should be ordered under the name of one lead investigator at that institution.

Active CTEP-registered investigators and investigator-designated shipping designees and ordering designees can submit agent requests through the PMB Online Agent Order Processing (OAOP) application. Access to OAOP requires the establishment of a CTEP Identity and Access Management (IAM) account and the maintenance of an

"active" account status and a "current" password. For questions about drug orders, transfers, returns, or accountability, call or email PMB any time. Refer to the PMB's website for specific policies and guidelines related to agent management.

8.1.2 Agent Accountability

The investigator, or a responsible party designated by the investigator, must maintain a careful record of the receipt, dispensing and final disposition of all agents received from the PMB using the appropriate NCI Investigational Agent (Drug) Accountability Record (DARF) available on the CTEP forms page. Store and maintain separate NCI Investigational Agent Accountability Records for each agent, strength, formulation and ordering investigator on this protocol.

8.1.3 Investigator Brochure Availability

The current versions of the IBs for the agents will be accessible to site investigators and research staff through the PMB OAOP application. Access to OAOP requires the establishment of a CTEP IAM account and the maintenance of an "active" account status and a "current" password. Questions about IB access may be directed to the PMB IB Coordinator via email.

8.1.4 Useful Links and Contacts

- CTEP Forms, Templates, Documents: http://ctep.cancer.gov/forms/
- NCI CTEP Investigator Registration: RCRHelpDesk@nih.gov
- PMB policies and guidelines: http://ctep.cancer.gov/branches/pmb/agent_management.htm
- PMB Online Agent Order Processing (OAOP) application: https://eapps-ctep.nci.nih.gov/OAOP/pages/login.jspx
- CTEP Identity and Access Management (IAM) account: https://ctepcore.nci.nih.gov/iam/index.jsp
- CTEP Associate Registration and IAM account help: ctepreghelp@ctep.nci.nih.gov
- IB Coordinator: IBCoordinator@mail.nih.gov
- PMB email: PMBAfterHours@mail.nih.gov
- PMB phone and hours of service: (240) 276-6575 Monday through Friday between 8:30 am and 4:30 pm (ET)

8.2 Other Investigational Agent(s): N/A

8.3 Irinotecan hydrochloride injection (CPT-11, NSC#616348)

Chemical Name: (4S)-4,11-diethyl-4-hydroxy-9-[(4-piperidino-piperidino) carbonyloxy]-1H-pyrano[3',4':6,7]indolizino [1,2-b]quinoline-3,14(4H,12H)dione hydrochloride trihydrate

Classification: antineoplastic agent of the topoisomerase I inhibitor class.

Molecular Formula: $C_{33}H_{38}N_4O_6{\bullet}HCl{\bullet}3H_2O$ **M.W.**: 677.19

Description: Pale yellow to yellow crystalline powder.

How Supplied: Irinotecan is supplied as a sterile, pale yellow, clear, aqueous solution. It is available in two single-dose sizes: 2 mL-fill vials contain 40 mg irinotecan hydrochloride and 5 mL-fill vials contain 100 mg irinotecan hydrochloride. Each milliliter of solution contains 20 mg of irinotecan hydrochloride (on the basis of the trihydrate salt), 45 mg of sorbitol NF powder, and 0.9 mg of lactic acid, USP. The pH of the solution has been adjusted to 3.5 (range, 3.0 to 3.8) with sodium hydroxide or hydrochloric acid. CAMPTOSAR is intended for dilution with 5% Dextrose Injection, USP (D5W), or 0.9% Sodium Chloride Injection, USP, prior to intravenous infusion. The preferred diluent is 5% Dextrose Injection, USP.

Storage: Stored at controlled room temperature 15° to 30°C (59° to 86°F). Protect from light. It is recommended that the vial (and backing/plastic blister) should remain in the carton until the time of use.

Route of Administration: Intravenous infusion over a period of 90 minutes.

Solution preparation: Irinotecan must be diluted prior to infusion. Irinotecan should be diluted in 5% Dextrose Injection, USP, (preferred) or 0.9% Sodium Chloride Injection, USP, to a final concentration range of 0.12 to 2.8 mg/mL. In most clinical trials, irinotecan was administered in 250 mL to 500 mL of 5% Dextrose Injection, USP. The solution is physically and chemically stable for up to 24 hours at room temperature and in ambient fluorescent lighting. Solutions diluted in 5% Dextrose Injection, USP, and stored at refrigerated temperatures (approximately 2° to 8°C), and protected from light are physically and chemically stable for 48 hours. Refrigeration of admixtures using 0.9% Sodium Chloride Injection, USP, is not recommended due to a low and sporadic incidence of visible particulates. Freezing irinotecan and admixtures of irinotecan may result in precipitation of the drug and should be avoided. Because of possible microbial contamination during dilution, it is advisable to use the admixture prepared with 5% Dextrose Injection, USP, within 24 hours if refrigerated. In the case of admixtures prepared with 5% Dextrose Injection, USP, or Sodium Chloride Injection, USP, the solutions should be used within 6 hours if kept at room temperature. Other drugs should not be added to the infusion solution. Parenteral drug products should be inspected visually for particulate matter and discoloration prior to administration whenever solution and container permit.

Availability: Irinotecan HCL injection is commercially available.

Additional Information: CAMPTOSAR (irinotecan hydrochloride injection) Package Insert. New York, NY: Pfizer Inc.; 2006.
http://www.pfizer.com/pfizer/download/uspi_camptosar.pdf

9. CORRELATIVE/SPECIAL STUDIES

9.1 Laboratory Correlative Studies (DOSE ESCALATION PORTION ONLY – THIS SECTION IS NOT USED IN THE EXPANSION PORTION. SEE SECTION 9.2 FOR LABOARATORY CORRELATES RELATED TO THE EXPANSION PORTION AND SECTION 9.3 FOR LABORATORY CORRELATES RELATED TO THE DOSE

ESCALATION FOR INTERMITTENT ABT-888 PORTION)

See Appendix C for a full PK and PD sample collection schedule.

9.1.1 PAR Immunoassay (Dose Escalation Portion ONLY)

9.1.1.1 Outcome Measure(Dose Escalation Portion ONLY)

PAR levels from specimens will be reported as pg/mL and as pg or PAR per 10^7 PBMC isolated. PAR levels will be compared from baseline and from predetermined timepoints to derive a percentage change. Standard Operating Procedures for the PAR immunoassay are included in Appendix D and Appendix E.

9.1.1.2 Assessment(Dose Escalation Portion ONLY)

PAR concentration is determined by immunoassay. Briefly, 96-well microtitre plates were coated with a monoclonal antibody specific for PAR. After a blocking step, samples are diluted into a buffer and incubated with samples (16 hours at 4 ^oC). Following a washing step, a rabbit antiserum to PAR is added to each well, and an additional 2 hour incubation (at 22 ^oC) is performed. Another washing step is performed, and a peroxidase-conjugated anti-rabbit antibody is added to each well, and incubated for one hour (at 22 ^oC). Wells are washed again, a luminol substrate is added, and the assay wells are read in a Tecan Luminometer. A set of calibrators and assay controls set at different PAR levels are run with each assay.

Collection and processing of PBMCs and tumor samples(Dose Escalation Portion ONLY)

PBMCs should be collected in CPT citrate tubes and processed within 2 hours of collection, according to the standard operating procedure (SOP) provided in Appendix D.

Surgical tumor tissue should be collected in pre-cooled cryovials and immediately flash frozen in liquid nitrogen, then stored at -80 degrees C. Tissues should be shipped on dry ice by overnight delivery service. The National Clinical Target Validation Laboratory (NCTVL) should be notified at least one day in advance to schedule specimen delivery time and locations, including room number and contact phone number. Questions concerning collection, storage, and shipment of tumor tissue should be directed to:

[redacted], Ph D
National Clinical Target Validation Laboratory
DTP, DCTD, National Cancer Institute
37 Convent Drive
[redacted]
Bethesda, MD 20892
[redacted]
Email: [redacted]

Timing of Assessment(Dose Escalation Portion ONLY)

Dose escalation portion:
Based on the data derived from the phase 0 study, the following collection schedule will be used:

PBMC collection times should occur prior to therapy, and then 3.5, 5.5, 8.5, 28, and 48 hours after the start of infusion of irinotecan as a single agent (starting Day 1, Cycle 1) or with irinotecan in combination with ABT-888 (starting Day 8, Cycle 1). In Cycle 2, PBMC will be collected prior to ABT-888 dosing on Cycle 2, Day -1, and at the following times starting Day 8, Cycle 2: prior to CPT-11 infusion/ABT-888 dosing, 5.5 h after start of infusion/ABT-888 dosing, and 28 h after start of infusion/ABT-888 dosing. The first dose of ABT-888 to be administered on Day 8 of Cycle 1 and Day 8 of Cycle 2 will be taken at the start of CPT-11 infusion, so sampling can be lined up for both drugs. The blood samples drawn on Day 9 of Cycle 1 and Day 9 of Cycle 2 will occur 28 hours after start of the combination of ABT-888 and chemotherapy on Day 8 of Cycle 1 and Cycle 2, respectively, and 4 hrs after the first Day 9 dose of ABT-888 of Cycle 1 and Cycle 2, respectively. Measurements of PAR levels over time in PBMC's for the initial few patients might be used to more optimally define precise biopsy times in subsequent patients.

Tumor collection should occur at two times:

- First biopsy: 28 hours after chemotherapy alone (Day 2, Cycle 1)
- Second Biopsy: 28 hours after start of the combination of ABT-888 and chemotherapy (Day 9, Cycle 1) and 4 hrs after the first Day 9, Cycle 1 dose of ABT-888.

9.1.2 Pharmacokinetic (PK) Evaluation (Dose Escalation Portion only)

9.1.2.1 Collection and processing (Dose Escalation Portion ONLY)

The study of systemic disposition of CPT-11 and ABT-888 will be conducted in all treated patients at the following times:

Sampling time:

- Cycle 1, Day 1, 2, and 3 : (to characterize the PK of CPT-11 alone):
  Blood samples will be collected at: pre-infusion (8ml), 30 min after the start of infusion (4ml), immediately at the end of infusion (EOI) (~89 min, 4ml), and 2h (4ml), 3.5h (8ml), 5.5h (8ml), 8.5h (8ml) 28h (8 ml) and 48h (8ml) after the start of infusion. Blood drawn at pre-infusion, and 3.5 hours, 5.5 hours, 28 hours, and 48 hours after the start of infusion will be used for separation of plasma (for PK assay) and PBMC (for either PAR assay or PD assay, see Appendix C).
- Cycle 2, day -1 and 1: (to characterize the PK of ABT-888 alone)

A single-dose of ABT-888 will be given on Day -1 while in the clinic, and blood samples will be obtained at: pre-dosing (8ml), 0.5h (4ml), 1h (4ml), 1.5h (4ml), 3.5h (4ml), 5.5h (8ml), 8.5h (4ml), 10h (4ml), and 28h (8ml) after the dosing. Blood drawn at pre-dosing, 5.5 hours, and 28 hours after the dosing will be used for separation of plasma (for PK assay) at KCI and PBMC (for PD assay) at KCI (see SOP for PK-PBMC collection, appendix G). ABT-888 on Cycle 2, Day 1 will be taken twice, with the first daily dose taken after the 28h blood draw.

- Cycle 2, day 8, 9, and 10: (to characterize the steady-state PK of CPT-11 and ABT-888 in the combination)
  The first and second dose of ABT-888 to be administered on day 8 will be dispensed in the clinic. The first dose of ABT-888 will be taken at the start of CPT-11 infusion, so PK sampling can be lined up for both drugs. Blood samples will be collected at: pre-infusion (8ml), 30 min (4ml) and 1 hour (4ml) after the beginning of infusion, immediately end of infusion (EOI) (~89 min, 4ml), and 2h (4ml), 3.5h (4ml), 5.5h (8ml), 8.5h (4ml), 10h (4ml, just prior to the administration of the second dose of ABT-888 on day 8), 28 h (8ml, prior to ABT-888 administration on day 9), and 48 h (4ml, prior to ABT-888 administration on day 10). Blood drawn at pre-infusion, 5.5 hours after the infusion, and 28 hours after the infusion will be used for separation of plasma (for PK assay) and PBMC (for PD assay).

9.1.2.2 Sample processing(Dose Escalation Portion ONLY):

9.1.2.2.1 4ml blood processing(Dose Escalation Portion ONLY):

At the time points specified in Section 9.1.2.1 above, if 4 ml blood sample is collected, the sample will be processed as detailed below in order to separate plasma for PK assay only:

4 ml blood sample will be collected into a tube with sodium citrate anticoagulant. The actual day and time of sample collection will be recorded on the PK worksheet (see Appendix G). Within 1 h of the collection, the blood sample will be centrifuged at 25^{0}C, at 3000 rpm for 10 min, and plasma will be collected immediately after centrifugation and transferred to 4 screw-cap polypropylene cryogenic tubes (4 aliquots). The tube will be labeled with the patient's initial, study number, sample collection day and time, and frozen at -70^{0}C or below until analysis.

9.1.2.2.2 8 ml Blood processing(Dose Escalation Portion ONLY):

At the time points specified in Section 9.1.2.1 above, if 8 ml blood sample is collected, the sample will be processed as detailed below in order to separate plasma (for PK assay) and PBMC (for PD assay):
8 ml blood samples will be collected into 8-ml BD Vacutainer® CPT™

Cell Preparation Tube with sodium citrate anticoagulant (BD REF No. 362761, Franklin Lakes, NJ), and put upright at room temperature until centrifuge. The actual day and time of sample collection will be recorded on the PK worksheet (see Appendix G). Within 1 h of the collection, the sample will be mixed by inverting the tube for 5-8 times and centrifuged at 1500 g for 30 min at 18-25°C. Two thirds of plasma will be collected immediately after centrifugation and transferred to four screw-cap polypropylene cryogenic tubes (four aliquots), and freeze at -70^{0}C or below until analysis of ABT-888 and/or irinotecan concentration. Whitish PBMC layer (middle layer) will be transferred into the labeled 15-ml conical centrifuge tube with cap. The PBMC will be washed twice, and viable cell number will be counted. The cell pellet will be re-suspended to yield a cell suspension containing 3 x 10^6 viable PBMCs per ml. 0.5 ml aliquots of PBMC cell suspension will be transferred into 2.0-ml cryovials, and centrifuged at 2000 x g for 10 minutes at 4-10°C. The resulted cell pellets will be frozen at -80°C until shipped out.

9.1.2.3 Bioanalysis assays(Dose Escalation Portion ONLY):

The plasma concentrations of CPT-11 and its metabolites (i.e., SN-38, SN-38G, NPC, and APC) will be measured using a validated high-performance liquid chromatographic (HPLC) method with tandem mass spectrometric detection (59). A reversible, pH-dependent hydrolysis converts the closed lactone form of CPT-11 and its metabolites (i.e., SN-38, SN-38G, NPC, and APC) to the open carboxylate form of each compound. Simultaneously determination of the lactone and carboxylate forms is compromised by stability problem associated with lactone-carboxylate inter conversion (60). It has been reported that the pharmacokinetics of total CPT-11 and SN-38 are significantly correlated with those of lactone CPT-11 and SN-38, respectively (61). Our method will quantitatively measure the total CPT-11 and its metabolites by acidifying the samples prior to analysis to convert the carboxylate form to lactone form of each compound.

The plasma concentrations of ABT-888 and its main metabolite (A-925088) will be measured using a validated HPLC method with tandem mass spectrometer (LC-MS/MS).

All PK samples will be processed, stored, and analyzed in the Barbara Ann Karmanos Cancer Institute Pharmacology Core Laboratory according to Standard Operating Procedures.

9.1.2.4 PK-PD analysis(Dose Escalation Portion ONLY):

Non-compartmental analysis using WinNonlin 5.0 (Pharsight Corp., Mountain View, CA) will be used to derive drug exposure parameters (i..e, C_{max}, C_{min}, and AUC). Population PK-PD analysis using NONMEM program (version V) (University of California, San Francisca, CA) will be performed to explore drug-drug interaction and

PK-PD relationship.

9.1.2.5 Shipping(Dose Escalation Portion ONLY)

At the end of the PK collection period for each patient's cycle, samples will be shipped to the Barbara Ann Karmanos Cancer Institute sample processing unit. Shipments should only be made Monday through Wednesday. Prior to any shipment, please notify the KCI Pharmacology Laboratory via email and/or telephone. The samples will be shipped to the following address:

Attention: [redacted]
Barbara Ann Karmanos Cancer Institute
Wayne State University
4100 John R, HW05AO
[redacted]
Detroit, MI 48201
Office: [redacted]
Fax: [redacted]
Email: [redacted]

9.1.3 Pharmacodynamic (PD) Evaluation (Dose Escalation Portion ONLY)

9.1.3.1 Outcome measure (Dose Escalation Portion ONLY)

PD endpoints are PAR activity inhibition in peripheral blood mononuclear cells and tumor cells (PBMC, as explained in Section 9.1.1 above), as well as the detection of γ-H2AX foci and/or Rad51 foci in pre- and post-treatment tumor biopsies. In addition, the status of repair proteins such as ERCC1, Topo I, p53 and BRCA1 levels will be determined in archival tissue blocks. The latter two proteins could prove useful in predicting which patient population might benefit most from ABT-888/irinotecan treatment and aid future selection of patients that are likely to respond.

The detection of phosphorylated γ-H2AX in tumor cells by immunocytochemical methods (immunofluorescence and quantification of γ-H2AX foci), is an established procedure in the KCI Translational Research Laboratory (TRL) and is currently being employed for PD endpoint detection in another NCI trial with a TRI component (62,63).

The KCI TRL is equipped with a Leica DM5500 microscope with fluorescence light and phase contrast light sources, cooled camera with monochrome and color filters and imaging software (Openlab, Improvision). The latter allows for quantification of γ-H2AX foci and/or Rad51 foci formation.

9.1.3.2 Collection and processing (Dose Escalation Portion ONLY):

The sampling scheme will be employed as follows:

PD Sampling:

Baseline gamma-H2AX, Rad51 and expression of ERCC1 and Topo-I should be determined in the biopsy obtained 28 hours after the start of single agent irinotecan (CPT-11) treatment (Cycle 1, Day 2). Biopsies for the measurement of H2AX and Rad51 foci formation are collected 28 hours after the beginning of infusion of the second treatment of CPT 11 combined with ABT-888 (Cycle 1, Day 9) and 4 hours after the first Cycle 1, Day 9 dose of ABT-888. This allows for the measurement of the occurrence of DSB after single agent CPT-11 in absence of PARP inhibition and in combination with ABT-888.

PBMCs for PD assays will be separated from plasma collected for the purpose of PK analysis at the following timepoints:

- Cycle 1, Day 1: Pre-infusion and 5.5 hours after start of single agent irinotecan (CPT-11) infusion
- Cycle 1, Day 2: 28 hours after start of single agent irinotecan (CPT-11) infusion
- Cycle 2, Day -1: 5.5 hours after single agent ABT-888 dosing.
- Cycle 2, Day 1: 28 hours after single agent ABT-888 dosing

γ-H2AX and Rad51 in PBMCs and tumor tissues will be performed if sufficient tumor material is available for analyses. Using archival tissue blocks, ERCC1, p53, BRCA1, and Topo-I protein expression will be determined by immunohistology and ERCC1 polymorphism examined with DNA extracted from 5-10 paraffin sections.

9.1.3.3 Specimen Processing (Dose Escalation Portion ONLY):

Blood samples: One 8cc BD vaccutainer with sodium heparin will be used to collect blood for the isolation of PBMCs.
Cytospins and protein lysates should be prepared from PBMCs and tumor according to Standard Operating Procedures and assay requirements in the Translational Laboratories at NCI or KCI (see Appendix C).

Tissue biopsies: Two core biopsies (one core biopsy for PD analysis at KCI and the other to be sent to the NCI National Clinical Target Validation Laboratory [NCTVL]) will be collected and kept on ice (at 4°C) until further processed. The core biopsy for the NCTVL will be collected according to their provided SOP (Appendix E).

Cytospins and/or cryosections (see Appendix F): Cytospin slides will first be dried for 5 to 10 minutes on air. Cells will then be fixed in a 1:1 mixture of ice cold methanol and acetone for 1 minute, the procedures is repeated three times. Slide are subsequently dried for 15 min. at RT. Cryosections will be prepared by placing a portion of the biopsy tissue into OCT freezing media and 5-10 μm sections cut with a cryostat, fixed and air dried. Slides can directly be used for staining as detailed below.

H2AX and Rad51 foci formation: Slides will be re-hydrated in PBS for 10 min and blocked in PBS with 5% bovine serum albumin overnight. The cells are then incubated with 1:100 anti-phospho-histone 2A.X (Ser 139, Upstate) and 1:1,000 anti-Rad51 (H-92, St. Cruz) for two hours at RT. Secondary antibodies are anti-mouse FITC (Sigma) 1:100 for 2 hours in 5% BSA or anti-rabbit TRITC 1:100 for 2 hours in BSA. Images will be captured with a Leica DM5500 fluorescence microscope and Retiga cooled camera using Improvision Openlab 5.0 software. Signal intensity will be evaluated by using Openlab quantification software as well as the number of positive cells per 100 cells counted and reported.

<u>ERCC1, p53, BRCA1 and Topo-I immunohistochemistry (optional):</u> Cytospins or dewaxed and hydrated paraffin sections (5 µm) will be stained with either anti-ERCC1 (mouse, clone 8F1, Neomarkers), p53 , BRCA1 or Topo-I antibodies (mab 6B5, St. Cruz) and developed using immunoperoxidase methods (Envision Plus kit, DAKO) and DAB (diaminobenzidine) as a substrate as described by others before (64,65).

<u>ERCC1 polymorphism:</u> DNA extraction from paraffin section is established in our laboratory as published by us before (66). ERCC1 codon 118 polymorphism genotyping will be performed according to procedures published by Viguier and colleagues (44).

9.1.3.4 Shipping for PD samples (Dose Escalation Portion ONLY):

PD samples for the analysis of PARP activity inhibition in peripheral blood leukocytes will be shipped to the National Clinical Target Validation Laboratory at the NIH as explained above. Analysis of PD samples for phosphorylated H2AX and Rad51 foci formation, Topoisomerase I, p53 and BRCA1 expression, and expression and polymorphism analyses of ERCC1 will be performed at the Translational Research Laboratory of Karmanos Cancer Institute. Archival tissue blocks or ten paraffin sections freshly cut from archival tissue blocks can be provided to the TRL at KCI and should be shipped separately at room temperature. Cytospin slides and frozen tissue must be stored at –20°C and shipped to KCI on dry ice.

Shipments should only be made Monday through Wednesday. Prior to any shipment, please notify the KCI Pharmacology Laboratory via email and/or telephone. The samples will be shipped to the following address:

Attention: [redacted]
Barbara Ann Karmanos Cancer Institute
Wayne State University
4100 John R, HW05AO

[redacted]
Detroit, MI 48201
Office [redacted]
Fax: [redacted]
Email: [redacted]

9.1.4 Pharmacogenomic Evaluation (Dose Escalation Portion)

9.1.4.1 Outcome measure (Dose Escalation Portion ONLY):

Polymorphisms in UGT1A1 (e.g., UGT1A1*28 variant), CYP2C9 (e.g., CYP2C9*2 and *3 variants) and CYP2C19 (e.g., CYP2C19*2 and *3 variants), and ABCG2 (e.g., ABCG2 421C>A) will be determined using previously reported methods (50-53).

9.1.4.2 Specimen processing (Dose Escalation Portion ONLY):

One 10 mL blood sample will be collected in EDTA (purple top) tube at pretreatment (within one week), and be frozen at -70°C or below until the extraction of DNA using commercial DNA isolation kits, such as Puregene (Gentra Systems, Inc., Minneapolis, MN).

9.1.4.3 Shipping (Dose Escalation Portion ONLY):

Pharmacogenetic analyses will be performed in the Barbara Ann Karmanos Cancer Institute Pharmacology Core Laboratory.

Shipments should only be made Monday through Wednesday. Prior to any shipment, please notify the KCI Pharmacology Laboratory via email and/or telephone. The samples will be shipped to the following address:

Attention: [redacted]
Barbara Ann Karmanos Cancer Institute
Wayne State University
4100 John R, HW05AO
[redacted]
Detroit, MI 48201
Office: [redacted]
Fax: [redacted]
Email: [redacted]

9.1.5 Tissue Sample Priority (Dose escalation portion ONLY)

Tumor tissue will be triaged as specified. Top priority will be given for the first core biopsy for PAR assays, second priority will be given for the second core biopsy for gamma-h2AX assays, and lowest priority for leftover tissue from the second core biopsy for all other assays and touch preps.

1.) First core biopsy for PAR assay (NCTVL)
2.) Second core biopsy: for cytospin or cryosectioning for simultaneous measurement of phosphorylated H2AX and Rad51 foci formation. (Dr. [redacted] Karmanos)

3.) Leftover tissue from second core biopsy for hematoxylin and eosin (H&E) pathology confirmation (institutional)
4.) Leftover tissue from second core biopsy for RNA or protein extraction (Dr. [redacted] Karmanos)
5.) Touch preps (Dr. [redacted] Karmanos)

9.2 Laboratory Correlative Studies (EXPANSION PORTION)

For the Expansion Portion of this study, blood, fresh tissue, and archived tissue, will be collected according to Table 2 in Appendix C. Below is a summary of the sample collection:

Blood:

At baseline, 1-2 acid citrate dextrose (ACD) yellow top collection tubes with 10-20mls of peripheral blood will be collected using standard blood collection techniques and will be shipped to TGen overnight on ice for the detection of somatic mutations.

PBMCs will be collected and shipped to the NCTVL on dry ice. PBMCs will be collected at the time of the baseline biopsy, and then immediately prior to therapy, 4-6, 8, 22, and 24 hours after the start of infusion of irinotecan as a single agent (starting Day 1, Cycle 1) or with irinotecan in combination with ABT-888 (starting Day 8, Cycle 1). At each time point, 8 mL PBMCs should be collected in CTP citrate tubes and processed within 2 hours of collection, according to the standard operating procedure provided in Appendix D.

Whole blood for the purpose of CTC analysis collection times should occur at the following times (8 ml each timepoint): at the time of the baseline biopsy, and then immediately prior to therapy, 4-6, 8, 22, and 24 hours after the start of infusion of irinotecan as a single agent (starting Day 1, Cycle 1) and with irinotecan in combination with ABT-888 (starting Day 8, Cycle 1). Finally, a single draw of CTCs will occur on Day 15 of Cycle 1. Samples will be shipped to the Pharmacodynamic Assay Development and Implementation Section Laboratory of Human Toxicology & Pharmacology (LHTP) at room temperature.

Fresh Tissue:

For fresh tissue collection, the first biopsy will occur within 2 weeks of first treatment; second biopsy will occur 4-6 hours after beginning of the Day 1, Cycle 1 infusion of single agent irinotecan; third biopsy will occur 4-6 hours after beginning of the Day 8, Cycle 1 infusion of irinotecan (the second daily dose of ABT-888 scheduled on this day will occur following the biopsy).

Each biopsy sampling will consist of 4-6 passes with a 16 or 18 gauge needle or a surgically removed specimen if the lesion is easily accessible by a surgeon. One core biopsy sample (or approx. 1/4 of total tissue sample) will be snap frozen and sent to the National Clinical Target Validation Laboratory (NCTVL) for analysis of gamma-H2AX/ERCC1 on dry ice. Two core biopsy samples (or approx. 1/2 of total tissue sample) will be placed in culture media (MEGM Lonza CC3051A) and sent by overnight express on ice/cold packs to the laboratory of Dr. [redacted] at the University of Michigan. A fourth core biopsy sample (or approx. 1/4 of total tissue

sample) will be snap frozen and shipped to TGen for genomic analysis of the bulk tumor.

If the availability of fresh tissue is very limited, a triaging system will be applied (see section 9.2.5).

Archived Tissue:
When available, archival blocks will be sent to AbbVie and made into slides for subsequent IHC analyses.

NOTE:
When a patient has been enrolled and a biopsy date set, please notify the following people so that they can reserve time for their research equipment:
[redacted]

9.2.1 Measurement of DNA damage repair pathway consequences of PARP inhibition treatment with chemotherapy in PBMCs (Expansion Portion)

9.2.1.1 Outcome Measure(EXPANSION PORTION)

PD endpoints include levels of γ-H2AX, ERCC1, and other markers of the DNA damage repair pathway as warranted.. Levels will be compared from baseline and from predetermined timepoints coinciding with CTC and biopsy collection to derive a percentage change and compare such changes across sample types. Standard Operating Procedures for the collection of PBMCs are included in Appendix D.

9.2.1.2 Collection and processing of PBMCs (EXPANSION PORTION)

PBMCs: At each time point, 8 mL PBMCs should be collected in CTP citrate tubes and processed within 2 hours of collection, according to the standard operating procedure (SOP) provided in Appendix D.

Briefly, an email will be sent in advance to advise recipient of scheduled shipping time and ask the recipient to reply the email for sample receiving conformation. Sample tubes/vials for each patient will be inserted in watertight biohazard specimen bags and placed in a shipping container with sufficient dry ice to maintain the samples in frozen state for at least 8 hours. Note that specimens should be shipped Monday through Thursday and may not be shipped over Federal Holiday Weekends (when the receiving facility is closed).

[redacted] Ph D
National Clinical Target Validation Laboratory
DTP, DCTD, National Cancer Institute
37 Convent Drive
[redacted]
Bethesda, MD 20892
[redacted]

[redacted]
Email: [redacted]

Timing of Assessment (EXPANSION PORTION)

PBMC collection times should occur at the time of the baseline biopsy, and then immediately prior to therapy, 4-6, 8, 22, and 24 hours after the start of infusion of irinotecan as a single agent (starting Day 1, Cycle 1) or with irinotecan in combination with ABT-888 (starting Day 8, Cycle 1). The first dose of ABT-888 to be administered on Day 8 of Cycle 1 will be taken at the start of CPT-11 infusion, so sampling can be lined up for both drugs. The blood samples drawn on Day 9 of Cycle 1 will occur 22 and 24 hours after start of the combination of ABT-888 and chemotherapy on Day 8 of Cycle 1. The first dose of ABT-888 taken on Day 9 should occur following the 24-hr blood draw.

9.2.2 Gamma-H2AX Assay and ERCC1 evaluation in CTCs (EXPANSION PORTION)

9.2.2.1 Outcome Measure (EXPANSION PORTION)

The outcome measure of this assay is reported as the percent γH2AX positive nuclei-containing cells. Circulating tumor cells (CTCs) will be extracted from both tumor tissue and whole blood. Standard Operating Procedures for the preparation of CTCs for the Gamma-H2AX assay are included in Appendix J. The core biopsies for the NCTVL will be collected according to their provided SOP (Appendix E).

9.2.2.2 Assessment (EXPANSION PORTION)

The γH2AX immunofluorescence assay (IFA) is an immunohistochemistry-based staining assay developed to quantify the nuclear DNA damage marker, histone H2AX phosphorylated at serine 139 (γH2AX). The assay uses a biotinylated-γH2AX monoclonal antibody as the detector and an Alexa Fluor 488-streptavidin conjugate (Strp488) as the reporter for immunostaining.

Collection and processing of CTC samples from whole blood (EXPANSION PORTION)

CTCs are enriched from whole blood and isolated using the CellSearch System, an automated system for the purification and enumeration of CTCs with the capability of detecting a single CTC in 7.5 mL of whole blood. For this study, 8 ml whole blood will be collected in a CellSave Preservation Tube and shipped to the NCI-PADIS to be processed in the CellSearch system within 96 hours, according to the standard operating procedures (SOP) provided in Appendix J.

Collection and processing of tumor tissue (EXPANSION PORTION)

Tumor needle biopsies will be collected at the clinical site and preserved by cryopreservation. One core biopsy to be sent to the NCI National Clinical Target Validation Laboratory [NCTVL] will be collected and immediately cryopreserved in separate tubes in liquid nitrogen until further processed. This single core biopsy will be used for PD biomarker analyses (gamma-H2AX, ERCC1). The core biopsy for the NCTVL will be collected according to their provided SOP (Appendix E).

Shipping (EXPANSION PORTION):
CTCs: Samples should be shipped in a shipping container that is appropriate for shipping glass tubes containing human blood, provides shock absorption, and can maintain RT (15°C to 25°C) for at least 72 h. CTC specimen shipping kits will be provided by PADIS/LHTP/NCI-Frederick. The kits will have pre-addressed shipping labels and a billing account number. See Appendix J for sample handling, processing, and shipping instructions. Note that specimens should be shipped Monday through Thursday and may not be shipped over Federal Holiday Weekends (when the receiving facility is closed). The Pharmacodynamic Assay Development and Implementation Section (PADIS) should be notified at least one day in advance, preferably by email, to schedule specimen delivery time and locations, including room number and contact phone number. Please also call Dr. [redacted] before shipping. Questions concerning collection, storage, and shipment of CTCs should be directed to:

[redacted] and [redacted]

The CTC shipping address is:
Dr. [redacted]
PADIS, LHTP
FNLCR/SAIC-Frederick, Inc.
1050 Boyles Street
[redacted]
Frederick, MD 21702-1201
U.S.A.

Tissue: Samples should be shipped on dry ice by overnight delivery service. The National Clinical Target Validation Laboratory (NCTVL) should be notified at least one day in advance to schedule specimen delivery time and locations, including room number and contact phone number. Questions concerning collection, storage, and shipment of tumor tissue should be directed to:

[redacted] Ph D

National Clinical Target Validation Laboratory
DTP, DCTD, National Cancer Institute
37 Convent Drive
[redacted]
Bethesda, MD 20892
[redacted]
Email: [redacted]

Timing of Assessment (EXPANSION PORTION)

Whole blood for the purpose of CTC analysis collection times should occur at the following times (8 ml each timepoint): at the time of the baseline biopsy, and then immediately prior to therapy, 4-6, 8, 22, and 24 hours after the start of infusion of irinotecan as a single agent (starting Day 1, Cycle 1) and with irinotecan in combination with ABT-888 (starting Day 8, Cycle 1). Finally, a single draw of CTCs will occur on Day 15 of Cycle 1. The first dose of ABT-888 to be administered on Day 8 of Cycle 1 will be taken at the start of CPT-11 infusion, so sampling can be lined up for both drugs. The CTC samples drawn on Day 9 of Cycle 1 will occur 22 and 24 hours after start of the combination of ABT-888 and chemotherapy on Day 8 of Cycle 1. The first dose of ABT-888 taken on Day 9 should occur following the 24-hr blood draw. The CTC sample drawn on Day 15 will occur 4 hrs after the first Day 15 dose of ABT-888 scheduled that day. Measurements of biomarker levels over time in CTCs for the initial few patients might be used to more optimally define precise biopsy times in subsequent patients.

Tumor collection (4-6 passes with an 18 gauge needle or a surgically removed specimen if the lesion is subcutaneous and easily accessible by a surgeon) should occur at three times:

- First biopsy: within 2 weeks prior to treatment on Day 1
- Second biopsy: 4-6 hours after the start of infusion of irinotecan (Day 1, Cycle 1)
- Third Biopsy: 4-6 hours after the combination of ABT-888 and start of infusion of irinotecan (Day 8, Cycle 1). The second daily dose of ABT-888 scheduled on this day will occur following the biopsy.

9.2.3 Breast Cancer Stem Cell Evaluations (EXPANSION PORTION)

9.2.3.1 Assay Descriptions (EXPANSION PORTION)

9.2.3.1.1 Mammosphere Assay (EXPANSION PORTION)

Dr. [redacted] laboratory will asses the percentage of breast cancer stem cells in the tissue using methods as previously described. Briefly, single-cell suspensions established from biopsies will be stained with antibodies against the breast cancer markers CD24, CD44, and/or ALDH and analyzed by flow cytometry. In addition, the

potential of cells from biopsy samples to form mammospheres in culture will also be compared. Mammosphere-forming efficiency (MSFE) assays will be performed by plating isolated single-cell suspensions on non-adherent plastic and seeding 20,000 cells in ultra-low attachment dishes. Primary mammospheres will be allowed to grow 3 weeks before being counted.

9.2.3.1.2 Biomarker Study (EXPANSION PORTION)

Dr. [redacted] laboratory will examine the activities of DNA repair pathway molecules including RAD51. Stem cells will be isolated from core biopsy samples using flow cytometry with Aldefluor To separate ALDH+ and ALDH- cells. Then the cells will be cytospun and stained for RAD51 foci.

For RT-PCR determinations, total RNA will be extracted using Trizol Reagent (Invitrogen). Total RNA (100 ng per sample, in triplicate) will be reverse-transcribed (M-MLV Reverse Transcriptase, Invitrogen) following the manufacturer's protocol. The resulting cDNA will be analyzed using an Applied Biosystems 7500-Fast thermocycler for TaqMan quantitative PCR (Q-PCR) using standard conditions. TaqMan Assay On Demand primers and probes will be acquired from Applied Biosystems.

*AQUA© Methodology*The AQUA© process is a two-phase experimental method. The first step is a modified immunofluorescent histochemical technique involving the double immunolabeling of the tissue, with primarily a 'tumor mask' (diagnosis specific, i.e. cytokeratin for epithelial cells) which labels all the cancer cells within a tissue section, and secondly the reaction of the section with the antibody directed at the target of interest (ALDH1, RAD51, etc.). All antibodies are individually validated using AQUA© proprietary algorithms. The immuno-reactivity is developed using species-specific secondary antibodies labeled with different fluorochromes. Finally, the sections are counterstained with DAPI and cover-slipped using an anti-fade mounting media. The second phase of the AQUA© process is the acquisition of separate images using the different fluorochromes (DAPI, FITC, Alexafluor, CY3, CY5 and CY7). The AQUA© software will examine each tissue section, and the absolute AQUA© score on each tested protein will be determined.

The Department of pathology at the University of Michigan owns an AQUA© system, and has demonstrated expertise with this technology and this instrument. This effort has been led by Dr. [redacted], a fully trained pathologist

at the University of Michigan Health System with experience using a DAKO Cytomation autostainer and HistoRx PM2000 (AQUA© analysis machine) in the Molecular Pathology Research laboratory, a facility dedicated to the quantitative analysis of markers in pathology specimens. The staining, imaging, and data organization and analysis will be performed by Dr. [redacted]

9.2.3.1.3 Exome Profiling (EXPANSION PORTION)

We will capture and resequence all annotated human coding regions using the Agilent SureSelect All Exon kit (50Mb version) and Illumina Next Generation sequencing. We will sequence the exon regions of genomic DNA isolated from germline (as described in section 9.2.4) and breast cancer stem cells to ~100X coverage. Following alignment of sequence data to the human genome reference sequence, we will employ a custom analysis pipeline to detect tumor specific coding mutations including point mutations and small insertion/deletions (indels). These will be reported in an annotation file that will provide reference and mutated allele, genome position, gene coding position and alleles, reference amino acid and mutated amino acid designation, and mutation quality score.

9.2.3.1.4 **Transcriptome Profiling (EXPANSION PORTION)**

Whole transcriptome profiling of both bulk tumor tissue (as described in section 9.2.4) and breast cancer stem cell populations will be attempted on patients at the MTD. This will be done in an "exploratory" manner using RNAseq technology through NGS. Total RNA will be collected from the cancer stem cell fraction and bulk tumor. RNA-seq will be carried out using the Illumina methodology and sequencing system. These studies are aimed to not only explore a possible differential molecular characteristic profile between the two cell populations, but also to determine clinical feasibility of carrying out such types of studies in a clinical trial. Although only 24 anticipated tumor evaluable sets of tissue will be characterized, the intent is to provide valuable information in three areas. First, we wish to assess if there is a correlative biomarker profile of the responders in this subset of patients. Second, the investigators wish to assess if there is a differential and characteristic profile between the tumor cell and stem cell populations. Finally, we will determine if the therapeutic interventions have differential effects on stem cells versus bulk tumor cell populations. If we can demonstrate the

feasibility of this approach, then it can potentially be used to assess the effects of any therapy on cancer stem cell versus bulk tumor cell populations.

9.2.3.2 Assessment (EXPANSION PORTION)

9.2.3.2.1 Mammosphere-forming efficiency (MSFE) will be calculated by dividing the number of mammospheres by the number of seeded live cells. % of CD44+/CD24- cells and ALDH+ cells will be assessed from the number of positive cells per total events analyzed by FACS (fluorescence activated cell sorting). The stem cell marker expression in tissues will either be quantified using a 0-3+ scoring system or if RT-PCR is done as relative expression intensity compared to a housekeeping gene. See Statistical Considerations for additional details.

9.2.3.2.2 **RAD51 Assay Methodologies (EXPANSION PORTION)**

For Rad51 foci staining, the primary antibody used in this study will be rabbit polyclonal anti-RAD51 (sc-8349, Santa Cruz) at a dilution of 1:200. The secondary antibody is Alexa-flour 488 (Invitrogen, A11008) at a dilution of 1:1000. Images will be obtained with a Leica inverted confocal microscope using a 100x oil immersion objective. Images were processed using Adobe PhotoShop (Abacus Inc). At least 100 nuclei were counted on each slide. Nuclei containing more than five RAD51 foci were classified as positive.

The percent of CD44+/CD24- cells and ALDH+ cells will be assessed from the number of positive cells per total events analyzed by FACS (fluorescence activated cell sorting). The biomarker and stem cell marker expression in tissues will be quantified by AQUA©. The absolute AQUA© score will be determined according to software algorithm as detailed in the "**AQUA© Methodology"** section (9.1.3.3.2) above. This method will also allow for assessment of nuclear β-catenin.

9.2.3.2.3 **Exome Profiling (EXPANSION PORTION)**

Exome sequencing will be performed as described above. Following alignment of sequence data to the human genome reference sequence, we will employ a custom analysis pipeline to detect tumor specific coding mutations including point mutations and small insertion/deletions (indels). These will be reported in an annotation file that will provide reference and mutated allele, genome position, gene coding position and alleles, reference amino acid and mutated amino acid designation, and mutation quality

score. Somatic analysis will be performed for the germline versus breast cancer stem cells.

9.2.3.2.4 Transcriptome Profiling (EXPANSION PORTION)

For whole transcriptome sequencing (RNAseq), we will utilize Illumina-based Next Generation Sequencing technologies to sequence libraries generated from total RNA derived from each specimen. RNA-seq reads will be mapped against the human genome and transcript reference sequences. The software packages Cufflinks and Tophat will be employed for mapping, read count generation, and junction detection (PMID: 20436464; PMID: 19289445). For differential gene expression analysis between samples based on RNAseq data, we will also utilize both the EdgeR and DESeq analysis packages (PMID: 19910308; PMID: 20979621). These technologies provide deep gene expression with increased dynamic range, allow for an analysis of differential splicing and exon usage. A weighted gene analysis will be performed to identify genes and molecular characteristics which discriminate between treatment responsive versus refractory patients, using methodologies previously described (67).

Deep genomic and transcriptomic profiling will allow us to dissect and understand the biologic complexity and variable molecular pathology operating in each tumor sample, and assist with establishment of a genomic classifier predictive of response to ABT-888/CPT-11 combination therapy. To identify possible molecular mechanisms or pathways associated with lack of response to ABT-888/CPT-11 combination therapy, tumor profiles will be examined using a systems biology approach including use of gene ontology software from Gene Go (www.Genego.com). The Gene Ontology is a controlled vocabulary describing a hierarchy of functionally annotated genes. This is a powerful tool to identify molecular signatures, characteristics and pathways, which reflect tumor pathobiology with statistical significance.

A key component to this investigation will be to perform an in depth molecular characterization of the flow cytometry-enriched breast cancer stem cell (BCSC) populations compared to the non BCBC tumor fraction from the same patient, Exome and RNA sequencing will be performed to survey the somatic coding genome and transcriptome. A comparative analysis will also be performed against heterogeneous (non-fractionated) metastatic tumor samples. Correlative studies following stratification of

treatment responsive versus refractory patients will also be performed from these profiles. This will address the contribution of the BCSC component in this patient cohort. In sum, the proposed comprehensive high resolution molecular profiling will provide tremendous insight toward identification and validation of additional tractable molecular targets in treatment-refractory patients.

Shipping to University of Michigan (EXPANSION PORTION) :
Two tissue core samples will be collected in 15 ml culture media (MEGM Lonza CC3051A) and sent by overnight express on ice/cold packs to the laboratory of Dr. [redacted] at the University of Michigan. Prior to shipping, tissue must be stored 2-8°C and must be shipped the same day (not held overnight). No shipments will occur over weekends or holidays.

Prior to any shipment, please notify [redacted] via email and/or telephone. The samples will be shipped to the following address:

Attention: [redacted]
B026 NCRC, Room [redacted]
2800 Plymouth Road
Ann Arbor, MI 48109
Office: [redacted]
Fax: [redacted]
Email: [redacted]

Timing of Assessment (EXPANSION PORTION)

Tumor collection (4-6 passes with an 18 gauge needle or a surgically removed specimen if the lesion is subcutaneous and easily accessible by a surgeon) should occur at three times:

- First biopsy: within 2 weeks prior to treatment on Day 1
- Second biopsy: 4-6 hours after the start of infusion of irinotecan (Day 1, Cycle 1)
- Third Biopsy: 4-6 hours after the combination of ABT-888 and start of infusion of irinotecan (Day 8, Cycle 1). The second daily dose of ABT-888 scheduled on this day will occur following the biopsy.

9.2.4 Next Generation Molecular Profiling (Expansion Portion)

9.2.4.1 Outcome measure (EXPANSION PORTION)

In addition to the stem cell population, TGen will sequence the coding genome and transcriptome from both normal and tumor tissue from each study patient in the two arms of the study using a three-pronged Next Generation Sequencing (NGS) approach. First, we will sequence exomes for both tumor and normal at roughly 100X coverage, allowing for sufficient read level power to detect point mutations

(single nucleotide substitutions or small insertion/deletions) even in the face of high tissue heterogeneity (i.e. low tumor content). Second, we will sequence whole genomes of both tumor and normal using a long-insert library (>1.5 kb) at low coverage (~5X-10X). The large inserts will provide high 'clonal' or 'physical coverage' (>100X) to detect structural changes and copy number events occurring between paired or mated reads, recognizing base-level coverage will be approximately 8X outside exonic regions. Third, we will resequence RNA from the tumor and a matched normal tissue. This will allow for robust expression analysis and will provide a mechanism to interpret the consequence of a mutation. For example, our preliminary data shows three examples where a translocation, a moderate deletion, and a small deletion all impact expression of key genes (Figure 5). Additionally, this powerful and comprehensive genome and transcriptome approach delivers a comprehensive list of changes, that give way to full understanding of the pathway perturbations that occur in a tumor through multiple mechanisms further powering our abilities to detect therapeutically relevant concepts associated with drug sensitivity or resistance.

At the tumor somatic level, this strategy will impart the opportunity to detect: 1) somatic point mutations and small *indels;* 2) somatic copy number changes (amplifications and deletions); and 3) large *cis* rearrangements and translocations. Also at the tumor level, gene expression analysis by array-based technology gives way to RNA-seq with a far wider dynamic range, at the gene, exon, and in some cases allele-specific levels. At the constitutional plane, our strategy will also provide a profile for germline variants within highly penetrant cancer genes such as *BRCA1/2*, *TP53*, *CHK2*, or other genes defined by the Human Genome Mutation Database. Furthermore, we will have access to germline variants associated with steroid and drug metabolism (i.e., cytochrome P450, UDP-glucuronosyltransferase, N-acetyltransferases, *GSTP1*, *ABCB1*, *XRCC1*, *ERCC2*, and *UGT1A1*). Although not a direct focus of this study, we will generate data for the vast majority of germline variants for each individual within the study.

9.2.4.2 **Assessment (EXPANSION PORTION)**

To support these activities, TGen will leverage its NGS technology platform which includes seven Life Technologies SOLiD 4 systems, four Illumina HiSeq2000 systems, two Illumina GAII systems, and one Roche 454 Jr. system. Furthermore, data processing and analysis will be supported by a 5500 parallel processor supercomputing environment and an informatics staff that are participants in the NIH 1000 Genomes initiative. This informatics team has developed custom pipelines for the discovery of somatic alterations from paired normal/tumor Exome-seq data, as well as RNA-seq data.

9.2.4.3 Collection and processing (EXPANSION PORTION)

The following samples will be collected:

- 1-2 acid citrate dextrose (ACD) yellow top collection tubes with 10-20mls of peripheral blood using standard blood collection techniques. Ship overnight (Mon-Thur) with an ice pack.
- One snap frozen 18 gauge needle biopsy(s) (1cm in length) from tumor. Core biopsies to be sent to TGen will be collected and immediately cryopreserved in separate tubes in liquid nitrogen until further processed. Surgical tumor tissue should be collected in pre-cooled cryovials and immediately flash frozen in liquid nitrogen. Store at -80°C until ready to ship. Ship overnight (Mon-Thur) on dry ice.

9.2.4.4 Shipping for PD samples (EXPANSION PORTION):

One 18-guage core tissue samples will be flash frozen and shipped overnight on dry ice to the laboratory of Dr. [redacted] at TGen. 1-2 acid citrate dextrose (ACD) yellow top collection tubes with 10-20mls of peripheral blood will be shipped overnight with an ice pack. Received samples will be maintained at -80°C until processing. No shipments will occur on over weekends or holidays. Prior to any shipment, please notify [redacted] in Dr. [redacted] lab via email and/or telephone. The samples will be shipped to the following address:

[redacted]
Translational Genomics Research Institute (TGen)
445 N. Fifth Street
Phoenix, Arizona 85004
Phone: [redacted]
Email: [redacted]

9.2.5 Tissue Sample Priority (Expansion portion)

Fresh Tumor tissue will be triaged as specified:

1.) First core biopsy for gamma-H2AX/ERCC1 (NCTVL)
2.) Second core biopsy: stem cell analyses (Dr. [redacted] University of Michigan)
3.) Third core biopsy: PD biomarker analysis (Dr. [redacted] University of Michigan)
4.) Fourth core biopsy for genomic analysis of tumor (Dr. [redacted] TGen)

9.2.6 Archival Tissue – AbbVie (Expansion Portion)

9.2.6.1 Tissue Collection for IHC and FISH DNA Mutational/Methylation Analysis

If available, fixed samples from most recent pathological analysis will be collected from subjects who have not refused consent. Immunohistochemistry (IHC), tumor DNA methylation and mutational analysis, fluorescence in situ hybridization (FISH) and/or quantitative polymerase chain reaction (qPCR) may be performed on tissue slides from archived, diagnostic, formalin fixed, paraffin embedded (FFPE) tissue blocks from all subjects who consent in the study.

The site may either submit an archived tissue block or cut tissue section on slides. From each representative formalin fixed paraffin embedded tumor tissue, the local pathology laboratory should apply 10 slices of tissue with a thickness of approximately 10 microns and 10 slices of tissue with a thickness of approximately 4 to 6 microns to positively charged slides to be used for IHC, mutational and FISH analysis. Therefore, a minimum of 20 slices of tissue sections should be collected from each subject block. In cases where there is not enough appropriate tissue available to provide these sections, the investigator will communicate with the pathology laboratory to determine the maximum number of slides that can be provided.

To ensure optimal sampling, two quality control slides must also be prepared by the pathology laboratory and included in the shipment of slides to designated lab. These quality control slides will be representative of the beginning and of the end of the tissue section. These slides are to be stained using Hematoxylin and Eosin (H&E) and reviewed by the local pathologist to ensure the diagnostic quality of viable tumor and normal cells (i.e., large regions of necrosis or areas composed primarily of fibrous connective tissue or adipose tissue are not the predominant feature). The remaining tissue prepared for the unstained slides will be procured from the sections closest to the section that is of adequate diagnostic quality.

Included with each shipment should be a copy of the pathology report with all specific subject identification information removed/blacked out and a completed shipment inventory form. The FFPE tissue block or slide boxes should be labeled with study drug number, sample matrix (tissue), protocol number, subject number and collection date. Slide boxes should be packaged using suitable shipping materials and sent to the designated lab at ambient temperature. If the blocks are to be returned, please provide clear instructions and the return address.

9.2.6.2 Assay Description (EXPANSION PORTION):

9.2.6.2.1 Stratification Markers

Genetic amplification, chromosomal loss and/or mutational status of various genes, including but not limited to those in the DNA repair pathway represent genetic lesions potentially associated with subject outcome. Gene and protein expression, FISH and/or mutational.methylation analysis may be conducted on tissue from archived biopsy tumor samples from subjects participating in this study to assess modifications which may prove to be informative. The potential relationship between amplification/loss/mutation of these entities and the clinical outcome in these subjects may be examined as a patient stratification tool.

DNA methylation regulates gene expression (inactivates certain genes) and aberrant methylation of specific genes

is associated with cancer development and poor clinical outcome. Tumor-derived DNA may be examined for variable methylation status of genes that are known to have prognostic implications associated with subjects enrolled in this trial. The list of potential genes includes, but is not restricted to: RASSF1A, CDH1, Cyclin D2 and TWIST.

9.2.6.2.2 Protein RNA and DNA Analysis (EXPANSION PORTION)

Tissue slides from diagnostic biopsies, may be used to assess molecular characteristics and/or expression of protein, nucleic acids and metabolites. Additionally, these samples may be examined for mutations and/or methylation of nucleic acids. Protein analysis of relevant proteins, including but not limited to DNA repair proteins such as ERCC1 and XPF, may be performed on sources of tumor tissue obtained from each consented subject when feasible. Mutational analysis of tumor-derived DNA may include, but is not limited to acquired secondary mutations in the BRCA genes that have been reported to restore the activity of the molecule. These analyses might reveal putative stratification and/or resistance markers for correlation with efficacy.

9.2.6.3 Shipping (EXPANSION PORTION):

Archival tissue samples will be shipped to the address below. An email should be sent to AbbVie Sample Receiving and [redacted] upon shipping any samples. Any supporting documents, i.e. tracker of any samples being shipped, and specify study number should be included - this will make it easier to identify the samples being received at the depot.

AbbVie Sample Receiving<br>Dept R43F, Bldg AP13A, Room 2310<br>c/o: Delivery Services<br>1150 S. Northpoint Blvd<br>Waukegan, IL 60085
(847) 937-0889
Sample.receiving@abbvie.com
[redacted]

9.3 Laboratory Correlative Studies (DOSE ESCALATION FOR INTERMITTENT ABT-888 PORTION)

For the intermittent dose escalation portion of this study, blood and fresh tissue will be collected as described in sections below.

If the availability of fresh tissue is very limited, a triaging system will be applied (see section 9.3.6).

NOTE:
When a patient has been enrolled and a biopsy date set, please notify the following people so that they can reserve time for their research equipment:

[redacted]

9.3.1 Assay Descriptions (DOSE ESCALATION FOR INTERMITTENT ABT-888 PORTION)

9.3.1.1 Development of a PARP 1 and 2 Trapping Assay (DOSE ESCALATION FOR INTERMITTENT ABT-888 PORTION)

The Trapping, PARP 1 & 2 assay, and additional biomarker testing to help better define PARP inhibition and Trapping are being funded, in part, by AbbVie Pharmaceuticals. Work funded by AbbVie is being conducted in the laboratory of Dr. [redacted]. The relevant work is described in the paragraph(s) below.

As described in section 2.4.7, recent research suggests that the ability of PARP1 and 2 to be trapped in the chromatin fraction of cells after treatment with a PARP inhibitor correlates with cytoxicity (53). The goal of our exploratory studies is to develop assays to detect trapping of PARP1 and 2 in tumor biopsy tissue in response to treatment with Irinotecan plus a PARP inhibitor, in this case ABT-888. We will work on the development of three assays and these include the subcellular fractionation assay, the FANCD2 monoubiquitination assay, and the STORM assay for PARP trapping.

9.3.1.2 Additional Assay (DOSE ESCALATION FOR INTERMITTENT ABT-888 PORTION)

We will bank tissue in Dr. [redacted] laboratory at Yale University for work on an assay to be named later.

9.3.2 Assessment (DOSE ESCALATION FOR INTERMITTENT ABT-888 PORTION)

9.3.2.1 Timing of Assessment (DOSE ESCALATION FOR INTERMITTENT ABT-888 PORTION)

Tumor collection (4-6 passes with an 18 gauge needle or a surgically removed specimen if the lesion is subcutaneous and easily accessible by a surgeon) should occur at three times:

- First biopsy: within 2 weeks prior to treatment

- Second biopsy: within 4 hours after the day -11 ABT-888 morning dose (during ABT-888 run-in period)
- Third biopsy: within 4 hours of ABT-888 on Cycle 1 Day 11

9.3.3 Future Studies (DOSE ESCALATION FOR INTERMITTENT ABT-888 PORTION)

Blood and tissue will be collected and processed as described in section 9.3.3.1. These samples will be stored in the laboratory of Dr. [redacted] of Yale School of Medicine for future studies.

9.3.3.1 **Collection and processing (DOSE ESCALATION FOR INTERMITTENT ABT-888 PORTION)**

The following samples will be collected at baseline only (biopsy #1):

- 1-2 acid citrate dextrose (ACD) yellow top collection tubes with 10-20mls of peripheral blood using standard blood collection techniques. Ship overnight (Mon-Thur) with an ice pack.
- Two snap frozen 18 gauge needle biopsy(s) (1cm in length) from tumor. Core biopsies to be sent to Yale University's Clinical Research Support Lab (CRSL) will be collected and immediately cryopreserved in separate tubes in liquid nitrogen until further processed. Surgical tumor tissue should be collected in pre-cooled cryovials and immediately flash frozen in liquid nitrogen. Store at -80°C until ready to ship. Ship overnight (Mon-Thur) on dry ice.

9.3.3.2 **Shipping for Future Studies (DOSE ESCALATION FOR INTERMITTENT ABT-888 PORTION)**

Blood: 1-2 acid citrate dextrose (ACD) yellow top collection tubes with 10-20mls of peripheral blood will be shipped overnight with an ice pack to the Laboratory of Dr. [redacted]. Received samples will be maintained at -80°C until processing. No shipments will occur on over weekends or holidays. Prior to any shipment, please notify Dr. [redacted] via email and/or telephone. The samples and shipping manifest in Appendix K will be shipped to the following address:

Laboratory of Dr. [redacted]
310 Cedar St. BML113, Department of Pathology
Yale School of Medicine
New Haven, CT 06520-8023
Phone: [redacted]
Email: [redacted]

Tissue: Two 18-guage core tissue samples will be flash frozen and shipped overnight on dry ice to the Yale University's Clinical Research Support Lab (CRSL). Received samples will be maintained at -80˚C until processing. No shipments will occur on over weekends or holidays. Prior to any shipment, please notify [redacted] in the CRSL and [redacted] via email and/or telephone. The samples and shipping manifest in Appendix L will be shipped to the following address:

Clinical Research Support Lab
Yale University
Smilow Cancer Hospital
35 Park St, room [redacted]
Attn: [redacted]
New Haven, CT 06510
Phone: [redacted]
Email: [redacted]

9.3.4 PARP 1 and 2 Trapping (DOSE ESCALATION FOR INTERMITTENT ABT-888 PORTION)

9.3.4.1 The subcellular fractionation assay (DOSE ESCALATION FOR INTERMITTENT ABT-888 PORTION)

This is based upon previous studies using the DU-145 prostate cancer cell line (53), in which cells were treated with various PARP inhibitors, lysed, fractionated, and western-blotted with antisera raised against PARP1 and PARP2. Our challenge is to expand this type of assay to tumor biopsy tissue. Our team has experience in lysis and cellular fractionation of tumor tissue, and also in western blotting. Our first goal is to import the subcellular fractionation assay to our laboratory. We have DU-145 cells. These cells will be grown and treated with different concentrations of various PARP inhibitors including ABT-888 and olaparib, lysed, and subjected to cellular fractionation into nuclear and chromatin as described (53), based upon incubation with increasing salt concentrations. The fractions will be resolved by polyacrylamide gel electrophoresis (PAGE), blotted to nitrocellulose, and incubated with antisera raised against PARP1, PARP2, TOP1 (to mark the soluble nuclear fraction), histone H3 (to mark the chromatin fraction), and γ -tubulin as a loading control. Quantification will be as we describe (54), by normalization to γ-tubulin. We expect to repeat the results of the original assay (53) and demonstrate dose-dependent PARP1 and 2 trapping in the chromatin fraction.

Our second experiment will be a repeat of the above experiment but also include irinotecan, as this agent is used in the protocol. We expect to observe similar levels of PARP1 and 2 trapping as in the earlier experiment but realize that we may observe greater or lesser amounts of trapping upon addition of this drug.

Our third set of experiments will be with human triple negative breast tumor xenografts, harvested from untreated mice or mice treated with irinotecan alone or in combination with ABT-888, using doses and conditions that mimic the ones used in the clinical protocol. At least three tumors from each treatment, in addition to negative controls, will be homogenized and this lysate will be strained to separate the cells from the debris and fractionated using the Thermo Scientific Subcellular Fractionation Kit for Tissue. We have successfully fractionated cells from tissues using this kit. After fractionation, the samples will be processed as described above. We expect to observe PARP1 and 2 trapping in chromatin at levels similar to what is observed in cells treated with ABT-888. Optimization may be necessary to achieve

these results and could include homogenization conditions, incubation and centrifugation times.

Once an optimized protocol is in place, we will initiate studies with core biopsy tissue. Two core biopsies will provide sufficient numbers of cells to perform up to 6 PARP trapping assays. However, we expect to require only three independent assays to reliably quantify trapping of PARP 1 and 2 in response to treatment with irinotecan and irinotecan plus ABT-888.

9.3.4.2 The FANCD2 monoubiquitination assay (DOSE ESCALATION FOR INTERMITTENT ABT-888 PORTION)

Monoubiquitination of FANCD2 occurs in response to replication stress (55). Interestingly, cancer cells in which PARP1 is trapped in chromatin after treatment with various PARP inhibitors have been shown to have significantly increased levels of FANCD2 monoubiquitination in nuclear fractions (53). Therefore, this could serve as a biomarker for PARP trapping. In order to test this hypothesis, we will resolve aliquots of the of cytoplasmic and nuclear fractions with appropriate markers, as described above, on PAGE gels and immunoblot with antisera raised against FANC2, which can detect its monoubiquitinated form. Quantification of monoubiquitinated FANCD2 after normalization to tubulin in three replicate experiments will be performed. Statistics, using GraphPad Prism, will be performed to assess correlation between levels of PARP trapping and FANCD2 monubiquitination. A linear regression model that supports R2 for goodness of fit and a P value for the slope of the line being significantly different from zero will be used to assess a potential correlation. If a strong correlation exists it will be consistent with the idea that levels of monoubiquitinated FANCD2 correlate with levels of PARP trapping in chromatin. We will correlate with patient outcomes.

9.3.4.3 The STORM assay (DOSE ESCALATION FOR INTERMITTENT ABT-888 PORTION)

This constitutes that most exploratory aspect of this proposal. Stochastic optical reconstruction microscopy (STORM) is a super-resolution technique for imaging of single molecules within cells or tissues. This novel imaging technique has successfully detected clusters of RNA polymerase molecules within chromatin (56). In collaboration with Dr. David Warshaw, University of Vermont, and a world renowned expert in STORM imaging, we will develop this technique to image clusters of PARP1 molecules, using standard techniques (56). Chromatin will be visualized by immunostaining using antibodies against either H2B or linker H1 or both. PARP1 will also be visualized by immunostaining. Initially, conditions will include cells treated or not with PARP inhibitor. The distribution of chromatin width is likely to reflect PARP1 clustering such that trapping may result in either aberrantly tight or lose (more open) chromatin versus no trapping. Once these endpoints are established, we will image chromatin from tumor samples and correlate our quantitative measurements of chromatin width with PARP1 trapping levels in the fractionation assay of the same tissue. Our ultimate goal is to develop a relatively easy and efficient imaging assay to evaluate the ability of PARP inhibitors to trap PARP1 in tumor tissue.

9.3.4.4 Collection and processing for PARP (DOSE ESCALATION FOR INTERMITTENT ABT-888 PORTION)

The following samples will be collected at baseline (biopsy #1):

- Two snap frozen 18 gauge needle biopsy(s) (1cm in length) from tumor. Core biopsies should be collected in pre-labeled and pre-cooled cryovials and immediately flash frozen in liquid nitrogen. Surgical tumor tissue should be collected in pre-cooled cryovials and immediately flash frozen in liquid nitrogen. Store at -80°C until ready to ship. Ship overnight (Mon-Thur) on dry ice.

  The labeling should be done with VWR freezer-specific markers or Tough Tags. Tubes should containing the following information on the label:

  NCI 7977
  Subject ID number
  Collection Date
  Study Time Point (BL/RI/C1D11)
  (BL=baseline; RI=run in; C1D11 = cycle 1 day 11)
  Core number

The following samples will be collected after treatment with study drug(s) at Run-In Day # -11 (biopsy #2) and Cycle 1 Day 11 (biopsy #3):

- 1-3 snap frozen 18 gauge needle biopsy(s) (1cm in length) from tumor. Core biopsies should be collected in pre-labeled and pre-cooled cryovials and immediately flash frozen in liquid nitrogen. Surgical tumor tissue should be collected in pre-cooled cryovials and immediately flash frozen in liquid nitrogen. Store at -80°C until ready to ship. Ship overnight (Mon-Thur) on dry ice.

  The labeling should be done with VWR freezer-specific markers or Tough Tags. Tubes should containing the following information on the label:

  NCI 7977
  Subject ID number
  Collection Date
  Study Time Point (BL/RI/C1D11)
  (BL=baseline; RI=run in; C1D11 = cycle 1 day 11)
  Core number

  Note: 2 cores will be collected at BL; 3 cores collected at RI; 3 cores at C1D11

  Catalog numbers are provided for **information only**. These specific item numbers **DO NOT** have to be used.

  - Cryogenic 2 ml vials: Fisher cat # 12-567-501
  - Cryolabels for 2 ml tubes: Fisher cat# 15-920-D
  - Cryogenic marker Fisher Cat # 11-679-117 (Thermo Scientific # 4000222)
  - Moisture-proof permanent marker: VWR cat# 52877-310

9.3.4.5 Shipping for PARP samples (DOSE ESCALATION FOR INTERMITTENT ABT-888 PORTION)

At baseline (biopsy #1), two snap frozen 18 gauge needle biopsy(s) (1cm in length) from tumor. Core biopsies should be collected in pre-labeled and pre-cooled cryovials and immediately flash frozen in liquid nitrogen. Surgical tumor tissue should be collected in pre-cooled cryovials and immediately flash frozen in liquid nitrogen. Store at -80°C until ready to ship. Ship overnight (Mon-Thur) on dry ice.

At the time of on-treatment samples (biopsies #2 and #3), one to three snap frozen 18 gauge needle biopsy(s) (1cm in length) from tumor. Core biopsies should be collected in pre-labeled and pre-cooled cryovials and immediately flash frozen in liquid nitrogen. Surgical tumor tissue should be collected in pre-cooled cryovials and immediately flash frozen in liquid nitrogen. Store at -80°C until ready to ship. Ship overnight (Mon-Thur) on dry ice.
No shipments will occur on over weekends or holidays. Prior to any shipment, please notify Dr. [redacted] via email and/or telephone. The samples and shipping manifest in Appendix L will be shipped to the following address:

Clinical Research Support Lab
Yale University
Smilow Cancer Hospital
35 Park St, room [redacted]
Attn: [redacted]
New Haven, CT 06510
Phone: [redacted]

Email: [redacted]

9.3.5 Samples Collection and Handing Additional Assay (DOSE ESCALATION FOR INTERMITTENT ABT-888 PORTION)

All accessioned tissue specimens should be handled according to the College of American Pathologists' (CAP) guidelines, recording all steps related to acquisition, processing and transfer to create a detailed and accurate record of sample provenance. Tissue specimens will be stored in Sarstedt 2 mL cryovials, Tissue-Tek cryomolds or plastic cassettes, as appropriate, labeled with 2-dimensional barcodes on -190 °C-robust adhesive labels (Brady LabXpert) and stored in Taylor-Wharton LABS-40K liquid-nitrogen freezers except for formalin-fixed, paraffin-embedded tissue samples which are maintained at 22 °C.
We plan to have the one single pre-treatment biopsy and one core after each treatment modality to be fixed in formalin and stored in paraffin for subsequent biomarker analyses. The second core in each treatment arm will be snap-frozen in optimal cutting temperature (OCT) medium-filled cryomold. To minimize cold ischemic time, we recommend a processing time of no more than 10 minutes from tissue acquisition.

9.3.5.1 Shipping Additional Assay (DOSE ESCALATION FOR INTERMITTENT ABT-888 PORTION)

At baseline (biopsy #1), one core sample will be collected and sent to Yale University's Clinical Research Support Lab (CRSL) for distribution to Dr. [redacted] laboratory. At time of biopsy #2 and #3, one to two cores (any remaining tissue) will be collected and sent to Yale University's Clinical Research Support Lab (CRSL) for distribution to Dr. [redacted] laboratory.
All accessioned tissue specimens should be handled according to the College of American Pathologists' (CAP) guidelines, recording all steps related to acquisition, processing and transfer to create a detailed and accurate record of sample provenance. Tissue specimens will be stored in Sarstedt 2 mL cryovials, Tissue-Tek cryomolds or plastic cassettes, as appropriate, labeled with 2-dimensional barcodes on -190 °C-robust adhesive labels (Brady LabXpert) and stored in Taylor-Wharton LABS-40K liquid-nitrogen freezers except for formalin-fixed, paraffin-embedded tissue samples which are maintained at 22 °C.
We plan to have the one single pre-treatment biopsy and one core after each treatment modality to be fixed in formalin and stored in paraffin for subsequent biomarker analyses. The second core in each treatment arm will be snap-frozen in optimal cutting temperature (OCT) medium-filled cryomold. To minimize cold ischemic time, we recommend a processing time of no more than 10 minutes from tissue acquisition.

Frozen tissue and the shipping manifest in Appendix L will be shipped to Yale University's Clinical Research Support Lab (CRSL).

No shipments will occur on over weekends or holidays. Prior to any shipment, please notify [redacted] via email. The frozen tissue samples and the shipping manifest in Appendix L will be shipped to the following address:

Clinical Research Support Lab
Yale University
Smilow Cancer Hospital
35 Park St, room [redacted]
Attn: [redacted]
New Haven, CT 06510
Phone: [redacted]

Email [redacted]

Formalin-fixed, paraffin-embedded tissue samples and the shipping manifest in Appendix M will be shipped in attention to [redacted] at [redacted] laboratory at Yale University. No shipments will occur on over weekends or holidays. Prior to any shipment, please notify [redacted] via email. The samples will be shipped to the following address:

Attention: [redacted]
[redacted] Lab, Department of Pathology

Yale University
310 Cedar Street, BML112
New Haven, CT 06520-8023
Email: [redacted]

9.3.6 Tissue Sample Priority (DOSE ESCALATION FOR INTERMITTENT ABT-888 PORTION)

Baseline (biopsy #1) fresh tumor tissue will be triaged as specified:

1.) First and second core biopsies for PARP trapping analysis (Dr. [redacted] Yale University)
2.) Third and fourth core biopsies (Dr. [redacted], Yale University)
3.) Fifth core for future research (Dr. [redacted] Yale University)

On-treatment samples (biopsies #2 and #3) fresh tumor tissue will be triaged as specified:

1.) 1-3 core biopsies for PARP trapping analysis (Dr. [redacted] Yale University)
2.) 1-2 core biopsies (any remaining tissue) to be stored for future research (Dr. [redacted] Yale University)

10. STUDY CALENDAR

Baseline evaluations are to be conducted within 1 week prior to start of protocol therapy. Scans and x-rays must be done ≤ 4 weeks prior to the start of therapy. In the event that the patient's condition is deteriorating, lab evals should be repeated within 48 hr prior to initiation of the next cycle of therapy.

10.1 Weekly Calendar (Original Dose Escalation Portion)

	Pre-Study	Wk 1	Wk 2	Wk 3	Wk 4	Wk 5	Wk 6	Wk 7	Wk 8	Wk 9	Wk 10	Wk 11	Wk 12	Off Study[8]
ABT-888		X[1]	X[2]		X[3]	X[2]		X[4]	X[2]		X[4]	X[2]		
Irinotecan (CPT-11)[5]		X	X		X	X		X	X		X	X		
Informed consent	X													
Archival tissue collected	X													
Demographics	X													
Medical history	X													
Concurrent meds	X	X--X												
Physical exam	X	X	X	X	X	X		X	X		X	X		X
Vital signs	X	X	X	X	X	X		X	X		X	X		X
Height	X													
Weight	X	X	X		X	X		X	X		X	X		X
Performance status	X	X	X	X	X	X	X	X	X	X	X	X	X	X
CBC w/diff, plts	X	X	X	X	X	X	X	X	X	X	X	X	X	X
Serum chemistry[6]	X	X	X	X	X	X	X	X	X	X	X	X	X	X
EKG[15]	X	X		X										
AE evaluation		X--X												X
Tumor measurements	X	Tumor measurements are repeated every 6 weeks. Documentation (radiologic) must be provided for patients removed from study for PD. Confirmatory scans will also be obtained 6 weeks following initial documentation of an objective response (CR or PR).												X[8]
Radiologic evaluation	X	Radiologic measurements should be performed every 6 weeks. Confirmatory scans will also be obtained 6 weeks following initial documentation of an objective (complete or partial) response.												X[8]
β-HCG	X[7]													
UGT1A1 status	X[16]													
Blood draw -PK ,PG,PD	X[14]	X[9]	X[10]		X[11]	X[12]								
Biopsy (PD)		X[13]	X[13]											
Participant diary given		X			X			X			X			

1: ABT-888: Dose as assigned; Twice daily oral administration begins Day 3 of 21-day cycle.
2: ABT-888: Dose as assigned; Twice daily oral administration continues through Day 14 of 21-day cycle.
3: ABT-888: Dose as assigned; Once daily oral administration occurs Day -1 of 21-day cycle, twice daily oral administration begins Day 1 of 21-day cycle
4: ABT-888: Dose as assigned; Twice daily oral administration begins Day -1 of 21-day cycle.
5: Irinotecan: Dose as assigned; administered on days 1 and 8 of each cycle.
6: Albumin, alkaline phosphatase, total bilirubin, bicarbonate, BUN, calcium, chloride, creatinine, glucose, LDH, phosphorus, potassium, total protein, SGOT [AST], SGPT [ALT], sodium.
7: Serum pregnancy test (women of childbearing potential).
8: Off-study evaluation.
9: Blood for PK collected at each of the following beginning Cycle 1, day 1: pre-infusion (8ml), 30 min after the start of infusion (4ml), immediately at the end of infusion (EOI) (~89 min, 4ml), and 2h (4ml), 3.5h (8ml), 5.5h (8ml), 8.5h (8 ml), 28h (8ml), and 48h (8ml) after the start of infusion. PBMCs from the 3.5h, 8.5h, and 48h samples will be separated from plasma for use in PAR assay. PBMCs from the preinfusion, 5.5h, and 28h samples will be separated from plasma for PD assays. Separate 1x8 mL blood for PAR assay will be drawn at each of the following: pre-infusion, and 5.5h,and 28h after start of infusion
10: 1x8 mL blood for PAR assay at each of the following: pre-infusion, and 3.5, 5.5, 8.5, 28, and 48h after start of infusion
11: Blood for PK collected at each of the following beginning Cycle 2, Day -1: pre-dosing (8ml), 0.5 (4ml), 1 (4ml), 1.5 (4ml), 3.5 (4ml), 5.5 (8ml), 8.5 (4ml), 10 (4ml), and 28 h (8ml) after the dosing. PBMCs from the pre-dosing sample will be separated from plasma for use in PAR assay. PBMCs from the 5.5h, and 28h samples will be separated from plasma for PD assays.
12: 1x8 mL of blood for PK collected at each of the following beginning Cycle 2, Day 8: pre-infusion (8ml), 30 min (4ml) and 1 hour (4ml) after the infusion begins, immediately end of infusion (EOI) (~89 min, 4ml)), and 2h (4ml), 3.5h (4ml), 5.5h (8ml),

8.5h (4ml), 10h (4 ml, just prior to the administration of the second dose of ABT-888 on day 8), 28h (8 ml, prior to ABT-888 administration on day 9), and 48 h (4 ml, prior to ABT-888 administration on day 10) after the start of infusion. First dose of ABT-888 administered on day 8 will be taken at the start of CPT-11 infusion, so PK sampling can be lined up for both drugs. The blood samples drawn on Day 9 of Cycle 2 will occur 28 hours after start of the combination of ABT-888 and chemotherapy on Day 8 of Cycle 2, and 4 hrs after the first Cycle 2 Day 9 dose of ABT-888. PBMCs from the preinfusion, 5.5h, and 28h samples will be separated from plasma for PAR assays.

13: Biopsy to occur 28 hours after beginning of infusion on the following days: Day 2, Cycle 1 and Day 9, Cycle 1

14: Single 10 ml blood draw to determine UGT1A1 polymorphisms to be sent to KCI Pharmacology Core Laboratory

15: EKGs to occur prior to treatment (baseline) and after first dose of ABT-888 on both cycle 1, day 3 and cycle 1, day 14.

16: Standard lab test to determine absence of UGT1A1*28 allele. Only homozygous individuals will be excluded from eligibility

10.2 Daily Calendar (Cycle 1) Original Dose Escalation Portion

Studies & Tests	Pre	Cycle 1										
		Day 1	Day 2	Day 3	Day 4-7	Day 8	Day 9	Day 10	Day 11-13	Day 14	Day 15-20	Day 21[12]
Informed Consent	X											
Archival tissue collected	X											
Demographics	X											
Medical History	X											
Concurrent Meds	X	X				X				X		X
Vital Signs	X	X				X				X		X
Height	X											
Weight	X	X				X				X		X
CBC with differential;	X	X				X				X		
Serum chemistry [1]	X	X				X				X		
EKG	X			X						X		
AE Evaluation		X	X	X		X	X	X		X		X
Tumor Measurements	X											
Radiologic Evaluation	X											
β-HCG [2]	X											
UGT1A1	X[13]											
Physical and ECOG PS	X	X				X				X		
Irinotecan infusion		X				X						
ABT-888 given (po BID)				X	X	X	X	X	X	X		X[3]
Blood Draw - PK		X[4]	X[6]	X[8]								X[11]
Blood Draw - PAR		X[5]	X[7]			X[5]	X[7]	X[9]				
Blood Draw - PG	X[14]											
Participant Diary Given				X								X
Tumor collection			X[10]				X[10]					

1 Albumin, alkaline phosphatase, total bilirubin, bicarbonate, BUN, calcium, chloride, creatinine, glucose, LDH, phosphorus, potassium, total protein, SGOT [AST], SGPT [ALT], sodium.
2 Within 2 weeks of first day of study for women of childbearing potential
3 Single dose of ABT-888 only on Day 21, taken in clinic
4 Blood for PK collected at each of the following: pre-infusion (8ml), 30 min after the start of infusion (4ml), immediately at the end of infusion (EOI) (~89 min, 4ml), and 2h (4ml), 3.5h (8ml), 5.5h (8ml), 8.5h (8ml) after the start of infusion. PBMCs will be separated from plasma by Ficoll gradient at the following times for PD: pre-infusion and 5.5 hours after the start of infusion. PBMCs will be separated from plasma by Ficoll gradient at the following times for PAR assay: 3.5 hours and 8.5 hours after the start of infusion.
5 1x8 mL blood for PAR assay at each of the following: pre-infusion, and 5.5h after start of infusion
6 1x8 mL of blood for PK collected at 28 h from time of beginning of infusion. Blood sample will be separated by ficoll gradient for PD
7 1x8 mL of blood for PAR assay collected at 28 h from time of beginning of infusion.
8 1x8 mL of blood for PK collected at 48 h after beginning of infusion. Blood sample will be separated by ficoll gradient for PAR assay
9 1x8 mL of blood for PAR assay collected at 48 h after beginning of infusion
10 Tumor biopsies to occur 28 h from time of beginning of previous day's infusion. Day 9 biopsy to occur 4 hours after first dose of Day 9, Cycle 1 ABT-888 treatment
11 Blood for PK collected at each of the following: pre-ABT-888 dosing (8ml), 30 min after dosing (4ml), and 1h (4ml), 1.5h (4ml), 3.5h (4ml), 5.5h (8ml), 8.5h (4ml), and 10 h (4ml) after dosing. Blood drawn at pre-dosing will be

separated by Ficoll gradient for PAR assays. Blood drawn at 5.5 hours after the start of dosing will be separated by Ficoll gradient for PD assays

[12] Cycle 1,Day 21 is equivalent to Cycle 2, Day -1

[13] Standard lab test to determine absence of UGT1A1*28 allele. Homozygous patients will be excluded from study

[14] 10ml blood draw anytime prior to study registration for pharmacogenomic (PG) analysis to determine polymorphisms in UGT1A1, CYP2C9, CYP2C19, & ABCG2 to be sent to KCI Pharmacology Core Lab

10.3 Daily Calendar (Subsequent Cycles) – Original Dose Escalation Portion

Studies & Tests	Subsequent cycles									
	Day -1[1]	Day 1	Day 2-7	Day 8	Day 9	Day 10	Day 11-13	Day 14	Day 15-20	Day 21
Concurrent Meds	X			X				X		X
Vital Signs	X			X						X
Weight	X			X						X
CBC with differential; plts	X			X				X		X
Serum chemistry [1]	X			X				X		X
AE Evaluation	X	X		X	X	X		X		X
Tumor Measurements	Tumor measurements are repeated every 6 weeks. Documentation (radiologic) must be provided for patients removed from study for PD. Confirmatory scans will also be obtained 6 weeks following initial documentation of an objective response (CR or PR).									
Radiologic Evaluation	Radiologic measurements should be performed every 6 weeks. Confirmatory scans will also be obtained 6 weeks following initial documentation of an objective (complete or partial) response.									
Physical and ECOG PS	X			X						X
Irinotecan infusion		X		X						
ABT-888 given (po BID)	X[2]	X	X	X[8]	X	X	X	X		X
Blood Draw - PK	X[3]	X[4]		X[5]	X[6]	X[7]				
Participant Diary Given	X									X

[1] Day -1 is equivalent to Day 21 of previous cycle

[2] Cycle 2 only: Single dose of ABT-888 on Day -1, subsequent cycles have full, twice-daily dose

[3] Cycle 2 only: Blood for PK collected at each of the following: pre-ABT-888 dosing (8ml), 30 min after dosing (4ml), and 1h (4ml), 1.5h (4ml), 3.5h (4ml), 5.5h (8ml), 8.5h (4ml), and 10 h (4ml) after dosing. Blood drawn at pre-dosing will be separated by Ficoll gradient for PAR assays. Blood drawn at 5.5 hr after dosing will be separated by Ficoll gradient for PD assays.

[4] Cycle 2 only: 1x8 mL of blood for PK collected at 28 h from time of Day -1 ABT-888 dosing. Blood sample will be separated by ficoll gradient for PD

[5] Cycle 2 only: Blood for PK collected at each of the following: pre-infusion/ABT-888 dosing (8ml), 30 min (4ml) and 1 hr (4ml) after start of infusion/dosing, immediately at the end of infusion (EOI) (~89 min, 4ml), and 2h (4ml), 3.5h (4ml), 5.5h (8ml), 8.5h (4ml), and 10 h (4ml) after start of infusion/dosing. Blood drawn at pre-infusion/dosing and 5.5 hours after the start of infusion/dosing will be separated by ficoll gradient for PAR assays

[6] Cycle 2 only: 1x8 mL of blood for PK collected at 28 h from time of Day 8 infusion/ABT-888 dosing. Blood sample will be separated by ficoll gradient for PAR assay. The blood samples will be drawn 4 hrs after the first Cycle 2 Day 9 dose of ABT-888.

[7] Cycle 2 only: 1x4 mL of blood for PK collected at 48 h from time of Day 8 infusion.

[8] Cycle 2 only: The first dose of ABT-888 to be administered on Day 8 of Cycle 2 will be taken at the time of the start of irinotecan infusion, so PK sampling up to 48 h can be lined up for both drugs.

10.4 Weekly Calendar (Expansion Portion)

	Pre-Study	Wk 1	Wk 2	Wk 3	Wk 4	Wk 5	Wk 6	Wk 7	Wk 8	Wk 9	Wk 10	Wk 11	Wk 12	Off Study[6]
ABT-888		X[1]	X[1]	X[1]	X[2]	X[2]	X[2]	X[2]	X[2]	X[2]	X[2]	X[2]	X[2]	
Irinotecan (CPT-11)[3]		X	X		X	X		X	X		X	X		
Archival tissue[19]	X													
Informed consent	X													
Demographics	X													
Medical history	X													
Concurrent meds	X	X---X												
Physical exam	X	X	X	X	X	X		X	X		X	X		X
Vital signs	X	X	X	X	X	X		X	X		X	X		X
Height	X													
Weight	X	X	X	X	X	X		X	X		X	X		X
Performance status	X	X	X	X	X	X		X	X		X	X		X
CBC w/diff, plts	X	X	X	X	X	X		X	X		X	X		X
Serum chemistry[4]	X	X	X	X	X	X		X	X		X	X		X
EKG[15]	X	X		X										
AE evaluation		X---X												X
Tumor measurements	X	Tumor measurements are repeated every 6 weeks. Documentation (radiologic) must be provided for patients removed from study for PD. Confirmatory scans will also be obtained 6 weeks following initial documentation of an objective response (CR or PR).												X[6]
Radiologic evaluation	X	Radiologic measurements should be performed every 6 weeks. Confirmatory scans will also be obtained 6 weeks following initial documentation of an objective (complete or partial) response.												X[6]
β-HCG	X[5]													
UGT1A1 status	X[16]													
Blood draw -PBMCs	X[7]	X[8]	X[9]											
Blood draw - CTCs	X[10]	X[11]	X[12]	X[13]										
Blood draw - genomics	X[18]													
Biopsy (PD)	X[14]	X[14]	X[14]											
Participant diary given		X			X			X			X			
BRCA status[17]	X													

1: ABT-888: twice daily oral administration (40 mg po bid) begins Day 2 through Day 15 of 21-day cycle followed by 6 day rest.
2: ABT-888: twice daily oral administration (40 mg po bid) begins Day 1 through Day 15 of 21-day cycle followed by 6 day rest
3: Irinotecan: 100 mg/m^2 administered on days 1 and 8 of each cycle over 90 minutes i.v..
4: Albumin, alkaline phosphatase, total bilirubin, bicarbonate, BUN, calcium, chloride, creatinine, glucose, LDH, phosphorus, potassium, total protein, SGOT [AST], SGPT [ALT], sodium.
5: Serum pregnancy test (women of childbearing potential).
6: Off-study evaluation.
7: Blood for PBMCs (8 ml) collected at the time of biopsy, within two weeks of study treatment.
8: Blood for PBMCs (8 ml each timepoint) collected at each of the following beginning Cycle 1, day 1: pre-infusion,and 4-6, 8, 22, and 24 hours after the start of infusion.
9: Blood for PBMCs (8 ml each timepoint) collected at each of the following beginning Cycle 1, day 8: pre-infusion,and 4-6, 8, 22, and 24 hours after the start of infusion. The first dose of ABT-888 to be administered on Day 8 of Cycle 1 will be taken at the start of CPT-11 infusion, so sampling can be lined up for both drugs. The PBMC samples drawn on Day 9 of Cycle 1 will occur 22 and 24 hours after start of the combination of ABT-888 and chemotherapy on Day 8 of Cycle 1. The first dose of ABT-888 taken on Day 9 should occur following the 24-hr blood draw.
10: Blood for CTCs (8 ml) collected at the time of biopsy, within two weeks of study treatment.
11: Blood for CTCs (8 ml each timepoint) collected at each of the following beginning Cycle 1, day 1: pre-infusion,and 4-6, 8, 22, and 24 hours after the start of infusion.
12: Blood for CTCs (8 ml each timepoint) collected at each of the following beginning Cycle 1, day 1: pre-infusion,and 4-6, 8, 22, and 24 hours after the start of infusion. The first dose of ABT-888 to be administered on Day 8 of Cycle 1 will be taken at the start of CPT-11 infusion, so sampling can be lined up for both drugs. The CTC samples drawn on Day 9 of Cycle 1 will occur 22 and 24 hours after start of the combination of ABT-888 and chemotherapy on Day 8 of Cycle 1. The first dose of ABT-888 taken on Day 9 should occur following the 24-hr blood draw.

13: Blood for CTCs (8 ml) collected Day 15, at 4 hrs after the first Day 15 dose of ABT-888 scheduled that day.
14: Biopsy to occur within 2 weeks of first treatment, and 4-6 hours after beginning of infusion on the following days: Day 1, Cycle 1 and Day 8, Cycle 1. All biopsies will consist of 4-6 passes with a 16 or 18 gauge needle.
15: EKGs to occur prior to treatment (baseline) and after first dose of ABT-888 on both cycle 1, day 2 and cycle 1, day 15.
16: Standard lab test to determine absence of UGT1A1*28 allele. Only homozygous individuals will be excluded from eligibility
17: Verification of BRCA mutational status (at any time pre-study or during Cycle 1 for the first 10 patients enrolled on the Expansion Portion of the study. For patients 11-20, BRCA status must be verified prior to baseline biopsy)
18: 1-2 acid citrate dextrose (ACD) yellow top collection tubes with 10-20mls of peripheral blood using standard blood collection techniques for sequencing of somatic mutations.
19: Archival tissue block or paraffin sample from archival tissue block (approximately 20 sections): 10 slices of tissue with a thickness of approximately 10 microns and 10 slices of tissue with a thickness of approximately 4 to 6 microns

10.5 Daily Calendar (Cycle 1) Expansion Portion

Studies & Tests	Pre	Cycle 1							
		Day 1	Day 2	Day 3-7	Day 8	Day 9	Day 10-14	Day 15	Day 16-21
Informed Consent	X								
Demographics	X								
Medical History	X								
Archival tissue collected[18]	X								
Concurrent Meds	X	X			X			X	
Vital Signs	X	X			X			X	
Height	X								
Weight	X	X			X			X	
CBC with differential; platelets	X	X			X			X	
Serum chemistry [1]	X	X			X			X	
EKG	X		X					X	
AE Evaluation		X	X		X	X		X	
Tumor Measurements	X								
Radiologic Evaluation	X								
β-HCG [2]	X								
UGT1A1	X[15]								
Physical and ECOG PS	X	X			X			X	
Irinotecan infusion		X			X				
ABT-888 given (po BID)			X	X	X	X	X	X	
Blood Draw - PBMC	X[3]	X[4]	X[5]		X[6]	X[7]			
Blood Draw - CTC	X[8]	X[9]	X[10]		X[11]	X[12]		X[13]	
Blood Draw - genomics	X[17]								
Participant Diary Given			X						
Tumor collection	X[14]	X[14]			X[14]				
BRCA mutational status [16]	X								

1 Albumin, alkaline phosphatase, total bilirubin, bicarbonate, BUN, calcium, chloride, creatinine, glucose, LDH, phosphorus, potassium, total protein, SGOT [AST], SGPT [ALT], sodium.
2 Within 2 weeks of first day of study for women of childbearing potential
3: Blood for PBMCs (8 ml) collected at the time of biopsy, within two weeks of study treatment.
4: Blood for PBMCs (8 ml each timepoint) collected at each of the following beginning Cycle 1, day 1: pre-infusion, and 4-6, 8, hours after the start of irinotecan infusion.
5: Blood for PBMCs (8 ml each timepoint) collected at each of the following beginning Cycle 1, day 2: 22, and 24 hours after the start of Day 1 irinotecan infusion.
6: Blood for PBMCs (8 ml each timepoint) collected at each of the following beginning Cycle 1, day 8: pre-infusion,and 4-6 and 8, hours after the start of irinotecan infusion. The first dose of ABT-888 to be administered on Day 8 of Cycle 1 will be taken at the start of CPT-11 infusion, so sampling can be lined up for both drugs.
7: Blood for PBMCs (8 ml each timepoint) collected at each of the following beginning Cycle 1, day 9: 22 and 24 hours after the start of the combination of ABT-888 and chemotherapy on Day 8 of Cycle 1. The first dose of ABT-888 taken on Day 9 should occur following the 24-hr blood draw.
8: Blood for CTCs (8 ml) collected at the time of biopsy, within two weeks of study treatment.
9: Blood for CTCs (8 ml each timepoint) collected at each of the following beginning Cycle 1, day 1: pre-infusion, and 4-6, 8, hours after the start of irinotecan infusion.
10: Blood for CTCs (8 ml each timepoint) collected at each of the following beginning Cycle 1, day 2: 22, and 24 hours after the start of Day 1 irinotecan infusion.

11: Blood for CTCs (8 ml each timepoint) collected at each of the following beginning Cycle 1, day 8: pre-infusion,and 4-6 and 8, hours after the start of irinotecan infusion. The first dose of ABT-888 to be administered on Day 8 of Cycle 1 will be taken at the start of CPT-11 infusion, so sampling can be lined up for both drugs.
12: Blood for CTCs (8 ml each timepoint) collected at each of the following beginning Cycle 1, day 9: 22 and 24 hours after the start of the combination of ABT-888 and chemotherapy on Day 8 of Cycle 1. The first dose of ABT-888 taken on Day 9 should occur following the 24-hr blood draw.
13: Blood for CTCs (8 ml) collected Day 15, at 4 hrs after the first Day 15 dose of ABT-888 scheduled that day.
14: Biopsy to occur within 2 weeks of first treatment, and 4-6 hours after beginning of infusion on the following days: Day 1, Cycle 1 and Day 8, Cycle 1 Initial (baseline) biopsy will consist of 4-6 passes with a 16 or 18 gauge needle.
15: Standard lab test to determine absence of UGT1A1*28 allele. Homozygous patients will be excluded from study
16: Verification of BRCA mutational status (at any time pre-study or during Cycle 1 for the first 10 patients enrolled on the Expansion Portion of the study. For patients 11-20, BRCA status must be verified prior to baseline biopsy).
17: 1-2 acid citrate dextrose (ACD) yellow top collection tubes with 10-20mls of peripheral blood using standard blood collection techniques for sequencing of somatic mutations.
18: Archival tissue block or paraffin sample from archival tissue block (approximately 5-10 sections)): 10 slices of tissue with a thickness of approximately 10 microns and 10 slices of tissue with a thickness of approximately 4 to 6 microns

10.6 Daily Calendar (Subsequent Cycles) – Expansion Portion

Studies & Tests	Subsequent cycles				
	Day 1	Day 2-7	Day 8	Day 9-15	Day 16-21
Concurrent Meds	X		X		
Vital Signs	X		X		
Weight	X		X		
CBC with differential; plts	X		X		
Serum chemistry [1]	X		X		
AE Evaluation	X		X		
Tumor Measurements	Tumor measurements are repeated every 6 weeks. Documentation (radiologic) must be provided for patients removed from study for PD. Confirmatory scans will also be obtained 6 weeks following initial documentation of an objective response (CR or PR).				
Radiologic Evaluation	Radiologic measurements should be performed every 6 weeks. Confirmatory scans will also be obtained 6 weeks following initial documentation of an objective (complete or partial) response.				
Physical and ECOG PS	X		X		
Irinotecan infusion	X		X		
ABT-888 given (po BID)	X	X	X	X	
Participant Diary Given	X				

1 Albumin, alkaline phosphatase, total bilirubin, bicarbonate, BUN, calcium, chloride, creatinine, glucose, LDH, phosphorus, potassium, total protein, SGOT [AST], SGPT [ALT], sodium.

10.7 Weekly Calendar (Dose Escalation for Intermittent ABT-888)

Baseline evaluations are to be conducted within 2 weeks prior to start of protocol therapy. Scans and x-rays must be done ≤ 4 weeks prior to the start of therapy. In the event that the patient's condition is deteriorating, lab evals should be repeated within 48 hr prior to initiation of the next cycle of therapy.

	Pre-Study	Run-In	Wk 1	Wk 2	Wk 3	Wk 4[12]	Wk 5[12]	Wk 6	Wk 7[12]	Wk 8[12]	Wk 9	Wk 10[12]	Wk 11[12]	Wk 12	Off Study[5]
ABT-888[1]		X	X	X		X	X		X	X		X	X		
Irinotecan (CPT-11)[2]			X	X		X	X		X	X		X	X		
Informed consent	X														
Demographics	X														
Medical history	X														
Concurrent meds	X		X--X												
Physical exam	X	X	X	X		X	X		X	X		X	X		X
Vital signs	X	X	X	X		X	X		X	X		X	X		X
Height	X														
Weight	X	X	X	X		X	X		X	X		X	X		X
Performance status	X	X	X	X		X	X		X	X		X	X		X
CBC w/diff, plts	X	X	X	X	X	X	X		X	X		X	X		X
Serum chemistry[3]	X	X	X	X	X	X	X		X	X		X	X		X
EKG[6]	X														
AE evaluation		X---X													X
Tumor measurements	X		Tumor measurements are repeated every 6 weeks[11]. Documentation (radiologic) must be provided for patients removed from study for PD. Confirmatory scans will also be obtained 6 weeks following initial documentation of an objective response (CR or PR).												X[5]
Radiologic evaluation	X		Radiologic measurements should be performed every 6 weeks. Confirmatory scans will also be obtained 6 weeks following initial documentation of an objective (complete or partial) response.												X[5]
β-HCG	X[4]														
Biopsy[7]	X	X		X											
Blood Draw-genomics	X[8]														
Participant diary given		X[9]	X[10]			X[10]			X[10]			X[10]			

1: ABT-888: po bid days -14 to -12 and once on day -11 (morning dose only) of run-in, then ABT-888 bid on days 1 to day 4 and days 8 to 11 of each 21 day cycle
2: Irinotecan: administered on days 3 and 10 of each cycle over 90 minutes i.v.
3: Albumin, alkaline phosphatase, total bilirubin, bicarbonate, BUN, calcium, chloride, creatinine, glucose, LDH, phosphorus, potassium, total protein, SGOT [AST], SGPT [ALT], sodium.
4: Serum pregnancy test (women of childbearing potential).
5: Off-study evaluation.
6. EKGs at baseline and as directed by treating oncologist/principal investigator
7. Tumor biopsy will be performed at baseline, within 4 hours after morning ABT-888 dose on day -11 and within 4 hours of ABT-888 dose on cycle 1 day 11
8: 1-2 acid citrate dextrose (ACD) yellow top collection tubes with 10-20mls of peripheral blood using standard blood collection techniques for sequencing of somatic mutations.
9: Patient Pill Diary for "Run in" given Day -14
10: Patient Pill Diary for "All Cycles" Given Day 1
11. Tumor measurements will be performed 6 weeks after the first day of treatment (first day of treatment is considered Cycle 1, Day 1) and repeated every 6 weeks.
12. Starting with Cycle 2, we allow a window of +/- 1 day for visits (i.e. Cycle 2+, Day 1, Cycle 2+, Day 3 and Cycle 2+, Day 10)

10.8 Daily Calendar (Run-In and Cycle 1) Dose Escalation for Intermittent ABT-888

Baseline evaluations are to be conducted within 2 weeks prior to start of protocol therapy. Scans and x-rays must be done ≤ 4 weeks prior to the start of therapy. In the event that the patient's condition is deteriorating, lab evals should be repeated within 48 hr prior to initiation of the next cycle of therapy.

Studies & Tests	Pre	Run-In				Cycle 1										
		D -14	D -13 to -12	D -11	D -10 to -1	D 1	D 2	D 3	D 4	D 5-7	D 8-9	D 10	D 11	D 12-14	D 15	D 16-21
Informed Consent	X															
Demographics	X															
Medical History	X															
Concurrent Meds	X	X				X						X				
Vital Signs	X	X				X						X				
Height	X															
Weight	X	X				X						X				
CBC with differential; platelets	X	X				X						X			X	
Serum chemistry [1]	X	X				X						X			X	
EKG[6]	X															
AE Evaluation		X--X														
Tumor Measurements	X															
Radiologic Evaluation	X															
β-HCG [2]	X															
Biopsy[4]	X			X									X			
Research Blood Draw	X[5]															
Physical and ECOG PS	X	X				X						X				
Irinotecan infusion								X				X				
ABT-888[3]		X	X	X		X	X	X	X		X	X	X			
Participant Diary Given		X[7]				X[8]										

[1] Albumin, alkaline phosphatase, total bilirubin, bicarbonate, BUN, calcium, chloride, creatinine, glucose, LDH, phosphorus, potassium, total protein, SGOT [AST], SGPT [ALT], sodium.
[2] Within 2 weeks of first day of study for women of childbearing potential
[3] ABT-888 po bid days -14 to -12 and once on day -11 (morning dose only) of run-in, then ABT-888 bid on days 1 to day 4 and days 8 to 11
[4] Tumor biopsy will be performed at baseline, within 4 hours after morning ABT-888 dose on day -11 and within 4 hours of ABT-888 dose on cycle 1 day 11
[5] 1-2 acid citrate dextrose (ACD) yellow top collection tubes with 10-20mls of peripheral blood using standard blood collection techniques.
[6] EKGs at baseline and as directed by treating oncologist/principal investigator
[7] Patient Pill Diary for "Run in" given Day -14
[8] Patient Pill Diary for "All Cycles" Given Day 1

10.9 Daily Calendar (Subsequent Cycles) –Dose Escalation for Intermittent ABT-888

Studies & Tests			Subsequent cycles						
	Day 1[3]	Day 2	Day 3[3]	Day 4	Day 5-7	Day 8-9	Day 10[3]	Day 11	Day 12-21
Concurrent Meds	X						X		
Vital Signs	X						X		
Weight	X						X		
CBC with differential; plts	X						X		
Serum chemistry [1]	X						X		
AE Evaluation	X---X								
Tumor Measurements[2]		Tumor measurements are repeated every 6 weeks[2]. Documentation (radiologic) must be provided for patients removed from study for PD. Confirmatory scans will also be obtained 6 weeks following initial documentation of an objective response (CR or PR).							
Radiologic Evaluation		Radiologic measurements should be performed every 6 weeks. Confirmatory scans will also be obtained 6 weeks following initial documentation of an objective (complete or partial) response.							
Physical and ECOG PS	X						X		
Irinotecan infusion			X				X		
ABT-888 given (po BID)	X	X	X	X		X	X	X	
Participant Diary Given	X								

[1] Albumin, alkaline phosphatase, total bilirubin, bicarbonate, BUN, calcium, chloride, creatinine, glucose, LDH, phosphorus, potassium, total protein, SGOT [AST], SGPT [ALT], sodium.
[2] Tumor measurements will be performed 6 weeks after the first day of treatment (first day of treatment is considered Cycle 1, Day 1) and repeated every 6 weeks.
[3] Starting with Cycle 2, we allow a window of +/- 1 day for visits (i.e. Cycle 2+, Day 1, Cycle 2+, Day 3 and Cycle 2+, Day 10)

11 MEASUREMENT OF EFFECT

Although response is not the primary endpoint of this trial, patients with measurable disease will be assessed by standard criteria. For the purposes of this study, patients should be re-evaluated every 6 weeks (every 2 cycles). Due to the fact that a cycle is only 3 weeks in length, we elect not to perform scans more frequently due to lack of relevance of short interval comparison and third-party payer issues. In addition to a baseline scan, confirmatory scans will also be obtained 6 weeks following initial documentation of an objective response.

11.1 Antitumor Effect – Solid Tumors

Response and progression will be evaluated in this study using the new international criteria proposed by the revised Response Evaluation Criteria in Solid Tumors (RECIST) guideline (version 1.1).(68) Changes in the largest diameter (unidimensional measurement) of the tumor lesions and the shortest diameter in the case of malignant lymph nodes are used in the RECIST criteria.

11.1.1 Definitions

Evaluable for toxicity.

Original Dose Escalation and Expansion Portions: All patients will be evaluable for toxicity from the time of their first treatment with irinotecan. However, due to the investigational nature of this protocol, the primary toxicity endpoint is the combination of irinotecan and ABT-888.

Dose Escalation for Intermittent ABT-888 Portion:
All patients will be evaluable for toxicity from the time of their first treatment with ABT-888. However, due to the investigational nature of this protocol, the primary toxicity endpoint is the combination of irinotecan and ABT-888. Participants who do not complete the first cycle of treatment and do not experience a DLT will be replaced. If 6 patients are required as replacement patients to complete the necessary patient numbers for cohort completion, a hold will be placed on further patient recruitment until the PI and medical monitor at CTEP, NCI can evaluate causality for replacement. If patient "fall out" is deemed not secondary to a missed DLT, patient recruitment, including replacement, will continue.

Evaluable for objective response. Only those patients who have measurable disease present at baseline, have received at least one cycle of therapy, and have had their disease re-evaluated will be considered evaluable for response. These patients will have their response classified according to the definitions stated below. (Note: Patients who exhibit objective disease progression prior to the end of cycle 1 will also be considered evaluable.)

Evaluable Non-Target Disease Response. Patients who have lesions present at baseline that are evaluable but do not meet the definitions of measurable disease, have received at least one cycle of therapy, and have had their disease re-evaluated will be considered evaluable for non-target disease. The response assessment is based on the presence, absence, or unequivocal progression of the lesions.

11.1.2 **Disease Parameters**

Measurable disease. Measurable lesions are defined as those that can be accurately measured in at least one dimension (longest diameter to be recorded) as $\geq$20 mm by chest x-ray, as $\geq$10 mm with CT scan, or $\geq$10 mm with calipers by clinical exam. All tumor measurements must be recorded in millimeters (or decimal fractions of centimeters).

Note: Tumor lesions that are situated in a previously irradiated area might or might not be considered measurable. They will be considered measurable only if they had demonstrated tumor regrowth in previously irradiated areas.

Malignant lymph nodes. To be considered pathologically enlarged and measurable, a lymph node must be $\geq$15 mm in short axis when assessed by CT scan (CT scan slice thickness recommended to be no greater than 5 mm). At baseline and in follow-up, only the short axis will be measured and followed.

Non-measurable disease. All other lesions (or sites of disease), including small lesions (longest diameter <10 mm or pathological lymph nodes with ≥ 10 to <15 mm short axis), are considered non-measurable disease. Bone lesions, leptomeningeal disease, ascites, pleural/pericardial effusions, lymphangitis cutis/pulmonitis, inflammatory breast disease, and abdominal masses (not followed by CT or MRI), are considered as non-measurable.

Note: Cystic lesions that meet the criteria for radiographically defined simple cysts should not be considered as malignant lesions (neither measurable nor non-measurable) since they are, by definition, simple cysts.

'Cystic lesions' thought to represent cystic metastases can be considered as measurable lesions, if they meet the definition of measurability described above. However, if non-cystic lesions are present in the same patient, these are preferred for selection as target lesions.

Target lesions. All measurable lesions up to a maximum of 2 lesions per organ and 5 lesions in total, representative of all involved organs, should be identified as **target lesions** and recorded and measured at baseline. Target lesions should be selected on the basis of their size (lesions with the longest diameter), be representative of all involved organs, but in addition should be those that lend themselves to reproducible repeated measurements. It may be the case that, on occasion, the largest lesion does not lend itself to reproducible measurement in which circumstance the next largest lesion which can be measured reproducibly should be selected. A sum of the diameters (longest for non-nodal lesions, short axis for nodal lesions) for all target lesions will be calculated and reported as the baseline sum diameters. If lymph nodes are to be included in the sum, then only the short axis is added into the sum. The baseline sum diameters will be used as reference to further characterize any objective tumor regression in the measurable dimension of the disease.

Non-target lesions. All other lesions (or sites of disease) including any measurable lesions over and above the 5 target lesions should be identified as

non-target lesions and should also be recorded at baseline. Measurements of these lesions are not required, but the presence, absence, or in rare cases unequivocal progression of each should be noted throughout follow-up.

11.1.3 Methods for Evaluation of Measurable Disease

All measurements should be taken and recorded in metric notation using a ruler or calipers. All baseline evaluations should be performed as closely as possible to the beginning of treatment and never more than 4 weeks before the beginning of the treatment.

The same method of assessment and the same technique should be used to characterize each identified and reported lesion at baseline and during follow-up. Imaging-based evaluation is preferred to evaluation by clinical examination unless the lesion(s) being followed cannot be imaged but are assessable by clinical exam.

Clinical lesions Clinical lesions will only be considered measurable when they are superficial (e.g., skin nodules and palpable lymph nodes) and ≥10 mm diameter as assessed using calipers (e.g., skin nodules). In the case of skin lesions, documentation by color photography, including a ruler to estimate the size of the lesion, is recommended.

Chest x-ray Lesions on chest x-ray are acceptable as measurable lesions when they are clearly defined and surrounded by aerated lung. However, CT is preferable.

Conventional CT and MRI This guideline has defined measurability of lesions on CT scan based on the assumption that CT slice thickness is 5 mm or less. If CT scans have slice thickness greater than 5 mm, the minimum size for a measurable lesion should be twice the slice thickness. MRI is also acceptable in certain situations (e.g. for body scans).

Use of MRI remains a complex issue. MRI has excellent contrast, spatial, and temporal resolution; however, there are many image acquisition variables involved in MRI, which greatly impact image quality, lesion conspicuity, and measurement. Furthermore, the availability of MRI is variable globally. As with CT, if an MRI is performed, the technical specifications of the scanning sequences used should be optimized for the evaluation of the type and site of disease. Furthermore, as with CT, the modality used at follow-up should be the same as was used at baseline and the lesions should be measured/assessed on the same pulse sequence. It is beyond the scope of the RECIST guidelines to prescribe specific MRI pulse sequence parameters for all scanners, body parts, and diseases. Ideally, the same type of scanner should be used and the image acquisition protocol should be followed as closely as possible to prior scans. Body
scans should be performed with breath-hold scanning techniques, if possible.

PET-CT At present, the low dose or attenuation correction CT portion of a combined PET-CT is not always of optimal diagnostic CT quality for use with

RECIST measurements. However, if the site can document that the CT performed as part of a PET-CT is of identical diagnostic quality to a diagnostic CT (with IV and oral contrast), then the CT portion of the PET-CT can be used for RECIST measurements and can be used interchangeably with conventional CT in accurately measuring cancer lesions over time. Note, however, that the PET portion of the CT introduces additional data which may bias an investigator if it is not routinely or serially performed.

Ultrasound Ultrasound is not useful in assessment of lesion size and should not be used as a method of measurement. Ultrasound examinations cannot be reproduced in their entirety for independent review at a later date and, because they are operator dependent, it cannot be guaranteed that the same technique and measurements will be taken from one assessment to the next. If new lesions are identified by ultrasound in the course of the study, confirmation by CT or MRI is advised. If there is concern about radiation exposure at CT, MRI may be used instead of CT in selected instances.

Endoscopy, Laparoscopy The utilization of these techniques for objective tumor evaluation is not advised. However, such techniques may be useful to confirm complete pathological response when biopsies are obtained or to determine relapse in trials where recurrence following complete response (CR) or surgical resection is an endpoint.

Tumor markers Tumor markers alone cannot be used to assess response. If markers are initially above the upper normal limit, they must normalize for a patient to be considered in complete clinical response. Specific guidelines for both CA-125 response (in recurrent ovarian cancer) and PSA response (in recurrent prostate cancer) have been published [*JNCI* 96:487-488, 2004; *J Clin Oncol* 17, 3461-3467, 1999; *J Clin Oncol* 26:1148-1159, 2008]. In addition, the Gynecologic Cancer Intergroup has developed CA-125 progression criteria which are to be integrated with objective tumor assessment for use in first-line trials in ovarian cancer [*JNCI* 92:1534-1535, 2000].

Cytology, Histology These techniques can be used to differentiate between partial responses (PR) and complete responses (CR) in rare cases (e.g., residual lesions in tumor types, such as germ cell tumors, where known residual benign tumors can remain).

The cytological confirmation of the neoplastic origin of any effusion that appears or worsens during treatment when the measurable tumor has met criteria for response or stable disease is mandatory to differentiate between response or stable disease (an effusion may be a side effect of the treatment) and progressive disease.

FDG-PET While FDG-PET response assessments need additional study, it is sometimes reasonable to incorporate the use of FDG-PET scanning to complement CT scanning in assessment of progression (particularly possible 'new' disease). New lesions on the basis of FDG-PET imaging can be identified according to the following algorithm:

a. Negative FDG-PET at baseline, with a positive FDG-PET at follow-up is a sign of PD based on a new lesion.

b. No FDG-PET at baseline and a positive FDG-PET at follow-up: If the positive FDG-PET at follow-up corresponds to a new site of disease confirmed by CT, this is PD. If the positive FDG-PET at follow-up is not confirmed as a new site of disease on CT, additional follow-up CT scans are needed to determine if there is truly progression occurring at that site (if so, the date of PD will be the date of the initial abnormal FDG-PET scan). If the positive FDG-PET at follow-up corresponds to a pre-existing site of disease on CT that is not progressing on the basis of the anatomic images, this is not PD.

c. FDG-PET may be used to upgrade a response to a CR in a manner similar to a biopsy in cases where a residual radiographic abnormality is thought to represent fibrosis or scarring. The use of FDG-PET in this circumstance should be prospectively described in the protocol and supported by disease-specific medical literature for the indication. However, it must be acknowledged that both approaches may lead to false positive CR due to limitations of FDG-PET and biopsy resolution/sensitivity.

Note: A 'positive' FDG-PET scan lesion means one which is FDG avid with an uptake greater than twice that of the surrounding tissue on the attenuation corrected image.

11.1.4 Response Criteria

11.1.4.1 Evaluation of Target Lesions

<u>Complete Response (CR)</u>:	Disappearance of all target lesions. Any pathological lymph nodes (whether target or non-target) must have reduction in short axis to <10 mm.
<u>Partial Response (PR)</u>:	At least a 30% decrease in the sum of the diameters of target lesions, taking as reference the baseline sum diameters
<u>Progressive Disease (PD)</u>:	At least a 20% increase in the sum of the diameters of target lesions, taking as reference the smallest sum on study (this includes the baseline sum if that is the smallest on study). In addition to the relative increase of 20%, the sum must also demonstrate an absolute increase of at least 5 mm. (Note: the appearance of one or more new lesions is also considered progressions).
<u>Stable Disease (SD)</u>:	Neither sufficient shrinkage to qualify for PR nor sufficient increase to qualify for PD, taking as reference the smallest sum diameters while on study

11.1.4.2 Evaluation of Non-Target Lesions

<u>Complete Response (CR)</u>: Disappearance of all non-target lesions and normalization of tumor marker level. All lymph nodes must be non-pathological in size (<10 mm short axis)

Note: If tumor markers are initially above the upper normal limit, they must normalize for a patient to be considered in complete clinical response.

<u>Non-CR/Non-PD:</u> Persistence of one or more non-target lesion(s) and/or maintenance of tumor marker level above the normal limits

<u>Progressive Disease (PD)</u>: Appearance of one or more new lesions and/or *unequivocal progression* of existing non-target lesions. *Unequivocal progression* should not normally trump target lesion status. It must be representative of overall disease status change, not a single lesion increase.

Although a clear progression of "non-target" lesions only is exceptional, the opinion of the treating physician should prevail in such circumstances, and the progression status should be confirmed at a later time by the review panel (or Principal Investigator).

11.1.4.3 Evaluation of Best Overall Response

The best overall response is the best response recorded from the start of the treatment until disease progression/recurrence (taking as reference for progressive disease the smallest measurements recorded since the treatment started). The patient's best response assignment will depend on the achievement of both measurement and confirmation criteria.

For Patients with Measurable Disease (i.e., Target Disease)

Target Lesions	Non-Target Lesions	New Lesions	Overall Response	Best Overall Response when Confirmation is Required*
CR	CR	No	CR	≥4 wks. Confirmation**
CR	Non-CR/Non-PD	No	PR	≥4 wks. Confirmation**
CR	Not evaluated	No	PR	
PR	Non-CR/Non-	No	PR	

	PD/not evaluated			
SD	Non-CR/Non-PD/not evaluated	No	SD	documented at least once ≥4 wks. from baseline**
PD	Any	Yes or No	PD	no prior SD, PR or CR
Any	PD***	Yes or No	PD	
Any	Any	Yes	PD	

* See RECIST 1.1 manuscript for further details on what is evidence of a new lesion.
** Only for non-randomized trials with response as primary endpoint.
*** In exceptional circumstances, unequivocal progression in non-target lesions may be accepted as disease progression.

Note: Patients with a global deterioration of health status requiring discontinuation of treatment without objective evidence of disease progression at that time should be reported as "*symptomatic deterioration.*" Every effort should be made to document the objective progression even after discontinuation of treatment.

For Patients with Non-Measurable Disease (i.e., Non-Target Disease)

Non-Target Lesions	New Lesions	Overall Response
CR	No	CR
Non-CR/non-PD	No	Non-CR/non-PD*
Not all evaluated	No	not evaluated
Unequivocal PD	Yes or No	PD
Any	Yes	PD

* 'Non-CR/non-PD' is preferred over 'stable disease' for non-target disease since SD is increasingly used as an endpoint for assessment of efficacy in some trials so to assign this category when no lesions can be measured is not advised

11.1.5 Duration of Response

Duration of overall response: The duration of overall response is measured from the time measurement criteria are met for CR or PR (whichever is first recorded) until the first date that recurrent or progressive disease is objectively documented (taking as reference for progressive disease the smallest measurements recorded since the treatment started).

The duration of overall CR is measured from the time measurement criteria are first met for CR until the first date that progressive disease is objectively documented.

Duration of stable disease: Stable disease is measured from the start of the treatment until the criteria for progression are met, taking as reference the smallest measurements recorded since the treatment started, including the baseline measurements.

12. STUDY OVERSIGHT AND DATA REPORTING / REGULATORY REQUIREMENTS

Adverse event lists, guidelines, and instructions for AE reporting can be found in Section 7.0

(Adverse Events: List and Reporting Requirements).

12.1 Study Oversight

This protocol is monitored at several levels, as described in this section. The Protocol Principal Investigator is responsible for monitoring the conduct and progress of the clinical trial, including the ongoing review of accrual, patient-specific clinical and laboratory data, and routine and serious adverse events; reporting of expedited adverse events; and accumulation of reported adverse events from other trials testing the same drug(s). The Protocol Principal Investigator and statistician have access to the data at all times through the CTMS web-based reporting portal.

For the Phase 1 portion of this study, all decisions regarding dose escalation/expansion/de-escalation require sign-off by the Protocol Principal Investigator through the CTMS/IWRS. In addition, for the Phase 1 portion, the Protocol Principal Investigator will have at least monthly, or more frequently, conference calls with the Study Investigators and the CTEP Medical Officer(s) to review accrual, progress, and adverse events and unanticipated problems.

All Study Investigators at participating sites who register/enroll patients on a given protocol are responsible for timely submission of data via Medidata Rave and timely reporting of adverse events for that particular study. This includes timely review of data collected on the electronic CRFs submitted via Medidata Rave.

All studies are also reviewed in accordance with the enrolling institution's data safety monitoring plan.

12.2 Data Reporting

Data collection for this study will be done exclusively through Medidata Rave. Access to the trial in Rave is granted through the iMedidata application to all persons with the appropriate roles assigned in the Regulatory Support System (RSS). To access Rave via iMedidata, the site user must have an active CTEP IAM account (check at < https://ctepcore.nci.nih.gov/iam >) and the appropriate Rave role (Rave CRA, Read-Only, CRA (Lab Admin, SLA or Site Investigator) on either the LPO or participating organization roster at the enrolling site. To the hold Rave CRA role or CRA Lab Admin role, the user must hold a minimum of an AP registration type. To hold the Rave Site Investigator role, the individual must be registered as an NPIVR or IVR. Associates can hold read-only roles in Rave.

Upon initial site registration approval for the study in RSS, all persons with Rave roles assigned on the appropriate roster will be sent a study invitation e-mail from iMedidata. To accept the invitation, site users must log into the Select Login (https://login.imedidata.com/selectlogin) using their CTEP-IAM user name and password, and click on the "accept" link in the upper right-corner of the iMedidata page. Please note, site users will not be able to access the study in Rave until all required Medidata and study specific trainings are completed. Trainings will be in the form of electronic learnings (eLearnings), and can be accessed by clicking on the link in the upper right pane of the iMedidata screen.

Users that have not previously activated their iMedidata/Rave account at the time of initial site registration approval for the study in RSS will also receive a separate invitation from iMedidata

to activate their account. Account activation instructions are located on the CTSU website, Rave tab under the Rave resource materials (Medidata Account Activation and Study Invitation Acceptance). Additional information on iMedidata/Rave is available on the CTSU members' website under the Rave tab or by contacting the CTSU Help Desk at 1-888-823-5923 or by e-mail at ctsucontact@westat.com.

12.2.1 Method

CTMS Comprehensive Monitoring:
This study will be monitored by the Clinical Trials Monitoring Service (CTMS). Data will be submitted to CTMS at least once every two weeks via Medidata Rave (or other modality if approved by CTEP). Information on CTMS reporting is available at http://www.theradex.com/clinicalTechnologies/?National-Cancer-Institute-NCI-11. On-site audits will be conducted three times annually (one annual site visit and two data audits). For CTMS monitored studies, after users have activated their accounts, please contact the Theradex Help Desk at (609) 799-7580 or by email at CTMSSupport@theradex.com for additional support with Rave and completion of CRFs.

12.2.2 Responsibility for Data Submission

For ETCTN trials, it is the responsibility of the PI(s) at the site to ensure that all investigators at the ETCTN Sites understand the procedures for data submission for each ETCTN protocol and that protocol specified data are submitted accurately and in a timely manner to the CTMS via the electronic data capture system, Medidata Rave.

Data are to be submitted via Medidata Rave to CTMS on a real-time basis, but no less than once every 2 weeks. The timeliness of data submissions and timeliness in resolving data queries will be tracked by CTMS. Metrics for timeliness will be followed and assessed on a quarterly basis. For the purpose of Institutional Performance Monitoring, data will be considered delinquent if it is greater than 4 weeks past due.

Data from Medidata Rave and CTEP-AERS is reviewed by the CTMS on an ongoing basis as data is received. Queries will be issued by CTMS directly within Rave. The queries will appear on the Task Summary Tab within Rave for the CRA at the ETCTN to resolve. Monthly web-based reports are posted for review by the Drug Monitors in the IDB, CTEP. Onsite audits will be conducted by the CTMS to ensure compliance with regulatory requirements, GCP, and NCI policies and procedures with the overarching goal of ensuring the integrity of data generated from NCI-sponsored clinical trials, as described in the ETCTN Program Guidelines, which may be found on the CTEP (http://ctep.cancer.gov/protocolDevelopment/electronic_applications/adverse_events.htm) and CTSU websites.

An End of Study CRF is to be completed by the PI, and is to include a summary of study endpoints not otherwise captured in the database, such as (for phase 1 trials) the recommended phase 2 dose (RP2D), and a description of any dose-limiting toxicities (DLTs). CTMS will utilize a core set of eCRFs that are Cancer Data Standards Registry and Repository (caDSR) compliant (http://cbiit.nci.nih.gov/ncip/biomedical-informatics-resources/interoperability-and-semantics/metadata-and-models). Customized eCRFs will be included when appropriate to meet unique study requirements. The PI is

encouraged to review the eCRFs, working closely with CTMS to ensure prospectively that all required items are appropriately captured in the eCRFs prior to study activation. CTMS will prepare the eCRFs with built-in edit checks to the extent possible to promote data integrity.

CDUS data submissions for ETCTN trials activated after March 1, 2014, will be carried out by the CTMS contractor, Theradex. CDUS submissions are performed by Theradex on a monthly basis. The trial's lead institution is responsible for timely submission to CTMS via Rave, as above.

Further information on data submission procedures can be found in the ETCTN Program Guidelines (http://ctep.cancer.gov/protocolDevelopment/electronic_applications/adverse_events.htm).

12.3 Cooperative Research and Development Agreement (CRADA)/Clinical Trials Agreement (CTA)

The agent(s) supplied by CTEP, DCTD, NCI used in this protocol is/are provided to the NCI under a Collaborative Agreement (CRADA, CTA, CSA) between the Pharmaceutical Company(ies) (hereinafter referred to as ACollaborator(s)@) and the NCI Division of Cancer Treatment and Diagnosis. Therefore, the following obligations/guidelines, in addition to the provisions in the AIntellectual Property Option to Collaborator@ (http:// ctep.cancer.gov/industry) contained within the terms of award, apply to the use of the Agent(s) in this study:

1. Agent(s) may not be used for any purpose outside the scope of this protocol, nor can Agent(s) be transferred or licensed to any party not participating in the clinical study. Collaborator(s) data for Agent(s) are confidential and proprietary to Collaborator(s) and shall be maintained as such by the investigators. The protocol documents for studies utilizing investigational Agents contain confidential information and should not be shared or distributed without the permission of the NCI. If a copy of this protocol is requested by a patient or patient's family member participating on the study, the individual should sign a confidentiality agreement. A suitable model agreement can be downloaded from: http://ctep.cancer.gov.

2. For a clinical protocol where there is an investigational Agent used in combination with (an)other investigational Agent(s), each the subject of different collaborative agreements , the access to and use of data by each Collaborator shall be as follows (data pertaining to such combination use shall hereinafter be referred to as "Multi-Party Data."):

   a. NCI will provide all Collaborators with prior written notice regarding the existence and nature of any agreements governing their collaboration with NIH, the design of the proposed combination protocol, and the existence of any obligations that would tend to restrict NCI's participation in the proposed combination protocol.

   b. Each Collaborator shall agree to permit use of the Multi-Party Data from the clinical trial by any other Collaborator solely to the extent necessary to

allow said other Collaborator to develop, obtain regulatory approval or commercialize its own investigational Agent.

c. Any Collaborator having the right to use the Multi-Party Data from these trials must agree in writing prior to the commencement of the trials that it will use the Multi-Party Data solely for development, regulatory approval, and commercialization of its own investigational Agent.

3. Clinical Trial Data and Results and Raw Data developed under a Collaborative Agreement will be made available exclusively to Collaborator(s), the NCI, and the FDA, as appropriate and unless additional disclosure is required by law or court order. Additionally, all Clinical Data and Results and Raw Data will be collected , used and disclosed consistent with all applicable federal statutes and regulations for the protection of human subjects, including, if applicable, the *Standards for Privacy of Individually Identifiable Health Information* set forth in 45 C.F.R. Part 164.
4. When a Collaborator wishes to initiate a data request, the request should first be sent to the NCI, who will then notify the appropriate investigators (Group Chair for Cooperative Group studies, or PI for other studies) of Collaborator's wish to contact them.

5. Any data provided to Collaborator(s) for Phase 3 studies must be in accordance with the guidelines and policies of the responsible Data Monitoring Committee (DMC), if there is a DMC for this clinical trial.

6. Any manuscripts reporting the results of this clinical trial must be provided to CTEP for immediate delivery to Collaborator(s) for advisory review and comment prior to submission for publication. Collaborator(s) will have 30 days from the date of receipt for review. Collaborator shall have the right to request that publication be delayed for up to an additional 30 days in order to ensure that Collaborator's confidential and proprietary data, in addition to Collaborator(s)'s intellectual property rights, are protected. Copies of abstracts must be provided to CTEP for forwarding to Collaborator(s) for courtesy review as soon as possible and preferably at least three (3) days prior to submission, but in any case, prior to presentation at the meeting or publication in the proceedings. Press releases and other media presentations must also be forwarded to CTEP prior to release. Copies of any manuscript, abstract and/or press release/ media presentation should be sent to:

   Regulatory Affairs Branch
   E-mail: ncicteppubs@mail.nih.gov

   The Regulatory Affairs Branch will then distribute them to Collaborator(s). No publication, manuscript or other form of public disclosure shall contain any of Collaborators confidential/ proprietary information.

13. STATISTICAL CONSIDERATIONS

13.1 Original Dose Escalation Portion

13.1.1 Study Design/Endpoints

The study design for the dose escalation portion is a standard "cohorts of 3-6 patients" Phase I design, with a standard escalation decision rule described in Sec. 5.2. The primary endpoints are the OBD, the MAD, the MTD, and the RP2D of each agent, and the observed adverse events (AEs). The OBD is defined in Sec. 5.1. The MAD, MTD, and RP2D are defined in Sec. 5.2.

The dose levels are defined in Sec. 5.1. The adverse events will be graded as described in Sec. 7.2. As indicated in Sec. 11.1.1, all patients who receive any amount of the study drug will be evaluable for toxicity.

The Treatment Plan predefines 6 dose levels of ABT-888 and irinotecan. The dose levels are defined in Sec. 5.1.

The absolute *minimum* number of patients required for the dose escalation portion of this Phase I trial would result from the unlikely (but possible) event in which there were ≥ 2 DLT's among 6 patients treated at dose level 1, such that it became the MAD. We would discuss this hypothetical outcome with CTEP, together define a new dose level 0 as the MTD, and treat 10 patients at that dose level 0 for PK and PD data collection. Thus, we expect that the absolute *minimum* number of patients required for this Phase I trial would be 6 + 10 = 16. In this scenario, the OBD might never be determined, since only one instance of PARP data collection at 2 successive dose levels (levels 0 and 1) would have occurred.

The *maximum* number of patients required to find the OBD and/or the MTD would result from these 3 requirements or conditions: (1) needing to treat 6 patients at each of dose levels 1-6; (2) finding the OBD without observing any DLT's; and (3) needing to expand dose level 6 by 4 additional patients for PK and PD data collection. Thus, the *maximum* number of patients required would be:
(6*5) + (6 + 4) = 40. In this scenario, the OBD should hopefully be determined along with the MTD.

We anticipate that the *most likely (i.e., probable) maximum* number of patients required to find the OBD and/or the MTD derives from these 2 assumptions:
(1) there will be very few (if any) DLT's at any of dose levels 1-5 (thus requiring only 3 patients each); and (2) dose level 6 would still be expanded to 10 patients for PK and PD data collection. Thus, finding the OBD and/or the MTD will require a *probable maximum* of (5*3) + (6 + 4) = 25 patients to complete the entire dose escalation portion of this Phase I study.

13.1.2 Sample Size/Accrual Rate

Based on recent accrual to Phase I studies at the Barbara Ann Karmanos Cancer Institute (KCI), we anticipate enrolling 10-12 patients/year from KCI, even allowing for competing Phase I trials. We also expect to accrue an

additional 5-7 patients/year from the University of Maryland Cancer Center (UMCC) and another 5-7 patients/year from the Dana-Farber Cancer Institute (DFCI). That yields an expected combined accrual rate of 20-26 patients/year. At our anticipated combined 3-institution accrual rate of 20-26 patients/year, the patient accrual duration would then be a *probable maximum* of 12-15 months to enroll 25 patients for the entire dose escalation portion of the Phase I study of ABT-888 + irinotecan.

13.1.3 Stratification Factors

Not applicable in a Phase I study

13.1.4 Analysis of Secondary Endpoints

For all objectives, the occurrence rate of binary endpoints (e.g., specific types of toxicity at a certain severity grade, response, etc.) will be described by point estimates and exact 90% confidence intervals. The 90% confidence level is appropriate for small to modest sample sizes, e.g., 3-6 patients per dose level cohort, or even for the full study expected sample size of 25 patients.

Continuous endpoints (e.g, PARP, all PK and PD parameters, γ-H2AX, Rad51, etc.) will be summarized with standard descriptive statistics (N, median, mean, standard deviation, minimum, maximum, and 90% confidence interval for the mean).

13.1.5 Analysis of Exploratory Endpoints

For Exploratory Objectives 1.5.1 and 1.5.2, the PD parameters will be summarized descriptively, by dose level and overall.
For Exploratory Objective 1.5.3, the PK parameters of each drug (Irinotecan and ABT-888) will be summarized descriptively within each CYP2C9 polymorphism category, by dose level and overall. Similar descriptive analyses will be performed by CYP2C19 polymorphism category, and by ABCG2 polymorphism category,

13.2 RP2D Cohort Expansion Portion

13.2.1 Study Design/Endpoints

The RP2D cohort will be expanded to include 20 breast cancer patients, 10 who are triple negative, BRCA-mutant positive and 10 who are triple-negative, BRCA-mutant negative, for additional exploratory biomarker and genomic correlative studies.

13.2.2 Sample Size/Accrual Rate

13.2 2.1 Triple negative, BRCA-mutant positive breast cancer patients:

We anticipate enrolling 5/yr from KCI, and 20/yr from DFCI. At a combined 2-institution accrual rate of 25 patients/year, 10 BRCA (+) breast cancer patients should be accrued in about 5 months.

13.2.2.2 Triple negative breast cancer (TNBC) patients who are BRCA (-):

We anticipate enrolling 20-25/yr from KCI, and 30/yr from DFCI. At a combined 2-institution accrual rate of 50-55 patients/year, 10 TNBC patients should be accrued in about 3 months.

.

13.2.3 Stratification Factors

Not applicable in a Phase I study.

13.2.4 Analysis of Exploratory Endpoints

Continuous endpoints (e.g, PARP, γ-H_2AX, CTC count, ERCC1 expression level, etc.) will be summarized with standard descriptive statistics (N, median, mean, standard deviation, minimum, maximum, and 90% confidence interval for the mean). The 90% confidence level is appropriate for small to modest sample sizes, e.g.,10 patients in each of the two RP2D subcohorts.

Separate exploratory PD assessments will be made for the two expansion sub-cohorts (10 TNBC BRCA (+) breast cancer patients, and 10 TNBC BRCA(-) patients). These assessments will include γ-H_2AX response, in tumor tissue and CTC's, PARP inhibition, and ERCC1 expression.

For Exploratory Objective 1.6.1, γ-H_2AX response in tumor tissue at 4-6 hrs will be correlated with γ-H_2AX response in CTC's at 4-6 hrs and at 8-24 hrs;

For Exploratory Objectives 1.6.2 and 1.6.3, PARP inhibition will be correlated with γ-H_2AX response in CTC's at 4-6 hrs and at 8-24 hrs

For Exploratory Objective 1.6.4,γ-H_2AX and ERCC1 responses in CTCs will be assessed at 15 and at 21 days.

For Exploratory Objective 1.6.5, we will explore the relationship of γ-H2AX and ERCC1 in tumor tissue and in PBMC's. The 2 scatterplots of ERCC1 vs. γ-H2AX, plus summary statistics will be generated. An obvious difference in the 2 relationships might suggest a "tumor switch" between (tumor) tissue vs PBMC's.

For Exploratory Objective 1.6.6, the day 15 CTC count distribution will be described in detail.

For Exploratory Objective 1.6.7, a descriptive analysis will be performed ***separately*** for each of the 2 expansion sub-cohorts: the 10 BRCA(-) patients, and the 10 BRCA(+) breast cancer patients. Since paired tumor and normal tissue will be available from each patient, the tumor vs normal differences will be of primary interest.

For Exploratory Objective 1.6.8, the continuous endpoints (e.g., levels of Rad51), will be summarized with standard descriptive statistics (N, median, mean, standard deviation, minimum, maximum, and the 90% confidence interval for the mean).

For Exploratory Objective 1.6.9, the continuous endpoints (e.g., % of BCSC before and after irinotecan alone and after 1 cycle of treatment with the combination of irinotecan and ABT-888) will be summarized with standard

descriptive statistics (N, median, mean, standard deviation, minimum, maximum, and the 90% confidence interval for the mean).

For Exploratory Objective 1.6.10, the continuous endpoints (gene expression levels) will be summarized with descriptive statistics appropriate to such distributions.

For Exploratory Objective 1.6.11, the number of Rad51 foci will be correlated with the number of ALDH+ stem cell populations at 4-6 hrs and at 8-24 hrs

All of the bioinformatics data preprocessing will be done at Tgen. For each of the 10 patients/group, Tgen will identify:

1) the somatic point mutations found in tumor tissue and in normal tissue;
2) the copy number variations as a log2 ratio for tumor:normal tissue; and
3) the primary structural changes (translocations, or intrachromosomal rearrangements).

Within each group of 10 patients, we will:

1) identify point mutations found in tumor tissue but not in normal tissue, if such events occur in multiple patients (since observing that in only 1 patient may be a false positive);
2) identify copy number variations found in tumor tissue but not in normal tissue, if such events occur in multiple patients;
3) identify primary structural changes found in tumor tissue but not in normal tissue, if such events occur in multiple patients.

13.3 Dose Escalation for Intermittent ABT-888 Portion

13.3.1 Study Design/Endpoints

The study design for the dose escalation for Intermittent ABT-888 portion is a standard “cohorts of 3-6 patients” Phase I design, with a standard escalation decision rule described in Table 3 of Sec. 5.2. The primary endpoints are the MAD, the MTD, and the RP2D of the combination of irinotecan and ABT-888, and the observed adverse events (AEs). The MAD, MTD, and RP2D are defined in Sec. 5.2.

The dose levels are defined at the end of Sec. 5.1 on page 41. The AEs will be graded as described in Sec. 7.2. As indicated in Sec. 11.1.1, all patients who receive any amount of the study drug will be evaluable for toxicity.

The Treatment Plan predefines 6 dose levels of ABT-888 and irinotecan. The dose levels are defined at the end of Sec. 5.1 on page 41.

The absolute *minimum* number of patients required for the dose escalation portion of this Phase I trial would result from the unlikely (but possible) event in which there were ≥ 2 DLT’s among 6 patients treated at dose level 1, such that it became the MAD. We would discuss this hypothetical outcome with CTEP, and together define if and how to proceed. Thus, we expect that the absolute *minimum* number of patients required for this Phase I trial would be 6.

The *maximum* number of patients required to the MTD would result from needing to treat 6 patients at each of dose levels 1-6. Thus, the *maximum* number of patients required would be: (6*6) = 36.

13.3.2 Sample Size/Accrual Rate

Based on accrual to Phase I studies at the Yale, Vanderbilt, Barbara Ann Karmanos Cancer Institute, and the Dana Farber Cancer Institute, we anticipate an expected combined accrual rate of 2-3 advanced solid tumor patients per month or 24-36 patients/year. At our anticipated combined 4-institution accrual rate of 24-36 patients/year, the patient accrual duration would then be a probable maximum of 12-18 months to enroll 36 patients for the dose escalation for Intermittent ABT-888 portion.

13.3.3 Stratification Factors

Not applicable in a Phase I study.

13.3.4 Analysis of Primary and Secondary Endpoints

The occurrence rate of binary endpoints (e.g., specific types of AE at a certain severity grade, response, etc.) will be described by point estimates and Wilson type 90% confidence intervals. The 90% confidence level is appropriate for small to modest sample sizes, e.g., 3-6 patients per dose level cohort, or even for the maximum expected sample size of 36 patients overall.

13.3.5 Analysis of Exploratory Endpoints

In general, exploratory correlative data from the 3 biopsies will be analyzed descriptively with summary statistics. For continuous variables, that would include: N, median, mean, standard deviation, minimum, maximum, and the 90% confidence interval for the mean). Statistical graphics (boxplots and dotplots) will also be generated.

These descriptive analyses and graphics will be generated for each exploratory correlative measured at each biopsy, and for the (absolute) difference in each such correlative at successive biopsy time points. For the difference variables, the convention will be to take the later biopsy value minus the earlier biopsy value so that a negative difference indicates a decrease in that correlative.

For Exploratory Objective 1.7.1 (developing assays to detect PARP trapping), PARP1 and PARP2 trapping levels will be analyzed descriptively, from each biopsy at which at which they are measured. Three different PARP trapping assays are described in Section 9.3.4: the subcellular fractionation assay; the FANCD2 monoubiquination assay; and the STORM assay. For the PARP1 and PARP2 trapping levels measured by each assay at each biopsy, descriptive statistics and statistical graphics will be generated as described in the previous paragraph. This will also include the difference variables in each such correlative at successive biopsy time points.

With 2 types of PARP trapping, 3 types of assays, 3 biopsy time points, and 3 sets of time point difference variables, there will be a total of 2*3*3*3 = 54 variables to be analyzed descriptively. Some limited correlation and linear

regression modeling of the level of PARP trapping with the level of FANCD2 monoubiquination is also planned, as described in Section 9.3.4.2.

For Exploratory Objective 1.7.2 (Additional exploratory assay to be named later.), a similar statistical analysis plan to the one just described for Exploratory Objective 1.7.1 will be followed for the resulting correlative data from each of the 3 biopsies.

14. REFERENCES

1. Ame JC, Spenlehauer C, de Murcia G. The PARP superfamily. Bioessays 2004;26(8):882-93.
2. Tomoda T, Kurashige T, Moriki T, Yamamoto H, Fujimoto S, Taniguchi T. Enhanced expression of poly(ADP-ribose) synthetase gene in malignant lymphoma. Am J Hematol 1991;37(4):223-7.
3. Shiobara M, Miyazaki M, Ito H, Togawa A, Nakajima N, Nomura F, et al. Enhanced polyadenosine diphosphate-ribosylation in cirrhotic liver and carcinoma tissues in patients with hepatocellular carcinoma. J Gastroenterol Hepatol 2001;16(3):338-44.
4. Fukushima M, Kuzuya K, Ota K, Ikai K. Poly(ADP-ribose) synthesis in human cervical cancer cell-diagnostic cytological usefulness. Cancer Lett 1981;14(3):227-36.
5. Alderson T. New targets for cancer chemotherapy--poly(ADP-ribosylation) processing and polyisoprene metabolism. Biol Rev Camb Philos Soc 1990;65(4):623-41.
6. Wielckens K, Garbrecht M, Kittler M, Hilz H. ADP-ribosylation of nuclear proteins in normal lymphocytes and in low-grade malignant non-Hodgkin lymphoma cells. Eur J Biochem 1980;104(1):279-87.
7. Berger NA, Adams JW, Sikorski GW, Petzold SJ, Shearer WT. Synthesis of DNA and poly(adenosine diphosphate ribose) in normal and chronic lymphocytic leukemia lymphocytes. J Clin Invest 1978;62(1):111-8.
8. Hirai K, Ueda K, Hayaishi O. Aberration of poly(adenosine diphosphate-ribose) metabolism in human colon adenomatous polyps and cancers. Cancer Res 1983;43(7):3441-6.
9. Fernet M, Ponette V, Deniaud-Alexandre E, de Murcia JM, de Murcia G, Giocanti N, et al. Poly(ADP-ribose) polymerase, a major determinant of early cell response to ionizing radiation. Int J Radiat Biol 2000;76(12):1621-9.
10. Shall S, de Murcia G. Poly(ADP-ribose) polymerase-1: what have we learned from the deficient mouse model? Mutat Res 2000;460(1):1-15.
11. Masutani M, Nozaki T, Nakamoto K, Nakagama H, Suzuki H, Kusuoka O, et al. The response of Parp knockout mice against DNA damaging agents. Mutat Res 2000;462(2-3):159-66.
12. Ame JC, Rolli V, Schreiber V, Niedergang C, Apiou F, Decker P, et al. PARP-2, A novel mammalian DNA damage-dependent poly(ADP-ribose) polymerase. J Biol Chem 1999;274(25):17860-8.
13. Menissier de Murcia J, Ricoul M, Tartier L, Niedergang C, Huber A, Dantzer F, et al. Functional interaction between PARP-1 and PARP-2 in chromosome stability and embryonic development in mouse. Embo J 2003;22(9):2255-63.
14. Schreiber V, Ame JC, Dolle P, Schultz I, Rinaldi B, Fraulob V, et al. Poly(ADP-ribose) polymerase-2 (PARP-2) is required for efficient base excision DNA repair in association with PARP-1 and XRCC1. J Biol Chem 2002;277(25):23028-36.
15. Johansson M. A human poly(ADP-ribose) polymerase gene family (ADPRTL): cDNA cloning of two novel poly(ADP-ribose) polymerase homologues. Genomics 1999;57(3):442-5.
16. Yu SW, Wang H, Poitras MF, Coombs C, Bowers WJ, Federoff HJ, et al. Mediation of poly(ADP-ribose) polymerase-1-dependent cell death by apoptosis-inducing factor. Science 2002;297(5579):259-63.
17. D'Amours D, Sallmann FR, Dixit VM, Poirier GG. Gain-of-function of poly(ADP-ribose) polymerase-1 upon cleavage by apoptotic proteases: implications for apoptosis. J Cell Sci 2001;114(Pt 20):3771-8.
18. Memisoglu A, Samson L. Base excision repair in yeast and mammals. Mutat Res

2000;451(1-2):39-51.
19. Ruscetti T, Lehnert BE, Halbrook J, Le Trong H, Hoekstra MF, Chen DJ, et al. Stimulation of the DNA-dependent protein kinase by poly(ADP-ribose) polymerase. J Biol Chem 1998;273(23):14461-7.
20. Galande S, Kohwi-Shigematsu T. Poly(ADP-ribose) polymerase and Ku autoantigen form a complex and synergistically bind to matrix attachment sequences. J Biol Chem 1999;274(29):20521-8.
21. Boulton S, Kyle S, Durkacz BW. Interactive effects of inhibitors of poly(ADP-ribose) polymerase and DNA-dependent protein kinase on cellular responses to DNA damage. Carcinogenesis 1999;20(2):199-203.
22. Liu L, Taverna P, Whitacre CM, Chatterjee S, Gerson SL. Pharmacologic disruption of base excision repair sensitizes mismatch repair-deficient and -proficient colon cancer cells to methylating agents. Clin Cancer Res 1999;5(10):2908-17.
23. Jagtap P, Szabo C. Poly(ADP-ribose) polymerase and the therapeutic effects of its inhibitors. Nat Rev Drug Discov 2005;4(5):421-40.
24. Bryant HE, Schultz N, Thomas HD, Parker KM, Flower D, Lopez E, et al. Specific killing of BRCA2-deficient tumours with inhibitors of poly(ADP-ribose) polymerase. Nature 2005;434(7035):913-7.
25. Farmer H, McCabe N, Lord CJ, Tutt AN, Johnson DA, Richardson TB, et al. Targeting the DNA repair defect in BRCA mutant cells as a therapeutic strategy. Nature 2005;434(7035):917-21.
26. Masutani M, Suzuki H, Kamada N, Watanabe M, Ueda O, Nozaki T, et al. Poly(ADP-ribose) polymerase gene disruption conferred mice resistant to streptozotocin-induced diabetes. Proc Natl Acad Sci U S A 1999;96(5):2301-4.
27. de Murcia JM, Niedergang C, Trucco C, Ricoul M, Dutrillaux B, Mark M, et al. Requirement of poly(ADP-ribose) polymerase in recovery from DNA damage in mice and in cells. Proceedings of the National Academy of Sciences of the United States of America 1997;94(14):7303-7.
28. Veuger SJ, Curtin NJ, Richardson CJ, Smith GC, Durkacz BW. Radiosensitization and DNA repair inhibition by the combined use of novel inhibitors of DNA-dependent protein kinase and poly(ADP-ribose) polymerase-1. Cancer Res 2003;63(18):6008-15.
29. Brock WA, Milas L, Bergh S, Lo R, Szabo C, Mason KA. Radiosensitization of human and rodent cell lines by INO-1001, a novel inhibitor of poly(ADP-ribose) polymerase. Cancer Lett 2004;205(2):155-60.
30. Calabrese CR, Almassy R, Barton S, Batey MA, Calvert AH, Canan-Koch S, et al. Anticancer chemosensitization and radiosensitization by the novel poly(ADP-ribose) polymerase-1 inhibitor AG14361. J Natl Cancer Inst 2004;96(1):56-67.
31. Obach RS, Baxter JG, Liston TE, Silber BM, Jones BC, MacIntyre F, et al. The prediction of human pharmacokinetic parameters from preclinical and in vitro metabolism data. The Journal of pharmacology and experimental therapeutics 1997;283(1):46-58.
32. Camptosar (irinotecan hydrochloride injection) drug package insert. Pfizer Inc, NY, NY. Revised July 2012.
33. Rasheed ZA, Rubin EH. Mechanisms of resistance to topoisomerase I-targeting drugs. Oncogene 2003;22(47):7296-304.
34. Armand JP, Extra YM, Catimel G, Abigerges D, Marty M, Clavel M. Rationale for the dosage and schedule of CPT-11 (irinotecan) selected for phase II studies, as determined by European phase I studies. Ann Oncol 1996;7(8):837-42.
35. Recommendations from the EGAPP Working Group: can UGT1A1 genotyping reduce morbidity and mortality in patients with metastatic colorectal cancer treated with

irinotecan? Genet Med 2009;11(1):15-20.
36. Etienne-Grimaldi M-C, Boyer J-C, Thomas F, Quaranta S, Picard N, Loriot M-A, et al. UGT1A1 genotype and irinotecan therapy: general review and implementation in routine practice. Fundamental & Clinical Pharmacology 2015;29(3):219-37.
37. Tentori L, Leonetti C, Scarsella M, d'Amati G, Portarena I, Zupi G, et al. Combined treatment with temozolomide and poly(ADP-ribose) polymerase inhibitor enhances survival of mice bearing hematologic malignancy at the central nervous system site. Blood 2002;99(6):2241-4.
38. Tentori L, Leonetti C, Scarsella M, D'Amati G, Vergati M, Portarena I, et al. Systemic administration of GPI 15427, a novel poly(ADP-ribose) polymerase-1 inhibitor, increases the antitumor activity of temozolomide against intracranial melanoma, glioma, lymphoma. Clin Cancer Res 2003;9(14):5370-9.
39. Tentori L, Leonetti C, Scarsella M, Muzi A, Mazzon E, Vergati M, et al. Inhibition of poly(ADP-ribose) polymerase prevents irinotecan-induced intestinal damage and enhances irinotecan/temozolomide efficacy against colon carcinoma. Faseb J 2006;20(10):1709-11.
40. Miknyoczki SJ, Jones-Bolin S, Pritchard S, Hunter K, Zhao H, Wan W, et al. Chemopotentiation of temozolomide, irinotecan, and cisplatin activity by CEP-6800, a poly(ADP-ribose) polymerase inhibitor. Mol Cancer Ther 2003;2(4):371-82.
41. Kinders RJ, Palma J, Liu X. Development of a quantitative enzyme immunoassay for measurement of PAR as a pharmacodynamic biomarker of PARP activity. 2006 AACR International Conference on Molecular Diagnostics in Cancer Therapeutic Development Chicago, IL 2006.
42. Plummer ER, Middleton MR, Jones C, Olsen A, Hickson I, McHugh P, et al. Temozolomide pharmacodynamics in patients with metastatic melanoma: dna damage and activity of repair enzymes O6-alkylguanine alkyltransferase and poly(ADP-ribose) polymerase-1. Clin Cancer Res 2005;11(9):3402-9.
43. Xu Y, Villalona-Calero MA. Irinotecan: mechanisms of tumor resistance and novel strategies for modulating its activity. Ann Oncol 2002;13(12):1841-51.
44. Viguier J, Boige V, Miquel C, Pocard M, Giraudeau B, Sabourin JC, et al. ERCC1 codon 118 polymorphism is a predictive factor for the tumor response to oxaliplatin/5-fluorouracil combination chemotherapy in patients with advanced colorectal cancer. Clin Cancer Res 2005;11(17):6212-7.
45. Ratnam K, Low JA. Current development of clinical inhibitors of poly(ADP-ribose) polymerase in oncology. Clin Cancer Res 2007;13(5):1383-8.
46. Innocenti F, Undevia SD, Iyer L, Chen PX, Das S, Kocherginsky M, et al. Genetic variants in the UDP-glucuronosyltransferase 1A1 gene predict the risk of severe neutropenia of irinotecan. J Clin Oncol 2004;22(8):1382-8.
47. Iyer L, Das S, Janisch L, Wen M, Ramirez J, Karrison T, et al. UGT1A1*28 polymorphism as a determinant of irinotecan disposition and toxicity. The pharmacogenomics journal 2002;2(1):43-7.
48. Tukey RH, Strassburg CP, Mackenzie PI. Pharmacogenomics of human UDP-glucuronosyltransferases and irinotecan toxicity. Molecular pharmacology 2002;62(3):446-50.
49. de Jong FA, Marsh S, Mathijssen RH, King C, Verweij J, Sparreboom A, et al. ABCG2 pharmacogenetics: ethnic differences in allele frequency and assessment of influence on irinotecan disposition. Clin Cancer Res 2004;10(17):5889-94.
50. Monaghan G, Ryan M, Seddon R, Hume R, Burchell B. Genetic variation in bilirubin UPD-glucuronosyltransferase gene promoter and Gilbert's syndrome. Lancet

1996;347(9001):578-81.
51. Wang SL, Huang J, Lai MD, Tsai JJ. Detection of CYP2C9 polymorphism based on the polymerase chain reaction in Chinese. Pharmacogenetics 1995;5(1):37-42.
52. Dandara C, Masimirembwa CM, Magimba A, Sayi J, Kaaya S, Sommers DK, et al. Genetic polymorphism of CYP2D6 and CYP2C19 in east- and southern African populations including psychiatric patients. European journal of clinical pharmacology 2001;57(1):11-7.
53. Backstrom G, Taipalensuu J, Melhus H, Brandstrom H, Svensson AC, Artursson P, et al. Genetic variation in the ATP-binding cassette transporter gene ABCG2 (BCRP) in a Swedish population. Eur J Pharm Sci 2003;18(5):359-64.
54. Charafe-Jauffret E, Ginestier C, Iovino F, Wicinski J, Cervera N, Finetti P, et al. Breast cancer cell lines contain functional cancer stem cells with metastatic capacity and a distinct molecular signature. Cancer Res 2009;69(4):1302-13.
55. Murai J, Huang SY, Das BB, Renaud A, Zhang Y, Doroshow JH, et al. Trapping of PARP1 and PARP2 by Clinical PARP Inhibitors. Cancer Res 2012;72(21):5588-99.
56. Yamtich J, Nemec AA, Keh A, Sweasy JB. A germline polymorphism of DNA polymerase beta induces genomic instability and cellular transformation. PLoS genetics 2012;8(11):e1003052.
57. Kim H, D'Andrea AD. Regulation of DNA cross-link repair by the Fanconi anemia/BRCA pathway. Genes & development 2012;26(13):1393-408.
58. Wang Y, Maharana S, Wang MD, Shivashankar, G.V. Super-resolution microscopy reveals decondensed chromatin structure at transcription sites. Scientific Reports 2014;4:1-7.
59. Khan S, Ahmad A, Ahmad I. A sensitive and rapid liquid chromatography tandem mass spectrometry method for quantitative determination of 7-ethyl-10-hydroxycamptothecin (SN-38) in human plasma containing liposome-based SN-38 (LE-SN38). Biomed Chromatogr 2003;17(8):493-9.
60. de Bruijn P, Verweij J, Loos WJ, Nooter K, Stoter G, Sparreboom A. Determination of irinotecan (CPT-11) and its active metabolite SN-38 in human plasma by reversed-phase high-performance liquid chromatography with fluorescence detection. J Chromatogr B Biomed Sci Appl 1997;698(1-2):277-85.
61. Chabot GG. Clinical pharmacokinetics of irinotecan. Clinical pharmacokinetics 1997;33(4):245-59.
62. Phatak P, Cookson JC, Dai F, Smith V, Stevens MFG, Burger AM. Telomere Uncapping by the G-Quadruplex Ligand RHPS4 Inhibits Clonogenic Tumour Cell Growth In Vitro and In Vivo Consistent with a Cancer Stem Cell Targeting Mechanism. Br J Cancer in press.
63. Wilson MK, Stone E, Vitolo M. Response of breast cancer cell lines to aminoflavone (NSC 686288) is associated with histone H2AX phosphorylation and estrogen receptor expression. Proc Amer Assoc Cancer Res 2006;47:555.
64. Codegoni AM, Castagna S, Mangioni C, Scovassi AI, Broggini M, D'Incalci M. DNA-topoisomerase I activity and content in epithelial ovarian cancer. Ann Oncol 1998;9(3):313-9.
65. Olaussen KA, Dunant A, Fouret P, Brambilla E, Andre F, Haddad V, et al. DNA repair by ERCC1 in non-small-cell lung cancer and cisplatin-based adjuvant chemotherapy. The New England journal of medicine 2006;355(10):983-91.
66. Burger AM, Zhang X, Li H, Ostrowski JL, Beatty B, Venanzoni M, et al. Down-regulation of T1A12/mac25, a novel insulin-like growth factor binding protein related gene, is associated with disease progression in breast carcinomas. Oncogene 1998;16(19):2459-

67.
67. Bittner M, Meltzer P, Chen Y, Jiang Y, Seftor E, Hendrix M, et al. Molecular classification of cutaneous malignant melanoma by gene expression profiling. Nature 2000;406(6795):536-40.
68. Eisenhauer EA, Therasse P, Bogaerts J, Schwartz LH, Sargent D, Ford R, et al. New response evaluation criteria in solid tumours: revised RECIST guideline (version 1.1). Eur J Cancer 2009;45(2):228-47.

APPENDIX A: Performance Status Criteria

ECOG Performance Status Scale		Karnofsky Performance Scale	
Grade	Descriptions	Percent	Description
0	Normal activity. Fully active, able to carry on all pre-disease performance without restriction.	100	Normal, no complaints, no evidence of disease.
		90	Able to carry on normal activity; minor signs or symptoms of disease.
1	Symptoms, but ambulatory. Restricted in physically strenuous activity, but ambulatory and able to carry out work of a light or sedentary nature (e.g., light housework, office work).	80	Normal activity with effort; some signs or symptoms of disease.
		70	Cares for self, unable to carry on normal activity or to do active work.
2	In bed <50% of the time. Ambulatory and capable of all self-care, but unable to carry out any work activities. Up and about more than 50% of waking hours.	60	Requires occasional assistance, but is able to care for most of his/her needs.
		50	Requires considerable assistance and frequent medical care.
3	In bed >50% of the time. Capable of only limited self-care, confined to bed or chair more than 50% of waking hours.	40	Disabled, requires special care and assistance.
		30	Severely disabled, hospitalization indicated. Death not imminent.
4	100% bedridden. Completely disabled. Cannot carry on any self-care. Totally confined to bed or chair.	20	Very sick, hospitalization indicated. Death not imminent.
		10	Moribund, fatal processes progressing rapidly.
5	Dead.	0	Dead.

APPENDIX B: CTEP MULTICENTER GUIDELINES

NOT APPLICABLE

APPENDIX C: Pharmacokinetic, Pharmacodynamic, and Pharmacogenomic sample collection

1.) Dose Escalation Cohort

Cycle #	Study Day	Treatment	Sample collection protocol time	Amt of Blood Drawn in mL	Biopsy[1]
N/A	N/A	N/A	Prior to registration	10 ml (PG[6])	
1	1	CPT-11 single agent infusion	Pre- infusion	8 (PAR[3])<br>8 (PK-PBMC[4, 5])	
1	1		30 min after start of infusion	4 (PK[4])	
1	1		Immediately at the EOI[2] (~89 min)	4 (PK[4])	
1	1		2 h after start of infusion	4 (PK[4])	
1	1		3.5 h after start of infusion	8 (PAR[3], PK[4])	
1	1		5.5 h after start of infusion	8 (PAR[3])<br>8 (PK-PBMC[4, 5])	
1	1		8.5 h after start of infusion	8 (PAR[3], PK[4])	
1	2		28 h after start of infusion	8 (PAR[3])<br>8 (PK-PBMC[4, 5])	Biopsy[1]
1	3		48 h after start of infusion	8 (PAR[3], PK[4])	
1	8	CPT-11 infusion + ABT888 p.o.	Pre- infusion/ABT-888 dosing	8 (PAR[3])	
1	8		3.5 h after start of infusion/ABT-888 dosing	8 (PAR[3])	
1	8		5.5 h after start of infusion/ABT-888 dosing	8 (PAR[3])	
1	8		8.5 h after start of infusion/ABT-888 dosing	8 (PAR[3])	
1	9		28 h after start of infusion/ABT-888 dosing	8 (PAR[3])	Biopsy[1]
1	10		48 h after start of infusion/ABT-888 dosing	8 (PAR[3])	
2	-1	ABT888 oral administration	Pre-ABT-888 dosing	8 (PAR[3], PK[4])	
2	-1		30 min after ABT-888 dosing	4 (PK[4])	
2	-1		1 h after ABT-888 dosing	4 (PK[4])	
2	-1		1.5 h after ABT-888 dosing	4 (PK[4])	
2	-1		3.5 h after ABT-888 dosing	4 (PK[4])	
2	-1		5.5 h after ABT-888 dosing	8 (PK-PBMC[4, 5])	
2	-1		8.5 h after ABT-888 dosing	4 (PK[4])	
2	-1		10 h after ABT-888 dosing	4 (PK[4])	
2	1		28 h after ABT-888 dosing	8 (PK-PBMC[4, 5])	
2	8	CPT-11 infusion + ABT888 p.o.	Pre- infusion/ABT-888 dosing	8 (PAR[3], PK[4])	
2	8		30 min after start of infusion/ABT-888 dosing	4 (PK[4])	
2	8		1 h after start of infusion/ABT-888 dosing	4 (PK[4])	
2	8		Immediately at the EOI[2] (~89 min)	4 (PK[4])	
2	8		2 h after start of infusion/ABT-888 dosing	4 (PK[4])	
2	8		3.5 h after start of infusion/ABT-888 dosing	4 (PK[4])	
2	8		5.5 h after start of infusion/ABT-888 dosing	8 (PAR[3], PK[4])	
2	8		8.5 h after start of infusion/ABT-888 dosing	4 (PK[4,])	
2	8		10 h after start of infusion (prior to admin. of second dose of ABT-888 on day 8)	4 (PK[4])	
2	9		28 h after start of infusion (prior to ABT-888 administration on day 9)	8 (PAR[3], PK[4])	
2	10		48 h after start of infusion (prior to ABT-888 administration on day 10)	4 (PK[4])	

[1] First biopsy will occur 28 hours after beginning of the Day 1, Cycle 1 infusion of single agent irinotecan; second biopsy will occur 28 hours after beginning of the Day 8, Cycle 1 infusion of irinotecan and 4 hrs after the Day 9, Cycle 1 dose of ABT-888.
[2] EOI – end of infusion
[3] PAR – 8 ml blood collection will occur for separation of PBMC for use in PAR assay, see protocol in Appendix D
[4] PK – 4 ml or 8 ml blood sample collection will occur for separation of plasma for use in pharmacokinetic (PK) assay
[5] PBMC will be separated for use in pharmacodynamic (PD) assays, use protocol in Appendix G to isolate PBMCs

[6] 10ml blood draw anytime prior to study registration for pharmacogenomic (PG) analysis to determine polymorphisms in UGT1A1, CYP2C9, CYP2C19, & ABCG2

2. Expansion Cohort

Cycle #	Study Day	Treatment	Sample collection protocol time	Amt of Blood Drawn in mL	Biopsy[1]
N/A	N/A	N/A	Prior to treatment	8 ml (PBMC[2])<br>8 ml (CTC[3])<br>10-12 ml (genomic analysis)[4]	Biopsy[1]<br>Archival[5]
1	1	CPT-11 single agent infusion	Pre- infusion	8 (PBMC[2])<br>8 (CTC[3])	
1	1		4-6 hr after start of infusion	8 (PBMC[2])<br>8 (CTC[3])	Biopsy[1]
1	1		8 h after start of infusion	8 (PBMC[2])<br>8 (CTC[3])	
1	2		22 h after start of infusion	8 (PBMC[2])<br>8 (CTC[3])	
1	2		24 h after start of infusion	8 (PBMC[2])<br>8 (CTC[3])	
1	8	CPT-11 infusion + ABT888 p.o.	Pre- infusion/ABT-888 dosing	8 (PBMC[2])<br>8 (CTC[3])	
1	8		4-6 h after start of infusion/ABT-888 dosing	8 (PBMC[2])<br>8 (CTC[3])	Biopsy[1]
1	8		8 h after start of infusion/ABT-888 dosing	8 (PBMC[2])<br>8 (CTC[3])	
1	9		22 h after start of infusion/ABT-888 dosing	8 (PBMC[2])<br>8 (CTC[3])	
1	9		24 h after start of infusion/ABT-888 dosing	8 (PBMC[2])<br>8 (CTC[3])	
1	15	ABT888 oral administration	4 hrs after the first Day 15 dose of ABT-888 scheduled that day	8 (CTC[3])	

[1] First biopsy will occur within 2 weeks of first treatment; second biopsy will occur 4-6 hours after beginning of the Day 1, Cycle 1 infusion of single agent irinotecan; third biopsy will occur 4-6 hours after beginning of the Day 8, Cycle 1 infusion of irinotecan. All biopsies will consist of 4-6 passes with a 16 or 18 gauge needle.

[2] PBMC – 8 ml blood collection will occur for separation of PBMC for PD evaluation by the NCTVL, see protocol in Appendix D

[3] CTC - 8 ml blood collection will occur for CTC assay, see protocol in Appendix J

[4] Genomics - 1-2 acid citrate dextrose (ACD) yellow top collection tubes with 10-20mls of peripheral blood using standard blood collection techniques for sequencing of somatic mutations.

[5]Archival tissue block or paraffin sample from archival tissue block (approximately 5-10 sections)

APPENDIX D: Standard Operating Procedure (SOP) for Collection and Preparation of PBMC samples

The following SOP has been modified from the NCTVL SOP 340503 version 2.22.2007.

1.0 PURPOSE

To standardize the method for preparing PBMC samples from blood for Pharmacodynamic (PD) study of ABT-888.

2.0 SCOPE

This procedure applies to all personnel in the NCTVL involved in the PBMC sample preparation for PD study of ABT-888.

3.0 RESPONSIBILITIES

3.1. It is the responsibility of the Lab Supervisor/Manager to assure that all personnel are properly trained and qualified and their training is documented prior to being allowed to work on this procedure.

3.2. It is the responsibility of the Lab Supervisor to confirm scheduled specimen collection time points, to print all the labels in advance, and to check all the labels for accuracy.

3.3. It is the responsibility of the Lab Supervisor to print all data collection sheets in advance, review data entry, and archive all data sheets in the appropriate files.

3.4. It is the responsibility of each lab technician involved to follow this procedure and complete the required lab tasks and associated documentations including NCTVL label sheet, batch record and database.

4.0 MATERIALS & EQUIPMENT REQUIRED

4.1. Sorvall Legend RT centrifuge (Fisher # 75-004-377)

4.2. Sorvall Fresco centrifuge (Fisher #75005524/01)

4.3. Ice bucket

4.4. Pipettors (1000 µl, 200µl, 20µl) and tips

4.5. Electronic pipette

4.6. 3-ml Falcon transfer pipet (Fisher # 13-711-6)

4.7. 2.0-ml Sarstedt tubes (Fisher # 72.694.006)

4.8. 15-ml polypropylene tubes (Fisher # 14-959-49B)

4.9. BD Vacutainer CPT tubes (8 mL blue top tube, BD REF 362761, or 2 of 4 ml tube with REF 362760)

4.10. Plasma-Lyte A pH 7.4, USP (Baxter Healthcare Corporation; NDC 0338-631703 or NDC 0338-6317-04)

4.11. 100% ethanol

4.12. Dry ice

4.13. Hemacytometer

4.14. Trypan Blue, 0.4%, sterile (StemCell Technologies Inc, Cat #07050)

4.15. 81-cell chipboard storage boxes (Fisher # 12-565-182)

4.16. -80°C freezer

4.17. Thermoflask cooler

5.0 OPERATING PROCEDURES

Note: Assure that the phlebotomist is using a BD Vacutainer CPT to draw the blood sample. Assure that the anticoagulant being used (sodium citrate or sodium heparin) is correct for the intended use of the specimen and that the tube size is correct for the blood draw volume required by the protocol. Supply phlebotomy with a correct CPT tube, if necessary.

5.1. Print out two sets of BSI labels for each time point as defined by the PD study protocol, including protocol #, trial site if not NIH, patient ID, specimen type, dose level, cycle and time-point of pre-dose or post-dose. One set is for the assigned research nurse (RN) to use at the Clinical Center and another set is for NCTVL personnel to use at specimen processing lab.

5.2. Arrive at hospital site about 5 min ahead of the scheduled time points to make sure picking up samples on time.

5.3. Place one BSI label onto sample tube (8 ml) and one BSI label onto the RN sheet.

5.4. Transport sample at RT with double container from hospital to sample processing lab (Bldg. 10 Room 3-5360).

5.5. Mix the sample by inverting the tube for 5-8 times and centrifuge the sample at 1500 g for 30 min at 18-25°C, without break.

5.6. Place one BSI label onto the NCTVL sheet and place one BSI label onto a 15-ml conical tube.

5.7. After centrifuge, remove two thirds of plasma and transfer whitish PBMC layer into the labeled 15-ml conical tube using a 3-ml Falcon transfer pipet. Discard the Vacutainer CPT tube in biohazard waste container.

5.8. Add Plasma-Lyte A USP to the 15-ml tube to bring total volume to 14 ml, cap and mix by gentle inversion 5-8 times

5.9. Centrifuge the sample at 430 g for 10 min at 18-25°C, without break.

5.10. Using a new transfer pipet, aspirate as much supernatant as possible without disturbing the cell pellet and discard the supernatant into biohazardous liquid waste.

5.11. Re-suspend the cell pellet in 6 ml of Plasma-Lyte A USP by gently flicking the bottom of the tube with the index finger and then gently triturating 5 times using a 5 ml pipet.

5.12. Immediately after resuspending the cell pellet, transfer 20 ul of sample into a tube containing 60 ul Plasma-Lyte A and 20 ul of 0.4% Trypan, and set aside for a cell count.

5.13. Centrifuge the remaining 5.98 ml cell suspension at 430 g for 10 min at 18-25°C, without break. While centrifuging, incubate the Trypan Blue sample for 2-5 minutes, and then perform a total and viable cell count using a hemacytometer (SOP340005 **Determining Cell Count/Viability Using a Hemocytometer**). Record the following information into the Batch Record:

    5.14.1. Actual total cell counts in each hemacytometer square that was counted.

    5.14.2. Actual viable cell counts in each hemacytometer square that was counted.

    5.14.3. Calculated viable cell concentration in the 6.0 ml cell suspension.

    5.14.4. The viable cell yield in the remaining 5.98 ml cell suspension.

    5.14.5. The calculated volume required to make a suspension of 3×10^6 cells/ml.

5.14. Remove and discard supernatant without disturbing the cell pellet.

5.15. Re-suspend the cell pellet in the volume of Plasma-Lyte A USP calculated to yield a cell suspension containing 3 x 10^6 viable PBMCs per ml. Re-suspend the cell pellet by gently flicking the bottom of the tube with the index finger and then gently triturating 5 times using a 2 ml pipet for volumes of 2.5 ml or less, or a 5 ml pipet for volumes greater than 2.5 ml.

5.16. Transfer 1.0 ml aliquots of the PBMC cell suspension into individual 2.0-ml cryovials until the remaining volume of cell suspension is less than 1.0 ml. Record the number of 2.0-ml cryovials that have been prepared into the Batch Record. If at least 0.2 ml of residual volume of cell suspension remains in the BD Falcon tube, use a 1.0-ml pipet to measure its volume accurately (without any bubbles) while transferring it into a 1.5-ml cryovial. Mark the top of the 1.5-ml cryovial with a black Sharpie pen and write “partial” on the cryovial. Record the preparation of the single 1.5-ml cryovial in the Batch Record.

5.17. Centrifuge the cryovials in Sorvall Fresco centrifuge at 2000 x g for 10 minutes at 4-10°C.

5.18. Remove and discard as much supernatant as possible without disturbing the cell pellet.

5.19. Place BSI label onto each cryovial aliquot.

5.20. Snap freeze the cryovials containing the cell pellets using liquid nitrogen or a dry-ice/ethanol bath.

5.21. Store the frozen PBMC samples at -80°C until analysis or ship out.

5.22. Complete Batch Record and file the document after having the Lab Supervisor reviewed and signed.

5.23. Make sure to record shipping time onto the NCTVL label sheet when the specimens were shipped out.

6.0 SHIPPING PROCEDURES

6.1. Send an email in advance to advise recipient of scheduled shipping time and ask the recipient to reply the email for sample receiving conformation.

6.2. Generate a shipping list containing all the specimen records by using the shipping list template in excel file.

6.3. Verify the BSI ID, sample volume (if apply) and other information on shipping list against the samples to be shipped for accuracy.

6.4. Place sample tubes/vials for each patient in watertight biohazard specimen bags. Place the specimen bags in a shipping container with sufficient dry ice to maintain the samples in frozen state for at least 8 hours.

6.5. Label the container as biohazard specimens.
Ship the specimen to:

For PBMC blood samples:
[redacted], PhD
National Clinical Target Validation Lab
DTP, DCTD, NCI,
37 Convent Dr., [redacted]
Bethesda, MD 20892
[redacted]

BATCH RECORD

Operator: ___________________________ Date: ____________________________

Lot numbers:
CPT Vacutainer Product # and lot #:____

Plasma Lyte A USP lot # and expiration date: ______________________________________

15 ml polypropylene tubes (circle one): #352097 #352196 #352096 or Lot #: _____________

Cryovial lot #:____

Trypan Blue lot #_________________________ Dilution vials______________________

Serial numbers of equipment:
P-100 Pipetman: P-1000 Pipetman:

NOTES; Record times using military time (24-hour designation), for example 16:15 to indicate 4:15 pm Create a new Batch Record for each patient that contributes CPT specimens

Sample ID: ____

Study project ID: ____

Blood Volume ______________________ Time of Venous Blood Draw: ________________

Time Lab Processing Begins: ____

Time of PBMC Transfer into Plasma Lyte A: ____

Time of Cell Counts in Hemacytometer: ____

Record the following data:

Total cell counts in each hemacytometer square counted: ________________________

____

Viable cell counts in each hemacytometer square counted: ________________________

____

Hemacytometer dilution factor: ____

Calculated viable cell concentration in suspension:______________________________

Viable cell yield remaining in the 5.98 cc cell suspension: ________________________

Volume used to resuspend cell pellet to 3 x 10^6 viable PBMCs per ml:______________

Sample ID from page 1: ____

CHOOSE ONE of the following actions by checking the appropriate box:

☐ For PBMC yields of > 3 x 10^6 cells (>1.0 ml of cell suspension):

Number of 2.0 ml cryovials with 1.0 ml cell suspension: _______ Location: _____________

Number of PBMCs per 2.0 ml cryovial: _____3.0 x 10^6_______

☐ For PBMC yields of ≤ 3 x 10^6 cells (≤1.0 ml of cell suspension):

Number of 1.5 ml cryovials with <1.0 ml cell suspension: ______ Location:______________

Volume of PBMC suspension added per 1.5 ml cryovial: ________ ml

Number of PBMCs per 1.5 ml cryovial: ____

Time cryovials placed into -80°C storage: ______________________________________

Date/time sample information entered into NCTVL database: _______________________

Notes about this PBMC Preparation, including any deviations from the SOP:

APPENDIX E: Standard Operating Procedure (SOP) for Collection and Preparation of Tumor Biopsy samples for PAR Immunoassay and DNA-damage Response PD Assays

The following SOP has been modified from the NCTVL SOP 340507 version dated 2/22/2007.

1.0 PURPOSE

To standardize the method for collecting and processing tumor biopsy for Phase-1 trial of ABT888.

2.0 SCOPE

This procedure applies to all personnel in the NCTVL involved in the tumor biopsy specimen collection and processing. Tumor needle biopsies are collected under the assistance of image guidance (Radio-Oncology Interventional Radiology). Both core needle biopsies will be cryo-preserved immediately. Cryopreserved biopsies are stored at -80°C for further lab processing.

3.0 RESPONSIBILITIES

3.1. It is the responsibility of the Lab Supervisor to assure that all personnel are properly trained and qualified and their training is documented prior to being allowed to work on this procedure.

3.2. It is the responsibility of the Lab Supervisor to confirm scheduled specimen collection time points, to print all the labels in advance, and to check all the labels for accuracy.

3.3. It is the responsibility of the Lab Supervisor/Manager to print all data collection sheets in advance, review data entry, and archive all data sheets in the appropriate files.

3.4. It is the responsibility of each lab staff member who performs tasks to follow this procedure and complete the batch record.

3.5. It is the responsibility of each lab staff member who is assigned duties in the Interventional Radiology suite to arrive 10 minutes prior to the scheduled procedure.

4.0 MATERIALS & EQUIPMENT REQUIRED

4.1. Liquid nitrogen (LN_2)

4.2. Sterile, disposable tweezers (Fisher #NC9566754)

4.3. 2.0-mL cryogenic vials placed on dry ice in a small insulated bucket (Fisher #72.694.006)

4.4. 15-mL conical tubes (Fisher #14-959-49B)

4.5. 50-mL conical tubes (Fisher #14-432-22)

4.6. Laptop computer with BSI specimen processing data sheet

4.7. BSI sample ID barcode scanner with date and time function

4.8. Cell chipboard storage boxes (Fisher #12-565-182)

4.9. -80°C freezer

4.10. Thermoflask cooler

4.11. Bucket with ice

5.0 OPERATING PROCEDURES

5.1. Print out two sets of BSI labels for each biopsy in advance. One set is for the assigned research nurse (RN) to use in the study chart of the particular patient at the WSU/UMGCC/DFCI Clinics and another set for laboratory personnel to use at specimen collection and processing.

5.2. Send an email in advance to advice recipients (e.g., pharmacodynamic assay site, pathology service, project manager, etc) of scheduled biopsy and the estimated specimen processing time and estimated shipping time. It is critical that the tumor biopsies are to be transferred to the laboratories for immediate processing (cryopreservation for the NCI and preparation of cytospins, cryosections or pellets within 1 hour for the WSU translational research lab, see appendix F).

5.3. Prepare and bring necessary lab supplies and reagents including one 50-mL conical tube containing 2 mL PBS , Thermoflask cooler containing approximately 2/3 full LN_2, microscope slide box in a ziplock bag with new desiccant cartridge, six tweezers, four 1.5-mL cryogenic vials, laptop computer and BSI ID scanner.

5.4. Arrive at Interventional Radiology Special Procedures suite about 10 min ahead of the scheduled procedure giving time to set up lab supplies, reagents, laptop computer and scanner ready for biopsy specimen processing and data recording on time.

5.5. Place one BSI label onto each slide and sample tube and one of each BSI label onto the RN sheet. BSI label should include protocol #, trial site, patient ID, specimen type, dose level, cycle and time-point of pre-dose or post-dose.

5.6. Document appropriate BSI label into the trial laboratory notebook. Include:

5.7.1. Local anesthesia administered. Please note: there is a possibility that epinephrine in small quantities may interfere with the PAR assay. Therefore, the local anesthesia used for tumor biopsies should be **only** lidocaine whenever possible and case report forms should specify what agents (i.e., lidocaine alone or lidocaine plus epinephrine) were used for each biopsy procedure.
5.7.2. Skin incision
5.7.3. Guide needle introduced
5.7.4. Guide needle placement confirmed
5.7.5. 1st biopsy needle introduced
5.7.6. 1st biopsy transferred into tube with LN_2
5.7.7. 2nd biopsy needle introduced
5.7.8. 2nd biopsy transferred into tube with LN_2
5.7.9. Return to the WSU/UMGCC/DFCI specimen processing site
5.7.10. Cryopreserved biopsies placed at -80°C.

5.8. Transfer the core biopsy specimen into a labeled 1.5-mL cryogenic vial that is pre-chilled in dry ice, and immediately drop the vial with specimen into LN_2. Hold the biopsy with the sterile tweezers at one end, and touch the opposite end of the biopsy to the prechilled tube. This should attach the tissue to the tube, allowing it to be dropped into the tube while releasing the tissue from the tweezers without sticking. Dispose of the tweezers into medical waste for sharps. Immediately drop the vial into LN_2 once tissue is transferred.

5.9. Transport all biopsy specimens with double container from surgical suite to sample processing lab.

5.10. Transfer all cryopreserved biopsy specimens from LN_2 to -80°C and store until shipped out as directed by Lab Manager.

5.11. Send an e-mail to NCTVL (NCINCTVL@mail.NIH.gov) prior to shipping to advise recipient of scheduled shipping time. Be sure to request and receive a confirmation e-mail from NCTVL prior to shipping.

5.12. Generate a shipping list containing all the specimen records by using the Shipping Manifest template as shown below.

5.13. Place specimen tubes into an 81-place freezer box and then in a shipping container with sufficient dry ice to maintain the samples at -20ºC for at least 72 h. All weekly processing specimens are recommended to ship out via FedEx on the following Monday afternoon for delivery by 10 AM Tuesday (FedEx First Overnight).

5.14. Be sure to **check off** EACH specimen being shipped to NCTVL and verify the contents of the package match the Shipping Manifest.

5.15. Print and attach the shipping address onto the outside of the shipping container and be sure the container is labeled as containing biohazardous specimens.

Attn: [redacted] PhD
National Clinical Target Validation Laboratory (NCTVL)
DTP, DCTD, National Cancer Institute
37 Convent Drive
[redacted]
Bethesda, MD 20892
Phone: [redacted]

5.16. Record the shipping time in the Batch Record (below).

5.17 Ship the specimens with a copy of the Shipping Manifest and the completed Batch Records for all specimens. Retain copies of the completed Shipping Manifest(s) and Batch Records in your records.

5.18 E-mail NCTVL (NCINCTVL@mail.NIH.gov) a shipment notification. State "*Protocol Name* PD Specimen Shipment" in the subject line and reference the tracking number in the e-mail.

BATCH RECORD

(Page 1 of 1)

Operator: ____________________________ Date: _____________________________

Equipment ID or Serial Numbers:

-80°C freezer: ____

Processing Records:

Project: ABT-888 Phase I

Patient ID:

Needle Type: ___________________ Needle diameter: ______ gauge; and length: _______ cm

Sample ID: ______________________Pre dose: ____________ or Post dose: _____________

Time local anesthesia administered_________________ Time of skin incision_____________

Time guide needle introduced: ________Time guide needle placement confirmed: _________

Time of 1st biopsy needle introduced: ____

Time of 1st biopsy specimen transferred into N_2: ____________________________________

Time of 2nd biopsy needle introduced: ____

Time of 2nd biopsy specimen transferred into N_2: ____________________________________

Time of return to UMGCC/WSU/DFCI specimen processing site: _______________________

Time of biopsy specimens placed at -80°C: ____

Name(s) of local anesthetic used (from Res Study Nurse): ______

Dose of local anesthetic: ___ mg

Notes:

Reviewed by: ________________________ Date: _____________________________

TISSUE SPECIMEN COLLECTION AND SHIPPING

1. Sample Pre-printed Label for Tissue Samples

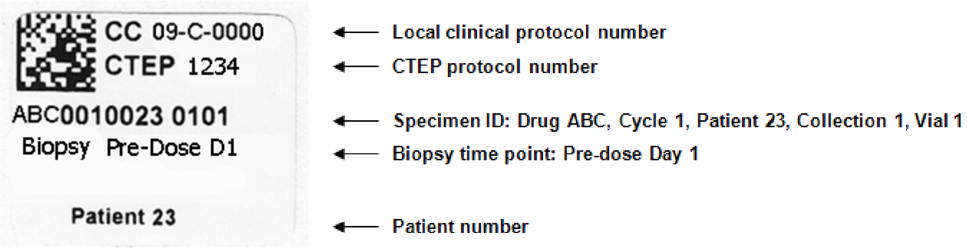

2. Sample Shipping Manifest

**Ship From:**<br>**Laboratory PI**<br>**Address**<br>**Contact Name**<br>**Contact Phone**	**NCTVL**<br>**Shipping Manifest**	**Ship To:**<br>**Attn:** [redacted] PhD<br>NCTVL/DTP/DCTD/NCI<br>B[redacted]<br>Bethesda, MD 20892<br>**Phone:** [redacted]<br>**Email:** NCINCTVL@mail.NIH.gov
**Shipping Date:**<br>**Clinical Protocol:**	**Carrier:**<br>**Shipping #:**	

In Package	#	Patient ID	Sample ID	Description	Collection Time Point	Collection Time	Collection Date MM/DD/YY
☐	*Test*	*21*	*XZ00100260201*	*Tumor*	*Pre-Dose D1*	*14:15*	*03/01/10*
☐	1						
☐	2						
☐	3						
☐	4						
☐	5						
☐	6						
☐	7						
☐	8						
☐	9						
☐	10						

APPENDIX F: Processing of 2nd Biopsy (Dose Escalation Portion ONLY)

A. Portioning of Biopsy

1. Remove the biopsy from the PBS and rinse it carefully with PBS. Put the biopsy in a petri dish (100 mm) that is placed on ice. Save the PBS. If sufficient biopsy tissue is available, cut the core into 3 pieces. Place one piece into a 1.5 cm cryovial and flash freeze in liquid N2. Place a second piece into a 1.5 ml vial with 200 µL OTC and store both pieces at -80°C. The biopsy tissue in OTC is later used for cryosectioning, the other for RNA or protein extraction. Directions for the third piece is below. If there is insufficient tissue for these 3 pieces, eliminate the OTC frozen piece.

2. Mince the third piece of the biopsy tissue with two scalpels. The tissue will look almost liquid but it will still have chunks of tissue in it. Transfer the homogenate to a 15 ml centrifuge tube using 2-3 ml PBS. Use several small aliquots of PBS in multiple transfers to make sure you transfer all of the tissue. Vortex gently to further break up the chunks. Allow the chunks to settle out for about 15-30 sec and transfer liquid to a clean tube. Save the chunks in case a second attempt is required. Centrifuge at 400 X g for 10 min. Remove supernatant and resuspend in 400 µl PBS. Proceed with cytospin slide preparation.

3. Instead of preparing cytospins from the third piece of the biopsy tissues, it is acceptable to embed it into OTC and cut frozen sections instead.

B. Preparation of cytospins

1. Basic protocol:

Prepare a single cell suspension of not more than 0.5 x 10^6 cells / ml of protein-containing medium.
Pre-label the slides using a pencil.

Procedure for old type cytocentrifuge (be careful, this is not biohazard proof!!)

Prepare the slides mounted with the paper pad and the cuvette in the metal holder.

click to enlarge the image

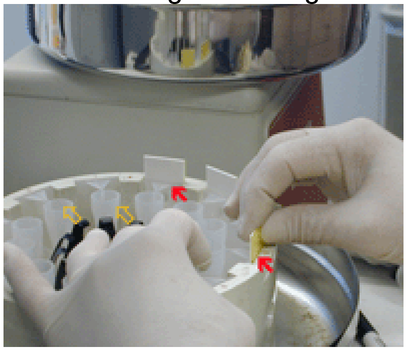

Load up to 200 µl of this suspension in each cuvette.
Spin at 800 rpm for 3 min.
Extract the slide, paper and cuvette without disarranging.
Carefully detach the cuvette and the paper without damaging the fresh cytospin. Is very important to hold firmly together glass slide and cuvette when extracting from metal holder.
Mark the area around the cytocentrifuged cells with dry point or permanent marker.
Proceed with either immediate fixation or drying. Store unfixed cytospins for max 2 days at room

temperature.

If you have little cells:
As few as 5 x 104 cells can be cytospun.

Proceed as follows:
Prepare the cell suspension with 10% FCS.
Pre run the loaded cytocentrifuge with 50 µl medium + serum, in order to wet the paper pads.

Run your sample with not fewer than 100 µl and not more than 250 µl.

Provider of cytospin material:

Shandon Inc. , 171 Industry Drive, Pittsburgh, PA 15275.

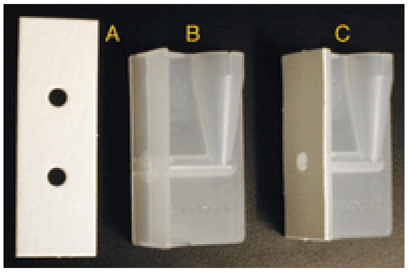

A: Shandon cards Cat n° 190005
B: re-used cuvette with glued paper residues removed.
C: Shandon pre-made cuvettes Cat n° 5991040 (box of 50).

2. Air dry the slides for 5 min.

3. Prepare a mixture (day before) of methanol and acetone at a ratio of 1:1 and put in to -20°C freezer to cool the mix down.

4. Fix the cells and permeabilize them by putting them 3 x for 1 min. each into the ice-cold methanol:acetone mixture. Use Copland jars for this step. After each 1 min. fixation let the slide dry on air before putting into a fresh lot of methanol:acetone.

5. Let the slide then air dry for 15 min. At this step the slide can be wrapped into cling wrap and stored at -20°C or directly processed further (as in 6.).

6. The slide is then re-hydrated for 10 min. in PBS and blocked for at least 45 min. with a solution of 5% BSA in PBS.

7. Now the primary antibody is added in PBS at its recommended dilution for immunofluorescence. The antibody is added for 2 hours up to over night in a moist chamber (e.g. box with lid and wet tissue).

8. Then the slide is washed 3 times in PBS (Copland jar) and the secondary antibody containing FITC (or TRITC) is added in a dil. of 1:200 (for FITC) for 3 hours.

9. The slide is washed again very thoroughly, for up to 5 times in PBS and then incubated with DAPI (2mg./ml stock) at 1:7500 dilution in PBS for 5 min. in the dark.

10. The slide is washed again for 5 times in PBS and then coverslipped with a solution that does contain anti-fading substance (e.g. VectaShield antifade). The slide can now be viewed and pictures taken.

11. From here on the slide has to be kept in the dark (aluminum foil) and can be stored in a moist chamber for several days.

Sample shipping:
Shipments should only be made Monday through Wednesday. Prior to any shipment, please notify the KCI Pharmacology Laboratory via email and/or telephone. The samples will be shipped to the following address:

Attention: [redacted]
Barbara Ann Karmanos Cancer Institute
Wayne State University
4100 John R, HW05AO
[redacted]
Detroit, MI 48201
Office: [redacted]
Fax: [redacted]
Email: [redacted]

APPENDIX G: Pharmacokinetic Worksheet

A Phase I Dose-Escalation Study of Oral ABT-888 (NSC #737664) Plus Intravenous Irinotecan (CPT-11, NSC#616348) Administered in Patients with Advanced Solid Tumors (Dose Escalation Portion ONLY)

Pharmacokinetic Worksheet

Subject Initials: _______________ **Study ID #:** _______________

History #: _______________

- **Label All Tubes with Protocol #, Sample Type, Patient Initials, Patient #, Protocol Time, and Actual Date and Time of sample.**
- **Please phone the Pharmacology Core Lab (313-576-8244) As Soon As Possible For Sample Processing.**

PK Samples:

At the time point specified above, **if 4 ml blood sample is collected**, the sample will be processed as the below procedure to separate plasma for PK assay only:

4 ml blood sample will be collected into a tube with sodium citrate anticoagulant. The actual day and time of sample collection will be recorded on the PK worksheet (see Appendix G). Within 1 h of the collection, the blood sample will be centrifuged at 25^0C, at 3000 rpm for 10 min, and plasma will be collected immediately after centrifugation and transferred to 4 screw-cap polypropylene cryogenic tubes (4 aliquots). The tube will be labeled with the patient's initials, study number, sample collection day/time, and frozen at ≤ -70^0C until analysis.

PK-PBMC samples:

At the time point specified above, **if 8 ml blood sample is collected**, the sample will be processed as the below procedure to separate plasma (for PK assay) and PBMC (for PD assays). For PBMCs isolated for the PAR assay, please see Appendix D.

8 ml blood sample will be collected into 8-ml BD Vacutainer® CPT™ Cell Preparation Tube with sodium citrate anticoagulant (BD REF No. 362761, Franklin Lakes, NJ), and put upright at room temperature until centrifuge. The actual day and time of sample collection will be recorded on the PK worksheet. Within 1 h of the collection, the sample will be mixed by inverting the tube for 5-8 times and centrifuged at 1500 g for 30 min at 18-25°C. Two thirds of plasma will be collected immediately after centrifugation and transferred to four screw-cap polypropylene cryogenic tubes (four aliquots), and freeze at -70^0C or below until analysis of ABT-888 and/or irinotecan concentration. Whitish PBMC layer (middle layer) will be transferred into the labeled 15-ml conical centrifuge tube with cap. The PBMC will be washed twice, and viable cell number will be counted. The cell pellet will be re-suspended to yield a cell suspension containing 3×10^6 viable PBMCs per ml. 0.5 ml aliquots of PBMC cell suspension will be transferred into 2.0-ml cryovials, and centrifuged at 2000 x g for 10 minutes at 4-10°C. The resulted cell pellets will be frozen at -80°C until shipped out.

Protocol #:

Subject Initials: ________________ **Study ID #: ________________**

Cycle 1, day 1	CPT-11 PK Sample Data

CPT-11 IV Infusion Start Date / Time:
____

CPT-11 IV Infusion Stop Date / Time:
____

CPT-11 infusion dose: ______________________

Infusion Site: _____________________________

Protocol Sample Time	Sample(s) Due Date	Actual Sample Time	Blood volume (ml)	Blood Draw Initials	Comments
Pre- infusion			8		
30 min after start of infusion			4		
Immediately at the end of infusion (EOI) (~89 min)			4		
2 h after start of infusion			4		
3.5 h after start of infusion			8		
5.5 h after start of infusion			8		
8.5 h after start of infusion			8		
28 h after start of infusion			8		
48 h after start of infusion			8		

Protocol #:

Subject Initials: _______________ **Study ID #: ________________**

Cycle 2, day -1	ABT888 PK Sample Data

ABT888 oral administration Date / Time: ____________________

ABT888 dose: ______________________

Site: ____________________________

(Note: A single dose of ABT888 (half of the total daily dose) is administered on Cycle 2, day -1)
***(Note: On Cycle 2, Day 1 first daily dose of ABT-888 should be taken after 28 hr blood draw is complete**

Protocol Sample Time	Sample(s) Due Date	Actual Sample Time	Blood volume (ml)	Blood Draw Initials	Comments
**Pre-dosing**			8		
**30 min after dosing**			4		
**1 h after dosing**			4		
**1.5 h after dosing**			4		
**3.5 h after dosing**			4		
**5.5 h after dosing**			8		
**8.5 h after dosing**			4		
**10 h after dosing**			4		
***28 h after dosing**			8		

Protocol #:

Subject Initials: _______________ **Study ID #:** _______________

**Cycle 2, day 8**	**CPT-11 and ABT888 combination PK Sample Data**

CPT-11 IV Infusion Start Date / Time: ______________________

CPT-11 IV Infusion Stop Date / Time: ______________________

CPT-11 infusion dose: _______________________

Infusion Site: ______________________________

ABT888 oral administration Date / Time: ____________________

ABT888 dose: _______________________

(Note: The first dose of ABT-888 to be administered on day 8 will be taken at the start of CPT-11 infusion, so PK sampling can be lined up for both drugs.)

**Protocol Sample Time**	**Sample(s) Due Date**	**Actual Sample Time**	**Blood volume (ml)**	**Blood Draw Initials**	**Comments**
**Pre- infusion**			8		
**30 min after start of infusion**			4		
**1 h after start of infusion**			4		
**Immediately at the end of infusion (EOI) (~89 min)**			4		
**2 h after start of infusion**			4		
**3.5 h after start of infusion**			4		
**5.5 h after start of infusion**			8		
**8.5 h after start of infusion**			4		
**10 h after start of infusion** (prior to the administration of the second dose of ABT-888 on day 8)			4		
**28 h after start of infusion** (prior to first dose of ABT-888 administration on day 9)			8		
**48 h after start of infusion** (prior to first dose of ABT-888 administration on day 10)			4		

If you have drawn a blood sample and your initials are listed above,

please write your initials and print and sign your name below.

Initials	Print Name	Signature

Sample shipping:
Shipments should only be made Monday through Wednesday. Prior to any shipment, please notify the KCI Pharmacology Laboratory via email and/or telephone. The samples will be shipped to the following address:

Attention: [redacted]
Barbara Ann Karmanos Cancer Institute
Wayne State University
4100 John R, HW05AO
[redacted]
Detroit, MI 48201
Office: [redacted]
Fax: [redacted]
Email: [redacted]

APPENDIX H: Patient Pill Diaries

1. Cycle 1 (Dose Escalation Portion)
2. Cycle 2 (Dose Escalation Portion)
3. All Cycles Following Cycle 2 (Dose Escalation Portion)
4. Cycle 1 (Expansion Portion)
5. All Cycles Following Cycle 2 (Expansion Portion)
6. Run-In Portion (Dose Escalation for Intermittent ABT-888 Portion)
7. All Cycles Following Run-In (Dose Escalation for Intermittent ABT-888 Portion)

A Phase I Dose-Escalation Study of Oral ABT-888 (NSC #737664) Plus Intravenous Irinotecan (CPT-11, NSC#616348) Administered in Patients with Advanced Solid Tumors

Patient Initials (fml): | | | |

Start Date: | | | / | | | / | | | | |
mm/dd/yyyy

Accession #:

Patient Pill Diary – DOSE ESCALATION PORTION - Cycle 1

Symptom	Date											
1. Chest Pain												
2. Chills												
3. Constipation												
4. Cough												
5. Diarrhea												
6. Dizziness												
7. Fatigue (tired)												
8. Fever												
9. Flushing												
10. Insomnia												
11. Nausea												
12. Pain (J-Joint, M-Muscle, H-Head, O-Other)												
13. Rash												
14. Runny nose												
15. Shortness of breath												
16. Stomach ache												
17. Swelling												
18. Vomiting												
19. Other:												
20. Other:												
21. Other:												
**Times Study Drug Pill (ABT-888) Taken:**	**A.M.** * **P.M.**	**A.M.** **P.M.**	**A.M.** **P.M.**	**A.M.** **P.M.**	**A.M.** **P.M.**	**A.M.** **P.M.**	**A.M.** **P.M.**	**A.M.** **P.M.**	**A.M.** **P.M.**	**A.M.** **P.M.**	**A.M.** **P.M.**	**A.M.** **P.M.**

Please store pills at room temperature at 15° to 25° C (59° to 77° F); protect from heat and moisture.
Study drug pill (ABT-888) to be taken while in the clinic for this scheduled time
If a dose of study drug pill (ABT-888) is missed or vomited, please do **not** repeat the dose.

Research Assistant Signature/Date: ______________________

Version: 07/21/2008

Patient Signature/Date: ______________________

A Phase I Dose-Escalation Study of Oral ABT-888 (NSC #737664) Plus Intravenous Irinotecan (CPT-11, NSC#616348) Administered in Patients with Advanced Solid Tumors

Patient Initials (fml): | | |

Start Date: | | / | | / | | | |
mm/dd/yyyy

Accession #:

Patient Pill Diary – DOSE ESCALATION PORTION - Cycle 2

Symptom	Date														
1. Chest Pain															
2. Chills															
3. Constipation															
4. Cough															
5. Diarrhea															
6. Dizziness															
7. Fatigue (tired)															
8. Fever															
9. Flushing															
10. Insomnia															
11. Nausea															
12. Pain (J-Joint, M-Muscle, H-Head, O-Other)															
13. Rash															
14. Runny nose															
15. Shortness of breath															
16. Stomach ache															
17. Swelling															
18. Vomiting															
19. Other:															
20. Other:															
21. Other:															
**Times Study Drug Pills (ABT-888) Taken:**	**A.M.** *	**A.M.** **P.M.**	**A.M.** **P.M.**	**A.M.** **P.M.**	**A.M.** **P.M.**	**A.M.** **P.M.**	**A.M.** **P.M.**	**A.M.** **P.M.**	**A.M.** * **P.M.**	**A.M.** **P.M.**	**A.M.** **P.M.**	**A.M.** **P.M.**	**A.M.** **P.M.**	**A.M.** **P.M.**	**A.M.** **P.M.**

Please store pills at room temperature at 15° to 25° C (59° to 77° F); protect from heat and moisture.
Study drug (ABT-888) pill to be taken while in the clinic for this scheduled time
If a dose of study drug pill (ABT-888) is missed or vomited, please do **not** repeat the dose.

Research Assistant Signature/Date: ______________________

Version: 07/21/2008

Patient Signature/Date: ______________________

Patient Initials (fml): | | | |

Start Date: | | | / | | | / | | | | |
mm/dd/yyyy

Accession #:

Patient Pill Diary – DOSE ESCALATION PORTION - All Cycles After Cycle 2

Symptom	Date														
1. Chest Pain															
2. Chills															
3. Constipation															
4. Cough															
5. Diarrhea															
6. Dizziness															
7. Fatigue (tired)															
8. Fever															
9. Flushing															
10. Insomnia															
11. Nausea															
12. Pain (J-Joint, M-Muscle, H-Head, O-Other)															
13. Rash															
14. Runny nose															
15. Shortness of breath															
16. Stomach ache															
17. Swelling															
18. Vomiting															
19. Other:															
20. Other:															
21. Other:															
**Times Study Drug (ABT-888) Taken:**	**A.M.**<br>**P.M.**	**A.M.**<br>**P.M.**	**A.M.**<br>**P.M.**	**A.M.**<br>**P.M.**	**A.M.**<br>**P.M.**	**A.M.**<br>**P.M.**	**A.M.**<br>**P.M.**	**A.M.**<br>**P.M.**	**A.M.**<br>**P.M.**	**A.M.**<br>**P.M.**	**A.M.**<br>**P.M.**	**A.M.**<br>**P.M.**	**A.M.**<br>**P.M.**	**A.M.**<br>**P.M.**	**A.M.**<br>**P.M.**

Please store pills at room temperature at 15° to 25° C (59° to 77° F); protect from heat and moisture.

Research Assistant Signature/Date: ______________________________

Version: 07/21/2008

Patient Signature/Date: ______________________________

A Phase I Dose-Escalation Study of Oral ABT-888 (NSC #737664) Plus Intravenous Irinotecan (CPT-11, NSC#616348) Administered in Patients with Advanced Solid Tumors

Patient Initials (fml): | | | |

Start Date: | | | / | | | / | | | | |
mm/dd/yyyy

Accession #: ____________________

If a dose of study drug pill (ABT-888) is missed or vomited, please do **not** repeat the dose.

Research Assistant Signature/Date: ____________________

Version: 07/21/2008

Patient Signature/Date: ____________________

Patient Initials (fml): [ ][ ][ ]

Start Date: [ ][ ] / [ ][ ] / [ ][ ][ ][ ]
mm/dd/yyyy

Accession #: [ ]

Patient Pill Diary – EXPANSION PORTION - Cycle 1

Symptom	Date													
1. Chest Pain														
2. Chills														
3. Constipation														
4. Cough														
5. Diarrhea														
6. Dizziness														
7. Fatigue (tired)														
8. Fever														
9. Flushing														
10. Insomnia														
11. Nausea														
12. Pain (J-Joint, M-Muscle, H-Head, O-Other)														
13. Rash														
14. Runny nose														
15. Shortness of breath														
16. Stomach ache														
17. Swelling														
18. Vomiting														
19. Other:														
20. Other:														
21. Other:														
**Times Study Drug (ABT-888) Taken:**	**A.M.** * **P.M.**	**A.M.** **P.M.**	**A.M.** **P.M.**	**A.M.** **P.M.**	**A.M.** **P.M.**	**A.M.** **P.M.**	**A.M.** * **P.M.** *	**A.M.** * **P.M.**	**A.M.** **P.M.**	**A.M.** **P.M.**	**A.M.** **P.M.**	**A.M.** **P.M.**	**A.M.** **P.M.**	**A.M.** * **P.M.**

Please store pills at room temperature at 15° to 25° C (59° to 77° F); protect from heat and moisture.
If a dose of study drug pill (ABT-888) is missed or vomited, please do **not** repeat the dose.
* Study drug (ABT-888) pill to be taken while in the clinic for this scheduled time

Research Assistant Signature/Date: ______________________

Version: 07/21/2008

Patient Signature/Date: ______________________

Patient Initials (fml): | | | |

Start Date: __ __ / __ __ / __ __ __ __
mm/dd/yyyy

Accession #: ____________________

Patient Pill Diary – EXPANSION PORTION - All Cycles After Cycle 1

**Symptom**	**Date**														
1. Chest Pain															
2. Chills															
3. Constipation															
4. Cough															
5. Diarrhea															
6. Dizziness															
7. Fatigue (tired)															
8. Fever															
9. Flushing															
10. Insomnia															
11. Nausea															
12. Pain (J-Joint, M-Muscle, H-Head, O-Other)															
13. Rash															
14. Runny nose															
15. Shortness of breath															
16. Stomach ache															
17. Swelling															
18. Vomiting															
19. Other:															
20. Other:															
21. Other:															
**Times Study Drug (ABT-888) Taken:**	**A.M.**<br>**P.M.**	**A.M.**<br>**P.M.**	**A.M.**<br>**P.M.**	**A.M.**<br>**P.M.**	**A.M.**<br>**P.M.**	**A.M.**<br>**P.M.**	**A.M.**<br>**P.M.**	**A.M.**<br>**P.M.**	**A.M.**<br>**P.M.**	**A.M.**<br>**P.M.**	**A.M.**<br>**P.M.**	**A.M.**<br>**P.M.**	**A.M.**<br>**P.M.**	**A.M.**<br>**P.M.**	**A.M.**<br>**P.M.**

Please store pills at room temperature (at 15° to 25° C (59° to 77° F); protect from heat and moisture.

Research Assistant Signature/Date: ____________________

Version: 07/21/2008

Patient Signature/Date: ____________________

A Phase I Dose-Escalation Study of Oral ABT-888 (NSC #737664) Plus Intravenous Irinotecan (CPT-11, NSC#616348) Administered in Patients with Advanced Solid Tumors

Patient Initials (fml): | | |

Start Date: | | / | | / | | | |
mm/dd/yyyy

Accession #:

If a dose of study drug pill (ABT-888) is missed or vomited, please do **not** repeat the dose.

Research Assistant Signature/Date: ______________________

Version: 07/21/2008

Patient Signature/Date: ______________________

Patient Pill Diary for Dose Escalation for Intermittent ABT-888 Portion Run-In Portion

Patient Initials (FML):

Start Date (mm/dd/yyyy):

		/			/				

NCI Protocol #: 7977 | Local Protocol # 1410014852 | Version: 06/06/2018

Accession # ________________________

Cycle Number ______________________ Dose _______________

Research Assistant Signature: _________________________________

Date: __________________

Patient Signature: ____

Date: __________________

Please carefully read and follow these instructions:

- Bring this Pill Diary and any remaining ABT-888 pills to clinic visits so your doctor can make sure that you are taking the pills correctly.
- Store the pills at room temperature at 15° to 25° C (59° to 77° F). Protect the pills from heat and moisture.
- Your doctor will tell you what ABT-888 dose you will be taking. You will be taking ABT-888 for 4 days and then have a break. *You can take the pills with or without meals.*

  If you miss taking a pill on time (a dose is missed) or you vomit after taking the pill, please do **not** repeat the dose.
- In the boxes below, note the **date** and **time** (under A.M. and P.M.) for each day you take the ABT-888 study drug pills:

Day -14	Day -13	Day -12	Day -11
**A.M. (in clinic)**	**A.M.**	**A.M.**	**A.M. (in clinic)***
_____________	_____________	_____________	_____________
**P.M.**	**P.M.**	**P.M.**	
_____________	_____________	_____________	

* Single (morning) dose only

Patient Pill Diary for Dose Escalation for Intermittent ABT-888 Portion All Cycles

Patient Initials (FML): | | |

Start Date (mm/dd/yyyy): | | / | | / | | | |

NCI Protocol #: 7977 | Local Protocol # 1410014852 | Version: 06/06/2018

Accession # ________________________

Cycle Number ______________________ Dose _______________

Research Assistant Signature: _________________________________

Date: __________________

Patient Signature: ____

Date: __________________

Please carefully read and follow these instructions:

- Bring this Pill Diary and any remaining ABT-888 pills to clinic visits so your doctor can make sure that you are taking the pills correctly.
- Store the pills at room temperature at 15° to 25° C (59° to 77° F). Protect the pills from heat and moisture.
- Your doctor will tell you what ABT-888 dose you will be taking. You will be taking ABT-888 for 4 days and then have a break. *You can take the pills with or without meals.*

  If you miss taking a pill on time (a dose is missed) or you vomit after taking the pill, please do **not** repeat the dose.
- In the boxes below, note the **date** and **time** (under A.M. and P.M.) for each day you take the ABT-888 study drug pills:

Day 1 (in clinic)<br>**A.M.**<br>_____________<br>**P.M.**<br>_____________	Day 2<br>**A.M.**<br>_____________<br>**P.M.**<br>_____________	Day 3<br>**A.M.**<br>_____________<br>**P.M.**<br>_____________	Day 4<br>**A.M.**<br>_____________<br>**P.M.**<br>_____________	Day 5<br>Rest (no ABT-888 taken)	Day 6<br>Rest (no ABT-888 taken)	Day 7<br>Rest (no ABT-888 taken)
Day 8<br>**A.M.**<br>_____________<br>**P.M.**<br>_____________	Day 9<br>**A.M.**<br>_____________<br>**P.M.**<br>_____________	Day 10<br>**A.M.**<br>_____________<br>**P.M.**<br>_____________	Day 11 (in clinic for cycle 1 only)<br>**A.M.**<br>_____________<br>**P.M.**<br>_____________	Day 12<br>Rest (no ABT-888 taken)	Day 13<br>Rest (no ABT-888 taken)	Day 14<br>Rest (no ABT-888 taken)
Day 15<br>Rest (no ABT-888 taken)	Day 16<br>Rest (no ABT-888 taken)	Day 17<br>Rest (no ABT-888 taken)	Day 18<br>Rest (no ABT-888 taken)	Day 19<br>Rest (no ABT-888 taken)	Day 20<br>Rest (no ABT-888 taken)	Day 21<br>Rest (no ABT-888 taken)

APPENDIX I: ABT-888 Administration/Dispensing Schedule

Dose Escalation portion:

Cycle #	Study Day	ABT-888 Dose	ABT-888 Administration/Dispensing
1	3	Early	Administered in clinic immediately after PK sample drawn for timepoint occurring 48 h after start of CPT-11 infusion
		Late	Dispensed to patient
1	4-14	Early/Late	Dispensed to patient
2	-1	Early	Administered in clinic
		Late	**N/A**[1]
2	1	Early	Administered in clinic immediately after PK sample drawn for timepoint occurring 28 h after Cycle 2, Day -1 ABT-888 dosing
		Late	Dispensed to patient
2	2-7	Early/Late	Dispensed to patient
2	8	Early	Administered in clinic at the start of CPT-11 infusion
		Late	Administered in clinic immediately after PK sample drawn for timepoint occurring 10 h after start of CPT-11 infusion
2	9	Early	Administered in clinic immediately after PK sample drawn for timepoint occurring 28 h after start of CPT-11 infusion
		Late	Dispensed to patient
2	10	Early	Administered in clinic immediately after PK sample drawn for timepoint occurring 48 h after start of CPT-11 infusion
		Late	Dispensed to patient
2	11-14	Early/Late	Dispensed to patient
> 2	-1-14	Early/Late	Dispensed to patient

[1] Cycle 2, Day -1: Half of normal total daily dose of ABT-888 to allow for PK sampling - no late dose administered/dispensed.

Expansion portion:

Cycle #	Study Day	ABT-888 Dose	ABT-888 Administration/Dispensing
1	2	Early	Administered in clinic immediately after 24 hour blood draws for PBMCs and CTCs
		Late	Dispensed to patient
1	3-7	Early/Late	Dispensed to patient
1	8	Early	Administered in clinic at the start of CPT-11 infusion
		Late	Dispensed to patient following 8 hr blood draw for PBMCs and CTCs
1	9	Early	Administered in clinic immediately after the 24 hour blood draw for PBMCs and CTCs
		Late	Dispensed to patient
1	10-14	Early/Late	Dispensed to patient
1	15	Early	Administered in clinic 4 hrs before the CTC blood draw
		Late	Dispensed to patient
1	16-21	Early/Late	Rest (no treatment)
> 1	1-15	Early/Late	Dispensed to patient

Dose Escalation for Intermittent ABT-888 portion:

Cycle #	Study Day	ABT-888 Dose	ABT-888 Administration/Dispensing
Run-In	-14	Early	Administered in clinic after all assessments completed
		Late	Dispensed to patient
Run-In	-13 & -12	Early/Late	Dispensed to patient
Run-In	-11	Early	Administered in clinic (within 4 hours prior to biopsy)
		Late	**N/A**[1]
1	1	Early	Administered in clinic after all assessments completed
		Late	Dispensed to patient
1	2-4	Early/Late	Dispensed to patient
1	5-7	Early/Late	Rest (no treatment)
1	8-10	Early/Late	Dispensed to patient
1	11	Early	Administered in clinic (within 4 hours prior to biopsy)
		Late	Dispensed to patient
1	12-21	Early/Late	Rest (no treatment)
2+	1	Early	Administered in clinic after assessments completed
		Late	Dispensed to patient
2+	2-4	Early/Late	Dispensed to patient
2+	5-7	Early/Late	Rest (no treatment)
2+	8-11	Early/Late	Dispensed to patient
2+	12-21	Early/Late	Rest (no treatment)

[1] Run-In Day -11: Half of normal total daily dose of ABT-888 to allow for biopsy - no late dose administered/dispensed.

APPENDIX J: Standard Operating Procedure (SOP) for Collection and Preparation of Circulating Tumor Cells (CTCs) for γH2AX-Assays

Title:	**CELLSAVE TUBE HUMAN BLOOD COLLECTION AND SPECIMEN SUBMISSION**				Page 1 of 6
Doc. #:	LHTP003.08.03	Revision:	00	Effective Date:	06/01/2011

Pharmacodynamic Assay Development and Implementation Section
Laboratory of Human Toxicology & Pharmacology (LHTP)
Applied Developmental Directorate
SAIC-Frederick, Inc.
NCI-Frederick Cancer Research

Originator: [redacted] Date: 06/01/2011__

Review: _Dr. [redacted]_________ Date: _______06/01/2011___

QA Review: __[redacted] Date: _______06/01/2011___

Approval: _Dr. [redacted]_________________________ Date: ___________________

Change History

**Revision**	**Approval Date**	**Description**	**Originator**	**Approval**
00		New Document	LW	

Title:	**CELLSAVE TUBE HUMAN BLOOD COLLECTION AND SPECIMEN SUBMISSION**				Page 2 of 6
Doc. #:	LHTP003.08.03	Revision:	00	Effective Date:	06/01/2011

1.0 PURPOSE

The purpose of this SOP is to describe the procedures for collection, preparation, and shipment of human blood specimens from clinical centers.

2.0 SCOPE

This SOP applies to all clinical center personnel responsible for collecting, preparing and shipping blood specimens from patients participating in clinical trials.

3.0 HEALTH & SAFETY

Handle all biological specimens and blood collection sharps according to the policies and procedures of the company. Use personal protective equipment to protect against blood spills and leaks and bloodborne pathogens.

4.0 RESPONSIBILITIES

4.1 It is the responsibility of the Clinical Site Coordinator to assure compliance with this procedure.

4.2 It is the responsibility of the Research Technician to execute this procedure.

5.0 MATERIALS AND EQUIPMENT REQUIRED

5.1 CellSave® Preservative Tubes, 10.0 ml capacity (Veridex)

5.2 ApoCell specimen collection kit:
- Styrofoam box
- Shipping cardboard box
- CRT packs
- Package labels

5.3 Blood shipping tube holder or a 50 ml Falcon centrifuge tube

5.4 Patient specimen labels

6.0 OPERATING PROCEDURES

6.1 Collection of specimens into CellSave® Preservative Tubes

6.1.1 Prepare labels for each specimen to be collected.

6.1.2 Draw the initial sample prior to initiation of a therapy regimen. Subsequent samples can be drawn after the start of a therapy regimen, usually at 3 to 4 week intervals, to follow CTC levels during therapy.

6.1.3 If the patient is on doxorubicin therapy, allow at least 7 days following administration of a dose of doxorubicin before blood draw.

6.1.4 Collect the whole blood aseptically by venipuncture or from a venous port into a CellSave® Preservative Tube only.

6.1.5 Fill a minimum of **8 ml volume for a 10 ml capacity tube** until the blood flow stops to ensure the correct ratio of sample to anticoagulant and preservative.

6.1.6 Immediately mix the specimen tube by gently **inverting** the tube eight times. Tube inversion prevents clotting. Inadequate or delayed mixing may result in inaccurate test results.

6.1.7 Specimens must be processed within 96 hours of collection.

6.1.8 Blood specimens may be stored or transported in CellSave® Preservative Tubes for up to 96 hours at room temperature (15 to 30°C) prior to processing. Do not refrigerate or freeze specimens.

6.1.9 **Label** each tube with specimen ID and type, time point – cycle and day number, sample date / time and operator's initials.

6.1.10 **SPECIMENS MUST BE SHIPPED WITHIN 24 HOURS OF COLLECTION** to ensure quality results, and SAIC is to be notified by email on the day of shipment.

6.1.11 Complete the appropriate section of the **Specimen Submission Form** (Attachment # 1) for each shipment.

6.2 **Packaging Instructions DO NOT SHIP ON A THURSDAY, FRIDAY or SATURDAY.**

6.2.1 Store CRT packs at room temperature (15 - 30°C).
DO NOT REFRIGERATE OR FREEZE.

6.2.2 Place a room temperature CRT pack in the bottom of the original Styrofoam shipping container.

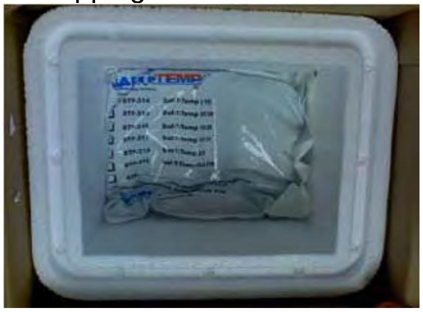

6.2.3 Wrap absorbent paper around the specimen filled CellSave® Preservative tube and place in a 50 ml Falcon centrifuge tube or blood shipping tube.

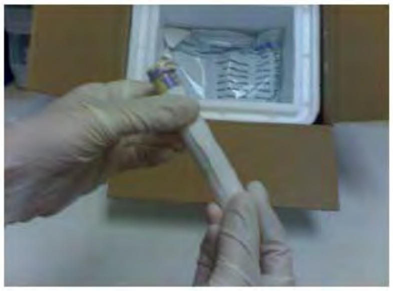

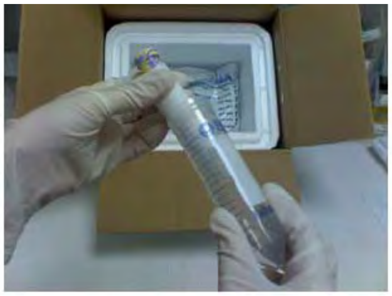

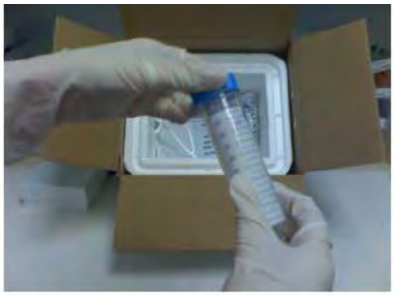

6.2.4 Place the tube in the Styrofoam box on top of the CRT pack.

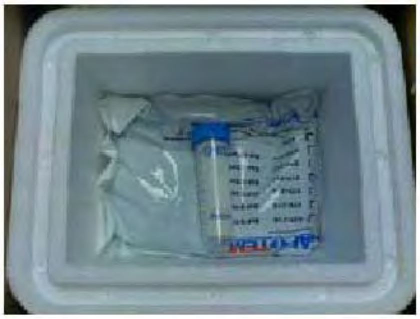

6.2.5 Put a second room temperature CRT pack on top of the tube.

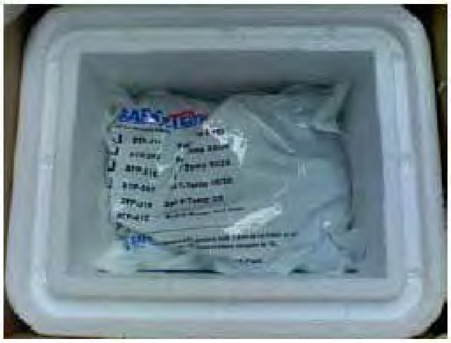

6.2.6 Insert the completed Specimen Submission Form in the box on top of the CRT gel pack.

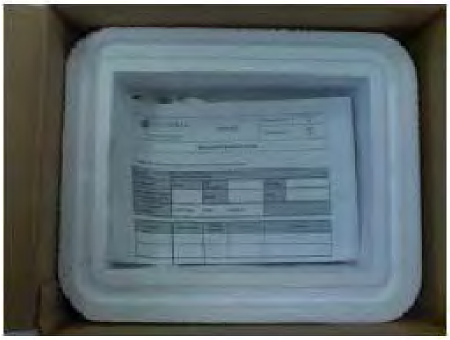

6.2.7 Close the styrofoam box and place in the shipping cardboard box.

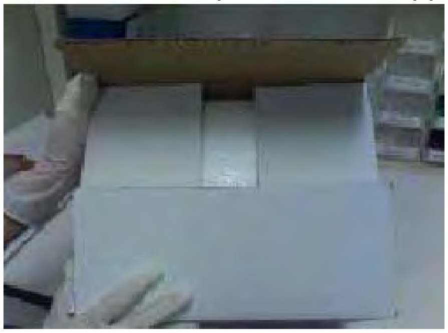

6.2.8 Seal and adhere the return shipping labels to the outside of the box.

6.2.9 Notify SAIC contact person of the shipment by email.

6.3 **Shipping Instructions SPECIMENS MUST BE SHIPPED ON DAY OF COLLECTION**

6.3.1 Contact Information:

[redacted]

6.3.2 Please call and send an email on the day of shipping to

[redacted]

6.3.3 **PLEASE SHIP SPECIMEN TO:**

Dr [redacted]
PADIS, LHTP
FNLCR/SAIC-Frederick, Inc.
1050 Boyles Street
[redacted]
Frederick, MD 21702-1201
U.S.A.

SAIC-Frederick / NCI-Frederick
Pharmacodynamic Assay Development and Implementation Section
Laboratory of Human Toxicology & Pharmacology (LHTP)
1050 Boyles Street, Frederick, MD 21702-1201
Telephone: 301-846-6870

CELLSAVE TUBE HUMAN BLOOD COLLECTION SPECIMEN SUBMISSION FORM

Complete the information below and submit with specimens:						
Protocol #:						
Total # Specimens:			Ship Date:			
Specimen ID / Specimen Type		Date of Collection	Time of Collection	Cycle#	Day#	Comments

If submitting more specimens than this space allows for, please attach a roster or spread sheet of specimens.

Specimen(s) prepared by____________________________________ Date:________________

Appendix K: Research Blood Shipping Manifest

RESEARCH BLOOD SPECIMEN COLLECTION AND SHIPPING

1. Information on Pre-printed Label for Research Blood Samples

- **CTEP Protocol Number (NCI#7977)**
- **Patient Study ID Number**
- **Date of Collection**
- **Time of Collection**

2. Sample Shipping Manifest

**Ship From:**<br>**Laboratory PI**<br>**Address**<br><br>**Contact Name**<br>**Contact Phone**<br>**Contact Email**	**NCI#7977**<br>**Yale University**<br>**Shipping Manifest**	**Ship To:**<br>Laboratory of Dr. [redacted]<br>310 Cedar St. BML113, Department of Pathology<br>Yale School of Medicine<br>New Haven, CT 06520-8023<br>**Phone:** [redacted]<br>**Email:** [redacted]
**Shipping Date:**<br>**Clinical Protocol:**	**Carrier:**<br>**Shipping #:**	

In Package	#	Patient Study ID #	Description	Collection Time	Collection Date MM/DD/YYYY
☐	*Test*	*21*	*Blood*	*14:15*	*03/01/2010*
☐	1				
☐	2				
☐	3				
☐	4				
☐	5				
☐	6				
☐	7				
☐	8				
☐	9				
☐	10				

3. Notify the following people prior to shipping:

[redacted]

Appendix L: Frozen Tissue Shipping Manifest

TISSUE SPECIMEN COLLECTION AND SHIPPING

1. Information on Pre-printed Label for Frozen Tissue Samples

- **CTEP Protocol Number (NCI#7977)**
- **Collection Visit Time Point: (BL/RI/C1D11)**
    - (BL=baseline; RI=run in; C1D11 = cycle 1 day 11)
- **Patient Study ID Number**
- **Tissue Core Number**
- **Date of Collection**

2. Sample Shipping Manifest

**Ship From:**<br>**Laboratory PI**<br>**Address**<br><br><br><br>**Contact Name**<br>**Contact Phone**<br>**Contact Email**	**NCI#7977**<br>**Yale University**<br>**Shipping Manifest**	**Ship To:**<br>Clinical Research Support Lab<br>Yale University<br>Smilow Cancer Hospital<br>35 Park St, room [redacted]<br>Attn: [redacted]<br>New Haven, CT 06510<br>**Phone:** [redacted]<br>**Email:** [redacted]

Shipping Date: **Carrier:**
Clinical Protocol: **Shipping #:**

In Package	#	Patient Study ID #	Tissue Core Number	Description	Collection Visit Time Point	Collection Time	Collection Date MM/DD/YYYY
☐	*Test*	*21*	*2 of 6*	*Tumor*	*BL*	*14:15*	*03/01/2010*
☐	1						
☐	2						
☐	3						
☐	4						
☐	5						
☐	6						
☐	7						
☐	8						
☐	9						
☐	10						

3. Notify the following people prior to shipping:

[redacted]

Appendix M: Formalin-Fixed, Paraffin-Embedded Tissue Shipping Manifest

FORMALIN-FIXED, PARAFFIN-EMBEDDED TISSUE SPECIMEN COLLECTION AND SHIPPING

1. Information on Pre-printed Label for Formalin-Fixed, Paraffin-Embedded Tissue Samples

- **CTEP Protocol Number (NCI#7977)**
- **Collection Visit Time Point: (BL/RI/C1D11)**
    - o (BL=baseline; RI=run in; C1D11 = cycle 1 day 11)
- **Patient Study ID Number**
- **Tissue Core Number**
- **Date of Collection**

2. Sample Shipping Manifest

<table>
<tr><td><u>Ship From:</u>
Laboratory PI
Address

Contact Name
Contact Phone
Contact Email</td><td colspan="3">NCI#7977
Yale University
Shipping Manifest</td><td colspan="4"><u>Ship To:</u>
Attention: [redacted]
[redacted] Lab, Department of Pathology
Yale University
310 Cedar Street, BML112
New Haven, CT 06520-8023
Phone: [redacted]
Email: [redacted]</td></tr>
<tr><td colspan="4">Shipping Date:
Clinical Protocol:</td><td colspan="4">Carrier:
Shipping #:</td></tr>
<tr><td>In Package</td><td>#</td><td>Patient Study ID #</td><td>Tissue Core Number</td><td>Description</td><td>Collection Visit Time Point</td><td>Collection Time</td><td>Collection Date MM/DD/YYYY</td></tr>
<tr><td>☐</td><td>Test</td><td>21</td><td>2 of 6</td><td>Tumor</td><td>BL</td><td>14:15</td><td>03/01/2010</td></tr>
<tr><td>☐</td><td>1</td><td></td><td></td><td></td><td></td><td></td><td></td></tr>
<tr><td>☐</td><td>2</td><td></td><td></td><td></td><td></td><td></td><td></td></tr>
<tr><td>☐</td><td>3</td><td></td><td></td><td></td><td></td><td></td><td></td></tr>
<tr><td>☐</td><td>4</td><td></td><td></td><td></td><td></td><td></td><td></td></tr>
<tr><td>☐</td><td>5</td><td></td><td></td><td></td><td></td><td></td><td></td></tr>
<tr><td>☐</td><td>6</td><td></td><td></td><td></td><td></td><td></td><td></td></tr>
<tr><td>☐</td><td>7</td><td></td><td></td><td></td><td></td><td></td><td></td></tr>
<tr><td>☐</td><td>8</td><td></td><td></td><td></td><td></td><td></td><td></td></tr>
<tr><td>☐</td><td>9</td><td></td><td></td><td></td><td></td><td></td><td></td></tr>
<tr><td>☐</td><td>10</td><td></td><td></td><td></td><td></td><td></td><td></td></tr>
</table>

3. Notify the following people prior to shipping:

[redacted]

APPENDIX N: PATIENT DRUG INFORMATION HANDOUT AND WALLET CARD

Information for Patients, Their Caregivers and Non-Study Healthcare Team on Possible Interactions with Other Drugs and Herbal Supplements

[Note to investigators: This appendix consists of an "information sheet" to be handed to the patient at the time of enrollment. Use or modify the text as appropriate for the study agent, so that the patient is aware of the risks and can communicate with their regular prescriber(s) and pharmacist. A convenient wallet-sized information card is also included for the patient to clip out and retain at all times. If you choose to use them, please note that the information sheet and wallet card will require IRB approval before distribution to patients.]

The patient ____________________________ is enrolled on a clinical trial using the experimental study drug **ABT-888 (veliparib)**. This clinical trial is sponsored by the National Cancer Institute. This form is addressed to the patient, but includes important information for others who care for this patient.

These are the things that you as a prescriber need to know:

ABT-888 (veliparib) may interact with certain transporter proteins that help move drugs in and out of cells.

- The proteins in question are ***OCT1/2, OATPs, MATE1, MATE2K, and P-gp.*** ABT-888 (veliparib) is a substrate of P-gp, OCT2, and MATE1/2K and may be affected by other drugs that inhibit these protein transporters. ABT-888 is an inhibitor of OATPs, OCT1/2, and MATE1/2K, and may affect transport of other drugs in and out of cells.

To the patient: Take this paper with you to your medical appointments and keep the attached information card in your wallet.

ABT-888 (veliparib) may interact with other drugs which can cause side effects. For this reason, it is very important to tell your study doctors of any medicines you are taking before you enroll onto this clinical trial. It is also very important to tell your doctors if you stop taking any regular medicines, or if you start taking a new medicine while you take part in this study. When you talk about your current medications with your doctors, include medicine you buy without a prescription (over-the-counter remedy), or herbal supplements such as St. John's Wort. It is helpful to bring your medication bottles or an updated medication list with you.

Many health care providers can write prescriptions. You must tell all of your health care providers (doctors, physician assistants, nurse practitioners, or pharmacists) you are taking part in a clinical trial.

These are the things that you and they need to know:

Use caution when administering ABT-888 (veliparib) with other medicines that need certain **transport protein to be effective or to be cleared from your system.** Before you enroll onto the clinical trial, your study doctor will work with your regular health care providers to review any medicines and herbal supplements.

- Please be very careful! Over-the-counter drugs (including herbal supplements) may contain ingredients that could interact with your study drug. Speak to your doctors or pharmacist to determine if there could be any side effects.
- Your regular health care provider should check a frequently updated medical reference or call your study doctor before prescribing any new medicine or discontinuing any medicine. Your study doctor's name is

_____________________________________ and he or she can be contacted at

_____________________________________. June 2017

STUDY DRUG INFORMATION WALLET CARD

You are enrolled on a clinical trial using the experimental study drug **ABT-888 (veliparib).** This clinical trial is sponsored by the NCI**.** **ABT-888 (veliparib)** may interact with drugs that need certain transport proteins in your body. Because of this, it is very important to:

- Tell your doctors if you stop taking any medicines or if you start taking any new medicines.
- Tell all your health care providers (doctors, physician assistants, nurse practitioners, or pharmacists) that you are taking part in a clinical trial.
- Check with your doctor or pharmacist whenever you need to use an over-the-counter medicine or herbal supplement.

Use caution as ABT-888 (veliparib) may interact with medicines that stop transport proteins **MATE1/2K, OCT2, and P-gp** to process further in the body**.**

- Before you enroll onto the clinical trial, your study doctor will work with your regular health care providers to review any medicines and herbal supplements.
- Before prescribing new medicines, your regular prescribers should go to a frequently-updated medical reference for a list of drugs to avoid, or contact your study doctor.
- Your study doctor's name is

  ____

  and can be contacted at _________________________.
